# Transition-metal-free C(sp^3^)–H/C(sp^3^)–H dehydrogenative coupling of saturated heterocycles with *N*-benzyl imines†

**DOI:** 10.1039/d0sc00031k

**Published:** 2020-03-31

**Authors:** Zhengfen Liu, Minyan Li, Guogang Deng, Wanshi Wei, Ping Feng, Quanxing Zi, Tiantian Li, Hongbin Zhang, Xiaodong Yang, Patrick J. Walsh

**Affiliations:** Key Laboratory of Medicinal Chemistry for Natural Resources, Ministry of Education and Yunnan Province, State Key Laboratory for Conservation and Utilization of Bio-Resources in Yunnan, School of Chemical Science and Technology, Yunnan University Kunming 650091 P. R. China xdyang@ynu.edu.cn zhanghb@ynu.edu.cn; Roy and Diana Vagelos Laboratories Penn/Merck Laboratory for High-Throughput Experimentation Department of Chemistry, University of Pennsylvania 231 South 34th Street Philadelphia PA USA pwalsh@sas.upenn.edu liminyan@sas.upenn.edu; Department of Soil and Water Science, University of Florida 2181 McCarty Hall A Gainesville FL 32611-0290 USA

## Abstract

A unique C(sp^3^)–H/C(sp^3^)–H dehydrocoupling of *N*-benzylimines with saturated heterocycles is described. Using super electron donor (SED) 2-azaallyl anions and aryl iodides as electron acceptors, single-electron-transfer (SET) generates an aryl radical. Hydrogen atom transfer (HAT) from saturated heterocycles or toluenes to the aryl radical generates alkyl radicals or benzylic radicals, respectively. The newly formed alkyl radicals and benzylic radicals couple with the 2-azaallyl radicals with formation of new C–C bonds. Experimental evidence supports the key hydrogen-abstraction by the aryl radical, which determines the chemoselectivity of the radical–radical coupling reaction. It is noteworthy that this procedure avoids the use of traditional strong oxidants and transition metals.

## Abstract 2

A unique transition-metal-free C(sp^3^)–H/C(sp^3^)–H dehydrocoupling of *N*-benzylimines with saturated heterocycles is presented using 2-azaallyl anions as super electron donors to initiate the generation of hydrogen atom abstracting aryl radicals.

## Graphical abstract

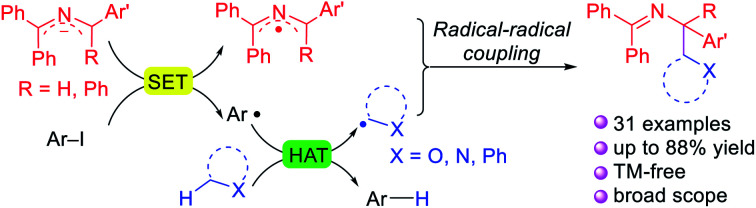

## Introduction

Cyclic ethers and amines are fundamental structural motifs in nature and are found in a vast number of bioactive compounds, small-molecule drugs^1^ and functional materials. As such, tremendous effort has been devoted to their synthesis.^2^ Despite significant progress in the synthesis of saturated heterocycles, approaches to their C–H activation and subsequent C–C bond formation remain in great demand. The high C–H bond dissociation energies (∼90–100 kcal mol^−1^)^3^ and low acidity (p*K*_a_'s usually >45) of saturated heterocycles have significantly hindered the development of methods to functionalize these robust C(sp^3^)–H bonds.

**Scheme 1:**
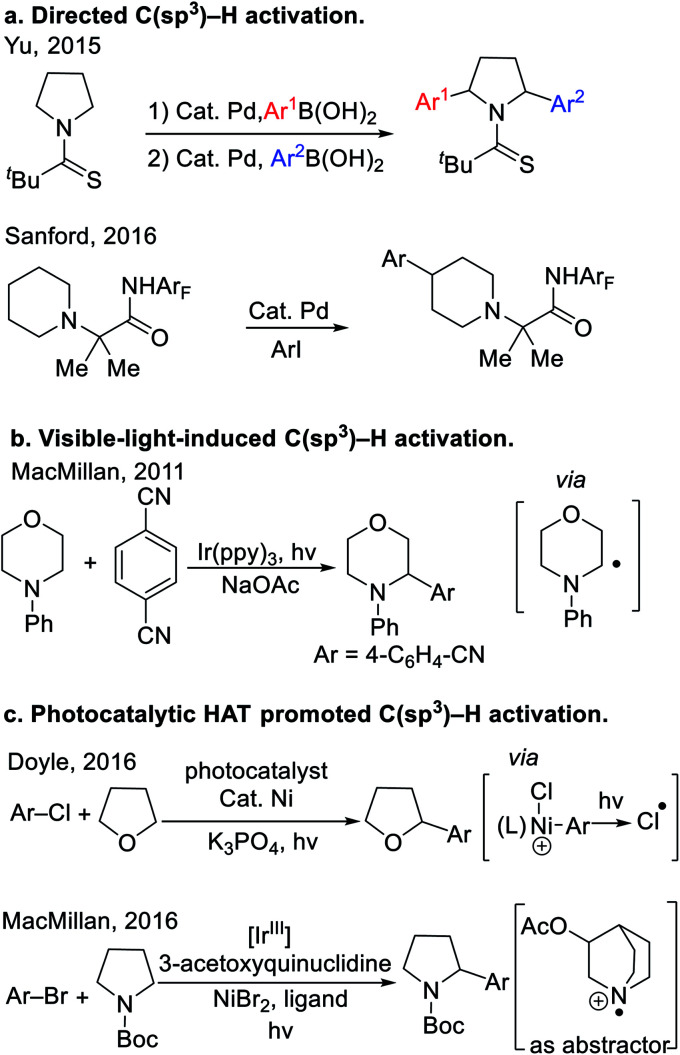
General strategies in C(sp^3^)–H activation of heterocyclic alkanes. (a) Directed C(sp^3^)–H activation. (b) Visible-light-induced C(sp^3^)–H activation. (c) Photocatalytic HAT promoted C(sp^3^)–H activation.

Recently, a variety of tactics have been introduced to tackle the functionalization of saturated heterocycles. Directing group facilitated C(sp^3^)–H activation has evolved into an enabling strategy to functionalize heterocycles. Recently, Yu^4^ and Sanford^5^ developed the arylation of nitrogen-containing heterocycles with the aid of thioamide and perfluorinated amide directing groups (Scheme 1a). In these examples, the C–H bonds of the substrates were cleaved by Pd catalysts, presumably through a concerted metalation-deprotonation (CMD) mechanism.^6^ These reactions are quite challenging, and even with highly optimized directing groups, require very high reaction temperatures (150 °C). Since 2011, visible-light-induced C–H bond functionalization has emerged as a powerful strategy for the activation of heterocycles,^7^ as shown in the representative example^8^ in Scheme 1b. Mechanistically, photoredox-driven single-electron transfer and α-C–H deprotonation enabled the activation of C–H bonds adjacent to nitrogen. Other strategies to activate the C(sp^3^)–H bonds of heterocycles include application of hydrogen atom transfer (HAT).^9^ For example, Porta *et al.* developed a radical coupling where HAT of aryl radical from THF was the key step for radical generation. More recently, Doyle and co-workers found that photolysis of a Ni(iii)–Cl intermediate generated chlorine radicals that underwent HAT with THF^10^ to achieve a unique THF arylation (Scheme 1c). In the same year, MacMillan and co-workers^11^ used photoredox catalysis to oxidize 3-acetoxyquinuclidine to the radical cation, which promoted HAT with cyclic amines (Scheme 1c). Wu's team^12^ developed a Eosin Y photocatalytic HAT activation of tetrahydrofurans.

**Scheme 2:**
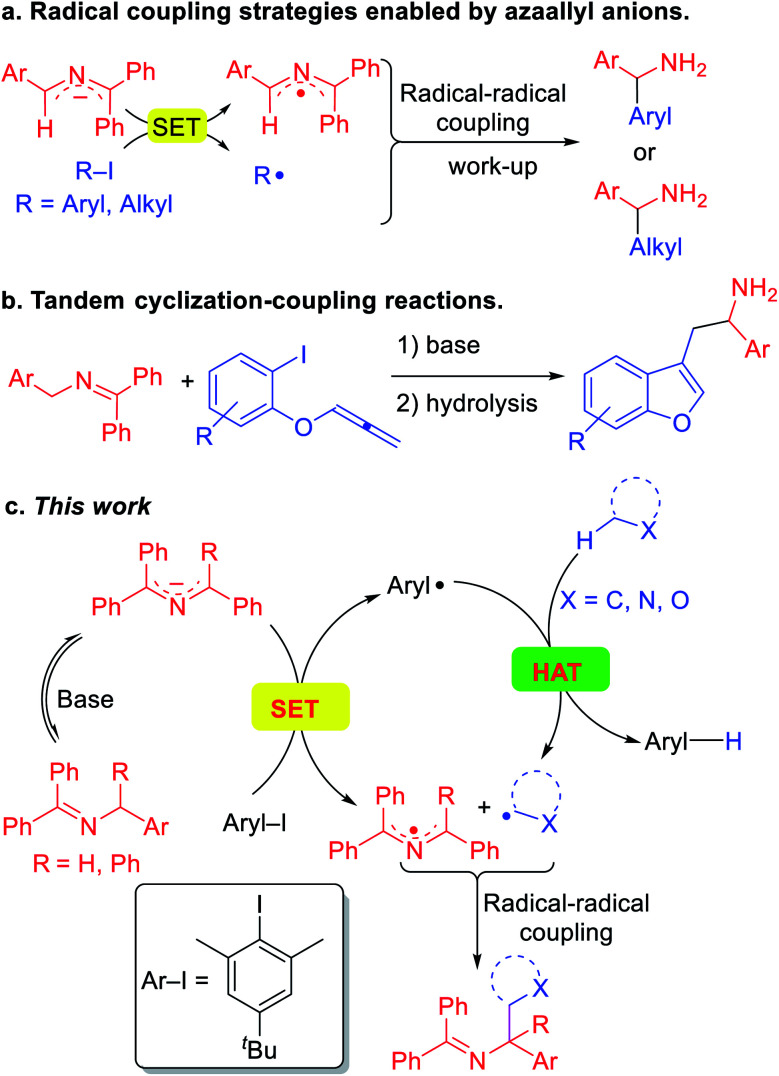
Application of 2-azaallyl anions in radical coupling reactions. (a) Radical coupling strategies enabled by 2-azaallyl anions as super-electron-donors. (b) Tandem reactions for the synthesis of heterocycles. (c) Radical relay design for the synthesis of heterocyclic amine derivatives.

These impressive and impactful advances inspired us to ponder the prospect of exploiting so called super electron donors (SED)^13^ to functionalize C(sp^3^)–H bonds in the absence of transition metals or photoredox catalysts. Our team has been pursuing the fascinating chemistry of 2-azaallyl anions,^14^ which are easily generated by deprotonation of readily accessible *N*-benzyl benzophenone imines.^15^ We identified the inherent reducing nature of the highly colored 2-azaallyl anions, which enabled the introduction of a series of transition metal free coupling reactions (Scheme 2a). In its simplest form, the 2-azaallyl anion reduces aryl iodides or tertiary alkyl iodides and bromides to generate aryl or alkyl radicals and the 2-azaallyl radical.^14*f*^ These two radicals then undergo selective coupling with each other to generate a new C–C bond, providing diarylmethyl amines and benzylic amines after hydrolysis. More sophisticated radical cyclization/coupling reactions to afford benzofurylethylamines (Scheme 2b) and their isochromene analogues have also been accomplished.^14*a*,*b*^ Given the high reactivity of aryl radicals, we hypothesized that if the aryl radical^16^ coupling with the 2-azaallyl radical in Scheme 2a could be sufficiently slowed, perhaps the aryl radical would preferentially undergo HAT with other organic compounds possessing weaker C(sp^3^)–H bonds. Such a HAT would generate new radicals that we envisioned would couple with the 2-azaallyl radical (Scheme 2c). The net result would be a cross-dehydrogenative coupling reaction^17^ between two C(sp^3^)–H bonds to form a C(sp^3^)–C(sp^3^) bond under mild and transition metal-free conditions. Although much of this study is dedicated to mapping the reactivity of the HAT and coupling steps, the synthetic utility of this process is also explored. The products of these reactions are heterocyclic methylamine derivatives, which are of importance in the pharmaceutical industry,^18^ but would be otherwise difficult to access under mild conditions.

Herein, we report a unique activation strategy of relatively inert heterocyclic C(sp^3^)–H bonds. The strategy is founded on the reducing character of 2-azaallyl anions and the HAT abstraction feature of aryl radicals (Scheme 2c). To inhibit the aryl radical from coupling with the 2-azaallyl radical, as in Scheme 2a, a sterically protected aryl radical precursor was employed and found to undergo single-electron-transfer with diverse 2-azaallyl anions. The resulting hindered aryl radical effectively abstracts hydrogen from the heterocycles' C(sp^3^)–H bond, generating alkyl radicals in a reaction akin to an intermolecular radical translocation process.^19^ The coupling of alkyl radicals and 2-azaallyl radicals furnishes valuable heterocyclic benzylic amines. This HAT protocol employs a sacrificial aryl radical to overcome the limitation of redox potentials and acidities. Finally, we disclose that toluene derivatives can also be activated and coupled with 2-azaallyl intermediates in this transformation.

## Results and discussion

With the mechanistic hypothesis in Scheme 2c in mind, we first investigated the aryl iodides as precursors to hydrogen abstractors. The benzophenone imine **1a** and tetrahydrofuran solvent (**2a**) were chosen as the model substrates for the coupling studies at 80 °C (Table 1). When iodobenzene **3a** was used, the target coupling product was obtained in an overall 35% assay yield (AY, determined by ^1^H NMR integration against an internal standard). We next examined substituent effects on the aryl motif. The electron donating alkyl of 1-(*tert*-butyl)-4-iodobenzene (**3b**) resulted in an increase in the overall assay yield to 56%. The more electron donating 4-OMe (**3c**) or halogenated 4-Cl (**3d**) exhibited decreased AY of 46 and 35%, respectively. Although it is known that the phenyl radical will react with THF very quickly (*k* = 4.8 × 10^6^ M^−1^ s^−1^),^20^ coupling products derived from reaction of the aryl radical with the 2-azaallyl radical were still observed (10–20% AY). To discourage the direct coupling event, sterically hindered substrates possessing two *ortho*-substituents were examined. While 2-iodo-1,3-dimethyl benzene (**3e**) afforded little improvement (combined AY of 43%), addition of a *tert*-Bu group, which proved beneficial in the reaction of **3b***vs.***3a**, again resulted in an increase in the AY. Thus, 5-(*tert*-butyl)-2-iodo-1,3-dimethylbenzene (**3f**) reacted to generate the coupling products with a combined AY of 59%. Further increasing the size of the flanking substituents on the aryl radical to isopropyl (**3g**) led to a combined AY of 48%. As might be expected, neopentyl iodide (**3h**) as HAT precursor did not participate in the C–H activation pathway, implying that sacrificial HAT abstractors derived from C(sp^3^)–X bonds are not viable in our approach.

**Table 1:** Effect of aryl iodide electron-acceptors and hydrogen abstractor precursors^a^

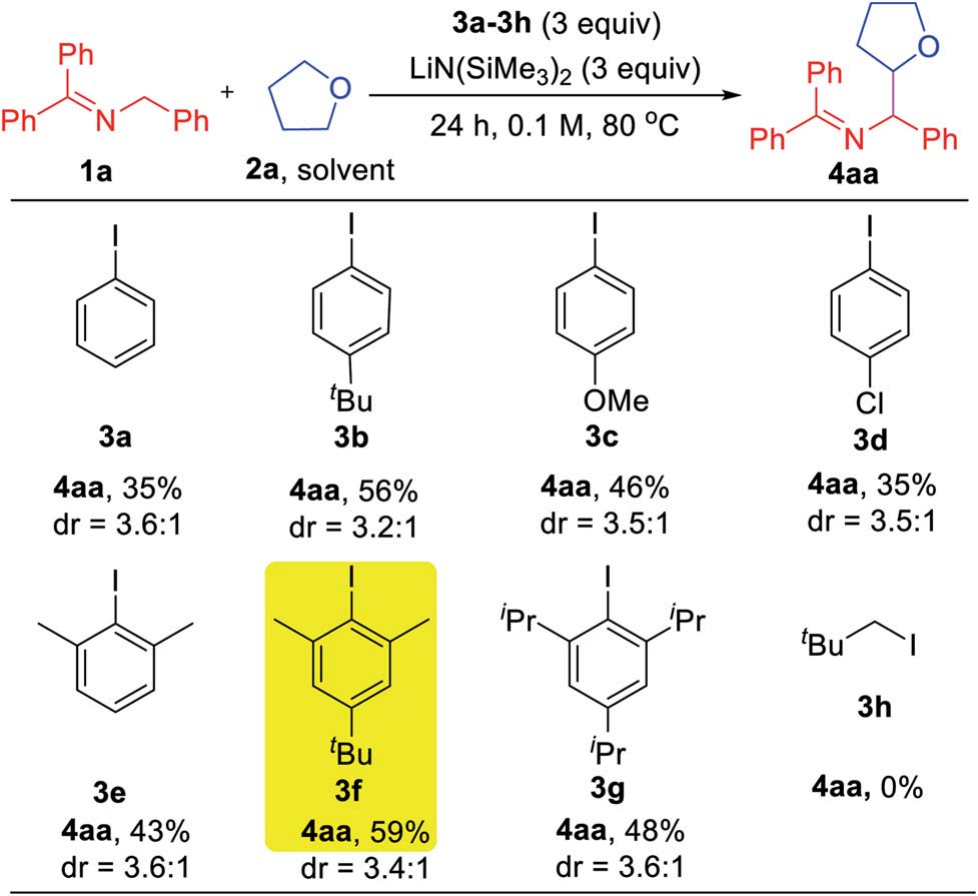

a Reactions conducted on a 0.1 mmol scale. Assay yield determined by ^1^H NMR spectroscopy of the crude reaction mixtures using C_2_H_2_Cl_4_ as an internal standard.

Based on these initial studies, we continued the optimization with aryl iodide **3f** as the hydrogen abstractor precursor. We next set out to determine the optimal reaction time and found that decreasing from 24 h to 1 h led to an increase in the AY from 49 to 73% (Table 2, entry 1–5). Replacing LiN(SiMe_3_)_2_ with NaN(SiMe_3_)_2_ led to an overall 81% AY (entry 6). For reasons that are not clear, using KN(SiMe_3_)_2_ resulted in formation of a complex mixture with no expected product detected (entry 7).

**Table 2:** Optimization studies of the coupling of ketimine **1a** and tetrahydrofuran **2a**^a^^,^^b^

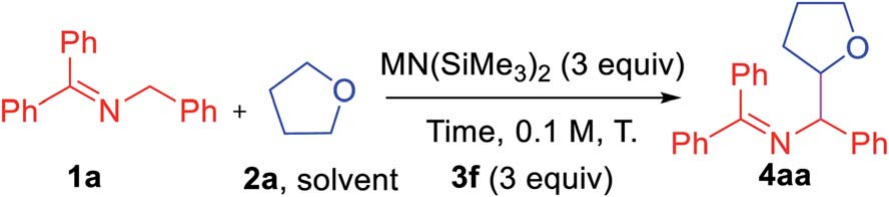				
Entry	M =	Time/h	*T*/°C	Yield of **4aa**/%
1	Li	24	80	49 (dr = 3.4 : 1)
2	Li	12	80	58 (dr = 2.8 : 1)
3	Li	6	80	64 (dr = 2.7 : 1)
4	Li	3	80	68 (dr = 2.8 : 1)
5	Li	1	80	73 (dr = 2.8 : 1)
6	Na	1	80	81 (dr = 1.6 : 1)
7	K	1	80	Complex mixture
8	Na	1	100	52 (dr = 1.5 : 1)
9	Na	1	60	69 (dr = 1.7 : 1)
10^c^	Na	1	80	81 (dr = 1.7 : 1)
11^c^^,^^d^	Na	1	80	84 (80)^e^ (dr = 1.7 : 1)

a Reactions conducted on a 0.1 mmol scale. Assay yields determined by ^1^H NMR spectroscopy of the crude reaction mixture using C_2_H_2_Cl_4_ as an internal standard.

b Diastereomeric ratio (dr) of alpha coupling product between **1a** and **2a** determined by HPLC. The beta coupling product was observed in trace amounts by HPLC but the dr could not be determined.

c 0.2 M.

d **3f** (2 equiv.).

e Isolated yield and diastereomeric ratio after chromatographic purification.

Under the same conditions with NaN(SiMe_3_)_2_ but increasing or decreasing the temperature resulted in diminished AY (entries 8–9). Finally, tuning the concentration to 0.2 M and decreasing the aryl iodide **3f** to 2 equiv. afforded product **4aa** in overall 84% assay yield and 80% isolated yield (dr = 1.7 : 1, entry 11).

In an effort to explore the scope of this transformation, we first examined saturated heterocycles. In general, we found that a wide variety of substrates with ring sizes ranging from 4 to 6 underwent the C–H functionalization/coupling process (54–87% isolated yields). The scope of the reaction is outlined in Table 3. With more expensive substrates, we explored the coupling reactions in benzene solvent. Thus, 5 equiv. of 3,3-dimethyloxetane (**2b**) coupled with ketimine **1a** in 56% yield (2.8 : 1 dr). As for the dioxolanes, 1,4-dioxane (**2c**) furnished 54% yield (1.4 : 1 dr) of the product isomers. The acyclic analogue 1,2-dimethoxyethane (DME, **2d**) was found to couple with ketimine **1a** in 73% yield exclusively at the methylene. The symmetrical 1,3,5-trioxane **2e** participated in the coupling reaction to furnish the protected α-amino aldehyde **4ae** in 58% yield. We were also interested in exploring selectivity between C–H's positioned α to oxygen *vs.* α to nitrogen. We were pleased to find that coupling of *N*-methylmorpholine **2f** provided the coupling product **4af** in 55% yield, with coupling observed only adjacent to the amino group (3.2 : 1 dr). Likewise, *N*-methyl pyrrolidine **2g**, *N*-phenyl pyrrolidine **2h** and *N*-phenyl piperidine **2i** coupled in 56, 81 and 87% yields (1 : 1, 4.1 : 1 and 1.9 : 1 dr), respectively. Moreover, *N*-methyl pyrrole **2j** afforded product **4aj** in 77% yield. Finally, 5 equiv. of *N*,*N*-dimethylaniline (**2k**) in benzene coupled with ketimine **1a** in 58% yield. These latter two examples highlight the potential utility of this chemistry for the synthesis of diamines. Coupling products with the following H-atom donor either failed or gave trace products: DMF, DMA, *N*,*N*-dimethylbenzamide and 1,3-dimethylimidazolidin-2-one afforded only tract product. Sulfur-containing substrates, such as tetrahydrothiophene and DMSO did not lead to coupling products under the standard conditions.

**Table 3:** Scope of saturated heterocycles^a^

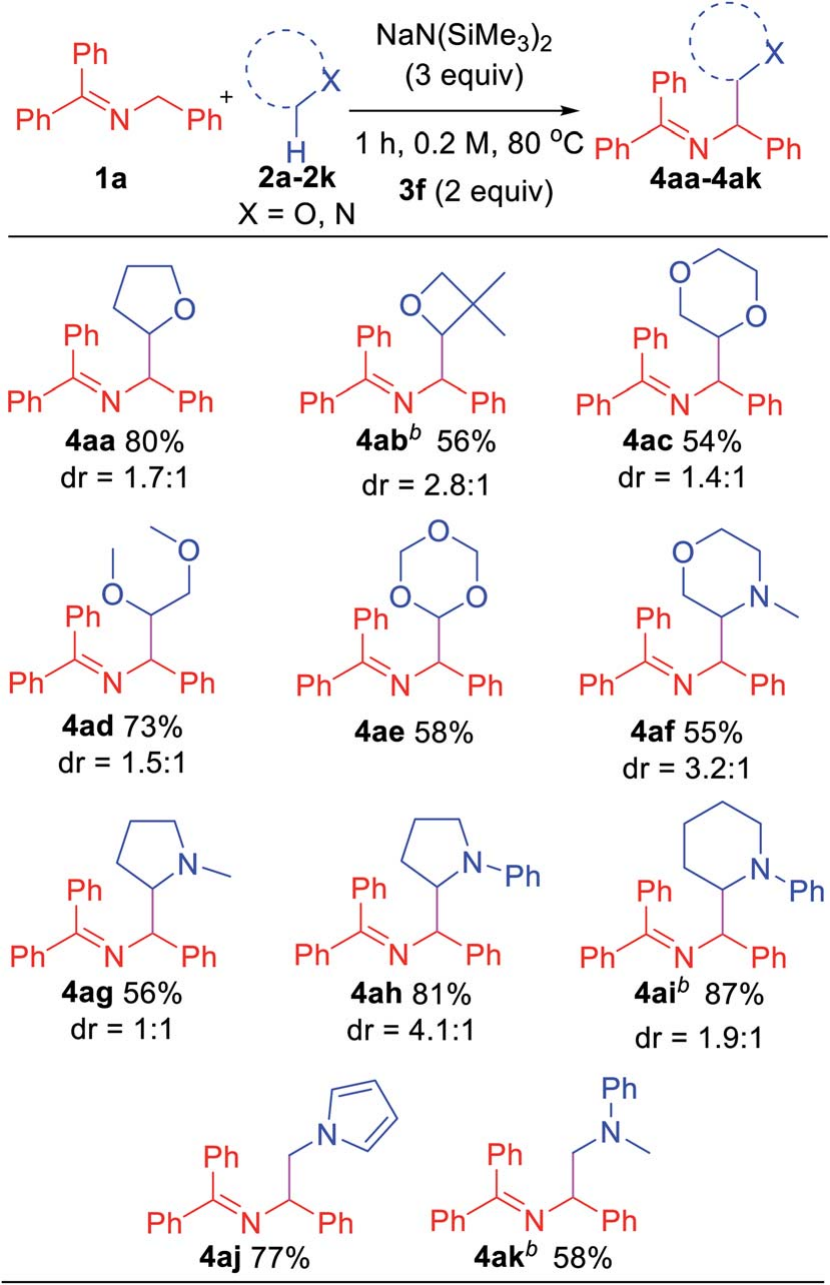

a Reactions conducted on 0.6 mmol scale using 1 equiv. of **1a**. Isolated yields and diastereomeric ratios after chromatographic purification.

b Benzene was used as the solvent with 5 equiv. substrate.

Encouraged by the results with saturated heterocycles, we selected tetrahydrofuran **2a** as coupling partner and investigated the imine scope. As shown in Table 4, the reaction proceeded with good overall yields (up to 88%). *N*-Benzyl imines bearing substituents on the arene, such as 4-*t*Bu (**1b**), generated the product **4ba** in 81% yield. For electron donating substituents (**1c**, 4-OMe and **1d**, dioxol), good yields of the coupled products were obtained (**4ca**, 84% yield and **4da**, 75% yield). The halide substituted ketimines (**1e**, 4-F, **1f**, 4-Cl and **1g**, 4-Br) underwent coupling with **2a** in lower yield (26–58%). Although the reason for this is not obvious, it could be attributed to the weaker reducing power of azaallyl anions or destabilization of the 2-azaallyl radical by the halide. Reaction with biphenyl substrate (**1h**) proceeded in 67% yield. Unfortunately, ketimines derived from heterocyclic benzylic amines (2-,3-,4-pyridyl, 2-furanyl and 2-thiophene) did not afford coupling products under our standard conditions.

**Table 4:** Scope of ketimines^a^

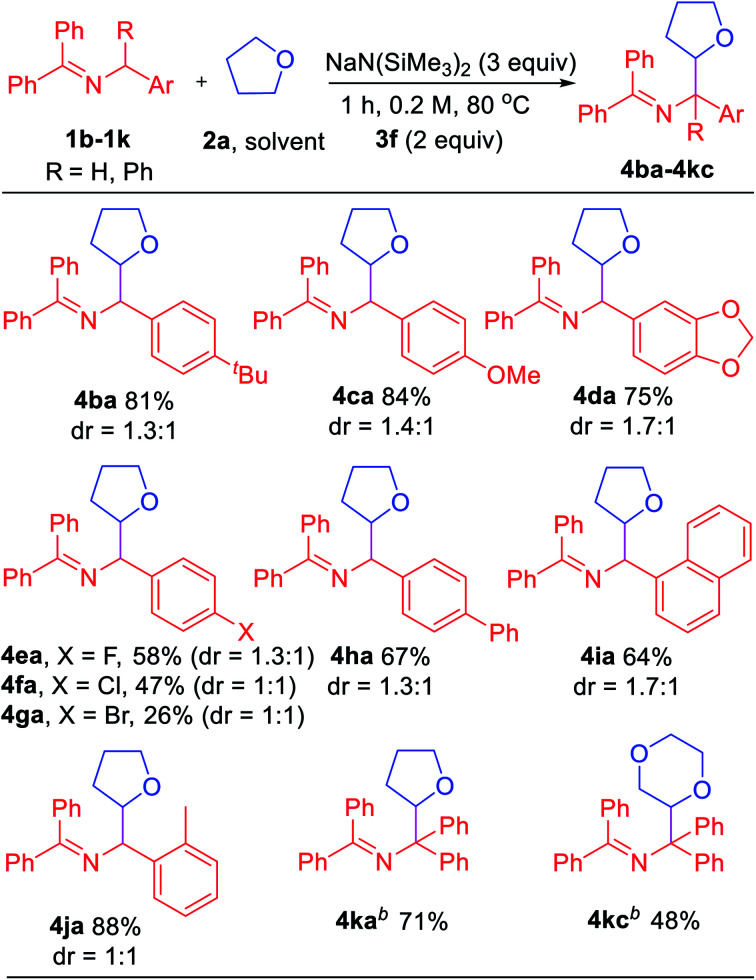

a Reactions conducted on 0.6 mmol scale using 1 equiv. of **1b–1k**. Isolated yields and diastereomeric ratios after chromatographic purification.

b 24 h.

The impact of more sterically hindered *N*-benzylimines was next probed. Interestingly, 1-naphthyl (**1i**) and 2-Me (**1j**) ketimines coupled with tetrahydrofuran in high yields (64% and 88%, respectively). Interestingly, tetraphenyl ketimine **1k**, which has proven unreactive in our previous radical coupling studies, successfully coupled with **2a** in 71% yield. This result demonstrates that the tetraphenyl 2-azaallyl anion is also strongly reducing. We also tested the coupling of tetraphenyl imine **1k** with 1,4-dioxane (**2c**) and isolated 48% yield of the coupled product. The final two entries in Table 4 would be challenging to efficiently prepare by other methods.

Toluene derivatives are abundant and inexpensive components of petroleum distillates, and we have been interested in their functionalization *via* deprotonation under relatively mild conditions.^21^ Here, however, we hypothesized that the aryl radical intermediate would selectively abstract a benzylic hydrogen to generate a benzylic radical. Subsequent radical–radical coupling between the benzyl radical and 2-azaallyl radical would give rise to benzylated imines. As shown in Table 5, under slightly modified conditions (110 °C), net dehydrocoupling of ketimine **1a** and toluene **5a** gave 60% yield of the tetrasubstituted imine. *Ortho*-xylene **5b** reacted with **1a** to form the desired product **6ab** in 71% yield. Similarly, *meta*-xylene **5c** and *para*-xylene **5d** afforded products **6ac** and **6ad** in 70 and 75% yields, respectively. Mesitylene **5e** formed the coupling product **6ae** in 73% yield. Sterically hindered 1-methylnaphthalene **5f** underwent coupling with **1a** to give **6af** in 45% yield. To demonstrate the versatility, ketimine derivatives with 4-Me (**1l**), 4-OMe (**1c**) and 2-Me (**1j**) groups were tested and gave yields of 70–74%.

**Table 5:** Scope of toluene derivatives^a^

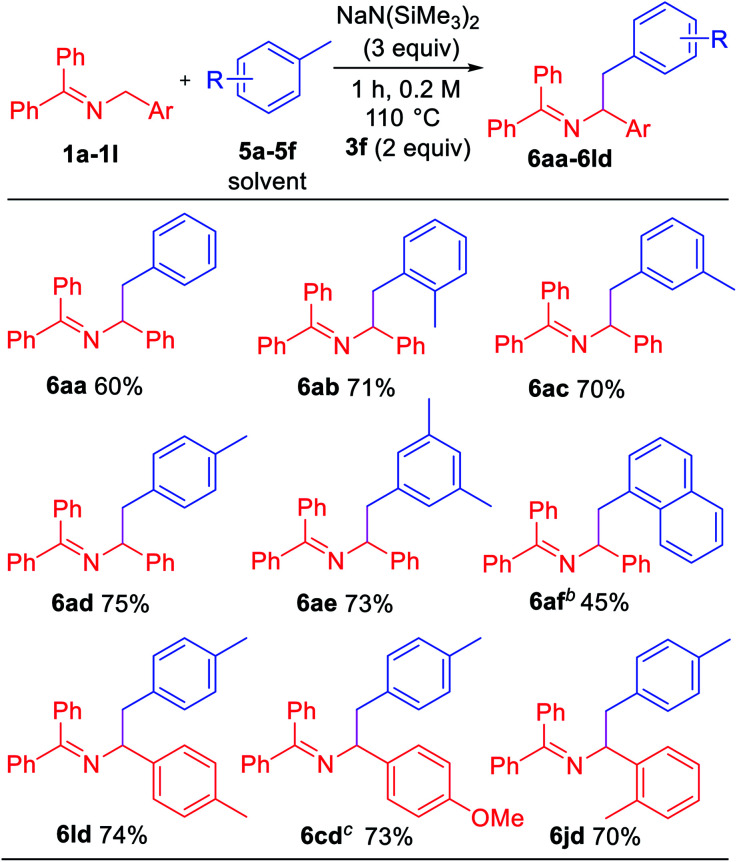

a Reactions conducted on 0.3 mmol scale using 1 equiv. of **1a–1l**. Isolated yield after chromatographic purification.

b 12 h, 150 °C.

c 12 h.

**Scheme 3:**
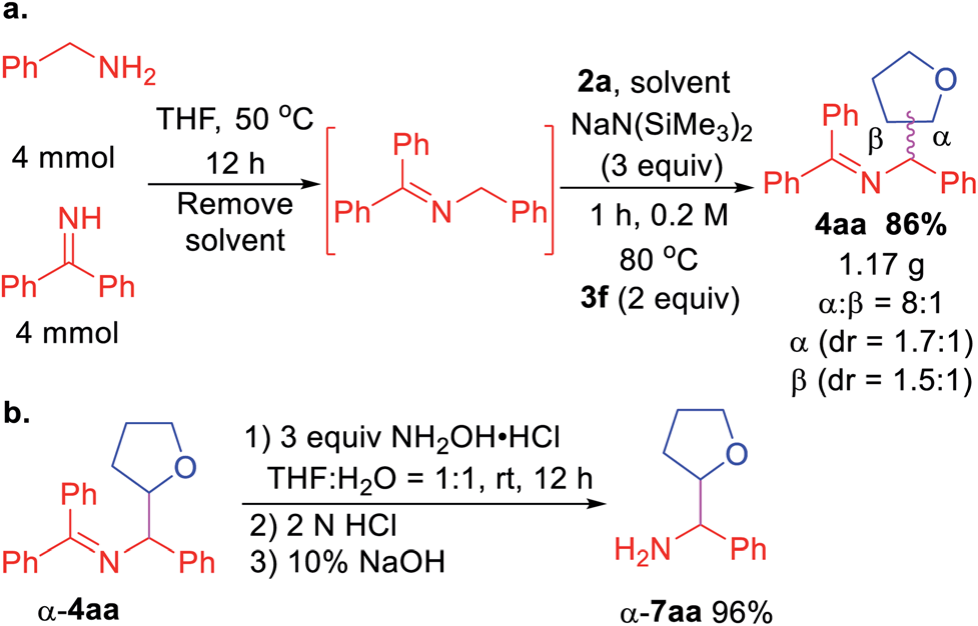
(a) Gram scale telescoped imine formation and coupling. (b) Product hydrolysis.

Scalability test and product hydrolysis were next conducted. We first formed the ketimine **1a** in gram scale (Scheme 3a). Next, treatment of unpurified **1a** with tetrahydrofuran **2a** and 3 equiv. NaN(SiMe_3_)_2_ following the standard procedure afforded 1.17 g of **4aa** (86% over 2 steps). It's worth noting that the β-coupling product was isolated together with α-coupling product at gram scale (8 : 1 regioselectivity). Using a single diastereomer of α-**4aa** as example, hydrolysis of the product afforded α-**7aa** in 96% yield (Scheme 3b).

**Scheme 4:**
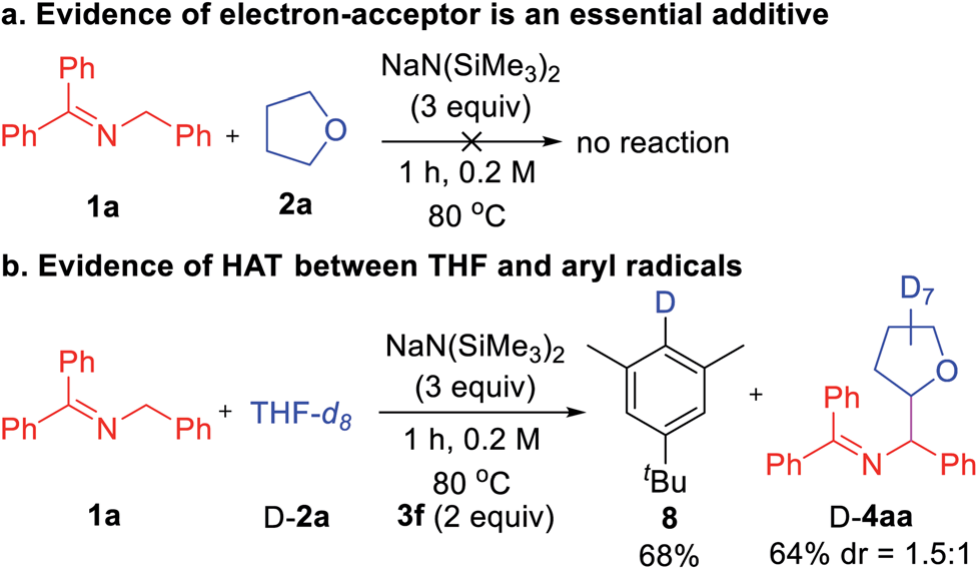
Mechanistic experiments.

Finally, we turned our attention to mechanistic studies to confirm the role of aryl iodide as a hydrogen abstractor. A control experiment was conducted in the absence of aryl iodide **3f** that led to no coupled product, confirming the essential role of aryl iodide (Scheme 4a). Next, subjecting THF-*d*_8_ (D-**2a**) and ketimine **1a** to the optimized conditions afforded deuterated arene **8** in 68% yield (Scheme 4b). Thus, we favor the mechanism outlined in Scheme 2c with key steps being SET from the 2-azaallyl anion to the aryl iodide leading to generation of the sterically protected aryl radical and the persistent 2-azaallyl radical.^22^ The aryl radical then reacts with the ether, amine, or toluene derivatives *via* HAT to generate the alkyl radical, which couples with the 2-azaallyl radical to furnish the products.

## Conclusions

In summary, a new cross-dehydrogenative coupling reaction between C(sp^3^)–H bonds of saturated heterocycles and the benzylic C–H of benzophenone imines has been described. Unlike many past successful advances, which are based on photoredox catalysts and their specialized reactors or on use of peroxides as radical initiators, this approach employs a novel reactivity from a readily accessible organic super electron donor, 2-azaallyl anions. The key advance in this system is the use of a sterically protected sacrificial hydrogen atom abstractor that enables redirection of the reactivity from coupling with the 2-azaallyl radical (Scheme 2b)^14*f*^ to H-abstraction from a donor (ether, amine, or toluene derivative, Scheme 2c). This reaction is highly efficient without traditional strong oxidants and tolerates a range of functional groups. We anticipate that the conceptual advances outlined herein can serve as the bases for introduction of new, transition metal-free strategies to cross-dehydrogenative coupling processes.

## Conflicts of interest

There are no conflicts to declare.

## Supplementary Material

SC-011-D0SC00031K-s001

SC-011-D0SC00031K-s002
