# Supplementary material for: Transition-metal-free C(sp3)–H/C(sp3)–H dehydrogenative coupling of saturated heterocycles with N-benzyl imines
Source: Chem Sci. 2020 Mar 31;11(29):7619–25. doi: 10.1039/d0sc00031k (PMC8152681; doi:10.1039/d0sc00031k)

Electronic Supplementary Material (ESI) for Chemical Science.
This journal is © The Royal Society of Chemistry 2020

Electronic Supplementary Information (ESI)

Transition-Metal-Free C(sp^3)–H/C(sp^3)–H Dehydrogenative Coupling of Saturated Heterocycles with *N*-Benzyl Imines

Zhengfen Liu,†[a] Minyan Li,*†[b] Guogang Deng,[a] Wanshi Wei,[a] Ping Feng,[a] Quanxing Zi,[a] Tiantian Li,[a,c] Hongbin Zhang,*[a] Xiaodong Yang,*[a] & Patrick J. Walsh*[b]

[a]Key Laboratory of Medicinal Chemistry for Natural Resources, Ministry of Education and Yunnan Province, School of Chemical Science and Technology, and State Key Laboratory for Conservation and Utilization of Bio-Resources in Yunnan, Yunnan University, Kunming, 650091, P. R. China. *e-mail: xdyang@ynu.edu.cn; zhanghb@ynu.edu.cn

[b]Roy and Diana Vagelos Laboratories, Penn/Merck Laboratory for High-Throughput Experimentation, Department of Chemistry, University of Pennsylvania, 231 South 34th Street, Philadelphia, Pennsylvania, 19104, USA. *e-mail: pwalsh@sas.upenn.edu; liminyan@sas.upenn.edu

[c]Department of Soil and Water Science, University of Florida. 2181 McCarty Hall A,, Gainesville, FL 32611-0290

†These authors contributed equally to this work.

TABLE OF CONTENT

1. General methods S2
2. Procedure and characterization for C(sp^3) − H/C(sp^3) − H dehydrogenative coupling of saturated heterocycles with *N*-benzyl imines S2
3. Gram scale sequential one-pot ketimine synthesis/alkylation of C(sp^3) − H bonds procedure S25
4. Imine product hydrolysis S24
5. Mechanistic study S25
6. X-ray crystal structures of compound **4aa** S26
7. X-ray crystal structures of compound **4af** S27
8. Supplementary references S27
9. NMR Spectra S27

Supplementary Methods:

General methods

All air- and moisture-sensitive solutions and chemicals were handled under a nitrogen atmosphere of a glovebox and solutions were transferred via "Titan" brand pipettor. Anhydrous solvents were purchased from Sigma-Aldrich and used without further purification. Unless otherwise stated, all reagents were commercially available and used as received without further purification. Chemicals were obtained from Sigma-Aldrich, Acros, TCI and Alfa-Aesar. TLC was performed with Merck TLC Silica gel60 F_{254} plates with detection under UV light at 254 nm. Silica gel (200-300 mesh, Qingdao) was used for flash chromatography. Deactivated silica gel was prepared by addition of 15 mL Et_3N to 1 L of silica gel. The products were purified with XDB-C_{18} (9.4 × 250 mm, 5 μm) column on an Agilent HPLC 1260 system. Proton nuclear magnetic resonance (^{1}H-NMR) spectra were recorded on a Bruker Avance 300 & Bruker DRX 400 & Bruker DRX 600 spectrometer at 300, 400 or 600 MHz. Carbon-13 nuclear magnetic resonance (^{13}C-NMR) was recorded on Bruker Avance 300, Bruker DRX 400 or Bruker DRX 600 spectrometer at 75, 100 or 150 MHz. Chemical shifts were reported in units of parts per million (ppm) downfield from tetramethylsilane (TMS), and all coupling constants were reported in hertz. The infrared (IR) spectra were measured on a Nicolet iS10 FTIR spectrometer with 4 cm^{-1} resolution and 32 scans between wavenumber of 4000 cm^{-1} and 400 cm^{-1}. High Resolution Mass spectra were taken on AB QSTAR Pulsar mass spectrometer and Waters Autospec Premier 776 magnetic mass spectrometer. Melting points were obtained on a XT-4 melting-point apparatus and were uncorrected.

Procedure and characterization for C(sp^3)–H/C(sp^3)–H dehydrogenative coupling of saturated heterocycles with *N*-benzyl imines

General Procedure A:

The synthesis of **4aa** is representative. An oven-dried 8 mL reaction vial equipped with a stir bar was charged with ketimine **1a** (162.8 mg, 0.6 mmol) and 5-(*tert*-butyl)-2-iodo-1,3-dimethylbenzene **3f** (345.8 mg, 1.2 mmol) under a nitrogen atmosphere in a glove box. A solution of $NaN(SiMe_3)_2$ (330.1 mg, 1.8 mmol) in 3 mL dry tetrahydrofuran was added by a "Titan" brand 1000 μL pipettor to the reaction vial at room temperature. The reaction mixture turned to a dark purple color upon addition of the base. The vial was sealed with a cap, removed from the glove box, and stirred for 1 h at 80 ℃. The reaction mixture was cooled to room temperature, opened to air, quenched with ten drops of H_2O, diluted with 6 mL of ethyl acetate, and filtered over a 2 cm pad of $MgSO_4$ and deactivated silica. The pad was rinsed with ethyl acetate (3 X 5 mL), and the combined organic solutions were concentrated *in vacuo*. The crude material was purified on an Agilent HPLC 1260 system using

acetonitrile:H_2O (70:30 vol./vol.) as mobile phase and flow rate of 3.0 mL/min with monitoring at 254 nm to give product.

General Procedure B:

The synthesis of **6aa** is representative. An oven-dried 8 mL reaction vial equipped with a stir bar was charged with ketimine **1a** (81.4 mg, 0.3 mmol) and 5-(*tert*-butyl)-2-iodo-1,3-dimethylbenzene **3f** (172.9 mg, 0.6 mmol) under a nitrogen atmosphere in a glove box. A solution of $NaN(SiMe_3)_2$ (165.1 mg, 0.9 mmol) in 1.5 mL dry toluene was added by a "Titan" brand 1000 μL pipettor to the reaction vial at room temperature. The reaction mixture turned to a dark purple color. Then the vial was sealed with a cap, removed from the glove box, and stirred for 1 h at 110 ℃. The reaction mixture was cooled to room temperature, opened to air, quenched with six drops of H_2O, diluted with 5 mL of ethyl acetate, and filtered over a 2 cm pad of $MgSO_4$ and deactivated silica. The pad was rinsed with ethyl acetate (3 X 5 mL), and the combined solutions were concentrated *in vacuo*. The crude material was purified by flash chromatography on deactivated silica gel (eluted with hexanes to diethyl ether:hexanes = 1:100) to give the product.

Product Characterization:

1,1-diphenyl-*N*-(phenyl(tetrahydrofuran-2-yl)methyl)methanimine (4aa):

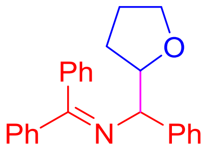

The reaction was performed following the General Procedure A with *N*-benzyl-1,1-diphenylmethanimine **1a** (162.8 mg, 0.6 mmol) and 3 mL dry tetrahydrofuran **2a** (0.2 mol/L). The crude material was purified on an Agilent HPLC 1260 system using acetonitrile:H_2O (70:30 vol./vol.) as mobile phase and flow rate of 3.0 mL/min at 254 nm to give the product **4aa** in 80% overall yield (**4aa**(major), 103.2 mg, 50% yield, **4aa**(minor), 60.7 mg, 30% yield, dr = 1.7:1).

4aa(major): white solid, m.p. = 115 – 116 ℃; R_f = 0.53 (hexanes:ethyl acetate = 8:1); ^{1}H NMR (400 MHz, Chloroform-*d*) δ 7.70 – 7.68 (m, 2H), 7.38 – 7.25 (m, 10H), 7.19 (ddd, *J* = 7.2, 5.6, 1.6 Hz, 1H), 7.05-7.02 (m, 2H), 4.48 (dd, *J* = 6.0, 2.0 Hz, 1H), 4.33 (qd, *J* = 6.4, 2.0 Hz, 1H), 3.81 – 3.74 (m, 1H), 3.72 – 3.67 (m, 1H), 2.03 – 1.94 (m, 1H), 1.92 – 1.73 (m, 3H) ppm; $^{13}C\{^1H\}$ NMR (100 MHz, Chloroform-*d*) δ 167.8, 142.8, 140.1, 137.0, 130.0, 128.7, 128.3, 128.3, 128.1, 127.7, 126.9, 84.3, 69.8, 68.7, 27.8, 26.0 ppm; IR (thin film): 3059, 2869,

1625, 1577, 1446, 1277, 1073, 1028, 940, 780, 699 cm^{-1}; HRMS (ESI^+) $[M+H]^+$ calc'd for $C_{24}H_{24}NO^+$: 342.1852, found: 342.1852.

4aa(minor): colorless oil; R_f = 0.44 (hexanes:ethyl acetate = 8:1); ^{1}H NMR (400 MHz, Chloroform-*d*) δ 7.68 (dd, *J* = 6.4, 1.6 Hz, 2H), 7.42 – 7.39 (m, 3H), 7.34 – 7.25 (m, 7H), 7.23 – 7.19 (m, 1H), 7.11 (dd, *J* = 6.4, 2.8 Hz, 2H), 4.41 – 4.33 (m, 2H), 3.79 – 3.75 (m, 1H), 3.72 – 3.66 (m, 1H), 1.76 – 1.59 (m, 3H), 1.54 – 1.44 (m, 1H) ppm; ^{13}C{^{1}H} NMR (100 MHz, Chloroform-*d*) δ 167.4, 141.9, 140.1, 137.3, 129.9, 128.8, 128.32, 128.29, 128.25, 128.22, 128.19, 128.0, 127.1, 83.4, 71.3, 68.4, 28.5, 25.9 ppm; IR (thin film): 3059, 2869, 1661, 1626, 1447, 1278, 1067, 942, 847, 777, 763, 700 cm^{-1}; HRMS (ESI^+) $[M+H]^+$ calc'd for $C_{24}H_{24}NO^+$: 342.1852, found: 342.1851.

_N_-((3,3-dimethyloxetan-2-yl)(phenyl)methyl)-1,1-diphenylmethanimine (4ab)

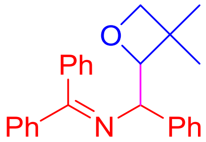

The reaction was performed following the General Procedure A with *N*-benzyl-1,1-diphenylmethanimine **1a** (162.8 mg, 0.6 mmol) and dry 3,3-dimethyloxetane **2b** (258 mg, 3.0 mmol). The crude material was purified on an Agilent HPLC 1260 system using acetonitrile:H_2O (70:30 vol./vol.) as mobile phase and flow rate of 3.0 mL/min at 254 nm to give the product **4ab** in 56% overall yield (**4ab**(major), 88.0 mg, 42% yield, **4ab**(minor), 31.4 mg, 15% yield, dr = 2.8:1).

4ab(major): colorless oil, R_f = 0.42 (hexanes:ethyl acetate = 8:1); ^{1}H NMR (600 MHz, Chloroform-*d*) δ 7.55 – 7.54 (m, 2H), 7.41 – 7.37 (m, 3H), 7.23 – 7.13 (m, 10H), 4.98 (d, *J* = 9.0 Hz, 1H), 4.73 (dd, *J* = 9.6, 1.2 Hz, 1H), 4.19 (d, *J* = 5.4 Hz, 1H), 3.91 (d, *J* = 5.4 Hz, 1H), 0.87 (s, 3H), 0.85 (s, 3H) ppm; ^{13}C{^{1}H} NMR (150 MHz, Chloroform-*d*) δ 168.3, 140.1, 139.9, 137.4, 129.9, 128.8, 128.7, 128.5, 128.30, 128.26, 127.9, 127.5, 92.3, 80.7, 68.4, 38.6, 26.7, 22.0 ppm; IR (thin film): 3060, 2958, 1623, 1577, 1491, 1464, 1446, 1315, 1279, 1028, 977, 776, 700 cm^{-1}; HRMS (ESI^+) $[M+H]^+$ calc'd for $C_{25}H_{26}NO^+$: 356.2009, found: 356.2007.

4ab(minor): colorless oil, R_f = 0.50 (hexanes:ethyl acetate = 8:1); ^{1}H NMR (600 MHz, Chloroform-*d*) δ 7.58 – 7.56 (m, 2H), 7.33 – 7.31 (m, 1H), 7.27 (td, *J* = 7.8, 3.0 Hz, 4H), 7.20 – 7.15 (m, 5H), 7.14 – 7.12(m, 1H), 6.85 (d, *J* = 7.2 Hz, 2H), 4.89 (d, *J* = 9.6 Hz, 1H), 4.45 (d, *J* = 9.6 Hz, 1H), 4.16 (d, *J* = 5.4 Hz, 1H), 3.91 (d, *J* = 5.4 Hz, 1H), 1.28 (s, 3H), 1.03 (s, 3H) ppm; ^{13}C{^{1}H} NMR (150 MHz, Chloroform-*d*) δ 169.0, 142.5, 140.2, 136.9, 130.3, 130.2, 128.8, 128.4, 128.2, 128.2, 128.1, 127.8, 127.1, 93.8, 80.7, 68.4, 38.7, 27.4, 21.7 ppm; IR (thin

film): 3060, 2869, 1654, 1577, 1491, 1447, 1315, 1278, 1075, 982, 775, 697 cm^{-1}; HRMS (ESI$^+$) $[M+H]^+$ calc'd for $C_{25}H_{26}NO^+$: 356.2009, found: 356.2009.

***N*-((1,4-dioxan-2-yl)(phenyl)methyl)-1,1-diphenylmethanimine (4ac)**

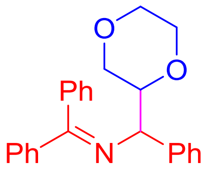

The reaction was performed following the General Procedure A with *N*-benzyl-1,1-diphenylmethanimine **1a** (162.8 mg, 0.6 mmol) and 3 mL dry 1,4-dioxane **2c** (0.2 mol/L). The crude material was purified on an Agilent HPLC 1260 system using acetonitrile:H_2O (70:30 vol./vol.) as mobile phase and flow rate of 3.0 mL/min at 254 nm to give the product **4ac** in 54% overall yield (**4ac**(major), 67.5 mg, 31% yield, **4ac**(minor), 48.3 mg, 23% yield dr = 1.4:1).

4ac(major): white solid, m.p. = 147 – 148 ℃, R_f = 0.34 (hexanes:ethyl acetate = 5:1); ^{1}H NMR (400 MHz, Chloroform-*d*) δ 7.73 – 7.71 (m, 2H), 7.47 – 7.45 (m, 3H), 7.41 – 7.28 (m, 8H), 7.15 – 7.13 (m, 2H), 4.47 (d, *J* = 7.2 Hz, 1H), 4.12 (ddd, *J* = 10.0, 7.2, 2.4 Hz, 1H), 3.84 – 3.74 (m, 2H), 3.69 – 3.66 (m, 1H), 3.59 – 3.50 (m, 2H), 3.26 (t, *J* = 10.8 Hz, 1H) ppm; ^{13}C{^{1}H} NMR (100 MHz, Chloroform-*d*) δ 168.5, 140.4, 140.1, 136.8, 130.0, 128.8, 128.5, 128.4, 128.3, 128.2, 128.1, 128.0, 127.5, 79.1, 68.8, 68.3, 67.0, 66.5 ppm; IR (thin film): 3059, 2854, 1625, 1491, 1447, 1278, 1122, 1108, 766, 700 cm^{-1}; HRMS (ESI$^+$) $[M+H]^+$ calc'd for $C_{24}H_{24}NO_2^+$: 358.1802, found: 358.1804.

4ac(minor): white solid, m.p. = 186 – 187 ℃, R_f = 0.39 (hexanes:ethyl acetate = 5:1); ^{1}H NMR (400 MHz, Chloroform-*d*) δ 7.60 – 7.58 (m, 2H), 7.33 – 7.28 (m, 4H), 7.26 – 7.19 (m, 6H), 7.17 – 7.14 (m, 1H), 6.90 (dd, *J* = 6.4, 2.8 Hz, 2H), 4.27 (d, *J* = 7.6 Hz, 1H), 4.00 (ddd, *J* = 10.0, 7.6, 2.4 Hz, 1H), 3.85 (dd, *J* = 11.6, 2.4 Hz, 1H), 3.60 – 3.42 (m, 4H), 3.35 (dd, *J* = 11.2, 10.0 Hz, 1H) ppm; ^{13}C{^{1}H} NMR (100 MHz, Chloroform-*d*) δ 168.5, 141.8, 139.7, 136.6, 130.3, 128.8, 128.6, 128.5, 128.4, 128.2, 128.0, 127.9, 127.3, 79.7, 69.3, 68.2, 67.2, 66.5 ppm; IR (thin film): 3059, 2849, 1625, 1577, 1491, 1316, 1276, 1124, 1078, 882, 777, 700 cm^{-1}; HRMS (ESI$^+$) $[M+H]^+$ calc'd for $C_{24}H_{24}NO_2^+$: 358.1802, found: 358.1804.

***N*-(2,3-dimethoxy-1-phenylpropyl)-1,1-diphenylmethanimine (4ad)**

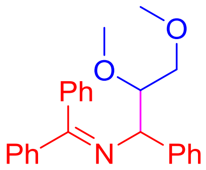

The reaction was performed following the General Procedure A with *N*-benzyl-1,1-diphenylmethanimine **1a** (162.8 mg, 0.6 mmol) and 3 mL dry 1,2-dimethoxyethane **2d** (0.2 mol/L). The crude material was purified on an Agilent HPLC 1260 system using acetonitrile:H_2O (70:30 vol./vol.) as mobile phase and flow rate of 3.0 mL/min at 254 nm to give the product **4ad** in 73% overall yield (157.5 mg, dr = 1.5:1).

colorless oil, R_f = 0.45 (hexanes:ethyl acetate = 5:1); Diastereomeric ratio was determined based on 1,2-dimethoxyethane group H^a (3H, ~ 3.3 – 3.2 ppm) and H^b (3H, ~ 3.1 ppm), see ^1H spectra (Page S34) for determination of diastereomeric ratio; HRMS (ESI$^+$) $[M+H]^+$ calc'd for $C_{24}H_{26}NO_2^+$: 360.1958, found: 360.1955.

N-((3,3-dimethyloxetan-2-yl)(phenyl)methyl)-1,1-diphenylmethanimine (4ae)

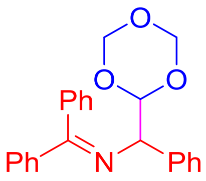

The reaction was performed following the General Procedure A with *N*-benzyl-1,1-diphenylmethanimine **1a** (162.8 mg, 0.6 mmol) and 3 mL dry 1,3,5-trioxane **2e** (0.2 mol/L). The crude material was purified on an Agilent HPLC 1260 system using acetonitrile:H_2O (70:30 vol./vol.) as mobile phase and flow rate of 3.0 mL/min at 254 nm to give the product **4ae** in 58% yield (123.7 mg).

white solid, m.p. = 188 – 189 ℃, R_f = 0.37 (hexanes:ethyl acetate = 5:1); ^1H NMR (600 MHz, Chloroform-*d*) δ 7.59 – 7.57 (m, 2H), 7.35 – 7.33 (m, 3H), 7.30 – 7.28 (m, 1H), 7.25 – 7.19 (m, 6H), 7.18 – 7.16 (m, 1H), 7.02 – 7.00 (m, 2H), 5.31 (d, *J* = 7.2 Hz, 1H), 5.18 (dd, *J* = 6.0, 1.2 Hz, 1H), 5.06 (d, *J* = 6.0 Hz, 1H), 5.04 (dd, *J* = 6.0, 1.2 Hz, 1H), 4.92 (d, *J* = 6.0 Hz, 1H), 4.55 (d, *J* = 7.2 Hz, 1H) ppm; $^{13}C\{^1H\}$ NMR (150 MHz, Chloroform-*d*) δ 169.8, 140.1, 139.6, 136.7, 130.2, 128.9, 128.6, 128.6, 128.5, 128.4, 128.3, 128.1, 127.7, 104.1, 93.6, 93.5, 69.4 ppm; IR (thin film): 3060, 2856, 1626, 1446, 1315, 1262, 1168, 1064, 982, 775, 699 cm^{-1}; HRMS (ESI$^+$) $[M+H]^+$ calc'd for $C_{23}H_{22}NO_3^+$: 360.1594, found: 360.1592.

N-((4-methylmorpholin-3-yl)(phenyl)methyl)-1,1-diphenylmethanimine (4af)

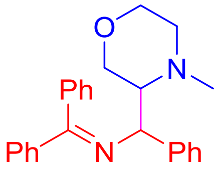

The reaction was performed following the General Procedure A with *N*-benzyl-1,1-diphenylmethanimine **1a** (162.8 mg, 0.6 mmol) and 3 mL dry 4-methylmorpholine **2f** (0.2 mol/L). The crude material was purified on an Agilent HPLC 1260 system using acetonitrile:H_2O (70:30 vol./vol.) as mobile phase and flow rate of 3.0

mL/min at 254 nm to give the product **4af** in 55% overall yield (**4af**(major), 93.2 mg, 42% yield, **4af**(minor), 29.1 mg, 13% yield, dr = 3.2:1).

4af(major): white solid, m.p. = 162 – 163 ℃, R_f = 0.52 (hexanes:ethyl acetate = 1:1); ^{1}H NMR (400 MHz, DMSO-d_6) δ 7.63 – 7.61 (m, 2H), 7.49 – 7.40 (m, 6H), 7.34 (t, *J* = 7.2 Hz, 2H), 7.26 (d, *J* = 7.2 Hz, 1H), 7.23 – 7.20 (m, 2H), 6.94 (d, *J* = 6.8 Hz, 2H), 4.74 (d, *J* = 3.2 Hz, 1H), 3.94 (dd, *J* = 11.2, 9.6 Hz, 1H), 3.63 (d, *J* = 10.8 Hz, 1H), 3.55 (dd, *J* = 11.2, 2.8 Hz, 1H), 3.44 (td, *J* = 11.2, 2.4 Hz, 1H), 2.66 – 2.62 (m, 1H), 2.33 (dt, *J* = 9.6, 3.2 Hz, 1H), 2.12 (td, *J* = 11.6, 3.2 Hz, 1H), 1.96 (s, 3H) ppm; ^{13}C{^{1}H} NMR (100 MHz, DMSO-d_6) δ 167.3, 143.2, 139.8, 135.9, 130.7, 129.3, 128.8, 128.74, 128.68, 127.8, 127.4, 127.3, 69.7, 66.6, 66.4, 63.7, 56.0, 43.2 ppm; IR (thin film): 3059, 2926, 1626, 1576, 1490, 1446, 1315, 1122, 1088, 782, 700 cm^{-1}; HRMS (ESI$^+$) $[M+H]^+$ calc'd for $C_{25}H_{27}N_2O^+$: 371.2118, found: 371.2117.

4af(minor): white solid, m.p. = 162 – 163 ℃, R_f = 0.52 (hexanes:ethyl acetate = 1:1); ^{1}H NMR (400 MHz, Chloroform-*d*) δ 7.59 – 7.56 (m, 2H), 7.34 – 7.31 (m, 3H), 7.28 – 7.21 (m, 3H), 7.20 – 7.15 (m, 5H), 7.00 (dd, *J* = 6.4, 2.8 Hz, 2H), 4.34 (d, *J* = 7.2 Hz, 1H), 3.98 (ddd, *J* = 10.0, 7.2, 2.0 Hz, 1H), 3.81 (ddd, *J* = 11.6, 3.6, 1.6 Hz, 1H), 3.66 (td, *J* = 11.6, 2.4 Hz, 1H), 2.58 (d, *J* = 11.2 Hz, 1H), 2.43 (d, *J* = 11.2 Hz, 1H), 2.14 (s, 3H), 2.04 (dt, *J* = 11.2, 5.6 Hz, 1H), 1.68 (t, *J* = 10.8 Hz, 1H) ppm; ^{13}C{^{1}H} NMR (100 MHz, Chloroform-*d*) δ 168.4, 140.8, 140.2, 137.0, 130.0, 128.9, 128.43, 128.37, 128.3, 128.0, 127.4, 79.3, 69.4, 66.6, 57.1, 54.8, 46.2 ppm; IR (thin film): 3059, 2926, 1626, 1576, 1490, 1446, 1315, 1122, 1088, 782, 700 cm^{-1}; HRMS (ESI$^+$) $[M+H]^+$ calc'd for $C_{25}H_{27}N_2O^+$: 371.2118, found: 371.2117.

***N*-((1-methylpyrrolidin-2-yl)(phenyl)methyl)-1,1-diphenylmethanimine (4ag)**

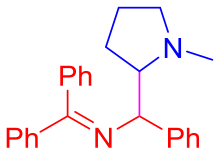

The reaction was performed following the General Procedure A with *N*-benzyl-1,1-diphenylmethanimine **1a** (162.8 mg, 0.6 mmol) and 3 mL dry 1-methylpyrrolidine **2g** (0.2 mol/L). The crude material was purified on an Agilent HPLC 1260 system using acetonitrile:H_2O (70:30 vol./vol.) as mobile phase and flow rate of 3.0 mL/min at 254 nm to give the product **4ag** in 56% overall yield (**4ag**(major), 59.6 mg, 28% yield, **4ag**(minor), 59.6 mg, 28% yield, dr = 1:1).

4ag(major): colorless oil, R_f = 0.43 (hexanes:ethyl acetate = 5:1); ^{1}H NMR (400 MHz, Methanol-d_4) δ 7.56 – 7.53 (m, 2H), 7.31 – 7.20 (m, 6H), 7.17 – 7.07 (m, 5H), 6.88 – 6.86 (m, 2H), 4.35 (d, *J* = 5.2 Hz, 1H), 2.89 – 2.85 (m, 1H), 2.64 – 2.59 (m, 1H), 2.17 – 2.06 (m, 2H), 1.83 (s, 3H), 1.73 – 1.56 (m, 3H) ppm; ^{13}C{^{1}H} NMR

(100 MHz, Methanol-d_4) δ 168.2, 143.4, 139.9, 137.1, 129.7, 128.21, 128.17, 128.0, 127.9, 127.6, 127.5, 127.4, 126.6, 72.8, 67.7, 57.7, 40.4, 26.6, 21.9 ppm; IR (thin film): 3059, 2964, 2775, 1627, 1599, 1491, 1447, 1314, 1146, 1029, 778, 669 cm^{-1}; HRMS (ESI$^+$) $[M+H]^+$ calc'd for $C_{25}H_{27}N_2^+$: 355.2167, found: 355.2166.

4ag(minor): colorless oil, R_f = 0.30 (hexanes:ethyl acetate = 5:1); ^{1}H NMR (400 MHz, Methanol-d_4) δ 7.64 – 7.61 (m, 2H), 7.43 – 7.39 (m, 3H), 7.35 – 7.28 (m, 3H), 7.25 – 7.18 (m, 3H), 7.17 – 7.14 (m, 2H), 7.02 – 6.99 (m, 2H), 4.37 (d, J = 7.6 Hz, 1H), 3.03 – 2.97 (m, 2H), 2.43 (s, 3H), 2.32 – 2.26 (m, 1H), 1.61 – 1.37 (m, 4H) ppm; ^{13}C{^{1}H} NMR (100 MHz, Methanol-d_4) δ 167.6, 142.8, 139.8, 136.9, 129.7, 128.13, 128.09, 127.9, 127.8, 127.7, 126.6, 71.5, 71.4, 58.0, 41.9, 28.6, 21.9 ppm; IR (thin film): 3059, 2961, 2778, 1624, 1577, 1491, 1446, 1314, 1029, 776, 699 cm^{-1}; HRMS (ESI$^+$) $[M+H]^+$ calc'd for $C_{25}H_{27}N_2^+$: 355.2169, found: 355.2166.

1,1-diphenyl-*N*-(phenyl(1-phenylpyrrolidin-2-yl)methyl)methanimine (4ah)

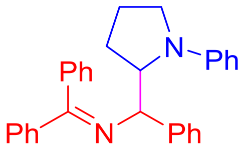

The reaction was performed following the General Procedure A with *N*-benzyl-1,1-diphenylmethanimine **1a** (162.8 mg, 0.6 mmol) and 3 mL dry 1-phenylpyrrolidine **2h** (0.2 mol/L). The crude material was purified on an Agilent HPLC 1260 system using acetonitrile:H_2O (70:30 vol./vol.) as mobile phase and flow rate of 3.0 mL/min at 254 nm to give the product **4ah** in 81% overall yield (**4ah**(major), 40.0 mg, 16% yield, **4ah**(minor), 162.5 mg, 65% yield, dr = 4.1:1).

4ah(major): white solid, m.p. = 133 – 134 ℃, R_f = 0.51 (hexanes:ethyl acetate = 5:1); ^{1}H NMR (400 MHz, Chloroform-*d*) δ 7.84 – 7.81 (m, 2H), 7.57 – 7.54 (m, 3H), 7.50 – 7.40 (m, 5H), 7.35 – 7.29 (m, 3H), 7.23 – 7.14 (m, 4H), 6.69 (t, J = 7.2 Hz, 1H), 6.46 (d, J = 8.0 Hz, 2H), 5.00 (d, J = 5.2 Hz, 1H), 3.99 (t, J = 6.8 Hz, 1H), 3.13 – 3.02 (m, 2H), 2.44 (dd, J = 12.8, 7.2 Hz, 1H), 1.94 – 1.84 (m, 1H), 1.70 – 1.63 (m, 1H), 1.02 – 0.90 (m, 1H) ppm; ^{13}C{^{1}H} NMR (100 MHz, Chloroform-*d*) δ 166.9, 147.4, 142.0, 139.8, 137.0, 130.1, 129.0, 128.8, 128.6, 128.5, 128.3, 128.1, 128.0, 126.9, 115.6, 112.2, 65.9, 64.5, 49.1, 26.8, 23.2 cm^{-1}; IR (thin film): 3058, 2969, 1622, 1596, 1504, 1446, 1364, 1180, 1028, 747, 703 cm^{-1}; HRMS (ESI$^+$) $[M+H]^+$ calc'd for $C_{30}H_{29}N_2^+$: 417.2325, found: 417.2324.

4ah(minor): colorless oil, R_f = 0.57 (hexanes:ethyl acetate = 5:1); ^{1}H NMR (400 MHz, Chloroform-*d*) δ 7.77 – 7.74 (m, 2H), 7.47 – 7.34 (m, 7H), 7.29 – 7.27 (m, 1H), 7.21 – 7.19 (m, 3H), 7.02 (t, J = 7.6 Hz, 2H), 6.67 (t, J = 7.2 Hz, 1H), 6.62 (d, J = 7.6 Hz, 2H), 6.52 (d, J = 8.0 Hz, 2H), 5.03 (d, J = 2.8 Hz, 1H), 4.21 (d, J = 8.4 Hz, 1H), 3.48 (td, J = 8.4, 3.2 Hz, 1H), 3.25 (q, J = 8.0 Hz, 1H), 2.62 (ddd, J = 12.4, 8.8, 4.0 Hz, 1H), 2.45 – 2.34 (m, 1H),

2.02 – 1.94 (m, 1H), 1.88 – 1.78 (m, 1H) ppm; $^{13}C\{^1H\}$ NMR (100 MHz, Chloroform-*d*) δ 168.6, 147.1, 143.3, 139.9, 136.1, 130.0, 129.1, 128.6, 128.4, 128.2, 127.8, 127.6, 127.5, 127.3, 126.7, 115.5, 112.2, 65.4, 65.2, 49.5, 26.6, 24.6 ppm; IR (thin film): 3058, 2968, 1623, 1596, 1504, 1447, 1362, 1344, 1175, 1028, 745, 699 cm^{-1}; HRMS (ESI^+) $[M+H]^+$ calc'd for $C_{30}H_{29}N_2^+$: 417.2325, found: 417.2325.

1,1-diphenyl-*N*-(phenyl(1-phenylpiperidin-2-yl)methyl)methanimine (4ai)

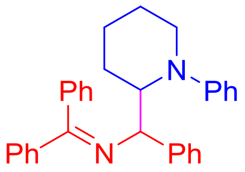

The reaction was performed following the General Procedure A with *N*-benzyl-1,1-diphenylmethanimine **1a** (162.8 mg, 0.6 mmol) and dry 1-phenylpiperidine **2i** (483.7 mg, 3.0 mmol). The crude material was purified on an Agilent HPLC 1260 system using acetonitrile:H_2O (70:30 vol./vol.) as mobile phase and flow rate of 3.0 mL/min at 254 nm to give the product **4ai** in 87% overall yield (224.8 mg, dr = 1.9:1).

colorless oil, R_f = 0.46 (hexanes:ethyl acetate = 5:1); Diastereomeric ratio was determined based on ketimine group H^a (1H, ~ 4.9 – 4.8 ppm) and 1-phenylpiperidine group H^b (1H, ~ 4.6 – 4.3 ppm), see ^1H spectra (Page S42) for determination of diastereomeric ratio; HRMS (ESI^+) $[M+H]^+$ calc'd for $C_{31}H_{31}N_2^+$: 431.2482, found: 431.2480.

1,1-diphenyl-*N*-(1-phenyl-2-(1*H*-pyrrol-1-yl)ethyl)methanimine (4aj)

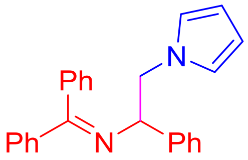

The reaction was performed following the General Procedure A with *N*-benzyl-1,1-diphenylmethanimine **1a** (162.8 mg, 0.6 mmol) and 3 mL dry 1-methyl-1*H*-pyrrole **2j** (0.2 mol/L). The crude material was purified on an Agilent HPLC 1260 system using acetonitrile:H_2O (70:30 vol./vol.) as mobile phase and flow rate of 3.0 mL/min at 254 nm to give the product **4aj** in 77% yield (161.9 mg).

white solid, m.p. = 108 – 109 ℃, R_f = 0.35 (hexanes:ethyl acetate = 5:1); ^1H NMR (600 MHz, Chloroform-*d*) 7.74 – 7.70 (m, 2H), 7.44 – 7.31 (m, 11H), 7.64 – 7.59 (m, 4H), 6.12 (dd, *J* = 14.4, 1.8 Hz, 2H), 4.67 – 4.62 (m, 1H), 4.44 (q, *J* = 13.2 Hz, 1H), 4.21 – 4.15 (m, 1H) ppm; $^{13}C\{^1H\}$ NMR (150 MHz, Chloroform-*d*) δ 168.8, 141.8, 139.7, 136.5, 130.2, 128.8, 128.7, 128.3, 128.1, 127.7, 127.6, 127.4, 121.3, 108.1, 68.4, 57.5 ppm; IR

(thin film): 3058, 2922, 1625, 1577, 1494, 1446, 1314, 1285, 1087, 723, 696 cm^{-1}; HRMS (ESI$^+$) [M+H]$^+$ calc'd for $C_{25}H_{23}N_2^+$: 351.1856, found:351.1857.

***N*-(2-((diphenylmethylene)amino)-2-phenylethyl)-*N*-methylaniline (4ak)**

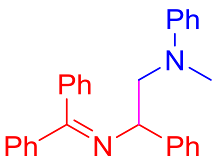

The reaction was performed following the General Procedure A with *N*-benzyl-1,1-diphenylmethanimine **1a** (162.8 mg, 0.6 mmol) and dry *N*,*N*-dimethylaniline **2k** (363.5 mg, 3.0 mmol). The crude material was purified on an Agilent HPLC 1260 system using acetonitrile:H_2O (70:30 vol./vol.) as mobile phase and flow rate of 3.0 mL/min at 254 nm to give the product **4ak** in 58% yield (135.9 mg).

yellow oil, R_f = 0.41 (hexanes:ethyl acetate = 5:1); ^{1}H NMR (400 MHz, Chloroform-*d*) δ 7.80 – 7.77 (m, 2H), 7.51 – 7.49 (m, 2H), 7.46 – 7.39 (m, 6H), 7.37 – 7.32 (m, 3H), 7.24 – 7.19 (m, 2H), 6.98 – 6.95 (m, 2H), 6.75 – 6.71 (m, 1H), 6.63 – 6.60 (m, 2H), 4.92 (dd, *J* = 8.4, 4.8 Hz, 1H), 3.92 (dd, *J* = 14.8, 8.4 Hz, 1H), 3.73 (dd, *J* = 14.8, 4.8 Hz, 1H), 2.85 (s, 3H) ppm; ^{13}C{^{1}H} NMR (100 MHz, Chloroform-*d*) δ 167.9, 148.9, 142.8, 139.8, 136.7, 130.0, 129.1, 128.7, 128.5, 128.23, 128.18, 128.1, 127.8, 127.4, 127.2, 115.8, 111.8, 64.7, 61.2, 39.6 ppm; IR (thin film): 3059, 2900, 1598, 1505, 1491, 1342, 1242, 1192, 1028, 746, 698 cm^{-1}; HRMS (ESI$^+$) [M+H]$^+$ calc'd for $C_{28}H_{27}N_2^+$: 391.2169, found: 391.2170.

***N*-((4-(*tert*-butyl)phenyl)(tetrahydrofuran-2-yl)methyl)-1,1-diphenylmethanimine (4ba)**

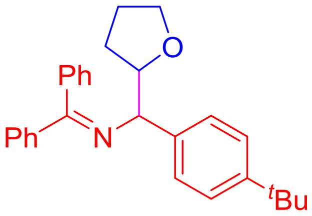

The reaction was performed following the General Procedure A with *N*-(4-(*tert*-butyl)benzyl)-1,1-diphenylmethanimine **1b** (196.5 mg, 0.6 mmol) and 3 mL dry tetrahydrofuran **2a** (0.2 mol/L). The crude material was purified on an Agilent HPLC 1260 system using acetonitrile:H_2O (70:30 vol./vol.) as mobile phase and flow rate of 3.0 mL/min at 254 nm to give the product **4ba** in 81% overall yield (**4ba**(major), 109.2 mg, 46% yield, **4ba**(minor), 84.0 mg, 35% yield, dr = 1.3:1).

4ba(major): colorless oil, R_f = 0.52 (hexanes:ethyl acetate = 5:1); ^{1}H NMR (600 MHz, Chloroform-*d*) δ 7.75 – 7.23 (m, 2H), 7.46 – 7.44 (m, 3H), 7.39 – 7.34 (m, 1H), 7.39 – 7.33 (m, 4H), 7.32 – 7.31 (m, 2H), 7.13 – 7.10 (m,

2H), 4.51 (dd, J = 6.0, 1.8 Hz, 1H), 4.41 – 4.37 (m, 1H), 3.85 – 3.81 (m, 1H), 3.71 – 3.74 (m, 1H), 2.04 – 2.00 (m, 1H), 1.98 – 1.92 (m, 1H), 1.91 – 1.82 (m, 2H), 1.35 (d, J = 1.8 Hz, 9H) ppm; $^{13}C\{^1H\}$ NMR (150 MHz, Chloroform-*d*) δ 167.4, 149.5, 140.3, 139.7, 137.1, 129.9, 128.7, 128.3, 128.2, 128.1, 127.3, 125.2, 84.2, 69.6, 68.7, 34.5, 31.6, 28.0, 26.0 ppm; IR (thin film): 3080, 2963, 1625, 1578, 1460, 1314, 1289, 1068, 696 cm^{-1}; HRMS (ESI^+) $[M+H]^+$ calc'd for $C_{28}H_{32}NO^+$: 398.2478, found: 398.2478.

4ba(minor): colorless oil, R_f = 0.39 (hexanes:ethyl acetate = 5:1); ^1H NMR (400 MHz, Chloroform-*d*) δ 7.61 – 7.59 (m, 2H), 7.33 (dd, J = 4.8, 2.0 Hz, 3H), 7.24 – 7.15 (m, 7H), 7.06 (dd, J = 6.8, 2.8 Hz, 2H), 4.31 (q, J = 6.8 Hz, 1H), 4.23 (d, J = 7.6 Hz, 1H), 3.69 (td, J = 7.6, 5.2 Hz, 1H), 3.61 (dt, J = 8.4, 6.4 Hz, 1H), 1.70 – 1.53 (m, 3H), 1.44 – 1.35 (m, 1H), 1.22 (s, 9H) ppm; $^{13}C\{^1H\}$ NMR (100 MHz, Chloroform-*d*) δ 167.1, 149.9, 140.2, 138.8, 137.4, 129.8, 128.8, 128.4, 128.23, 128.16, 128.0, 127.8, 125.2, 83.4, 71.1, 68.3, 34.6, 31.5, 28.6, 26.0 ppm; IR (thin film): 3056, 2963, 1625, 1445, 1314, 1271, 1068, 696 cm^{-1}; HRMS (ESI^+) $[M+H]^+$ calc'd for $C_{28}H_{32}NO^+$: 398.2478, found: 398.2475.

_N_-((4-methoxyphenyl)(tetrahydrofuran-2-yl)methyl)-1,1-diphenylmethanimine (4ca)

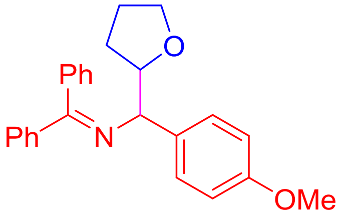

The reaction was performed following the General Procedure A with *N*-(4-methoxybenzyl)-1,1-diphenylmethanimine **1c** (180.8 mg, 0.6 mmol) and 3 mL dry tetrahydrofuran **2a** (0.2 mol/L). The crude material was purified on an Agilent HPLC 1260 system using acetonitrile:H_2O (70:30 vol./vol.) as mobile phase and flow rate of 3.0 mL/min at 254 nm to give the product **4ca** in 84% overall yield (**4ca**(major), 109.2 mg, 49% yield, **4ca**(minor), 78.0 mg, 35% yield, dr = 1.4:1).

4ca(major): colorless oil, R_f = 0.35 (hexanes:ethyl acetate = 5:1); ^1H NMR (400 MHz, Chloroform-*d*) δ 7.61 – 7.59 (m, 2H), 7.32 – 7.23 (m, 6H), 7.17 – 7.15 (m, 2H), 6.96 (dd, J = 6.4, 2.8 Hz, 2H), 6.76 – 6.74 (m, 2H), 4.32 (d, J = 6.0 Hz, 1H), 4.21 (q, J = 6.4 Hz, 1H), 3.72 – 3.66 (m, 4H), 3.64 – 3.58 (m, 1H), 1.87 – 1.80 (m, 2H), 1.78 – 1.68 (m, 2H) ppm; $^{13}C\{^1H\}$ NMR (100 MHz, Chloroform-*d*) δ 167.6, 158.5, 140.2, 137.1, 135.0, 130.0, 128.71, 128.66, 128.4, 128.1, 113.8, 84.3, 69.3, 68.7, 55.3, 28.0, 26.0 ppm; IR (thin film): 3058, 2870, 1611, 1509, 1445, 1314, 1246, 1174, 1067, 1036, 697 cm^{-1}; HRMS (ESI^+) $[M+H]^+$ calc'd for $C_{25}H_{26}NO_2^+$: 372.1958, found: 372.1955.

4ca(minor): colorless oil, R_f = 0.30 (hexanes:ethyl acetate =5:1); ^{1}H NMR (400 MHz, Chloroform-*d*) δ 7.74 – 7.71 (m, 2H), 7.47 – 7.44 (m, 3H), 7.37 – 7.31 (m, 3H), 7.30 – 7.26 (m, 2H), 7.17 (dd, *J* = 6.8, 2.8 Hz, 2H), 6.90 – 6.82 (m, 2H), 4.42 – 4.37 (m, 1H), 4.34 (d, *J* = 7.6 Hz, 1H), 3.84 – 3.78 (m, 4H), 3.77 – 3.71 (m, 1H), 1.82 – 1.65 (m, 3H), 1.59 – 1.49 (m, 1H) ppm; $^{13}C\{^{1}H\}$ NMR (100 MHz, Chloroform-*d*) δ 167.1, 158.7, 140.2, 137.3, 134.1, 129.8, 129.1, 128.8, 128.3, 128.2, 128.1, 127.9, 113.7, 83.4, 70.5, 68.4, 55.3, 28.4, 25.9 ppm; IR (thin film): 3058, 2871, 1610, 1510, 1446, 1315, 1247, 1174, 1067, 780, 697 cm^{-1}; HRMS (ESI^+) $[M+H]^+$ calc'd for $C_{25}H_{26}NO_2^+$: 372.1958, found: 372.1956.

***N*-(benzo[*d*][1,3]dioxol-5-yl(tetrahydrofuran-2-yl)methyl)-1,1-diphenylmethanimine (4da)**

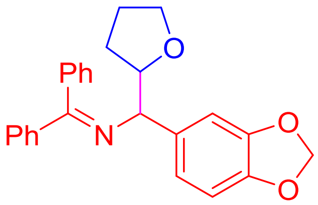

The reaction was performed following the General Procedure A with *N*-(benzo[*d*][1,3]dioxol-5-ylmethyl)-1,1-diphenylmethanimine **1d** (189.2 mg, 0.6 mmol) and 3 mL dry tetrahydrofuran **2a** (0.2 mol/L). The crude material was purified on an Agilent HPLC 1260 system using acetonitrile:H_2O (70:30 vol./vol.) as mobile phase and flow rate of 3.0 mL/min at 254 nm to give the product **4da** in 75% overall yield (**4da**(major), 109.2 mg, 47% yield, **4da**(minor), 64.3 mg, 28% yield, dr = 1.7:1).

4da(major): white solid, m.p. = 122 – 123 ℃; R_f = 0.40 (hexanes:ethyl acetate = 5:1); ^{1}H NMR (400 MHz, Chloroform-*d*) δ 7.77 – 7.74 (m, 2H), 7.49 – 7.37 (m, 6H), 7.15 – 7.10 (m, 2H), 7.01 (d, *J* = 1.2 Hz, 1H), 6.79 (d, *J* = 1.2 Hz, 2H), 5.96 (dd, *J* = 5.6, 1.6 Hz, 2H), 4.43 (d, *J* = 6.4 Hz, 1H), 4.35 (q, *J* = 6.4 Hz, 1H), 3.87 – 3.81 (m, 1H), 3.79 – 3.74 (m, 1H), 2.01 – 1.92 (m, 2H), 1.91 – 1.82 (m, 2H) ppm; $^{13}C\{^{1}H\}$ NMR (100 MHz, Chloroform-*d*) δ 167.6, 147.5, 146.4, 140.0, 136.9, 136.7, 130.0, 128.7, 128.4, 128.3, 128.1, 128.0, 120.7, 108.2, 108.1, 100.8, 84.2, 69.6, 68.7, 28.1, 25.9 ppm; IR (thin film): 3058, 2873, 1625, 1502, 1487, 1442, 1247, 1067, 1040, 698 cm^{-1}; HRMS (ESI^+) $[M+H]^+$ calc'd for $C_{25}H_{24}NO_3^+$: 386.1751, found: 386.1747.

4da(minor): colorless oil, R_f = 0.34 (hexanes:ethyl acetate = 3:1); ^{1}H NMR (400 MHz, Chloroform-*d*) δ 7.61 – 7.58 (m, 2H), 7.36 – 7.33 (m, 3H), 7.28 – 7.20 (m, 3H), 7.05 (dd, *J* = 6.8, 2.8 Hz, 2H), 6.87 (s, 1H), 6.64 – 6.57 (m, 2H), 5.84 (d, *J* = 4.0 Hz, 2H), 4.24 (q, *J* = 6.8 Hz, 1H), 4.17 (d, *J* = 7.2 Hz, 1H), 3.71 – 3.66 (m, 1H), 3.64 – 3.59 (m, 1H), 1.71 – 1.54 (m, 3H), 1.47 – 1.37 (m, 1H) ppm; $^{13}C\{^{1}H\}$ NMR (100 MHz, Chloroform-*d*) δ 167.4, 147.6, 146.6, 140.1, 137.2, 135.8, 130.0, 128.8, 128.3, 128.2, 128.0, 108.7, 108.1, 100.9, 83.4, 70.8, 68.4, 28.4,

26.0 ppm; IR (thin film): 3058, 2876, 1625, 1503, 1486, 1444, 1245, 1067, 1040, 698 m^{-1}; HRMS (ESI$^+$) $[M+H]^+$ calc'd for $C_{25}H_{24}NO_3^+$: 386.1751, found: 386.1752.

***N*-((4-fluorophenyl)(tetrahydrofuran-2-yl)methyl)-1,1-diphenylmethanimine (4ea)**

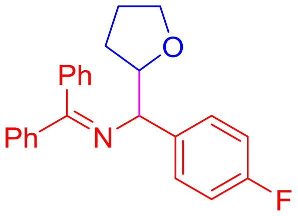

The reaction was performed following the General Procedure A with *N*-(4-fluorobenzyl)-1,1-diphenylmethanimine **1e** (173.6 mg, 0.6 mmol) and 3 mL dry tetrahydrofuran **2a** (0.2 mol/L). The crude material was purified on an Agilent HPLC 1260 system using acetonitrile:H_2O (70:30 vol./vol.) as mobile phase and flow rate of 3.0 mL/min at 254 nm to give the product **4ea** in 58% overall yield (**4ea**(major), 70.7 mg, 33% yield, **4ea**(minor), 54.1 mg, 25% yield, dr = 1.3:1).

4ea(major): yellow oil, R_f = 0.37 (hexanes:ethyl acetate = 5:1); ^{1}H NMR (600 MHz, Chloroform-*d*) δ 7.61 – 7.59 (m, 2H), 7.32 – 7.28 (m, 4H), 7.26 – 7.23 (m, 2H), 7.21 – 7.18 (m, 2H), 6.94 – 6.93 (m, 2H), 6.90 – 6.85 (m, 2H), 4.32 (d, *J* = 6.0 Hz, 1H), 4.20 (q, *J* = 6.6 Hz, 1H), 3.68 (dt, *J* = 7.8, 6.6 Hz, 1H), 3.63 – 3.60 (m, 1H), 1.83 – 1.79 (m, 2H), 1.76 – 1.69 (m, 2H) ppm; ^{13}C{^{1}H} NMR (150 MHz, Chloroform-*d*) δ 168.1, 161.9 (d, $^1J_{C\text{-}F}$ = 243.1 Hz), 140.0, 138.64, 138.62, 137.0, 130.2, 138.6 (d, $^3J_{C\text{-}F}$ = 3.1 Hz), 128.7, 128.5, 128.4, 128.2, 128.0, 115.1 (d, $^2J_{C\text{-}F}$ = 21.0 Hz), 84.3, 69.3, 68.7, 28.1, 25.9 ppm; IR (thin film): 3058, 2870, 1626, 1507, 1446, 1294, 1221, 1156, 1069, 696 cm^{-1}; HRMS (ESI$^+$) $[M+H]^+$ calc'd for $C_{24}H_{23}FNO^+$: 360.1758, found: 360.1756.

4ea(minor): yellow oil, R_f = 0.35 (hexanes:ethyl acetate = 5:1); ^{1}H NMR (600 MHz, Chloroform-*d*) δ 7.60 – 7.58 (m, 2H), 7.34 – 7.32 (m, 3H), 7.27 – 7.25 (m, 1H), 7.23 – 7.19 (m, 4H), 7.02 – 7.01 (m, 2H), 6.88 (t, *J* = 8.4 Hz, 2H), 4.28 – 4.25 (m, 1H), 4.24 – 4.21 (m, 1H), 3.68 (td, *J* = 7.8, 5.4 Hz, 1H), 3.61 (td, *J* = 7.8, 6.0 Hz, 1H), 1.67 – 1.63 (m, 1H), 1.62 – 1.52 (m, 2H), 1.45 – 1.40 (m, 1H) ppm; ^{13}C{^{1}H} NMR (150 MHz, Chloroform-*d*) δ 167.7, 162.1 (d, $^1J_{C\text{-}F}$ = 243.1 Hz), 140.0, 137.6 (d, $^3J_{C\text{-}F}$ = 3.3 Hz), 137.2, 130.0, 129.7, 129.6, 128.8, 128.4, 128.3, 128.2, 128.0, 115.1 (d, $^2J_{C\text{-}F}$ = 20.9 Hz), 83.3, 70.3, 68.4, 28.3, 25.9 ppm; IR (thin film): 3059, 2871, 1625, 1602, 1507, 1446, 1222, 1156, 1069, 780, 696 cm^{-1}; HRMS (ESI$^+$) $[M+H]^+$ calc'd for $C_{24}H_{23}FNO^+$: 360.1758, found: 360.1756.

***N*-((4-chlorophenyl)(tetrahydrofuran-2-yl)methyl)-1,1-diphenylmethanimine （4fa）**

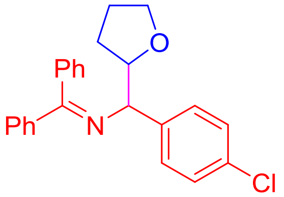

The reaction was performed following the General Procedure A with *N*-(4-chlorobenzyl)-1,1-diphenylmethanimine**1f** (183.5 mg, 0.6 mmol) and 3 mL dry tetrahydrofuran **2a** (0.2 mol/L). The crude material was purified on an Agilent HPLC 1260 system using acetonitrile:H_2O (70:30 vol./vol.) as mobile phase and flow rate of 3.0 mL/min at 254 nm to give the product **4fa** in 47% overall yield (**4fa**(major), 53 mg, 23.5% yield, **4fa**(minor), 53 mg, 23.5% yield, dr = 1:1).

4fa(major): colorless oil, R_f = 0.38 (hexanes:ethyl acetate = 5:1); ^{1}H NMR (400 MHz, Chloroform-*d*) δ 7.69 – 7.66 (m, 2H), 7.41 – 7.31 (m, 6H), 7.24 (d, *J* = 4.8 Hz, 4H), 7.03 – 7.00 (m, 2H), 4.39 (d, *J* = 6.4 Hz, 1H), 4.27 (q, *J* = 6.4 Hz, 1H), 3.76 (dt, *J* = 8.0, 6.4 Hz, 1H), 3.69 (dt, *J* = 8.0, 6.4 Hz, 1H), 1.93 – 1.86 (m, 2H), 1.84 – 1.77 (m, 2H) ppm; ^{13}C{^{1}H} NMR (100 MHz, Chloroform-*d*) δ 168.3, 141.4, 139.9, 136.9, 132.6, 130.2, 129.1, 128.7, 128.51, 128.47, 128.2, 128.0, 84.1, 69.4, 68.8, 28.1, 25.9 ppm; HRMS (ESI$^+$) [M+H]$^+$ calc'd for $C_{24}H_{23}ClNO^+$: 376.1463, found: 376.1464.

4fa(minor): colorless oil, R_f = 0.36 (hexanes:ethyl acetate = 5:1); ^{1}H NMR (400 MHz, Chloroform-*d*) δ 7.68 – 7.66 (m, 2H), 7.42 – 7.39 (m, 3H), 7.37 – 7.27 (m, 3H), 7.26 – 7.23 (m, 4H), 7.10 – 7.07 (m, 2H), 4.34 (d, *J* = 7.2 Hz, 1H), 4.29 (q, *J* = 6.4 Hz, 1H), 3.76 (td, *J* = 7.6, 5.2 Hz, 1H), 3.71 – 3.66 (m, 1H), 1.79 – 1.59 (m, 3H), 1.56 – 1.45 (m, 1H) ppm; ^{13}C{^{1}H} NMR (100 MHz, Chloroform-*d*) δ 167.9, 140.4, 137.1, 132.8, 130.1, 129.5, 128.8, 128.5, 128.4, 128.3, 128.1, 128.0, 83.1, 70.3, 68.5, 28.3, 25.9 ppm; HRMS (ESI$^+$) [M+H]$^+$ calc'd for $C_{24}H_{23}ClNO^+$: 376.1463, found: 376.1465.

***N*-((4-bromophenyl)(tetrahydrofuran-2-yl)methyl)-1,1-diphenylmethanimine (4ga)**

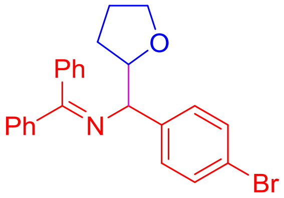

The reaction was performed following the General Procedure A with *N*-(4-bromobenzyl)-1,1-diphenylmethanimine**1g** (210.2 mg, 0.6 mmol) and 3 mL dry tetrahydrofuran **2a** (0.2 mol/L). The crude material was purified on an Agilent HPLC 1260 system using acetonitrile:H_2O (70:30 vol./vol.) as mobile phase and flow

rate of 3.0 mL/min at 254 nm to give the product **4ga** in 58% overall yield (**4ga**(major), 33 mg, 13% yield, **4ga**(minor), 33 mg, 13% yield, dr = 1:1).

4ga(major): yellow oil, R_f = 0.38 (hexanes:ethyl acetate = 5:1); ^{1}H NMR (400 MHz, Chloroform-*d*) δ 7.70 – 7.68 (m, 2H), 7.40 – 7.39 (m, 3H), 7.37 – 7.35 (m, 1H), 7.33 – 7.30 (m, 4H), 7.27 (d, *J* = 7.8 Hz, 1H), 7.23 – 7.19 (m, 1H), 7.05 – 7.03 (m, 2H), 4.47 (d, *J* = 6.0 Hz, 1H), 4.33 (q, *J* = 6.4 Hz, 1H), 3.81 – 3.75 (m, 1H), 3.73 – 3.67 (m, 1H), 2.20 – 1.94 (m, 1H), 1.92 – 1.76 (m, 3H) ppm; ^{13}C{^{1}H} NMR (100 MHz, Chloroform-*d*) δ 167.8, 142.8, 140.2, 137.0, 130.1, 128.7, 128.39, 128.36, 128.1, 127.7, 127.0, 84.3, 69.9, 68.8, 27.9, 26.0 ppm; HRMS (ESI$^+$) [M+H]$^+$ calc'd for $C_{24}H_{23}BrNO^+$: 420.0958, found: 420.0957.

4ga(minor): yellow oil, R_f = 0.36 (hexanes:ethyl acetate = 5:1); ^{1}H NMR (400 MHz, Chloroform-*d*) δ 7.69 – 7.67 (m, 2H), 7.43 – 7.40 (m, 3H), 7.34 – 7.27 (m, 6H), 7.24 – 7.22 (m, 1H), 7.12 (dd, *J* = 6.4, 2.8 Hz, 2H), 4.38 (q, *J* = 6.8 Hz, 1H), 4.34 (d, *J* = 7.2 Hz, 1H), 3.77 (td, *J* = 8.0, 5.2 Hz, 1H), 3.69 (dt, *J* = 8.0, 6.4 Hz, 1H), 1.75 – 1.68 (m, 1H), 1.67 – 1.61 (m, 2H), 1.55 – 1.46 (m, 1H) ppm; ^{13}C{^{1}H} NMR (150 MHz, Chloroform-*d*) δ 167.4, 141.9, 140.2, 137.3, 129.9, 128.8, 128.4, 128.33, 128.29, 128.26, 128.2, 128.0, 127.2, 83.4, 71.3, 68.4, 28.5, 26.0 ppm; HRMS (ESI$^+$) [M+H]$^+$ calc'd for $C_{24}H_{23}BrNO^+$: 420.0958, found: 420.0959.

_N_-([1,1'-biphenyl]-4-yl(tetrahydrofuran-2-yl)methyl)-1,1-diphenylmethanimine (4ha)

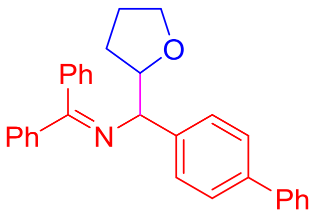

The reaction was performed following the General Procedure A with *N*-([1,1'-biphenyl]-4-ylmethyl)-1,1-diphenylmethanimine **1h** (208.5 mg, 0.6 mmol) and 3 mL dry tetrahydrofuran **2a** (0.2 mol/L). The crude material was purified on an Agilent HPLC 1260 system using acetonitrile:H_2O (70:30 vol./vol.) as mobile phase and flow rate of 3.0 mL/min at 254 nm to give the product **4ha** in 67% overall yield (**4ha**(major), 94.9 mg, 38% yield, **4ha**(minor), 73.0 mg, 29% yield, dr = 1.3:1).

4ha(major): colorless oil, R_f = 0.41 (hexanes:ethyl acetate = 5:1); ^{1}H NMR (400 MHz, Chloroform-*d*) δ 7.64 – 7.61 (m, 2H), 7.51 – 7.48 (m, 2H), 7.44 (d, *J* = 8.0 Hz, 2H), 7.35 – 7.31 (m, 7H), 7.29 – 7.21 (m, 4H), 7.01 – 6.97 (m, 2H), 4.42 (d, *J* = 6.0 Hz, 1H), 4.30 (q, *J* = 6.4 Hz, 1H), 3.75 – 3.69 (m, 1H), 3.67 – 3.61 (m, 1H), 1.91 – 1.82 (m, 2H), 1.81 – 1.71 (m, 2H) ppm; ^{13}C{^{1}H} NMR (100 MHz, Chloroform-*d*) δ 167.9, 142.0, 141.3, 140.1, 139.8, 137.0, 130.1, 128.79, 128.78, 128.5, 128.4, 128.2, 128.1, 127.2, 127.1, 84.3, 69.8, 68.8, 28.2, 26.0 ppm;

IR (thin film): 3057, 2869, 1625, 1497, 1446, 1315, 1277, 1068, 764, 697 cm^{-1}; HRMS (ESI^+) $[M+H]^+$ calc'd for $C_{30}H_{28}NO^+$: 418.2165, found: 418.2166.

4ha(minor): colorless oil, R_f = 0.41 (hexanes:ethyl acetate = 5:1); ^{1}H NMR (400 MHz, Chloroform-*d*) δ 7.63 – 7.61 (m, 2H), 7.50 (d, *J* = 7.6 Hz, 2H), 7.43 (d, *J* = 8.0 Hz, 2H), 7.35 – 7.30 (m, 7H), 7.26 – 7.20 (m, 4H), 7.06 (dd, *J* = 6.4 2.8 Hz, 2H), 7.36 – 7.31 (m, 2H), 3.73 – 3.61 (m, 2H), 1.71 – 1.56 (m, 3H), 1.52 – 1.42 (m, 1H) ppm; ^{13}C{^{1}H} NMR (100 MHz, Chloroform-*d*) δ 167.6, 141.1, 141.0, 140.1, 140.0, 137.3, 130.0, 128.9, 128.8, 128.6, 128.33, 128.32, 128.27, 128.0, 127.2, 127.14, 127.07, 83.3, 71.0, 68.4, 28.5, 26.0 ppm; IR (thin film): 3057, 2870, 1625, 1486, 1446, 1287, 1068, 836, 765, 696 cm^{-1}; HRMS (ESI^+) $[M+H]^+$ calc'd for $C_{30}H_{28}NO^+$: 418.2165, found: 418.2163.

_N_-(naphthalen-1-yl(tetrahydrofuran-2-yl)methyl)-1,1-diphenylmethanimine (4ia)

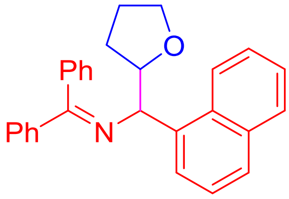

The reaction was performed following the General Procedure A with *N*-(naphthalen-1-ylmethyl)-1,1-diphenylmethanimine **1i** (208.5 mg, 0.6 mmol) and 3 mL dry tetrahydrofuran **2a** (0.2 mol/L). The crude material was purified on an Agilent HPLC 1260 system using acetonitrile:H_2O (70:30 vol./vol.) as mobile phase and flow rate of 3.0 mL/min at 254 nm to give the product **4ia** in 64% overall yield (**4ia**(major), 94.6 mg, 40% yield, **4ia**(minor), 55.7 mg, 24% yield, dr = 1.7:1).

4ia(major): colorless oil, R_f = 0.35 (hexanes:ethyl acetate = 5:1); ^{1}H NMR (400 MHz, Chloroform-*d*) δ 7.77 – 7.70 (m, 3H), 7.65 (dd, *J* = 16.0, 7.6 Hz, 3H), 7.34 – 7.18 (m, 9H), 6.88 (d, *J* = 7.2 Hz, 2H), 5.28 (d, *J* = 4.8 Hz, 1H), 4.42 (q, *J* = 6.0 Hz, 1H), 3.74 (q, *J* = 6.8 Hz, 1H), 3.60 (q, *J* = 6.8 Hz, 1H), 2.26 – 2.17 (m, 1H), 1.89 – 1.80 (m, 1H), 1.75 – 1.63 (m, 2H) ppm; ^{13}C{^{1}H} NMR (100 MHz, Chloroform-*d*) δ 168.0, 140.3, 138.8, 137.0, 133.9, 131.0, 130.1, 128.82, 128.79, 128.34, 128.31, 128.1, 127.4, 125.8, 125.7, 125.6, 125.3, 123.7, 83.5, 69.0, 65.5, 27.4, 26.3 ppm; IR (thin film): 3057, 2868, 1622, 1445, 1395, 1285, 1066, 777, 697 cm^{-1}; HRMS (ESI^+) $[M+H]^+$ calc'd for $C_{28}H_{26}NO^+$: 392.2009, found: 392.2010.

4ia(minor): colorless oil, R_f = 0.33 (hexanes:ethyl acetate = 5:1); ^{1}H NMR (400 MHz, Chloroform-*d*) δ 7.72 (d, *J* = 8.0 Hz, 1H), 7.66 – 7.62 (m, 4H), 7.42 – 7.19 (m, 10H), 6.98 (d, *J* = 7.2 Hz, 2H), 5.06 (d, *J* = 7.6 Hz, 1H), 4.62 (q, *J* = 7.2 Hz, 1H), 3.75 – 3.66 (m, 2H), 1.69 – 1.55 (m, 2H), 1.46 – 1.38 (m, 1H), 1.35 – 1.28 (m, 1H) ppm; ^{13}C{^{1}H} NMR (100 MHz, Chloroform-*d*) δ 167.8, 140.1, 138.1, 137.3, 134.1, 131.4, 130.0, 129.9, 128.7, 128.5,

128.3, 128.2, 128.0, 127.6, 126.9, 125.7, 125.5, 125.3, 124.4, 83.8, 68.4, 28.7, 26.0 ppm; IR (thin film): 3057, 2869, 1622, 1446, 1316, 1280, 1066, 777, 697 cm^{-1}; HRMS (ESI^+) $[M+H]^+$ calc'd for $C_{28}H_{26}NO^+$: 392.2009, found: 392.2007.

1,1-diphenyl-*N*-((tetrahydrofuran-2-yl)(*o*-tolyl)methyl)methanimine (4ja)

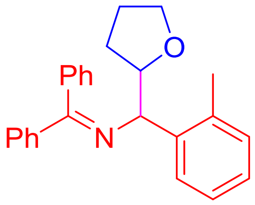

The reaction was performed following the General Procedure A with *N*-(2-methylbenzyl)-1,1-diphenylmethanimine **1j** (171.2 mg, 0.6 mmol) and 3 mL dry tetrahydrofuran **2a** (0.2 mol/L). The crude material was purified on an Agilent HPLC 1260 system using acetonitrile:H_2O (70:30 vol./vol.) as mobile phase and flow rate of 3.0 mL/min at 254 nm to give the product **4ja** in 88% overall yield (**4ja**(major), 93.8 mg, 44% yield, **4ja**(minor), 93.8 mg, 44% yield, dr = 1:1).

4ja(major): colorless oil, R_f = 0.47 (hexanes:ethyl acetate = 5:1); ^{1}H NMR (400 MHz, Chloroform-*d*) δ 7.79 – 7.73 (m, 3H), 7.46 – 7.38 (m, 6H), 7.23 (t, *J* = 7.6 Hz, 1H), 7.15 (td, *J* = 7.2, 1.6 Hz, 1H), 7.09 (d, *J* = 7.6 Hz, 1H), 7.04 –7.01 (m, 2H), 4.78 (d, *J* = 5.6 Hz, 1H), 4.36 (q, *J* = 6.0 Hz, 1H), 3.90 – 3.84 (m, 1H), 3.77 (q, *J* = 7.2 Hz, 1H), 2.29 – 2.20 (m, 1H), 2.03 – 1.83 (m, 6H) ppm; ^{13}C{^{1}H} NMR (100 MHz, Chloroform-*d*) δ 167.8, 141.6, 140.1, 137.6, 135.3, 129.99, 129.98, 128.6, 128.4, 128.2, 128.1, 127.8, 126.5, 126.1, 83.8, 68.8, 65.5, 27.8, 26.2, 19.2 ppm; IR (thin film): 3059, 2867, 1624, 1577, 1489, 1446, 1314, 1180, 1065, 780, 696 cm^{-1}; HRMS (ESI^+) $[M+H]^+$ calc'd for $C_{25}H_{26}NO^+$: 356.2009, found: 356.2009.

4ja(minor): colorless oil, R_f = 0.47 (hexanes:ethyl acetate = 5:1); ^{1}H NMR (600 MHz, Chloroform-*d*) δ 7.60 – 7.56 (m, 3H), 7.29 – 7.27 (m, 3H), 7.21 – 7.14 (m, 3H), 7.08 – 7.05 (m, 1H), 7.00 – 6.95 (m, 3H), 6.90 (d, *J* = 7.6 Hz, 1H), 4.59 (dd, *J* = 7.8, 3.0 Hz, 1H), 4.39 – 4.35 (m, 1H), 3.69 (t, *J* = 6.6 Hz, 2H), 1.76 (d, *J* = 2.4 Hz, 3H), 1.68 – 1.52 (m, 3H), 1.40 – 1.34 (m, 1H) ppm; ^{13}C{^{1}H} NMR (150 MHz, Chloroform-*d*) δ 167.2, 140.1, 137.7, 135.1, 130.2, 129.8, 129.1, 128.7, 128.3, 128.2, 128.1, 127.9, 126.5, 126.0, 83.8, 68.3, 66.4, 28.1, 25.9, 19.5 ppm; IR (thin film): 3059, 2868, 1624, 1577, 1489, 1446, 1314, 1280, 1180, 1067, 1029, 695 cm^{-1}; HRMS (ESI^+) $[M+H]^+$ calc'd for $C_{25}H_{26}NO^+$: 356.2009, found: 356.2007.

***N*-(diphenyl(tetrahydrofuran-2-yl)methyl)-1,1-diphenylmethanimine (4ka)**

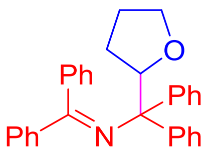

The reaction was performed following the General Procedure A with *N*-benzhydryl-1,1-diphenylmethanimine **1k** (208.5 mg, 0.6 mmol) and 3 mL dry tetrahydrofuran **2a** (0.2 mol/L). The crude material was purified on an Agilent HPLC 1260 system using acetonitrile:H_2O (70:30 vol./vol.) as mobile phase and flow rate of 3.0 mL/min at 254 nm to give the product **4ka** in 71% yield (177.9 mg).

colorless oil, R_f = 0.47 (hexanes:ethyl acetate = 10:1); ^{1}H NMR (400 MHz, Chloroform-*d*) δ 7.77 – 7.73 (m, 4H), 7.46 – 7.38 (m, 3H), 7.33 – 7.24 (m, 3H), 7.20 – 7.14 (m, 3H), 7.13 – 6.04 (m, 5H), 6.58 (d, *J* = 7.2 Hz, 2H), 4.99 (t, *J* = 7.2 Hz, 1H), 3.73 (td, *J* = 7.6, 5.2 Hz, 1H), 3.30 (q, *J* = 7.2 Hz, 1H), 2.13 – 1.95 (m, 2H), 1.70– 1.60 (m, 1H), 1.32– 1.23 (m, 1H) ppm; $^{13}C\{^1H\}$ NMR (100 MHz, Chloroform-*d*) δ 166.9, 147.9, 145.7, 142.3, 138.9, 130.6, 129.9, 129.4, 128.5, 128.0, 127.5, 127.4, 127.2, 126.8, 126.8, 126.2, 126.0, 82.0, 73.2, 69.0, 27.9, 25.7 ppm; IR (thin film): 3056, 2972, 1626, 1576, 1445, 1178, 1071, 756, 696 cm^{-1}; HRMS (ESI$^+$) $[M+H]^+$ calc'd for $C_{30}H_{28}NO^+$: 418.2165, found: 418.2167.

***N*-((1,4-dioxan-2-yl)diphenylmethyl)-1,1-diphenylmethanimine (4kc)**

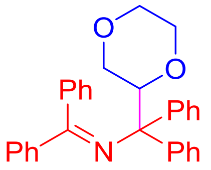

The reaction was performed following the General Procedure A with *N*-benzhydryl-1,1-diphenylmethanimine **1k** (208.5 mg, 0.6 mmol) and 3 mL dry 1,4-dioxane **2c** (0.2 mol/L). The crude material was purified on an Agilent HPLC 1260 system using acetonitrile:H_2O (70:30 vol./vol.) as mobile phase and flow rate of 3.0 mL/min at 254 nm to give the product **4kc** in 50% yield (130.1 mg).

white solid, m.p. = 153 – 154 ℃; R_f = 0.51 (hexanes:ethyl acetate = 8:1); ^{1}H NMR (400 MHz, Chloroform-*d*) δ 7.55 – 7.53 (m, 2H), 7.43 – 7.41 (m, 2H), 7.30 – 7.20 (m, 3H), 7.18 – 7.13 (m, 3H), 7.00 (t, *J* = 7.6 Hz, 1H), 6.96 – 6.92 (m, 1H), 6.90 – 6.85 (m, 6H), 6.41 (d, *J* = 7.6 Hz, 2H), 4.40 (dd, *J* = 10.0, 2.0 Hz, 1H), 3.99 (dd, *J* = 11.6, 2.0 Hz, 1H), 3.64 (dd, *J* = 8.0, 2.0 Hz, 1H), 3.48 (d, *J* = 11.6 Hz, 1H), 3.29 – 3.20 (m, 1H), 3.07 (dd, *J* = 11.6, 10.0 Hz, 1H) ppm; $^{13}C\{^1H\}$ NMR (100 MHz, Chloroform-*d*) δ 167.8, 145.8, 144.7, 141.9, 138.4, 130.24, 130.16, 129.3, 128.6, 128.1, 127.40, 127.37, 127.2, 126.9, 126.8, 126.5, 126.2, 79.2, 71.9, 68.7, 67.3, 66.5 ppm; IR (thin film): 3058, 2986, 1629, 1573, 1445, 1261, 1219, 1099, 772, 695 cm^{-1}; HRMS (ESI$^+$) $[M+H]^+$ calc'd for $C_{30}H_{28}NO_2^+$: 434.2115, found: 434.2115.

N-(1,2-diphenylethyl)-1,1-diphenylmethanimine (6aa)

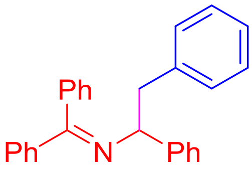

The reaction was performed following the General Procedure B with *N*-benzyl-1,1-diphenylmethanimine **1a** (81.4 mg, 0.3 mmol) and 1.5 mL dry toluene **5a** (0.2 mol/L). The crude material was purified by flash chromatography on deactivated silica gel (eluted with hexanes to diethyl ether:hexanes = 1:100) to give the product **6aa** (65.1 mg, 60%) as a yellow oil. The ^{1}H and $^{13}C\{^{1}H\}$ data for this compound match the literature data.[1]

1,1-diphenyl-*N*-(1-phenyl-2-(*o*-tolyl)ethyl)methanimine (6ab)

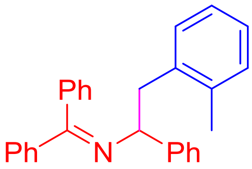

The reaction was performed following the General Procedure B with *N*-benzyl-1,1-diphenylmethanimine **1a** (81.4 mg, 0.3 mmol) and 1.5 mL dry *o*-xylene **5b** (0.2 mol/L). The crude material was purified by flash chromatography on deactivated silica gel (eluted with hexanes to diethyl ether:hexanes = 1:100) to give the product **6ab** in 71% yield (80.0 mg).

pale yellow oil, R_f = 0.59 (hexanes:ethyl acetate = 5:1); ^{1}H NMR (400 MHz, Chloroform-*d*) δ 7.56 – 7.53 (m, 2H), 7.32 (d, *J* = 7.2 Hz, 2H), 7.28 – 7.19 (m, 6H), 7.15 (t, *J* = 7.2 Hz, 3H), 7.01 – 6.86 (m, 4H), 6.34 (d, *J* = 7.2 Hz, 2H), 4.49 (dd, *J* = 9.2, 4.0 Hz, 1H), 3.15 (dd, *J* = 13.2, 9.2 Hz, 1H), 3.02 (dd, *J* = 13.2, 4.0 Hz, 1H), 1.97 (s, 3H) ppm; $^{13}C\{^{1}H\}$ NMR (100 MHz, Chloroform-*d*) δ 166.7, 144.9, 139.8, 137.5, 137.0, 136.9, 131.3, 130.1, 129.9, 128.6, 128.5, 128.1, 128.03, 128.00, 127.7, 127.2, 126.9, 126.3, 125.6, 67.1, 43.4, 19.4 ppm; IR (thin film): 3060, 1623, 1492, 1446, 1314, 1285, 1029, 771, 756, 698 cm^{-1}; HRMS (ESI^{+}) $[M+H]^{+}$ calc'd for $C_{28}H_{26}N^{+}$: 376.2060, found: 376.2058.

1,1-diphenyl-*N*-(1-phenyl-2-(*m*-tolyl)ethyl)methanimine (6ac)

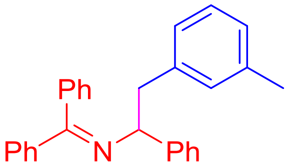

The reaction was performed following the General Procedure B with *N*-benzyl-1,1-diphenylmethanimine **1a** (81.4 mg, 0.3 mmol) and 1.5 mL dry *m*-xylene **5c** (0.2 mol/L). The crude material was purified by flash chromatography on deactivated silica gel (eluted with hexanes to diethyl ether:hexanes = 1:100) to give the product **6ac** in 70% yield (78.8 mg).

yellow oil, R_f = 0.64 (hexanes:ethyl acetate = 5:1); ^{1}H NMR (400 MHz, Chloroform-*d*) δ 7.71 – 7.68 (m, 2H), 7.47 (d, *J* = 7.2 Hz, 2H), 7.44 – 7.28 (m, 9H), 7.14 (t, *J* = 7.6 Hz, 1H), 7.04 (d, *J* = 7.6 Hz, 1H), 6.91 (d, *J* = 7.6 Hz, 2H), 6.57 (d, *J* = 7.2 Hz, 2H), 4.62 (dd, *J* = 9.2, 4.0 Hz, 1H), 3.29 (dd, *J* = 12.8, 9.2 Hz, 1H), 3.11 (dd, *J* = 12.8, 4.0 Hz, 1H), 2.30 (s, 3H) ppm; ^{13}C{^{1}H} NMR (100 MHz, Chloroform-*d*) δ 166.8, 144.7, 140.0, 139.3, 137.5, 137.0, 130.9, 129.8, 128.6, 128.5, 128.1, 128.03, 128.01, 127.8, 127.2, 127.1, 126.9, 126.7, 68.6, 46.2, 21.4 ppm; IR (thin film): 3059, 1624, 1491, 1446, 1285, 777, 698 cm^{-1}; HRMS (ESI$^+$) [M+H]$^+$ calc'd for $C_{28}H_{26}N^+$: 376.2060, found: 376.2059.

1,1-diphenyl-*N*-(1-phenyl-2-(*p*-tolyl)ethyl)methanimine (6ad)

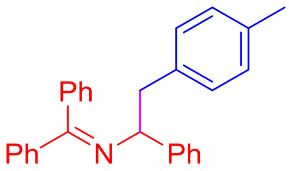

The reaction was performed following the General Procedure B with *N*-benzyl-1,1-diphenylmethanimine **1a** (81.4 mg, 0.3 mmol) and 1.5 mL dry *p*-xylene **5d** (0.2 mol/L). The crude material was purified by flash chromatography on deactivated silica gel (eluted with hexanes to diethyl ether:hexanes = 1:100) to give the product **6ad** in 75% yield (84.5 mg).

yellow oil, R_f = 0.58 (hexanes:ethyl acetate = 5:1); ^{1}H NMR (600 MHz, Chloroform-*d*) δ 7.70 – 7.68 (m, 2H), 7.43 – 7.31 (m, 10H), 7.30 – 7.27 (m, 1H), 7.04 (d, *J* = 7.8 Hz, 2H), 6.95 (d, *J* = 7.8 Hz, 2H), 6.61 (d, *J* = 7.2 Hz, 2H), 4.59 (dd, *J* = 9.0, 4.2 Hz, 1H), 3.26 (dd, *J* = 13.2, 9.0 Hz, 1H), 3.10 (dd, *J* = 13.2, 4.2 Hz, 1H), 2.35 (s, 3H) ppm; ^{13}C{^{1}H} NMR (150 MHz, Chloroform-*d*) δ 166.7, 144.7, 140.1, 137.1, 136.3, 135.4, 129.90, 129.86, 128.8, 128.7, 128.4, 128.13, 128.08, 128.0, 127.8, 127.3, 126.9, 68.8, 45.8, 21.2 ppm; IR (thin film): 3058, 2919, 1625, 1514, 1446, 1314, 1179, 1029, 778, 698 cm^{-1}; HRMS (ESI$^+$) [M+H]$^+$ calc'd for $C_{28}H_{26}N^+$: 376.2060, found: 376.2058.

***N*-(2-(3,5-dimethylphenyl)-1-phenylethyl)-1,1-diphenylmethanimine (6ae)**

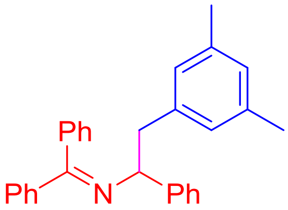

The reaction was performed following the General Procedure B with *N*-benzyl-1,1-diphenylmethanimine **1a** (81.4 mg, 0.3 mmol) and 1.5 mL dry mesitylene **5e** (0.2 mol/L). The crude material was purified by flash chromatography on deactivated silica gel (eluted with hexanes to diethyl ether:hexanes = 1:100) to give the product **6ae** in 73% yield (85.3 mg).

pale yellow solid, m.p. = 83 – 84 ℃; R_f = 0.60 (hexanes:ethyl acetate = 5:1); ^{1}H NMR (600 MHz, Chloroform-*d*) δ 7.65 (d, *J* = 7.8 Hz, 2H), 7.45 (d, *J* = 7.8 Hz, 2H), 7.40 – 7.27 (m, 9H), 6.84 (s, 1H), 6.69 (s, 2H), 6.54 (d, *J* = 6.6 Hz, 2H), 4.59 (dd, *J* = 9.6, 4.2 Hz, 1H), 3.22 (dd, *J* = 13.2, 9.6 Hz, 1H), 3.05 (dd, *J* = 13.2, 4.2 Hz, 1H), 2.24 (s, 6H) ppm; ^{13}C{^{1}H} NMR (150 MHz, Chloroform-*d*) δ 166.8, 144.9, 140.2, 139.2, 137.4, 137.2, 129.8, 128.7, 128.5, 128.1, 128.00, 127.97, 127.9, 127.6, 127.3, 126.9, 68.6, 46.2, 21.2 ppm; IR (thin film): 3058, 2917, 1624, 1491, 1446, 1284, 1029, 771, 698 cm^{-1}; HRMS (ESI$^+$) [M+H]$^+$ calc'd for $C_{29}H_{28}N^+$: 390.2216, found: 390.2214.

_N_-(2-(naphthalen-1-yl)-1-phenylethyl)-1,1-diphenylmethanimine (6af)

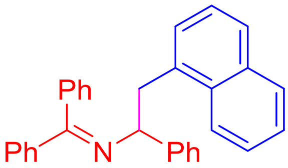

The reaction was performed following the General Procedure B with *N*-benzyl-1,1-diphenylmethanimine **1a** (81.4 mg, 0.3 mmol) and 1.5 mL dry 1-methylnaphthalene **5f** (0.2 mol/L). The crude material was purified by flash chromatography on deactivated silica gel (eluted with hexanes to diethyl ether:hexanes = 1:100) to give the product **6af** in 45% yield (55.5 mg).

colorless oil, R_f = 0.63 (hexanes:ethyl acetate = 5:1); ^{1}H NMR (400 MHz, Chloroform-*d*) δ 7.73 (d, *J* = 8.0 Hz, 1H), 7.68 (d, *J* = 8.4 Hz, 1H), 7.61 (d, *J* = 8.0 Hz, 1H), 7.50 (d, *J* = 7.2 Hz, 2H), 7.38 – 7.31 (m, 3H), 7.28 – 7.20 (m, 8H), 7.11 – 7.05 (m, 2H), 6.90 (t, *J* = 7.6 Hz, 2H), 6.12 (s, 2H), 4.69 (t, *J* = 6.4 Hz, 1H), 3.52 (d, *J* = 6.0 Hz, 2H) ppm; ^{13}C{^{1}H} NMR (100 MHz, Chloroform-*d*) δ 166.9, 144.8, 139.8, 136.6, 135.2, 133.9, 132.5, 129.9, 129.2, 128.6, 128.3, 128.0, 127.8, 127.29, 127.26, 127.0, 125.7, 125.4, 125.3, 124.0, 67.1, 43.1 ppm; IR (thin film): 3060, 2927, 1622, 1490, 1446, 1314, 1029, 791, 776, 698 -cm^{-1}; HRMS (ESI$^+$) [M+H]$^+$ calc'd for $C_{31}H_{26}N^+$: 412.2060, found: 412.2059.

N-(1,2-di-p-tolylethyl)-1,1-diphenylmethanimine (6ld)

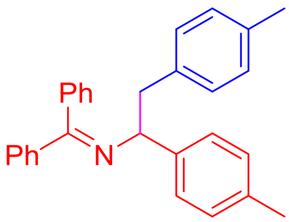

The reaction was performed following the General Procedure B with *N*-(4-methylbenzyl)-1,1-diphenylmethanimine **1l** (85.6 mg, 0.3 mmol) and 1.5 mL dry *p*-xylene **5d** (0.2 mol/L). The crude material was purified by flash chromatography on deactivated silica gel (eluted with hexanes to diethyl ether:hexanes = 1:100) to give the product **6ld** in 74% yield (86.5 mg).

colorless oil, R_f = 0.67 (hexanes:ethyl acetate = 5:1); ^{1}H NMR (400 MHz, Chloroform-*d*) δ 7.67 (d, *J* = 7.2 Hz, 2H), 7.42 – 7.28 (m, 8H), 7.17 (d, *J* = 7.6 Hz, 2H), 7.04 (d, *J* = 7.6 Hz, 2H), 6.95 (d, *J* = 7.6 Hz, 2H), 6.59 (d, *J* = 7.2 Hz, 2H), 4.55 (dd, *J* = 9.2, 4.4 Hz, 1H), 3.25 (t, *J* = 11.2 Hz, 1H), 3.08 (dd, *J* = 13.2, 4.4 Hz, 1H), 2.39 (s, 3H), 2.35 (s, 3H) ppm; ^{13}C{^{1}H} NMR (100 MHz, Chloroform-*d*) δ 166.5, 141.8, 140.1, 137.1, 136.4, 135.4, 129.9, 129.8, 129.1, 128.8, 128.6, 128.1, 128.0, 127.8, 127.2, 68.5, 45.8, 21.3, 21.2 ppm; IR (thin film): 3054, 2920, 1624, 1512, 1446, 1314, 1286, 1029, 718, 695 cm^{-1}; HRMS (ESI$^+$) [M+H]$^+$ calc'd for $C_{29}H_{28}N^+$: 390.2216, found: 390.2213.

N-(1-(4-methoxyphenyl)-2-(p-tolyl)ethyl)-1,1-diphenylmethanimine (6cd)

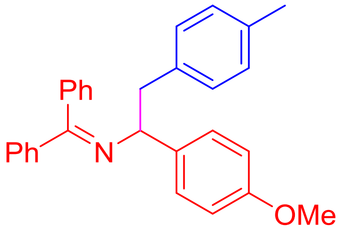

The reaction was performed following the General Procedure B with *N*-(4-methoxybenzyl)-1,1-diphenylmethanimine **1c** (90.4 mg, 0.3 mmol) and 1.5 mL dry *p*-xylene **5d** (0.2 mol/L). The crude material was purified by flash chromatography on deactivated silica gel (eluted with hexanes to diethyl ether:hexanes = 1:100) to give the product **6cd** in 73% yield (88.8 mg).

colorless oil, R_f = 0.52 (hexanes:ethyl acetate = 5:1); ^{1}H NMR (600 MHz, Chloroform-*d*) δ 7.54 – 7.52 (m, 2H), 7.26 – 7.16 (m, 8H), 6.88 (d, *J* = 7.8 Hz, 2H), 6.79 – 6.74 (m, 4H), 6.48 (d, *J* = 7.2 Hz, 2H), 4.39 (dd, *J* = 9.0, 4.8 Hz, 1H), 3.70 (d, *J* = 1.2 Hz, 3H), 3.08 (dd, *J* = 13.2, 8.4 Hz, 1H), 2.92 (dd, *J* = 13.2, 4.8 Hz, 1H), 2.20 (s, 3H) ppm; ^{13}C{^{1}H} NMR (150 MHz, Chloroform-*d*) δ 166.4, 158.6, 140.2, 137.2, 137.0, 136.4, 135.4, 129.9, 129.8, 128.8, 128.6, 128.3, 128.13, 128.06, 128.0, 127.8, 113.8, 68.1, 55.4, 45.9, 21.2 ppm; IR (thin film): 3056, 2919,

1611, 1510, 1445, 1314, 1245, 1173, 1036, 779, 696 cm^{-1}; HRMS (ESI^+) $[M+H]^+$ calc'd for $C_{29}H_{28}NO^+$: 406.2165, found: 406.2165.

1,1-diphenyl-*N*-(1-(*o*-tolyl)-2-(*p*-tolyl)ethyl)methanimine (6jd)

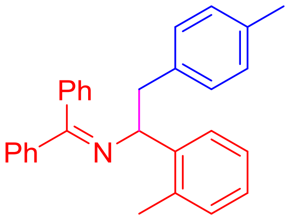

The reaction was performed following the General Procedure B with *N*-(2-methylbenzyl)-1,1-diphenylmethanimine **1j** (85.6 mg, 0.3 mmol) and 1.5 mL dry *p*-xylene **5d** (0.2 mol/L). The crude material was purified by flash chromatography on deactivated silica gel (eluted with hexanes to diethyl ether:hexanes = 1:100) to give the product **6jd** in 70% yield (81.8 mg).

colorless oil, R_f = 0.67 (hexanes:ethyl acetate = 5:1); ^{1}H NMR (600 MHz, Chloroform-*d*) δ 7.95 (d, *J* = 7.8 Hz, 1H), 7.76 – 7.75 (m, 2H), 7.45 – 7.39 (m, 4H), 7.36 – 7.31 (m, 3H), 7.20 (td, *J* = 7.2, 1.2 Hz, 1H), 7.12 (d, *J* = 7.2 Hz, 1H), 7.08 (d, *J* = 7.8 Hz, 2H), 6.99 (d, *J* = 7.8 Hz, 2H), 6.64 (d, *J* = 7.2 Hz, 2H), 4.84 (dd, *J* = 9.0, 4.2 Hz, 1H), 3.26 (dd, *J* = 13.2, 8.4 Hz, 1H), 3.07 (dd, *J* = 13.2, 4.2 Hz, 1H), 2.38 (s, 3H), 1.95 (s, 3H) ppm; ^{13}C{^{1}H} NMR (150 MHz, Chloroform-*d*) δ 166.7, 143.5, 140.1, 137.6, 136.5, 135.4, 134.3, 130.1, 129.8, 129.7, 128.8, 128.6, 128.2, 128.0, 127.8, 127.6, 126.34, 126.30, 64.7, 45.5, 21.2, 19.0 ppm; IR (thin film): 3057, 2920, 1623, 1514, 1466, 1314, 1289, 1029, 779, 695 cm^{-1}; HRMS (ESI^+) $[M+H]^+$ calc'd for $C_{29}H_{28}N^+$: 390.2216, found: 390.2213.

Gram scale sequential one-pot ketimine synthesis/alkylation of C(sp^3)–H bonds procedure

An oven-dried 100 mL Schlenk tube equipped with a stir bar was sealed with a rubber septum and degassed by nitrogen purge (repeated three times). Tetrahydrofuran (10 mL) was added under nitrogen via syringe through the rubber septum. Benzophenone imine (724.9 mg, 4.0 mmol) and benzyl amine (428.6 mg, 4.0 mmol) were added under nitrogen via syringe through the rubber septum at room temperature. The reaction was heated and stirred at 50 °C for 12 h, cooled to room temperature, the solvent was removed *in vacuo* and the Schlenk tube was filled with nitrogen. A solution (prepared in the glove box) of 5-(*tert*-butyl)-2-iodo-1,3-dimethylbenzene **3f** (2.30 g, 8.0 mmol) in 10 mL anhydrous tetrahydrofuran was added to the Schlenk tube via syringe through the rubber septum. Next, a solution of $NaN(SiMe_3)_2$ (2.20 g, 12.0 mmol) in 10 mL anhydrous tetrahydrofuran was added by syringe through the rubber septum at room temperature. Upon addition of the base, the reaction turned

purple. The reaction mixture was then heated and stirred for 1 h at 80 ℃. The reaction flask was then cooled to room temperature, opened to air, and quenched with 5 mL of H_2O, the solution color changed from purple to yellow. The layers were separated and the aqueous layer was extracted with DCM (3 X 25 mL). The combined organic layers were concentrated in *vacuo*. The crude material was loaded onto a deactivated silica gel column via pipette and purified by flash chromatography (eluted with hexanes to diethyl ether:hexanes = 1:50) to give the product **4aa** in 86% overall yield. (0.65 g, 48% yield, α-**4aa**(minor), 0.38 g, 28% yield, dr = 1.7:1); β-**4aa**(0.14g, 10% yield, dr = 1.5:1)).

1,1-diphenyl-*N*-(phenyl(tetrahydrofuran-3-yl)methyl)methanimine (β-4aa)

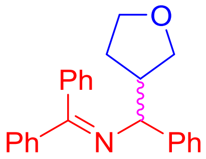

colorless oil, R_f = 0.40 (hexanes:ethyl acetate = 8:1); Diastereomeric ratio was determined based on tetrahydrofuran group H^a (1H, ~ 3.9 – 3.2 ppm) and H^b (1H, ~ 2.0 – 1.6 ppm), see ^{1}H spectra (Page S74) for determination of diastereomeric ratio; HRMS (ESI^+) $[M+H]^+$ calc'd for $C_{24}H_{24}NO^+$: 342.1852, found: 342.1850.

Imine product hydrolysis

Hydrolysis of product 4aa: An oven-dried 50 mL round bottom flask equipped with a stir bar was charged with **4aa** (68.3 mg, 0.2 mmol). 25 mL of 50 percent aqueous tetrahydrofuran taken up by syringe and added to the reaction flask under air. To this solution 3 equiv of $NH_2OH \cdot HCl$ (41.7 mg, 0.6 mmol) were added and the reaction mixture and it was stirred 12 h at ambient temperature. The THF was evaporated under reduced pressure to leave an aqueous solution and the remaining residue was treated with 30% aqueous HCl until pH = 1 was reached. The aqueous phase was extracted with ethyl acetate. The water phase was adjusted to pH = 10 with 30% aqueous NaOH and extracted with DCM (3 X 15 mL). The combined organic layers were concentrated in *vacuo*, loaded onto a deactivated silica gel column via pipette and purified by flash chromatography (ethyl acetate to ethyl acetate:methanol = 5:1) to give the amine product **7aa** (34.0 mg, 96% yield) as a colorless oil.

phenyl(tetrahydrofuran-2-yl)methanamine (7aa):

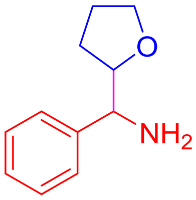

R_f = 0.36 (ethyl acetate:methanol = 1:1); ^{1}H NMR (400 MHz, Chloroform-*d*) δ 7.28 (d, *J* = 7.6 Hz, 2H), 7.23 (t, *J* = 7.2 Hz, 2H), 7.19 – 7.14 (m, 1H), 4.04 (d, *J* = 4.8 Hz, 1H), 3.97 (q, *J* = 6.0 Hz, 1H), 3.75 (q, *J* = 7.2 Hz, 1H), 3.66 (q, *J* = 7.2 Hz, 1H), 1.75 – 1.60 (m, 6H) ppm; ^{13}C{^{1}H} NMR (100 MHz, Chloroform-*d*) δ 142.8, 128.3, 127.2, 127.1, 83.5, 68.7, 58.3, 26.2, 26.0 ppm; IR (thin film): 3377, 3060, 2973, 2869, 1602, 1493, 1452, 1185, 1061, 702 cm^{-1}; HRMS (ESI$^+$) [M+H]$^+$ calc'd for $C_{11}H_{16}NO^+$ 178.1226, found 178.1225.

Mechanistic study

b. Evidence of HAT in alkylation of C(sp^3)-H bonds. Reaction of 1a with THF-*d*$_8$.

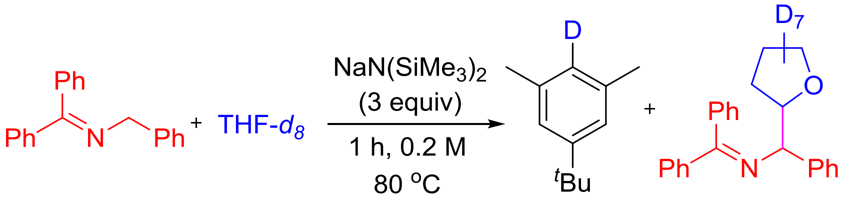

The reaction was performed following the General Procedure A with *N*-benzyl-1,1-diphenylmethanimine **1a** (162.8 mg, 0.6 mmol) and 3 mL dry tetrahydrofuran-*d*$_8$ D-**2a** (0.2 mol/L). The crude material was purified on an Agilent HPLC 1260 system using acetonitrile:H_2O (70:30 vol./vol.) as mobile phase and flow rate of 3.0 mL/min at 254 nm to give the product **8** (36.7 mg, 68% yield) as a colorless oil and the product D-**4aa** in 64% overall yield (D-**4aa**(major), 78.1 mg, 38% yield, D-**4aa**(minor), 52.0 mg, 26% yield, dr = 1.5:1).

1-(*tert*-butyl)-3-methylbenzene-4-*d* (8)

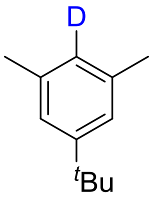

colorless oil; R_f = 0.62 (hexanes = 8:1); ^{1}H NMR (600 MHz, Chloroform-*d*) δ 7.15 (s, 2H), 2.45 (s, 6H), 1.45 (s, 9H) ppm; ^{13}C{^{1}H} NMR (150 MHz, Chloroform-*d*) δ 151.2, 137.3, 123.2, 34.5, 31.6, 21.6 ppm; HRMS (EI$^+$) [M]$^{+}$ calc'd for $C_{12}H_{17}D^{+}$: 163.1471, found: 163.1474.

Elemental Composition Report **Page 1**

Single Mass Analysis
Tolerance = 10.0 PPM / DBE: min = -10.0, max = 120.0
Selected filters: None

Monoisotopic Mass, Odd and Even Electron Ions
26 formula(e) evaluated with 1 results within limits (up to 51 closest results for each mass)
Elements Used:
C: 0-200 1H: 0-400 2H: 1-1

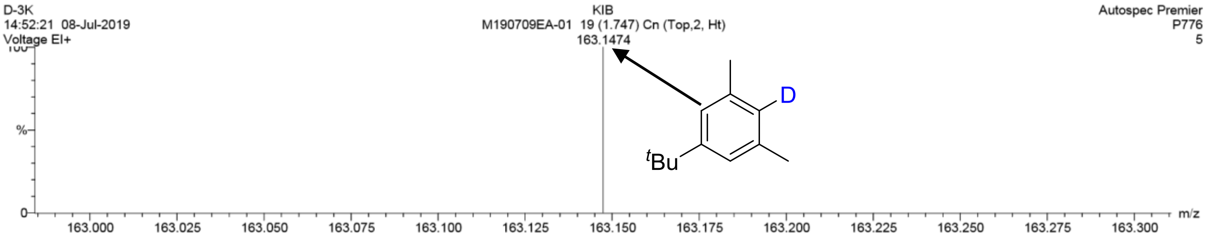

Minimum:				-10.0		
Maximum:		200.0	10.0	120.0		
Mass	Calc. Mass	mDa	PPM	DBE	i-FIT	Formula
163.1474	163.1471	0.3	1.8	4.0	5546025.5	C12 1H17 2H

1,1-diphenyl-*N*-(phenyl(tetrahydrofuran-2-yl-*d*$_7$)methyl)methanimine (D-**4aa**)

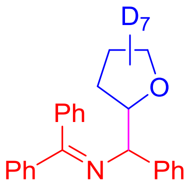

D-**4aa**(major): white solid, m.p. = 112 – 113 ℃; R$_f$ = 0.38 (hexanes:ethyl acetate = 5:1); ^{1}H NMR (400 MHz, Chloroform-*d*) δ 7.70 – 7.68 (m, 2H), 7.39 – 7.26 (m, 10H), 7.22 – 7.18 (m, 1H), 7.04 (dd, *J* = 6.4, 2.8 Hz, 2H), 4.47 (s, 1H) ppm; ^{13}C{^{1}H} NMR (100 MHz, Chloroform-*d*) δ 167.8, 142.8, 140.1, 137.0, 130.0, 128.7, 128.4, 128.3, 128.1, 127.7, 126.9, 69.7 ppm; IR (thin film): 3059, 2886, 1578, 1491, 1446, 1315, 1286, 1049, 780, 698 cm^{-1}; HRMS (ESI$^+$) [M+H]$^+$ calc'd for $C_{24}H_{17}D_7NO^+$: 349.2292, found: 349.2290.

D-**4aa**(minor): colorless oil, R$_f$ = 0.36 (hexanes:ethyl acetate = 5:1); ^{1}H NMR (400 MHz, Chloroform-*d*) δ 7.73 – 7.71 (m, 2H), 7.46 – 7.43 (m, 3H), 7.40 – 7.24 (m, 8H), 7.15 (dd, *J* = 6.4, 2.8 Hz, 2H), 4.37 (s, 1H) ppm; ^{13}C{^{1}H} NMR (100 MHz, Chloroform-*d*) δ 167.4, 141.9, 140.1, 137.3, 129.9, 128.8, 128.34, 128.31, 128.3, 128.22, 128.21, 128.0, 127.1, 71.2 ppm; IR (thin film): 3059, 2926, 1626, 1577, 1491, 1446, 1278, 1048, 779, 700 cm^{-1}; HRMS (ESI$^+$) [M+H]$^+$ calc'd for $C_{24}H_{17}D_7NO^+$: 349.2292, found: 349.2292.

X-ray crystal structures of compound 4aa

CCDC 1943984 contains the supplementary crystallographic data for compound **4aa**. The data can be obtained free of charge from The Cambridge Crystallographic Data Center via www.ccdc.cam.ac.uk/data_request/cif.

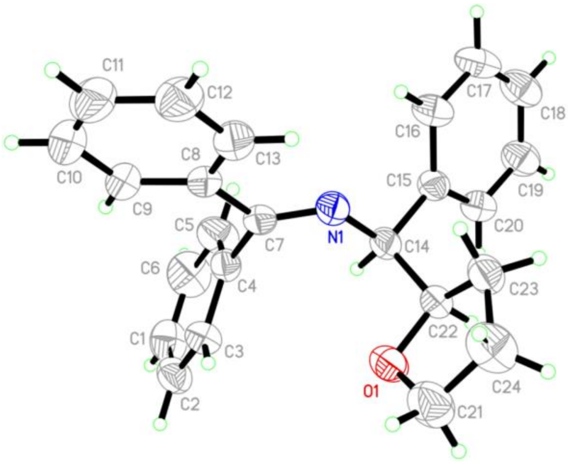

X-ray crystal structures of compound 4af

CCDC 1944046 contains the supplementary crystallographic data for compound **4af**. The data can be obtained free of charge from The Cambridge Crystallographic Data Center via www.ccdc.cam.ac.uk/data_request/cif.

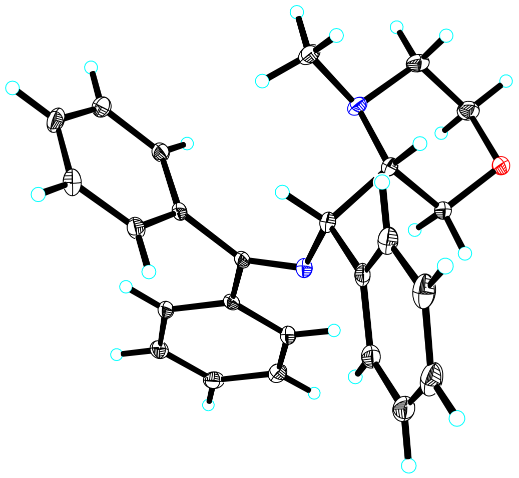

Supplementary references

(1) Fields, W. H.; Chruma, J. J., *Org. Lett.* **2010,** *12* (2), 316-319.

NMR Spectra

Figure S1. **^{1}H NMR spectra (400 MHz, Chloroform-*d*) of 1,1-diphenyl-*N*-(phenyl(tetrahydrofuran-2-yl)methyl)methanimine (4aa**(major)**):**

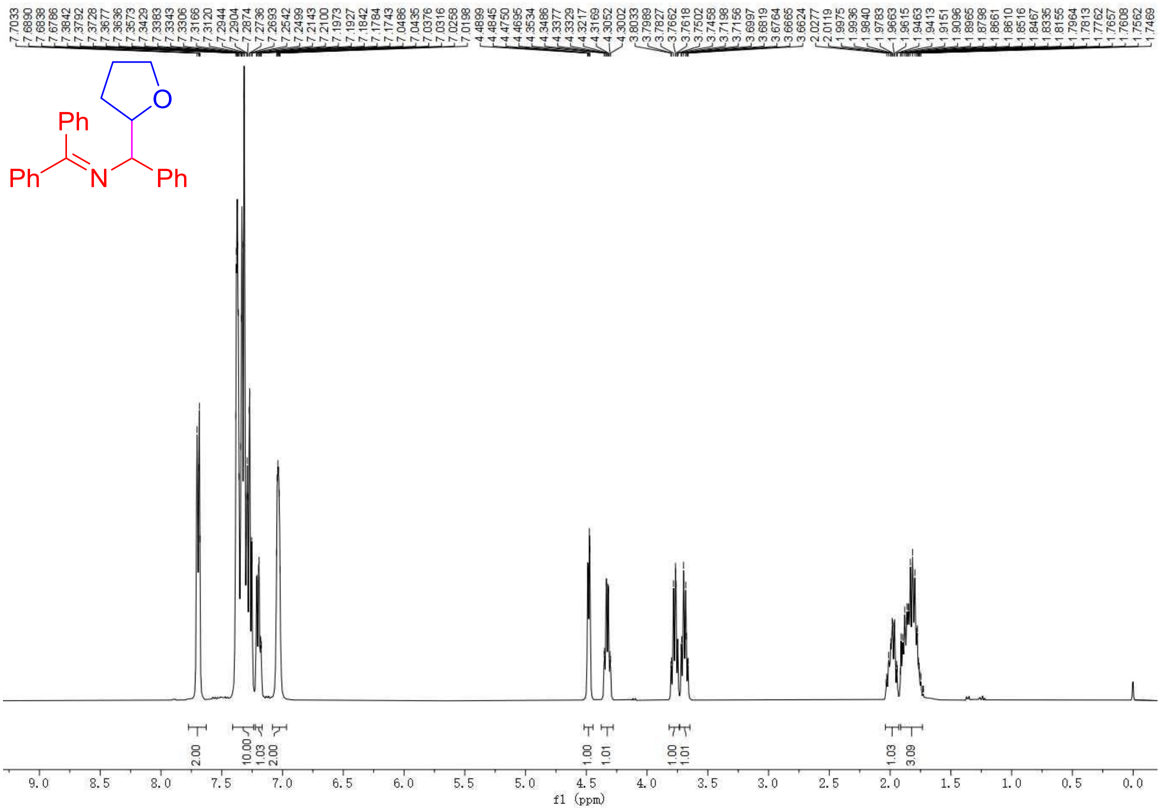

Figure S2. **^{13}C{^{1}H} NMR spectra (100 MHz, Chloroform-*d*) of 1,1-diphenyl-*N*-(phenyl(tetrahydrofuran-2-yl)methyl)methanimine (4aa**(major)**):**

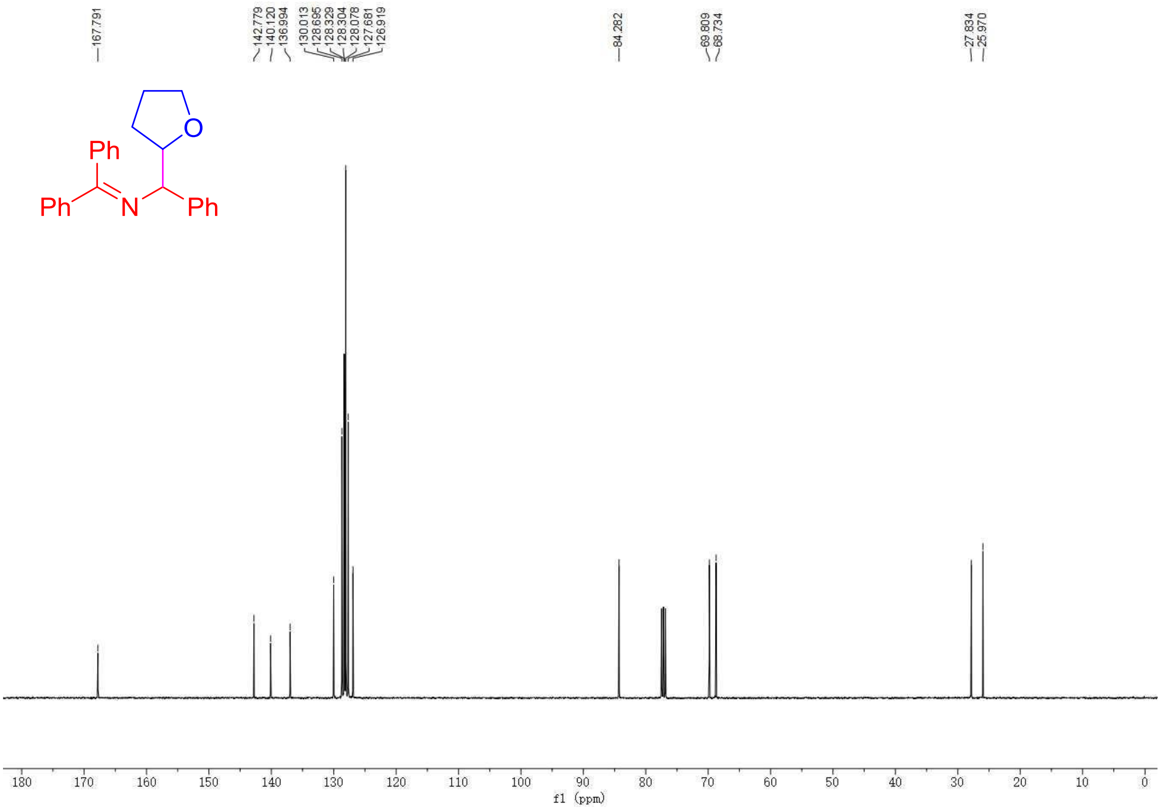

Figure S3. ^{1}**H NMR spectra (400 MHz, Chloroform-*d*) of 1,1-diphenyl-*N*-(phenyl(tetrahydrofuran-2-yl)methyl)methanimine (4aa**(minor)**):**

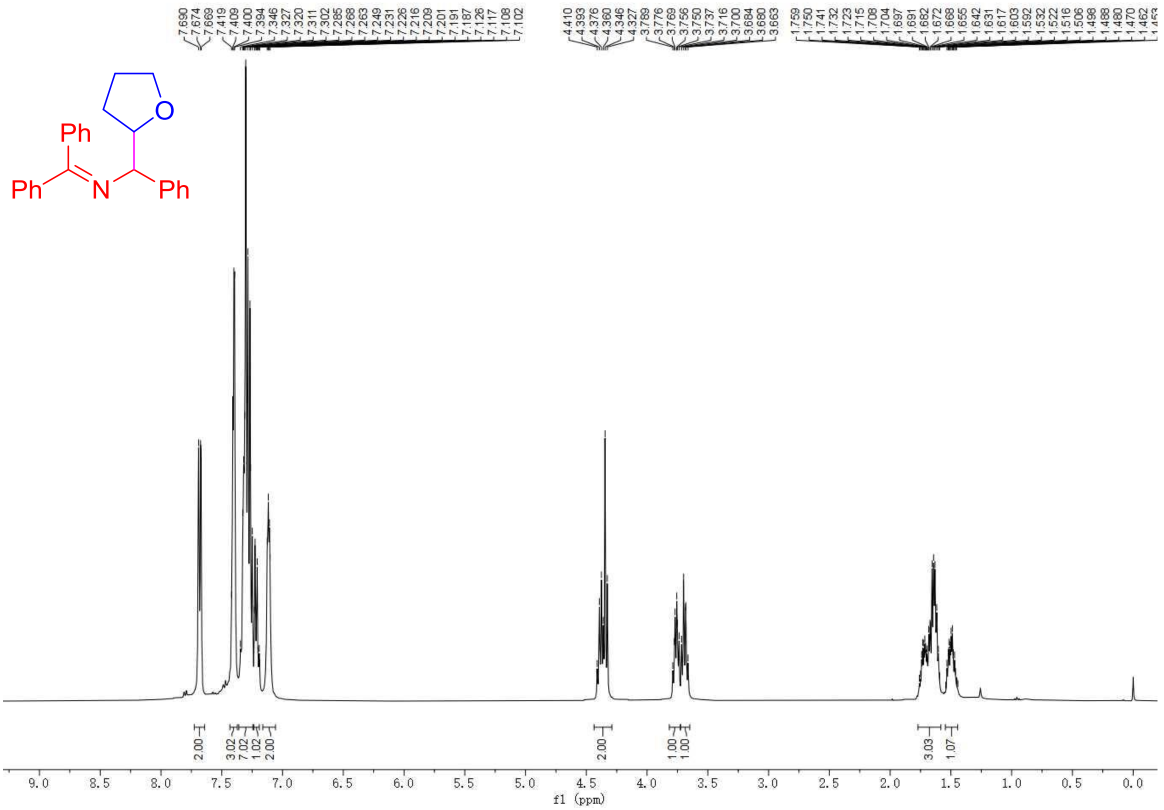

Figure S4. ^{13}**C{**^{1}**H} NMR spectra (100 MHz, Chloroform-*d*) of 1,1-diphenyl-*N*-(phenyl(tetrahydrofuran-2-yl)methyl)methanimine (4aa**(minor)**):**

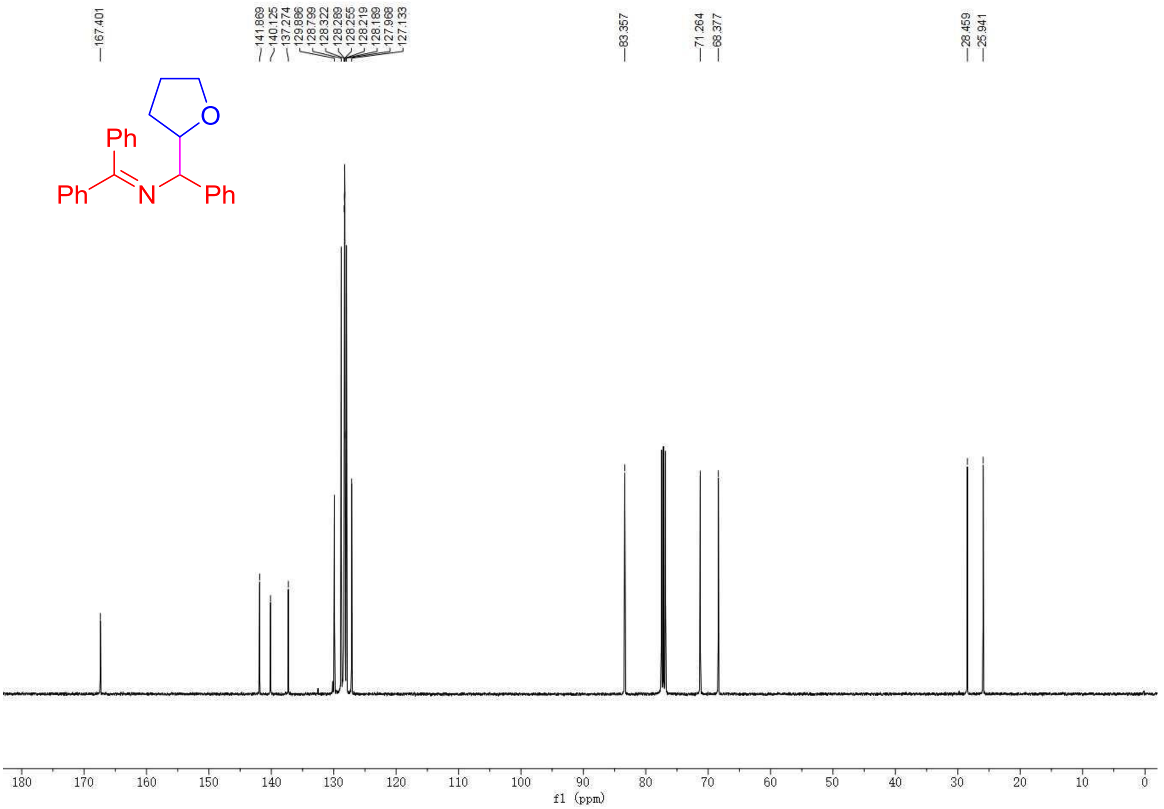

Figure S5. ^{1}**H NMR spectra (600 MHz, Chloroform-*d*) of *N*-((3,3-dimethyloxetan-2-yl)(phenyl)methyl)-1,1-diphenylmethanimine (4ab**(major)**):**

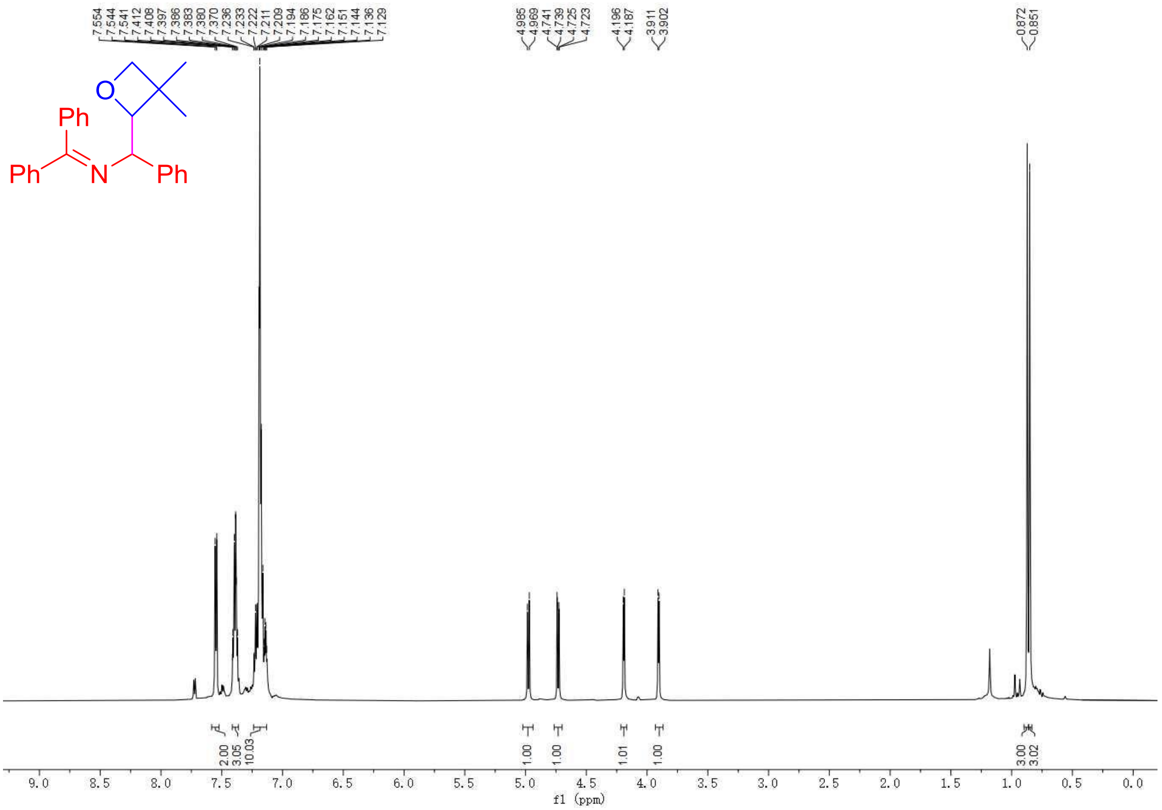

Figure S6. ^{13}**C{**^{1}**H} NMR spectra (150 MHz, Chloroform-*d*) of *N*-((3,3-dimethyloxetan-2-yl)(phenyl)methyl)-1,1-diphenylmethanimine(4ab**(major)**):**

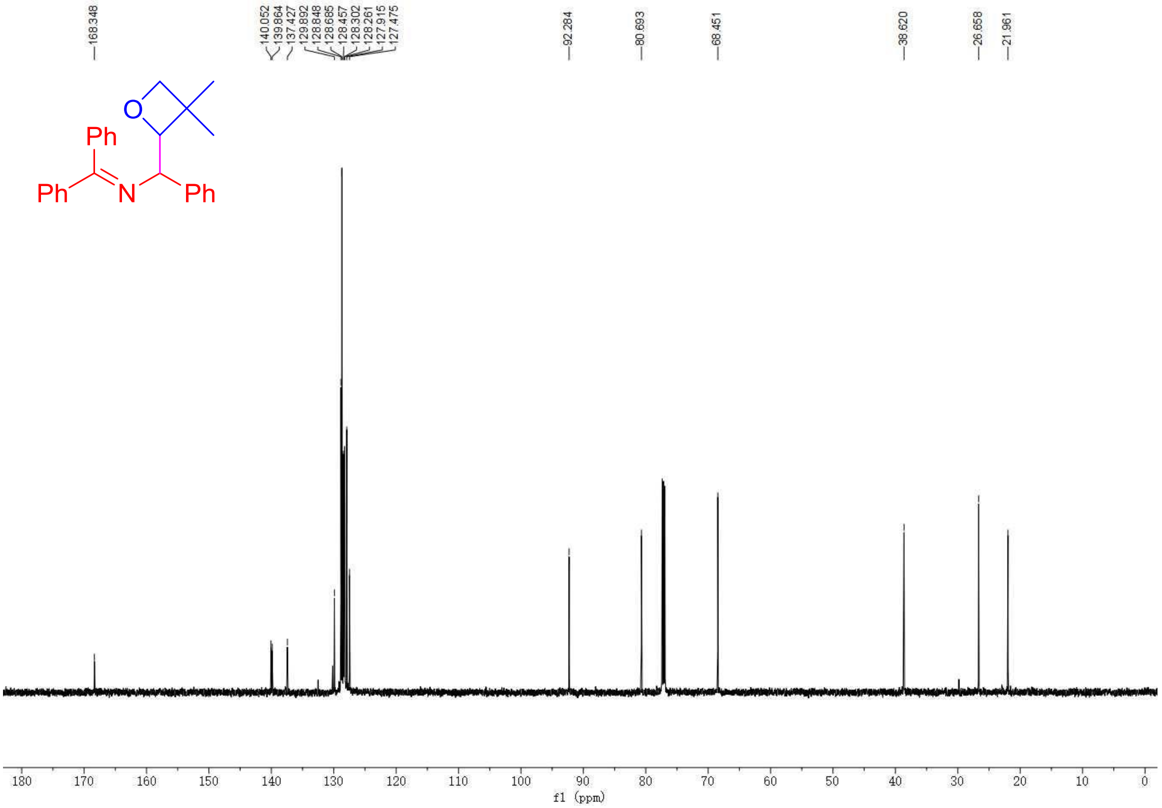

Figure S7. ^{1}**H NMR spectra (600 MHz, Chloroform-*d*) of *N*-((3,3-dimethyloxetan-2-yl)(phenyl)methyl)-1,1-diphenylmethanimine (4ab**(minor)**):**

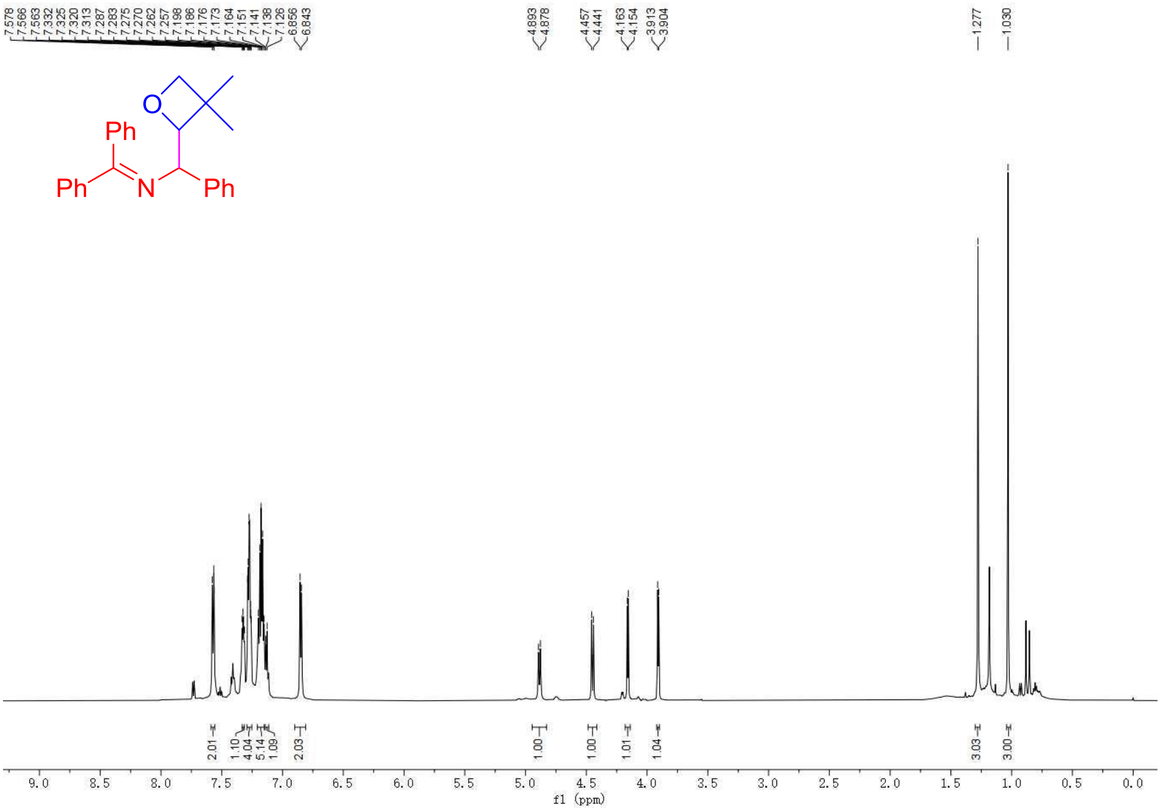

Figure S8. ^{13}**C{**^{1}**H} NMR spectra (150 MHz, Chloroform-*d*) of *N*-((3,3-dimethyloxetan-2-yl)(phenyl)methyl)-1,1-diphenylmethanimine (4ab**(minor)**):**

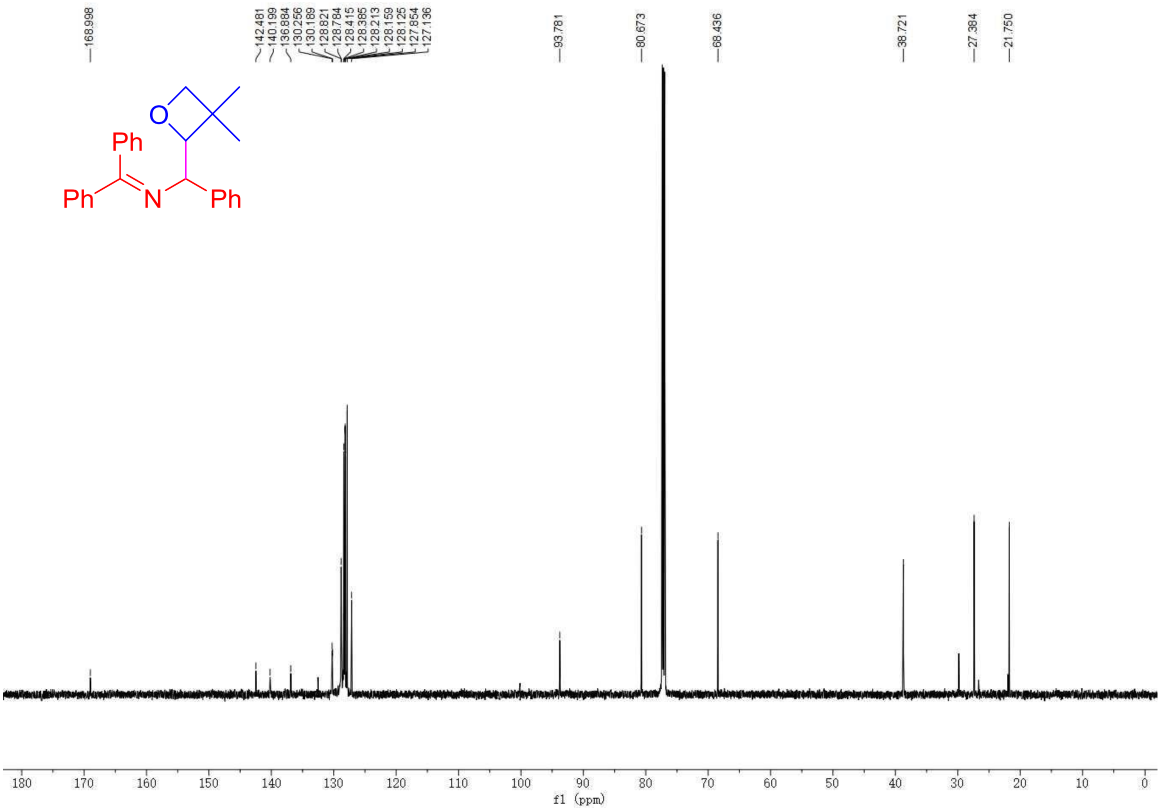

Figure S9. ^{1}H NMR spectra (400 MHz, Chloroform-*d*) of *N*-((1,4-dioxan-2-yl)(phenyl)methyl)-1,1-diphenylmethanimine (4ac(major)**):**

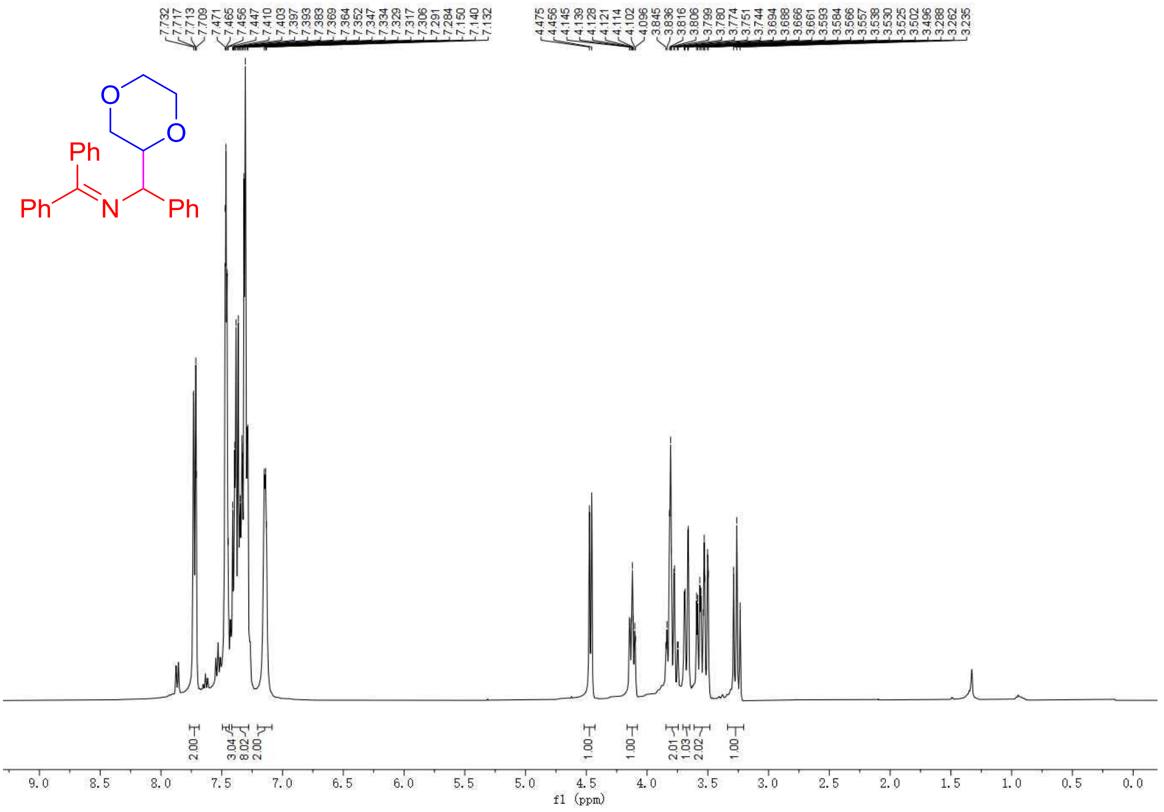

Figure S10. $^{13}C\{^{1}H\}$ NMR spectra (100 MHz, Chloroform-*d*) of *N*-((1,4-dioxan-2-yl)(phenyl)methyl)-1,1-diphenylmethanimine (4ac(major)**):**

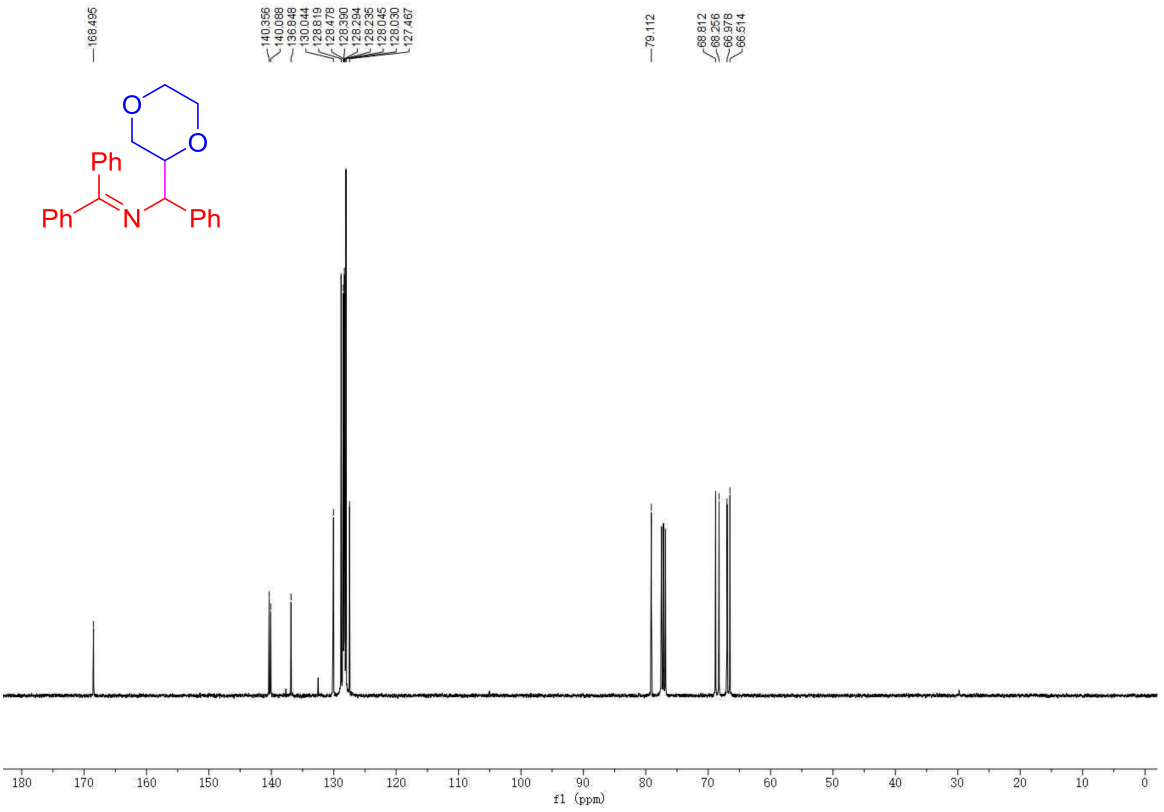

Figure S11. ^{1}H NMR spectra (400 MHz, Chloroform-*d*) of *N*-((1,4-dioxan-2-yl)(phenyl)methyl)-1,1-diphenylmethanimine (4ac(minor)):

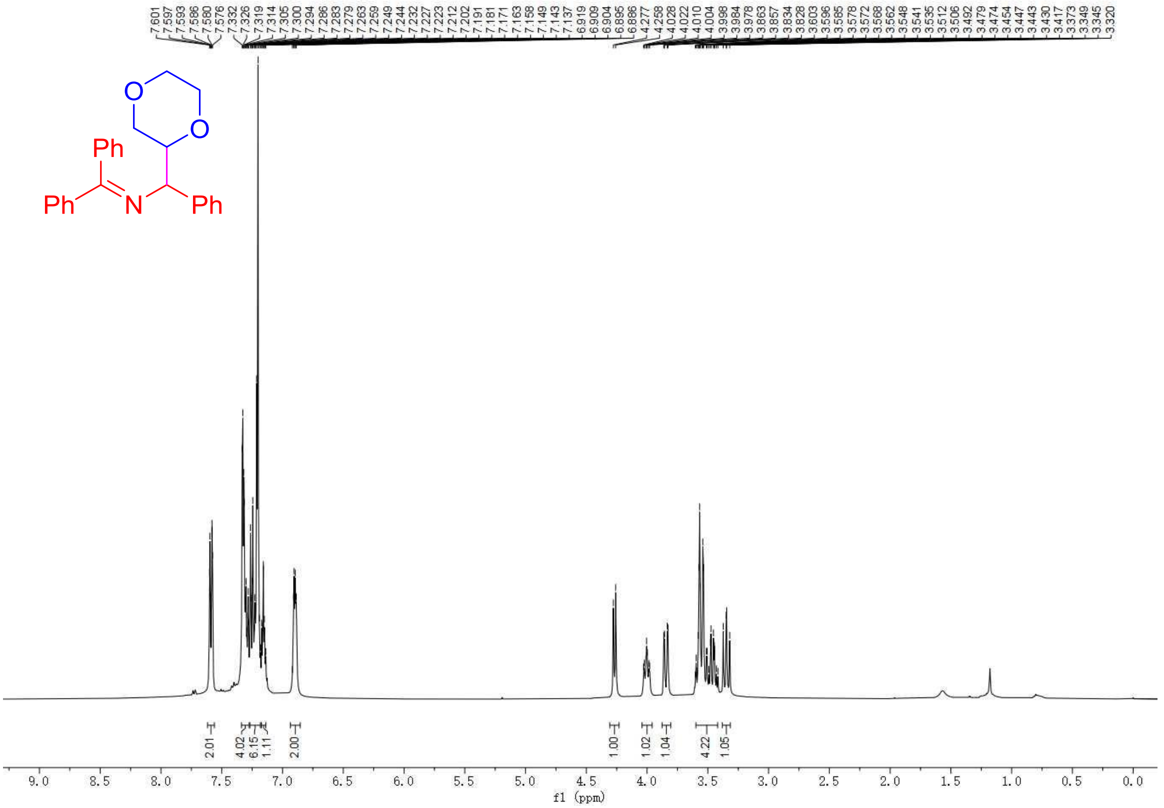

Figure S12. ^{13}C{^{1}H} NMR spectra (100 MHz, Chloroform-*d*) of *N*-((1,4-dioxan-2-yl)(phenyl)methyl)-1,1-diphenylmethanimine (4ac(minor)):

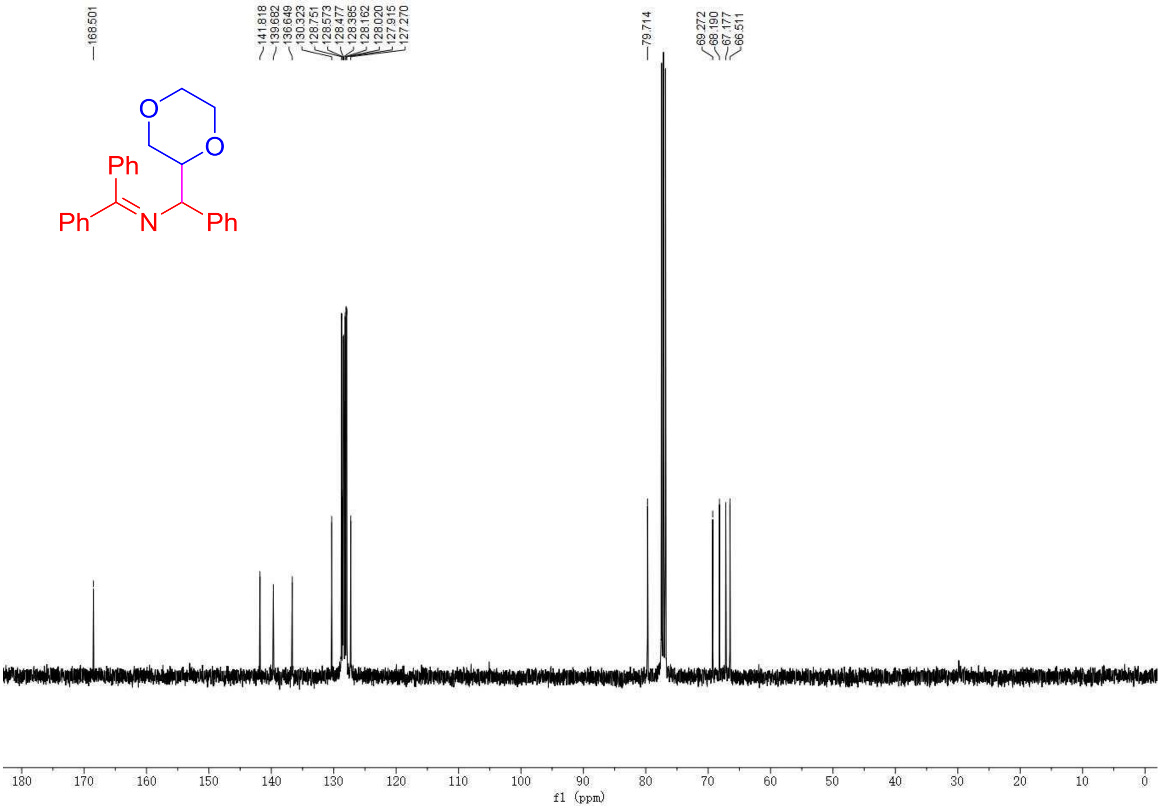

Figure S13. **^{1}H NMR spectra (600 MHz, Chloroform-*d*) of *N*-(2,3-dimethoxy-1-phenylpropyl)-1,1-diphenylmethanimine (4ad,** dr = 1.5:1**):**

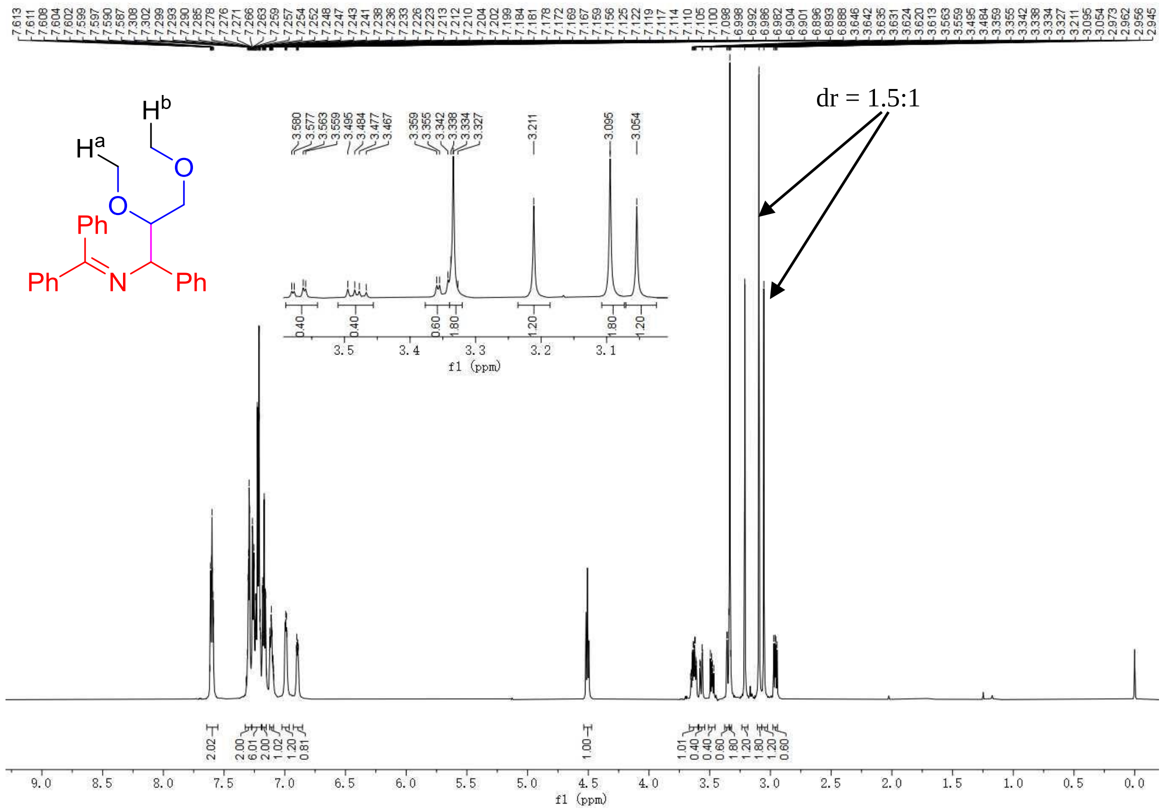

Figure S14. **^{13}C{^{1}H} NMR spectra (150 MHz, Chloroform-*d*) of *N*-(2,3-dimethoxy-1-phenylpropyl)-1,1-diphenylmethanimine (4ad,** dr = 1.5:1**):**

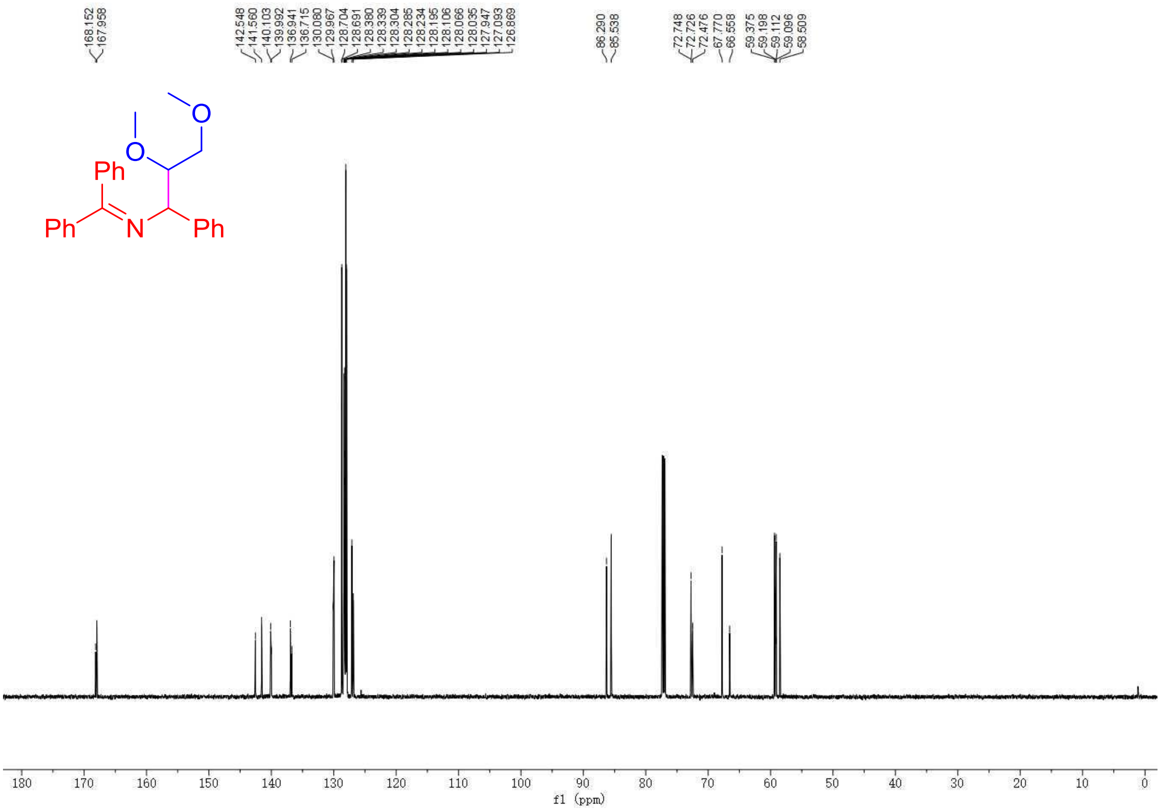

Figure S15. **^{1}H NMR spectra (600 MHz, Chloroform-*d*) of *N*-((3,3-dimethyloxetan-2-yl)(phenyl)methyl)-1,1-diphenylmethanimine (4ae):**

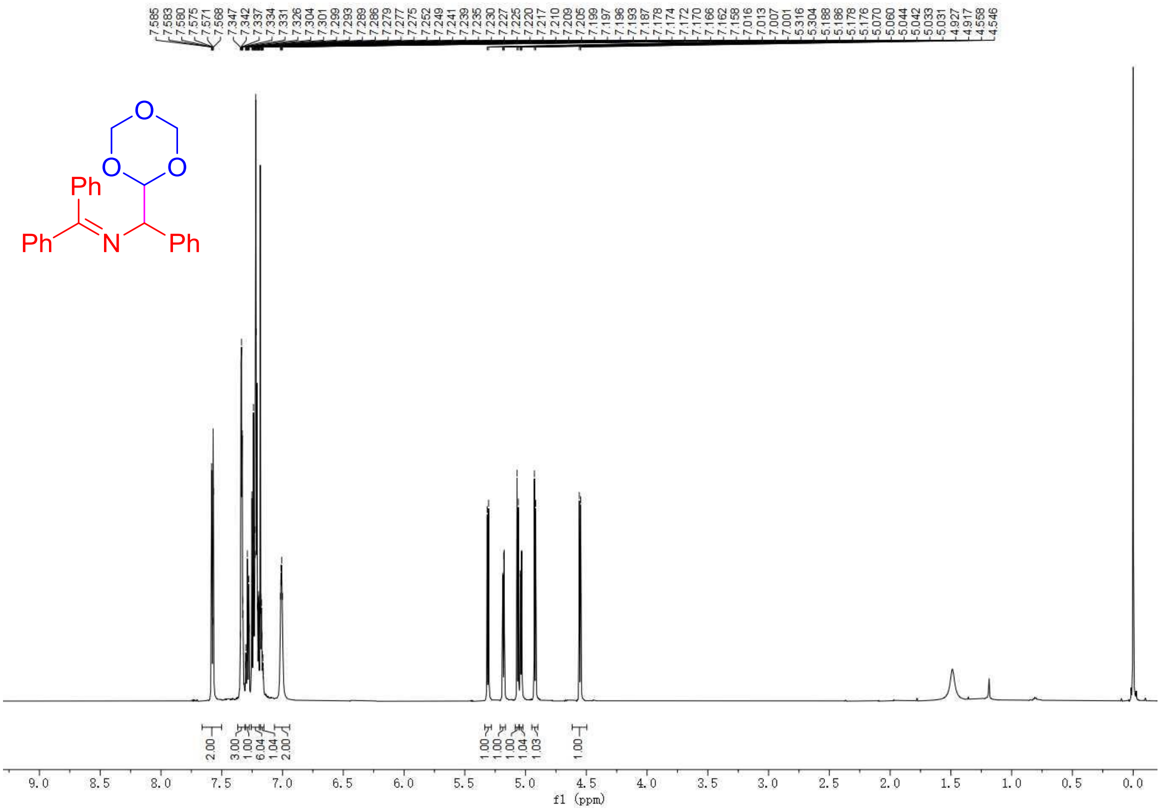

Figure S16. **^{13}C{^{1}H} NMR spectra (150 MHz, Chloroform-*d*) of *N*-((3,3-dimethyloxetan-2-yl)(phenyl)methyl)-1,1-diphenylmethanimine (4ae):**

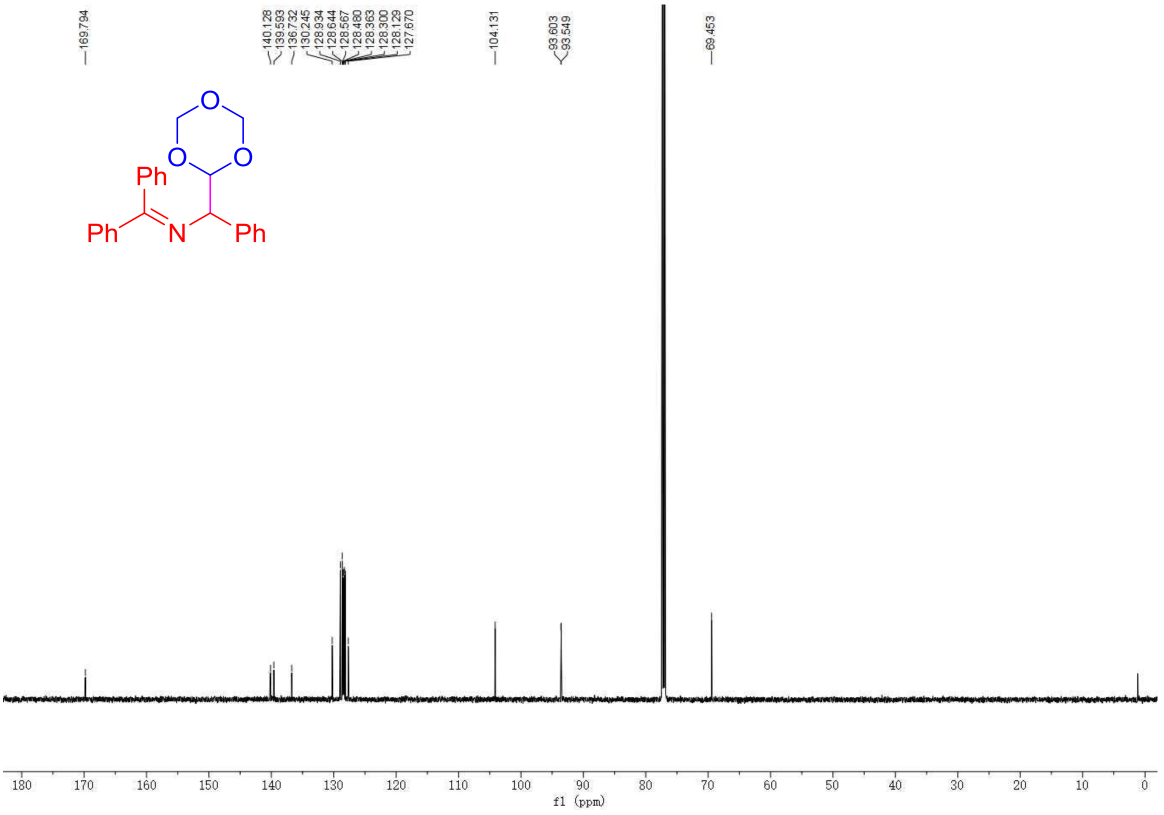

Figure S17. ^{1}H NMR spectra (400 MHz, DMSO-d_6) of *N*-((4-methylmorpholin-3-yl)(phenyl)methyl)-1,1-diphenylmethanimine (4af(major)**):**

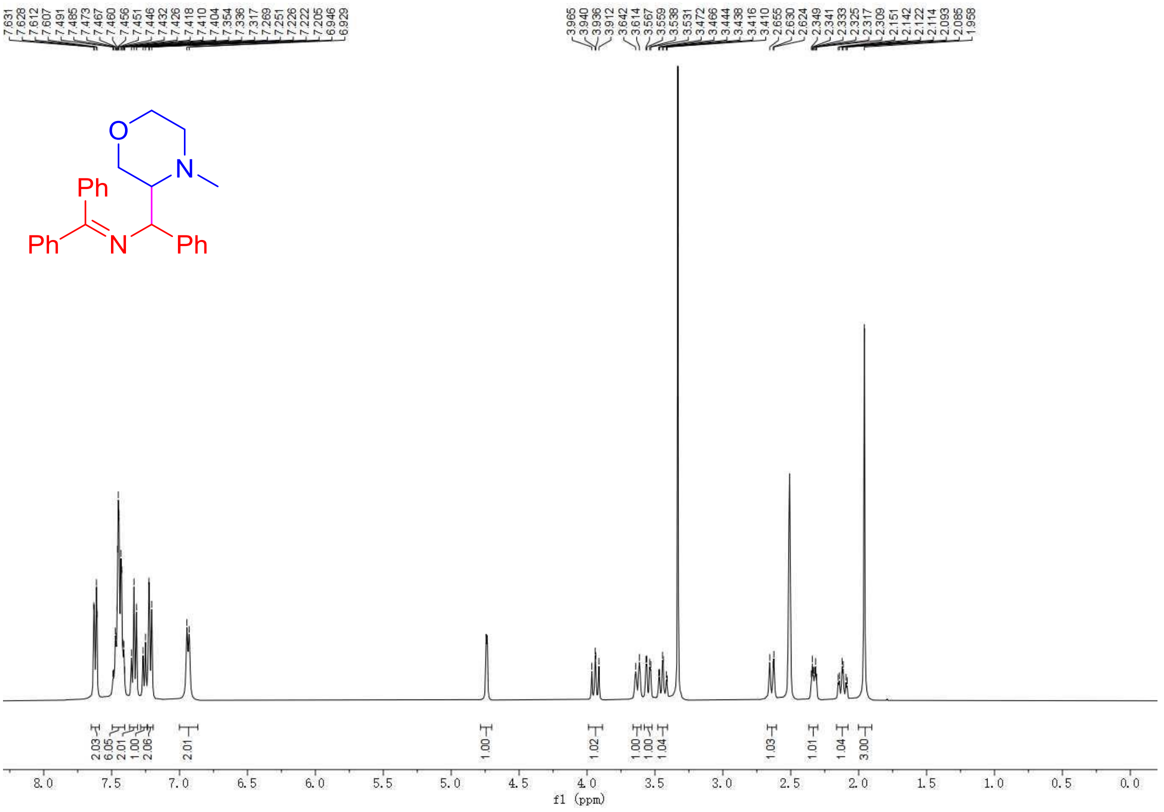

Figure S18. ^{13}C{^{1}H} NMR spectra (100 MHz, DMSO-d_6) of *N*-((4-methylmorpholin-3-yl)(phenyl)methyl)-1,1-diphenylmethanimine (4af(major)**):**

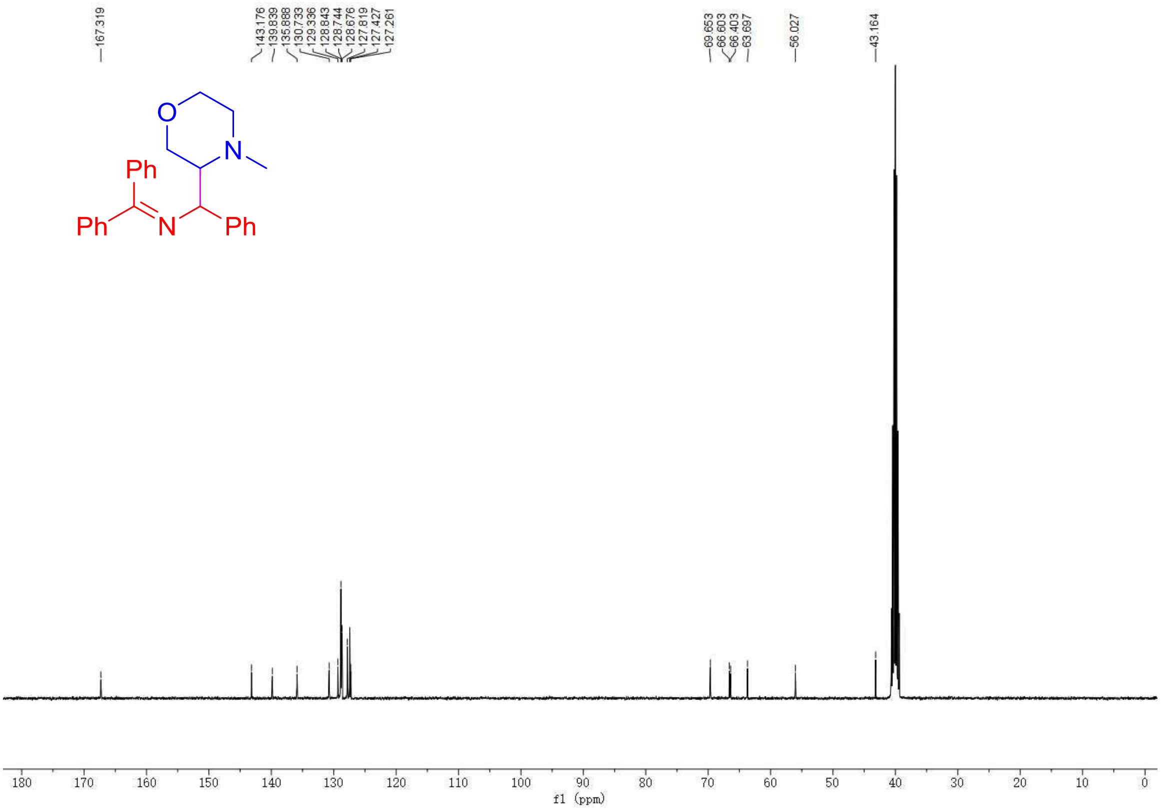

Figure S19. ¹H NMR spectra (400 MHz, Chloroform-*d*) of *N*-((4-methylmorpholin-3-yl)(phenyl)methyl)-1,1-diphenylmethanimine (4af(minor)**):**

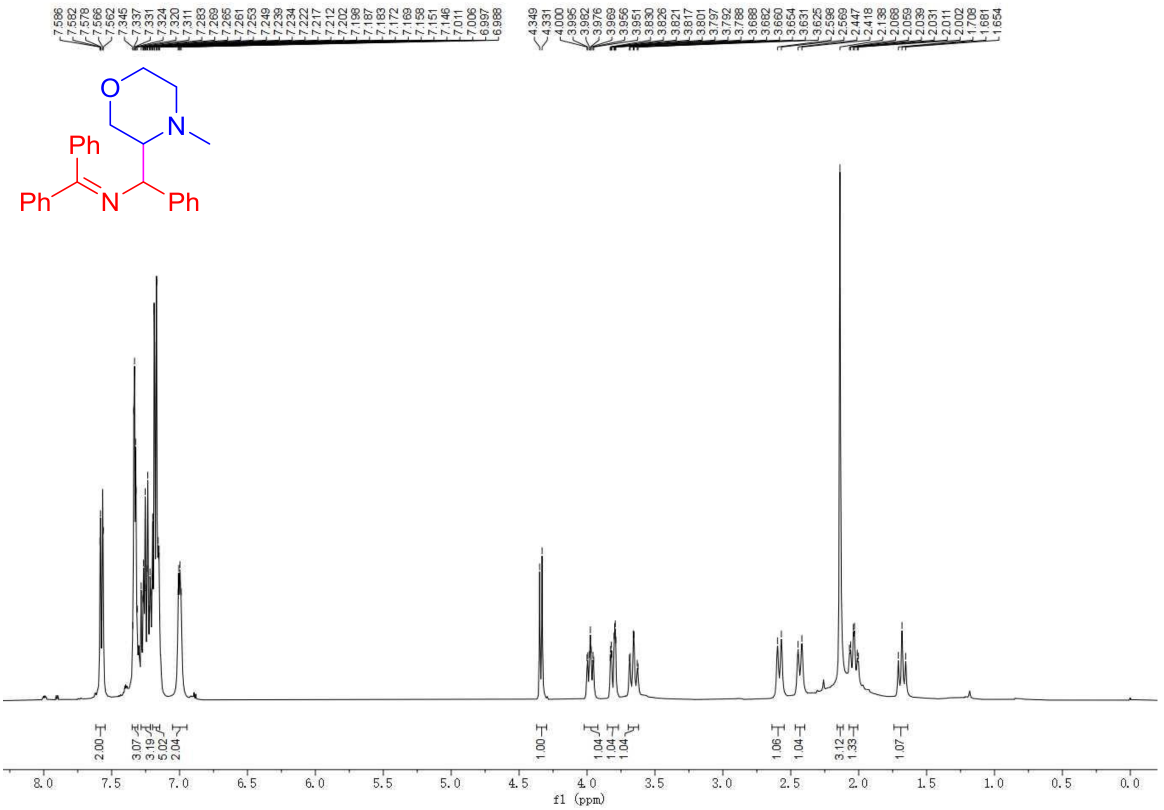

Figure S20. ¹³C{¹H} NMR spectra (100 MHz, Chloroform-*d*) of *N*-((4-methylmorpholin-3-yl)(phenyl)methyl)-1,1-diphenylmethanimine (4af(minor)**):**

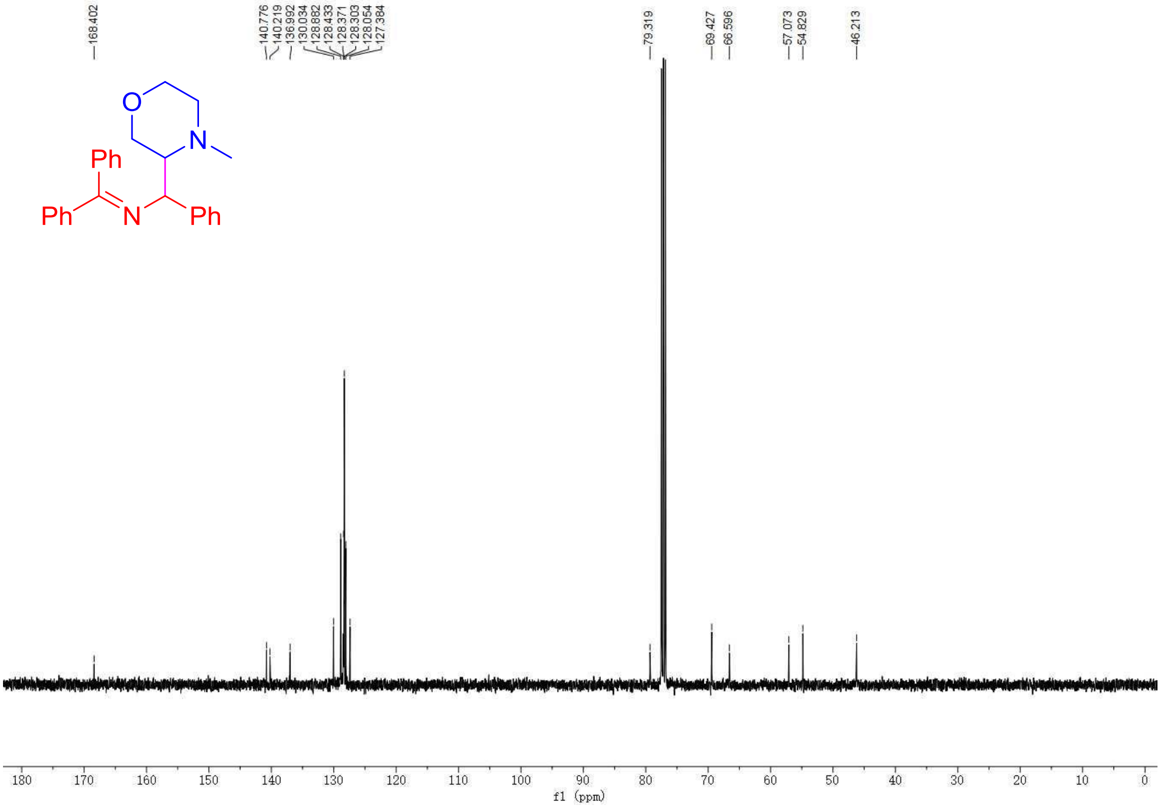

Figure S21. ^{1}H NMR spectra (400 MHz, Methanol-d_4) of *N*-((1-methylpyrrolidin-2-yl)(phenyl)methyl)-1,1-diphenylmethanimine (4ag(major)**):**

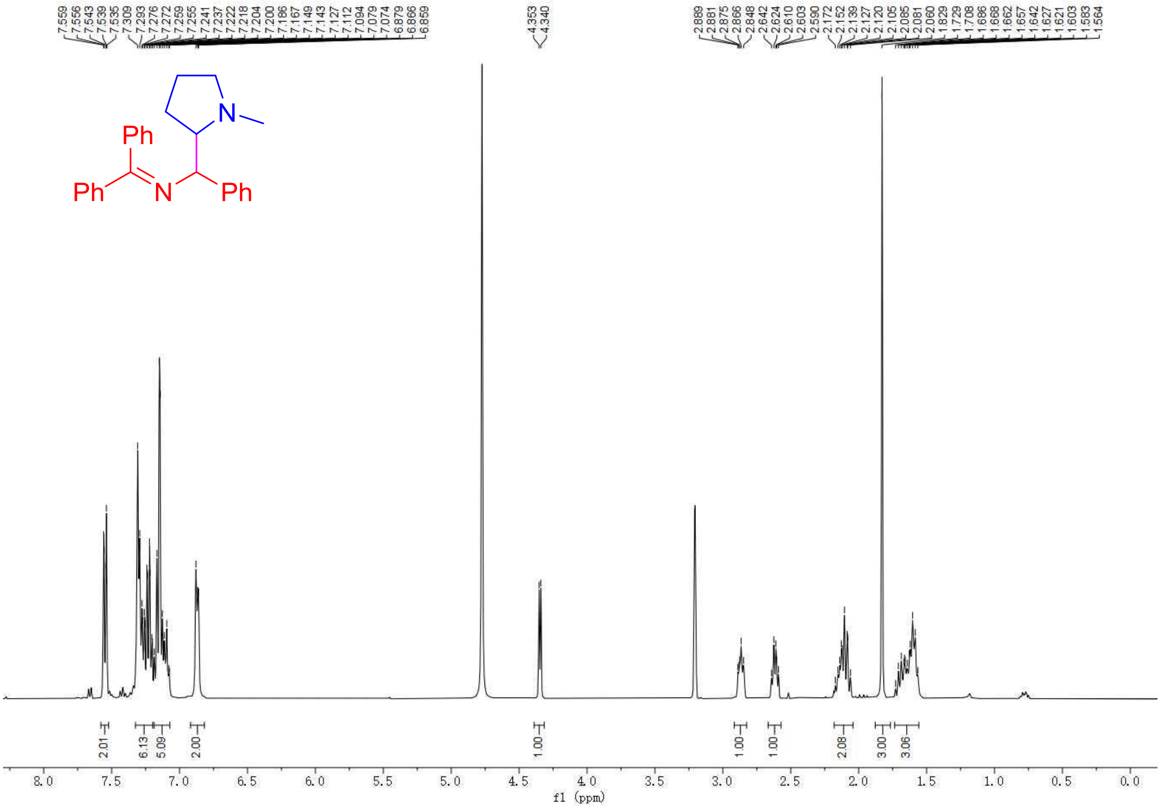

Figure S22. $^{13}C\{^1H\}$ NMR spectra (100 MHz, Methanol-d_4) of *N*-((1-methylpyrrolidin-2-yl)(phenyl)methyl)-1,1-diphenylmethanimine (4ag(major)**):**

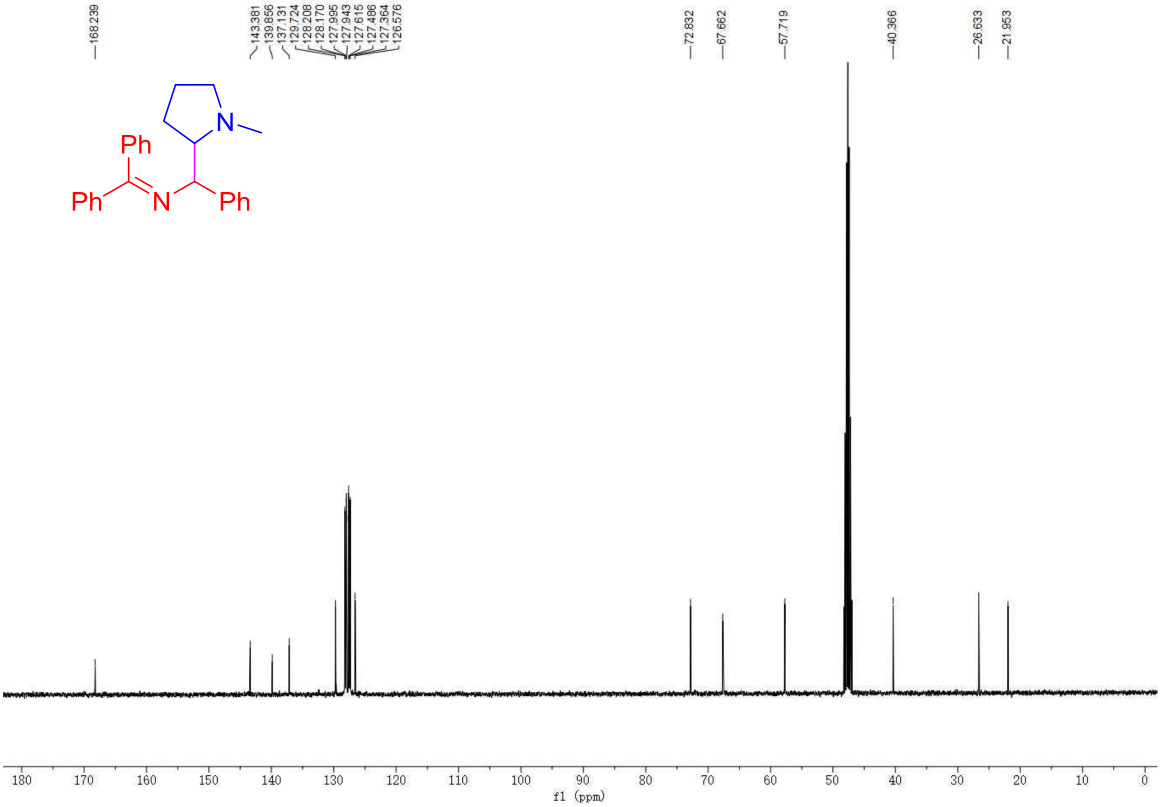

Figure S23. ^{1}**H NMR spectra (400 MHz, Methanol-*d*$_4$) of *N*-((1-methylpyrrolidin-2-yl)(phenyl)methyl)-1,1-diphenylmethanimine (4ag**(minor)**):**

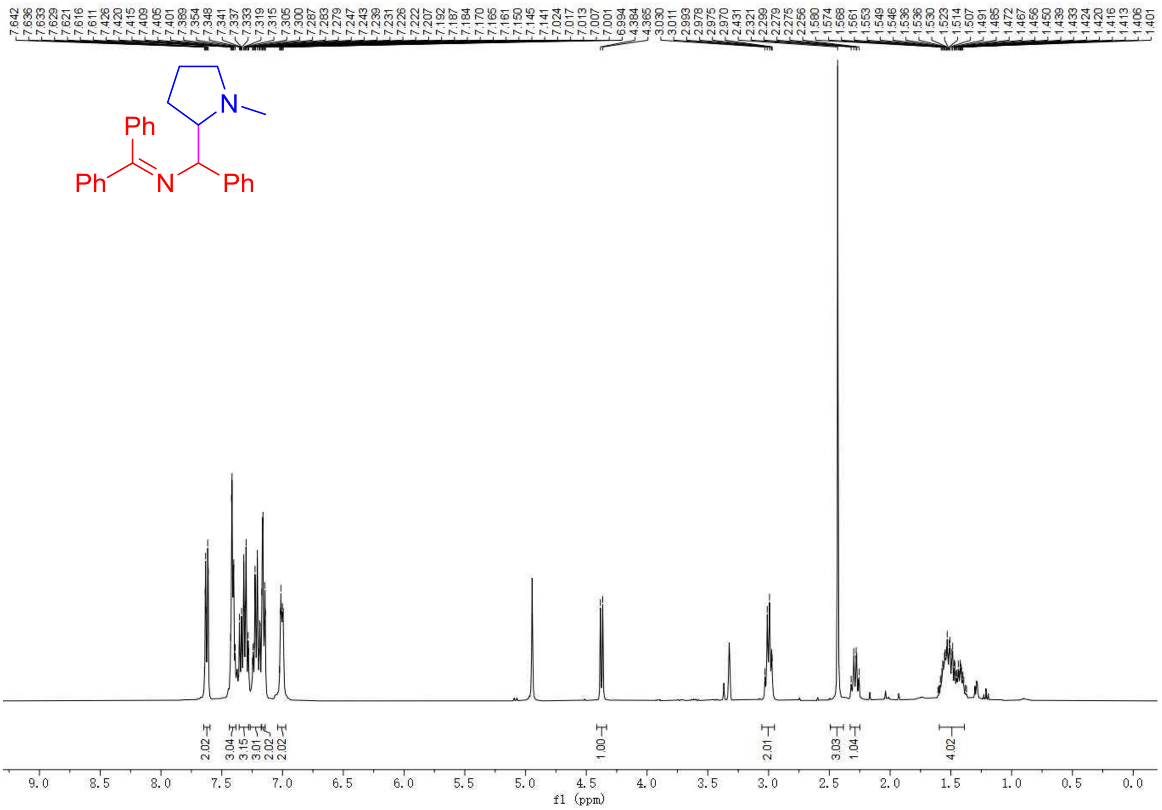

Figure S24. ^{13}**C{**^{1}**H} NMR spectra (100 MHz, Methanol-*d*$_4$) of *N*-((1-methylpyrrolidin-2-yl)(phenyl)methyl)-1,1-diphenylmethanimine (4ag**(minor)**):**

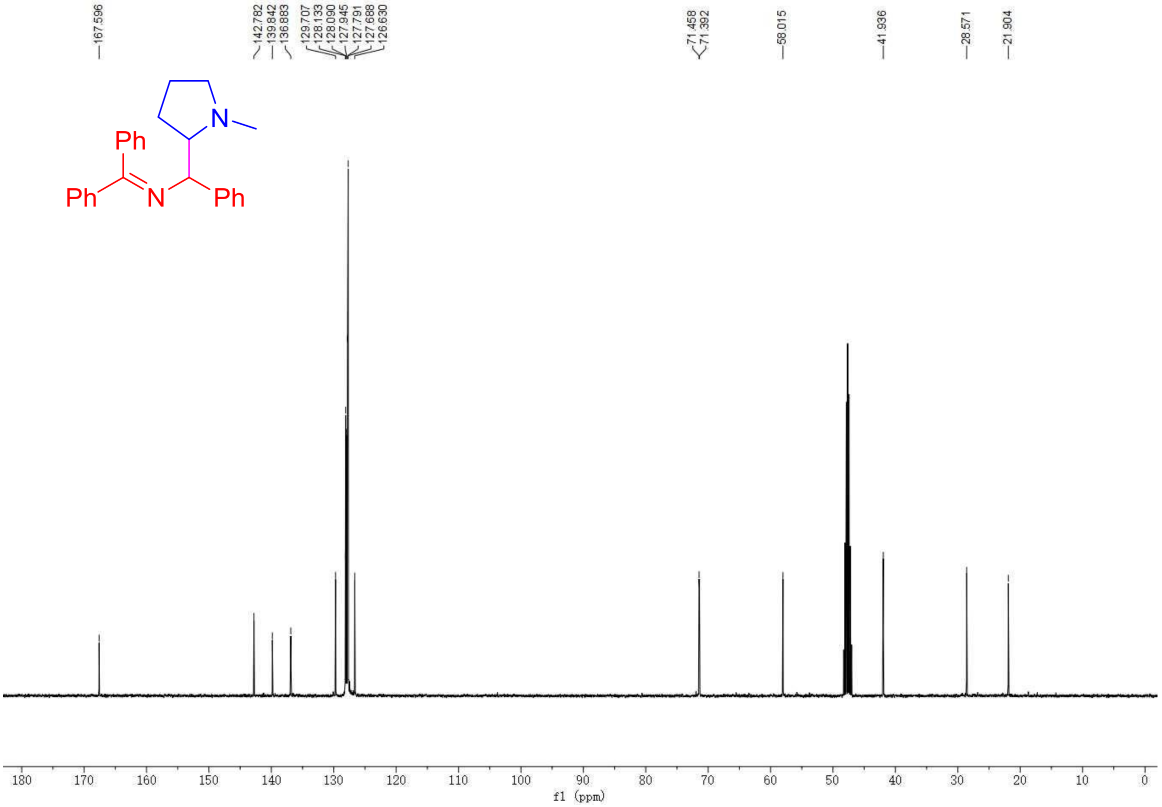

Figure S25. ^{1}H NMR spectra (400 MHz, Chloroform-*d*) of 1,1-diphenyl-*N*-(phenyl(1-phenylpyrrolidin-2-yl)methyl)methanimine (4ah(major)**):**

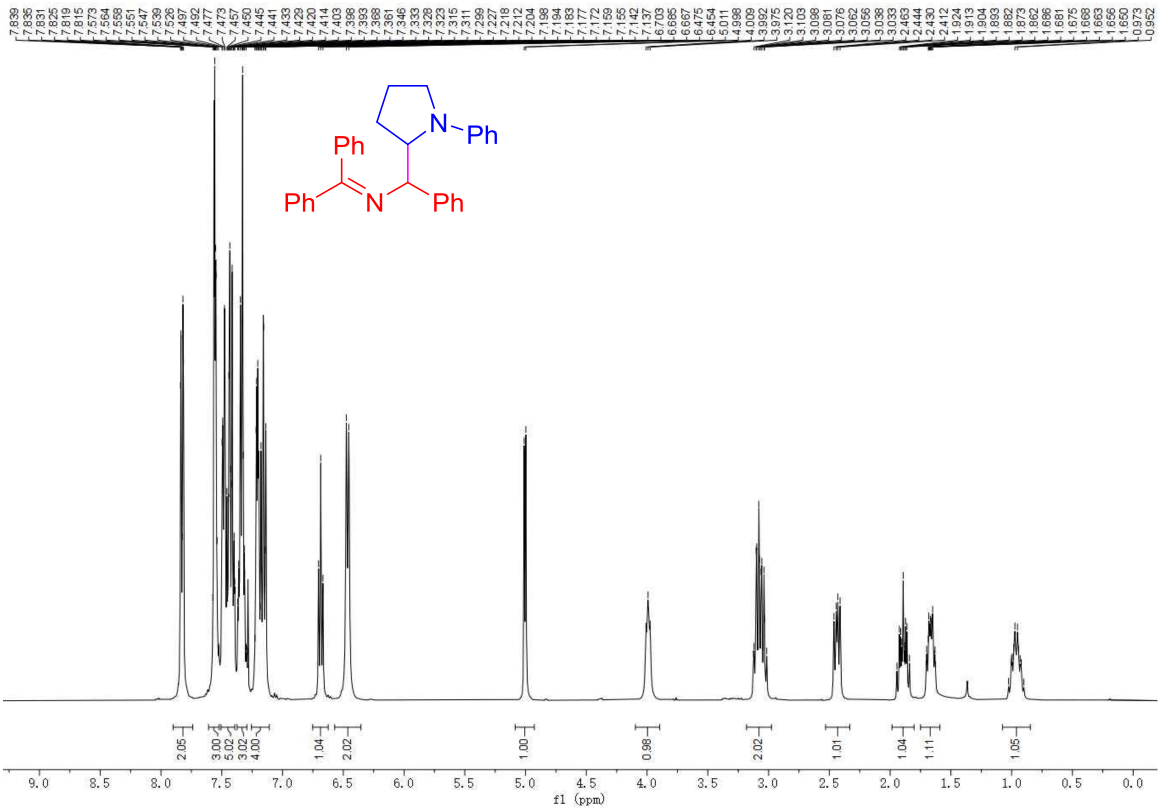

Figure S26. $^{13}C\{^{1}H\}$ NMR spectra (400 MHz, Chloroform-*d*) of 1,1-diphenyl-*N*-(phenyl(1-phenylpyrrolidin-2-yl)methyl)methanimine (4ah(major)**):**

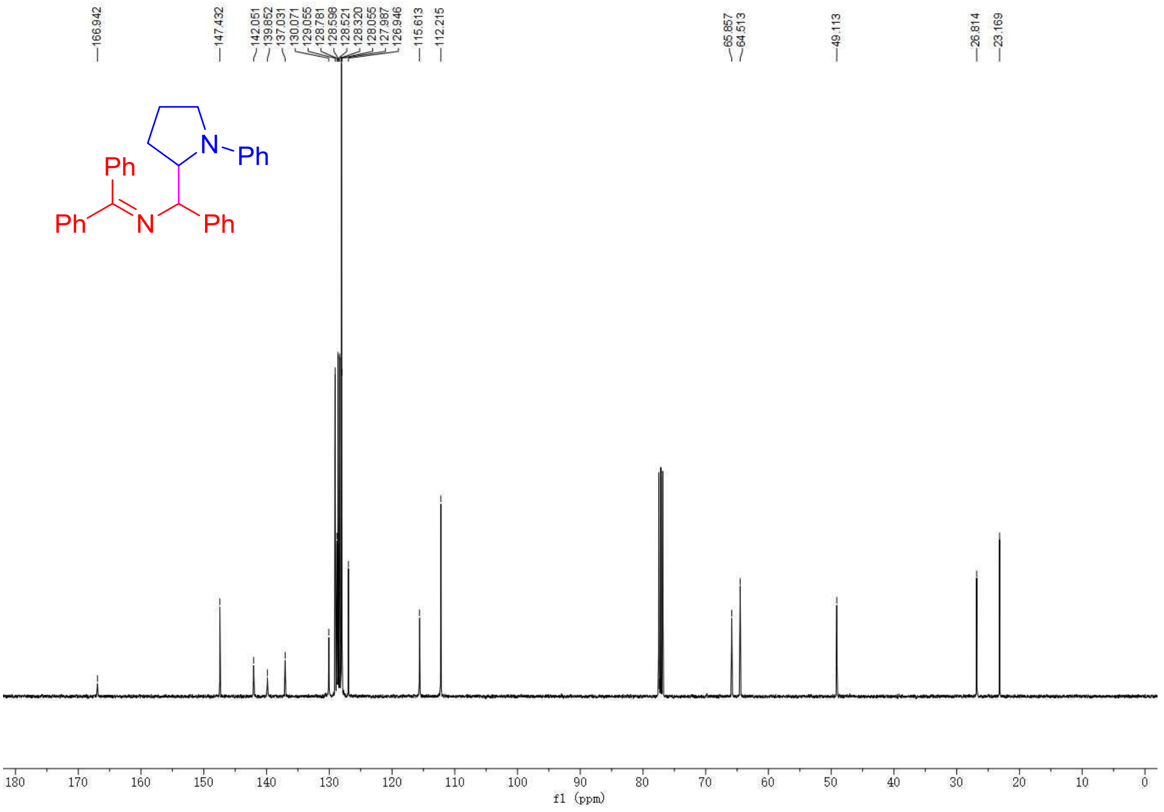

Figure S27. ^{1}H NMR spectra (400 MHz, Chloroform-*d*) of 1,1-diphenyl-*N*-(phenyl(1-phenylpyrrolidin-2-yl)methyl)methanimine (4ah(minor)**):**

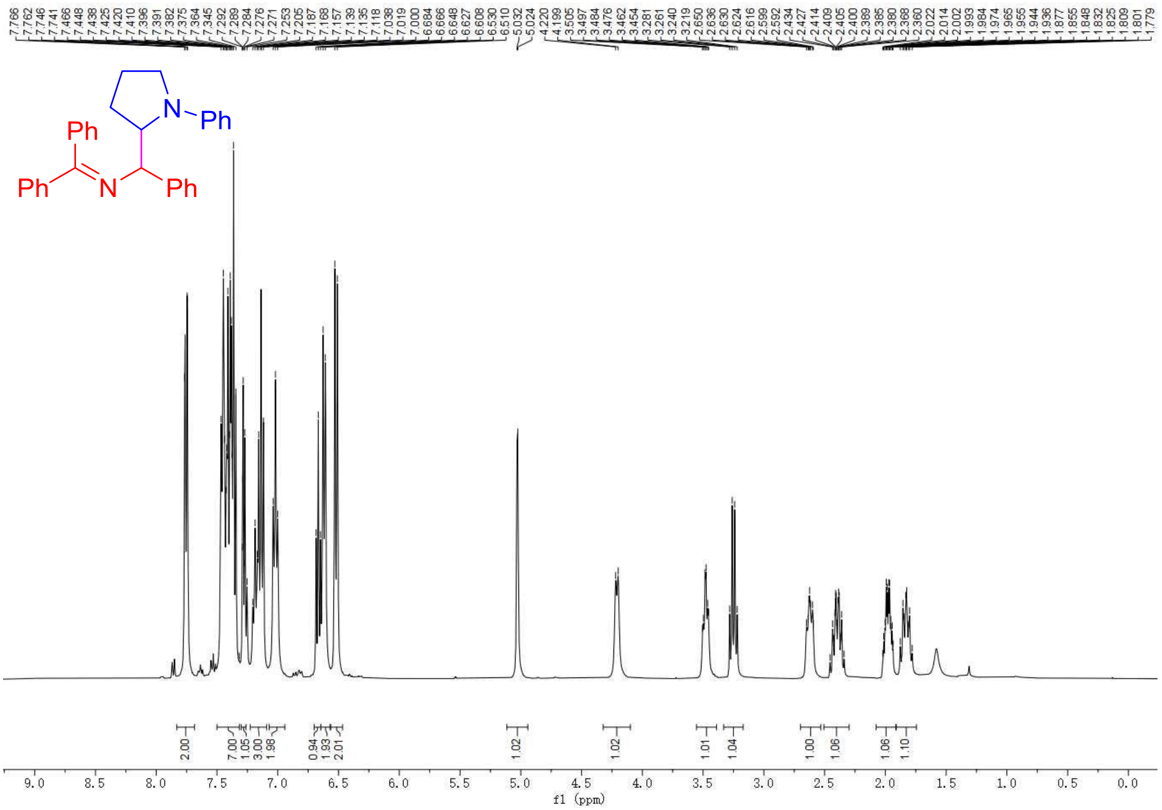

Figure S28. $^{13}C\{^{1}H\}$ NMR spectra (400 MHz, Chloroform-*d*) of 1,1-diphenyl-*N*-(phenyl(1-phenylpyrrolidin-2-yl)methyl)methanimine (4ah(minor)**):**

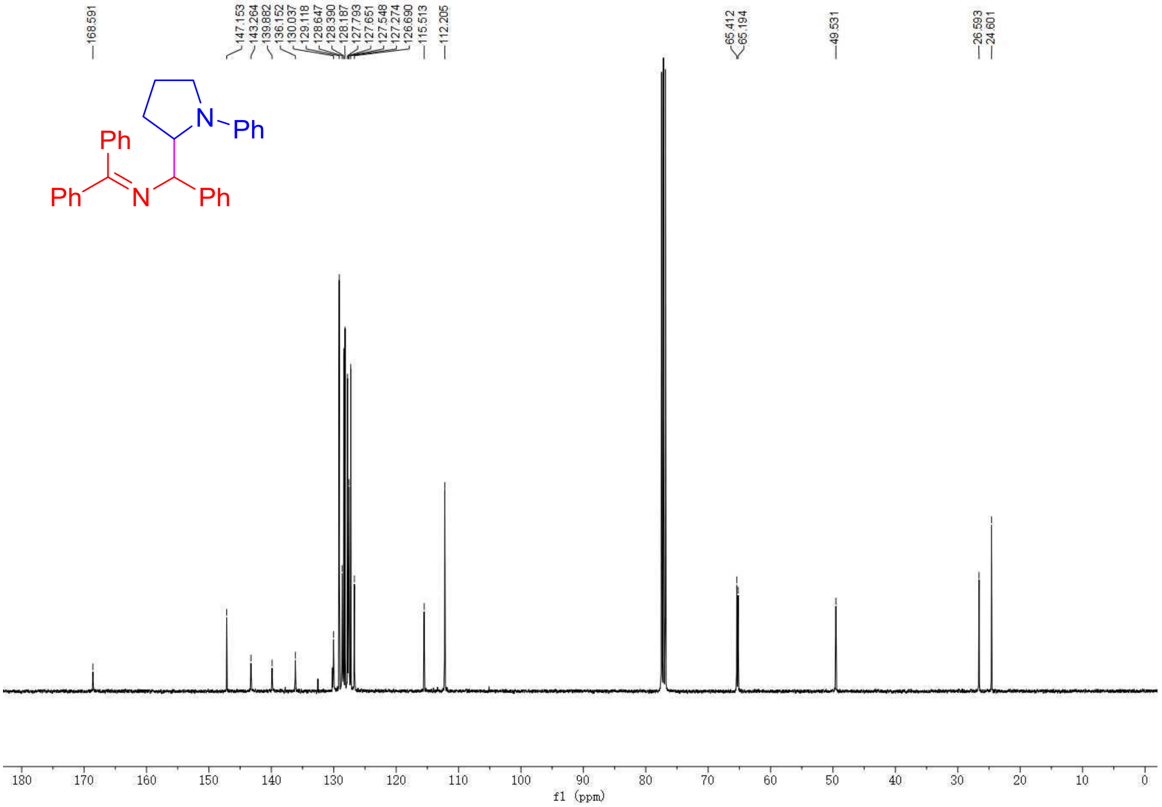

Figure S29. ¹H NMR spectra (400 MHz, Chloroform-*d*) of 1,1-diphenyl-*N*-(phenyl(1-phenylpiperidin-2-yl)methyl)methanimine (4ai, dr = 1.9:1**):**

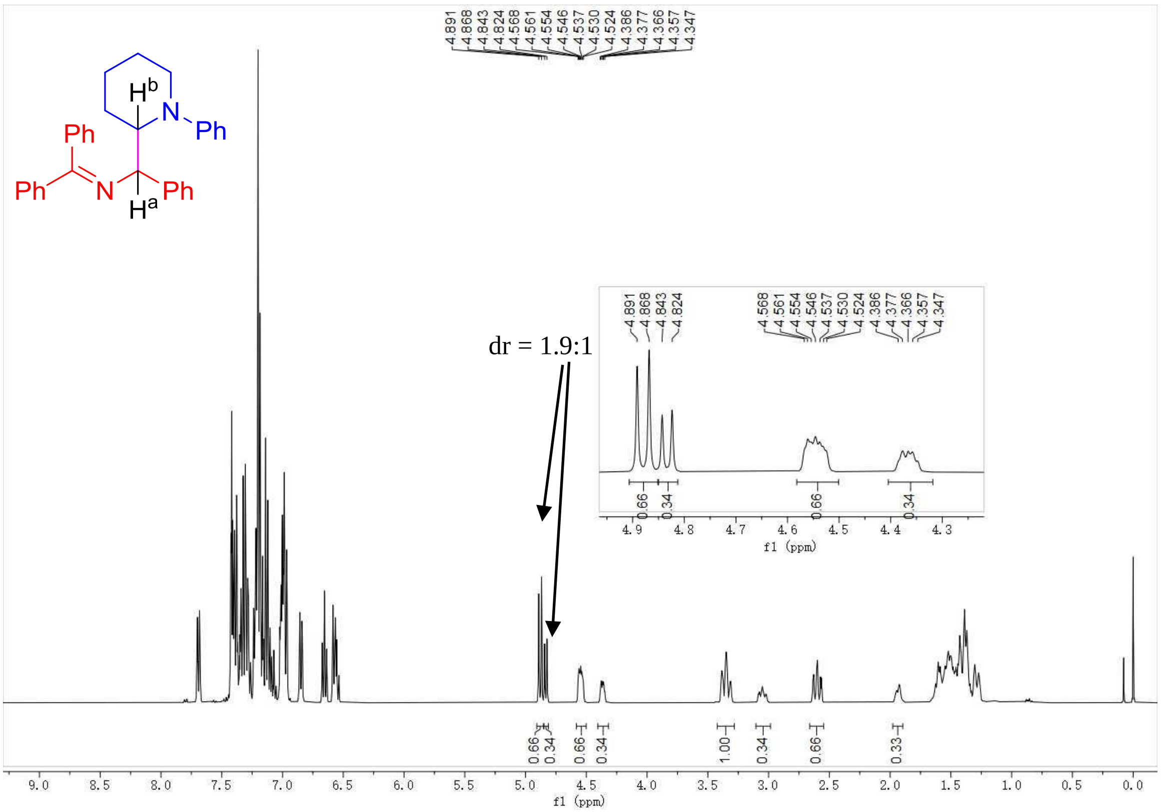

Figure S30. ¹³C{¹H} NMR spectra (100 MHz, Chloroform-*d*) of 1,1-diphenyl-*N*-(phenyl(1-phenylpiperidin-2-yl)methyl)methanimine (4ai, dr = 1.9:1**):**

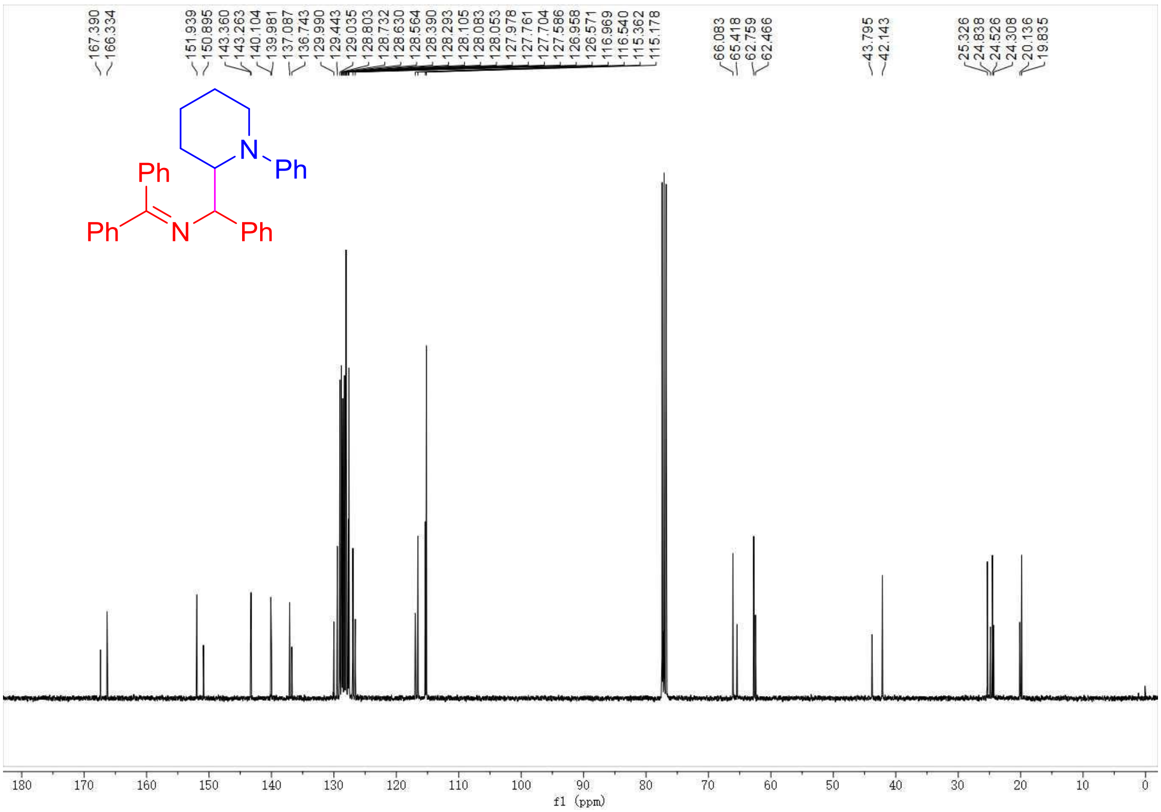

Figure S31. ^{1}H **NMR spectra (600 MHz, Chloroform-*d*) of 1,1-diphenyl-*N*-(1-phenyl-2-(1*H*-pyrrol-1-yl)ethyl)methanimine (4aj):**

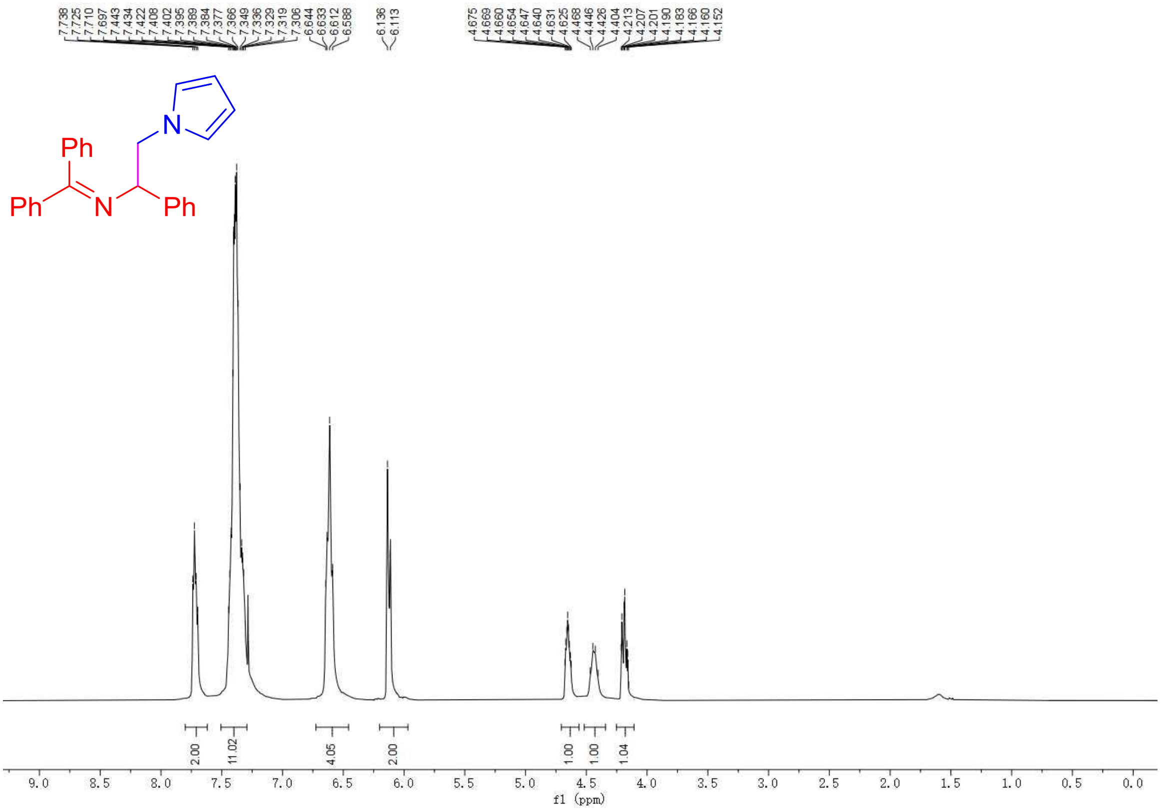

Figure S32. $^{13}C\{^{1}H\}$ **NMR spectra (150 MHz, Chloroform-*d*) of 1,1-diphenyl-*N*-(1-phenyl-2-(1*H*-pyrrol-1-yl)ethyl)methanimine (4aj):**

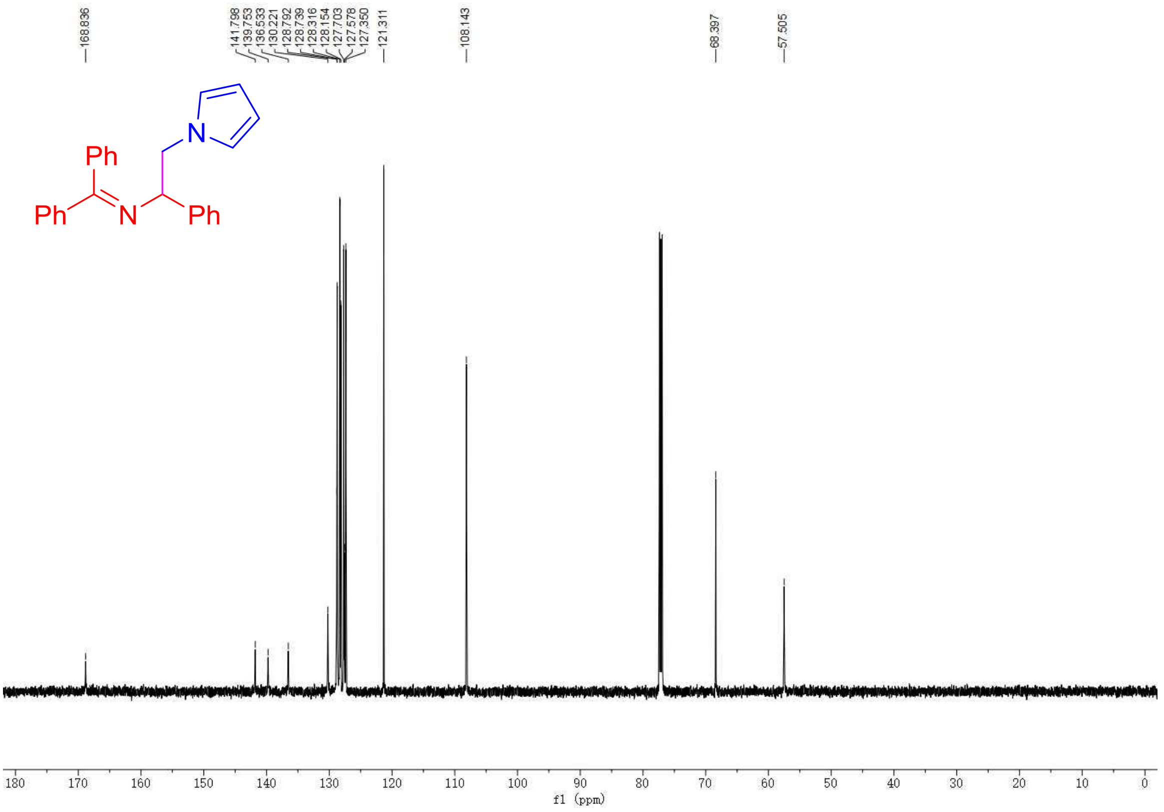

Figure S33. ^{1}H NMR spectra (400 MHz, Chloroform-*d*) of *N*-(2-((diphenylmethylene)amino)-2-phenylethyl)-*N*-methylaniline (4ak):

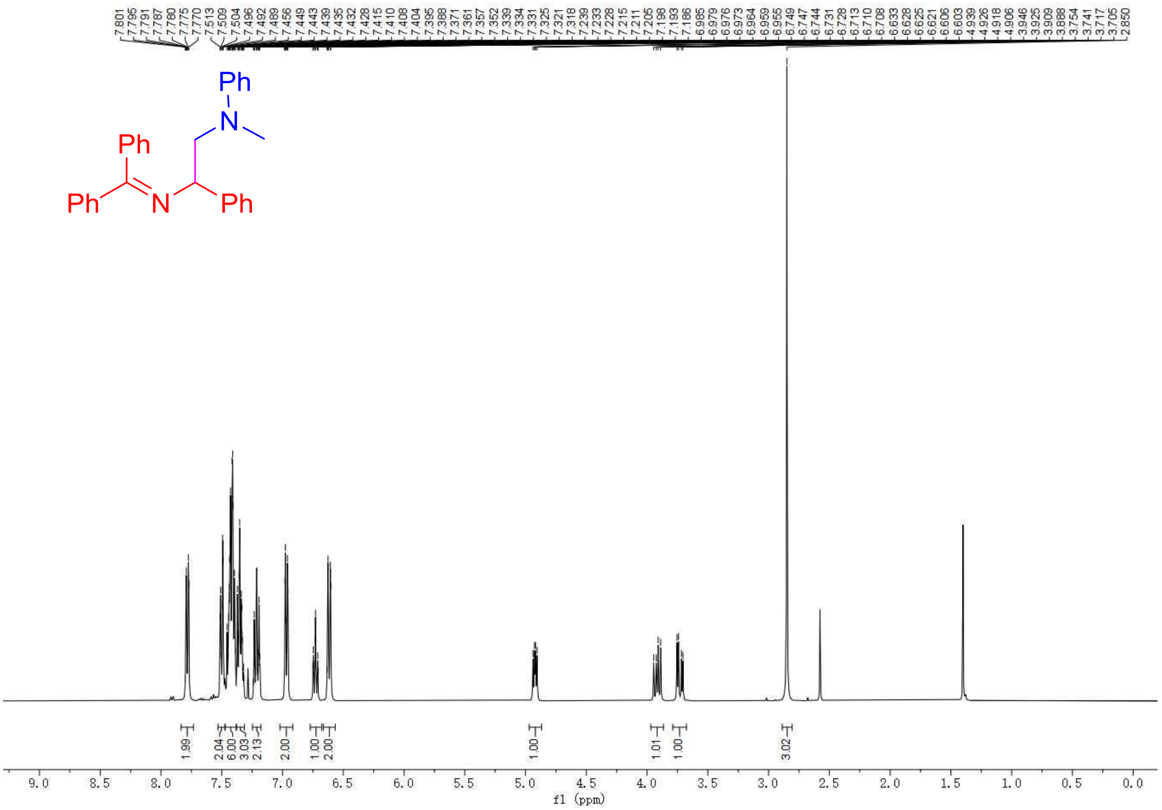

Figure S34. ^{13}C{^{1}H} NMR spectra (100 MHz, Chloroform-*d*) of *N*-(2-((diphenylmethylene)amino)-2-phenylethyl)-*N*-methylaniline (4ak):

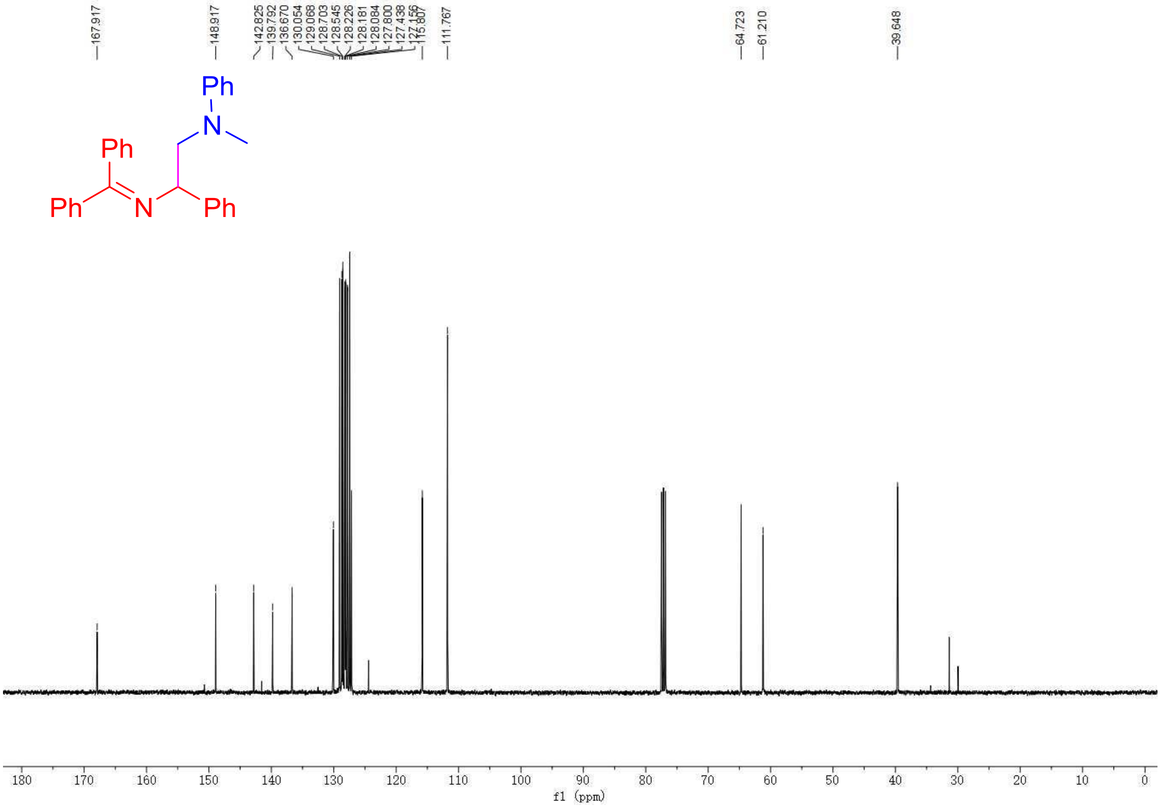

Figure S35. ^{1}H NMR spectra (600 MHz, Chloroform-*d*) of *N*-((4-(*tert*-butyl)phenyl)(tetrahydrofuran-2-yl)methyl)-1,1-diphenylmethanimine (4ba(major)):

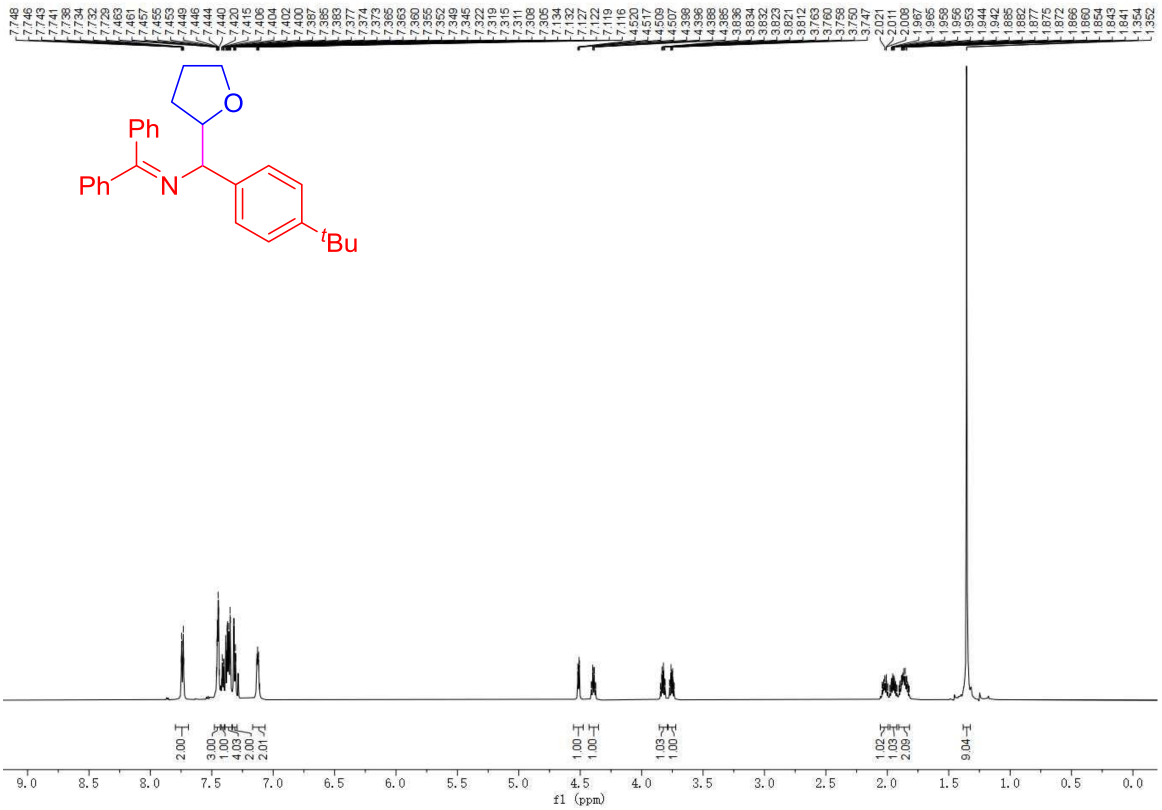

Figure S36. ^{13}C{^{1}H} NMR spectra (150 MHz, Chloroform-*d*) of *N*-((4-(*tert*-butyl)phenyl)(tetrahydrofuran-2-yl)methyl)-1,1-diphenylmethanimine (4ba(major)):

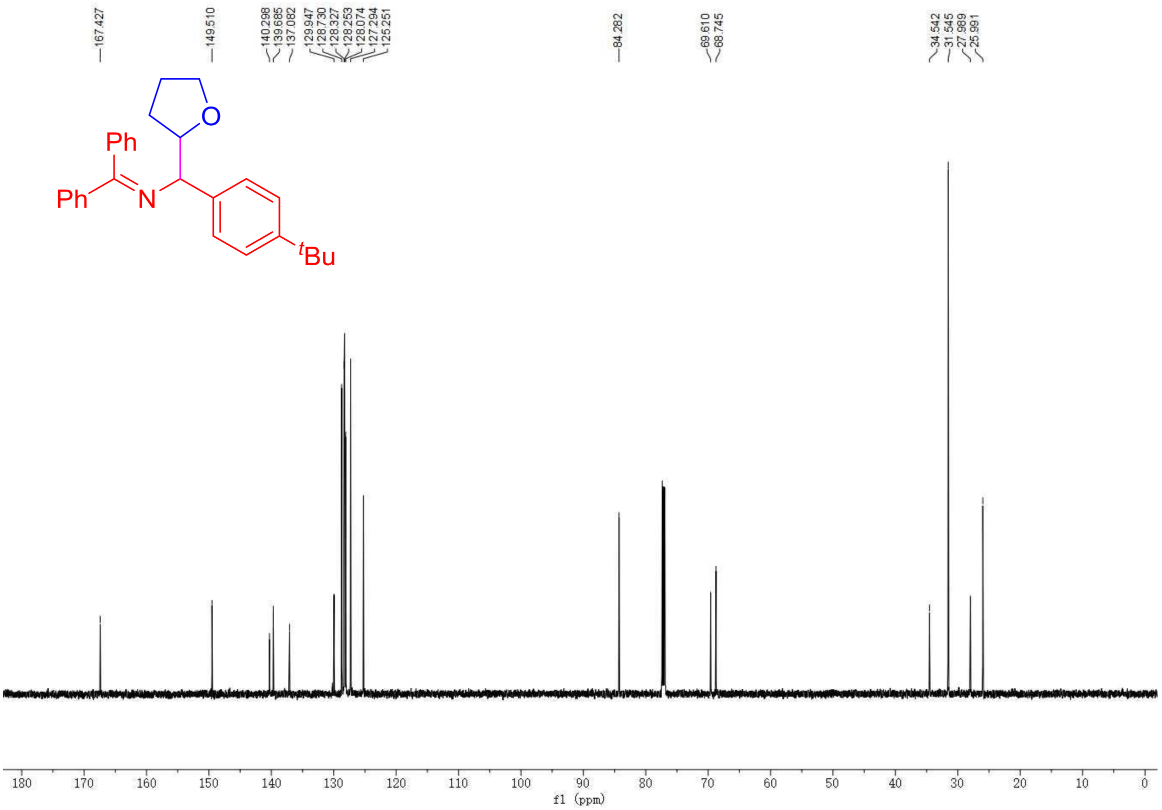

Figure S37. **^{1}H NMR spectra (400 MHz, Chloroform-*d*) of *N*-((4-(*tert*-butyl)phenyl)(tetrahydrofuran-2-yl)methyl)-1,1-diphenylmethanimine (4ba**(minor)**):**

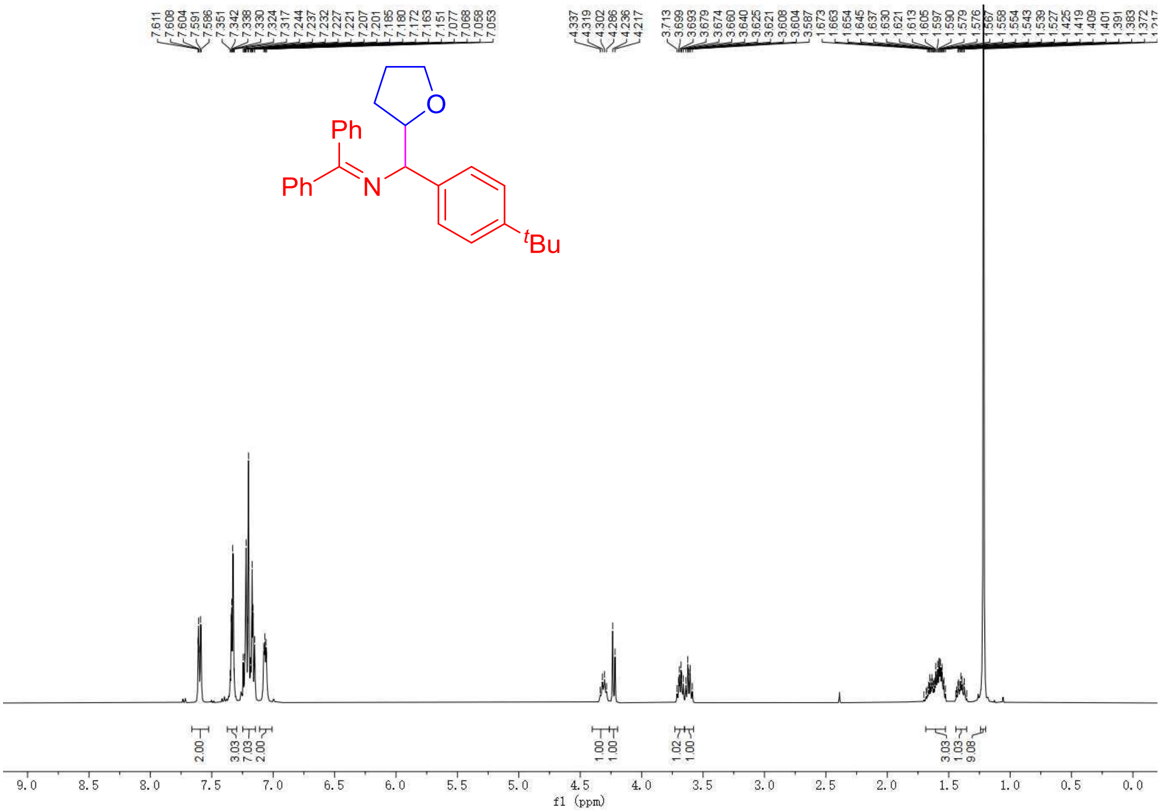

Figure S38. **^{13}C{^{1}H} NMR spectra (100 MHz, Chloroform-*d*) of *N*-((4-(*tert*-butyl)phenyl)(tetrahydrofuran-2-yl)methyl)-1,1-diphenylmethanimine (4ba**(minor)**):**

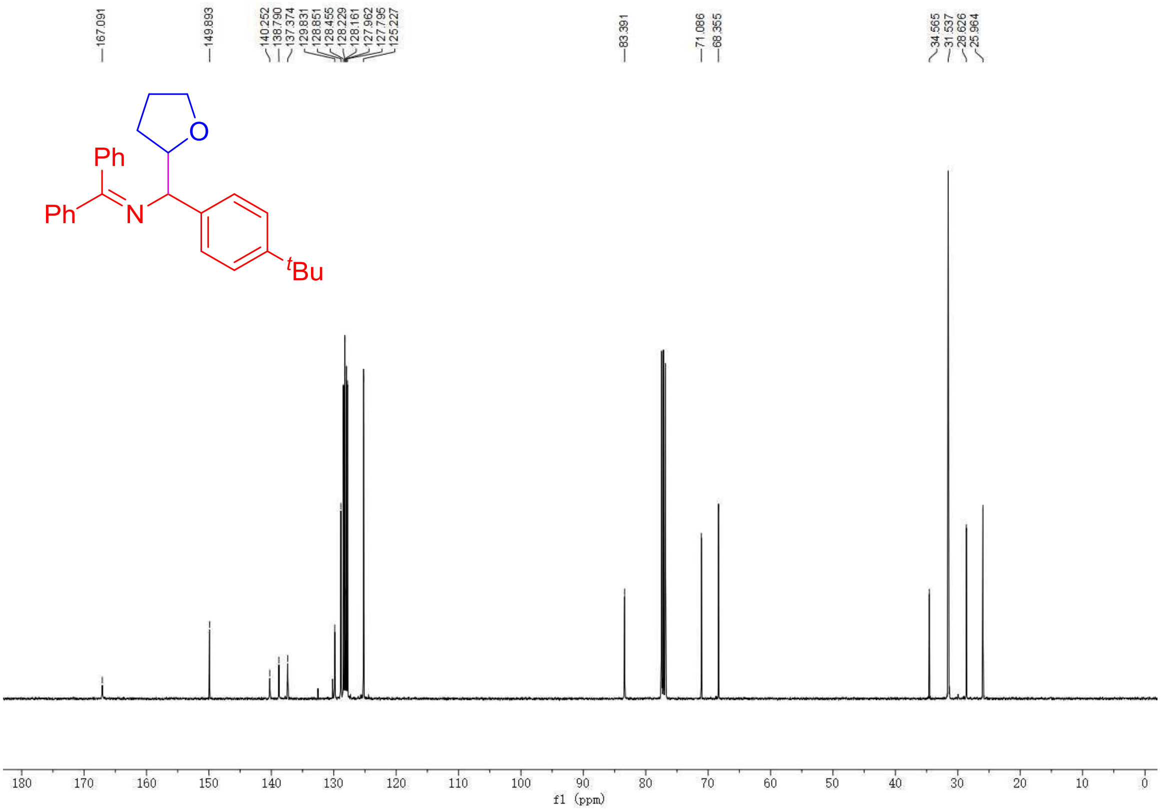

Figure S39. **^{1}H NMR spectra (400 MHz, Chloroform-*d*) of *N*-((4-methoxyphenyl)(tetrahydrofuran-2-yl)methyl)-1,1-diphenylmethanimine (4ca**(major)**):**

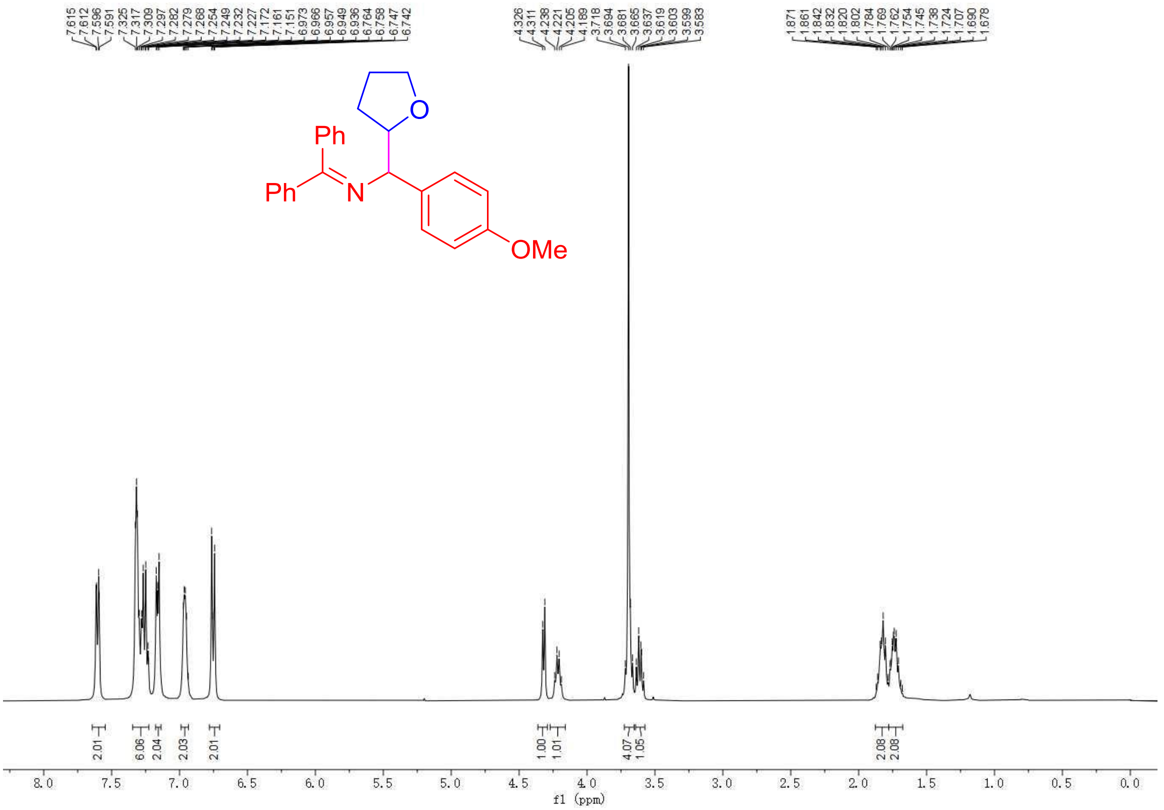

Figure S40. **^{13}C{^{1}H} NMR spectra (100 MHz, Chloroform-*d*) of *N*-((4-methoxyphenyl)(tetrahydrofuran-2-yl)methyl)-1,1-diphenylmethanimine (4ca**(major)**):**

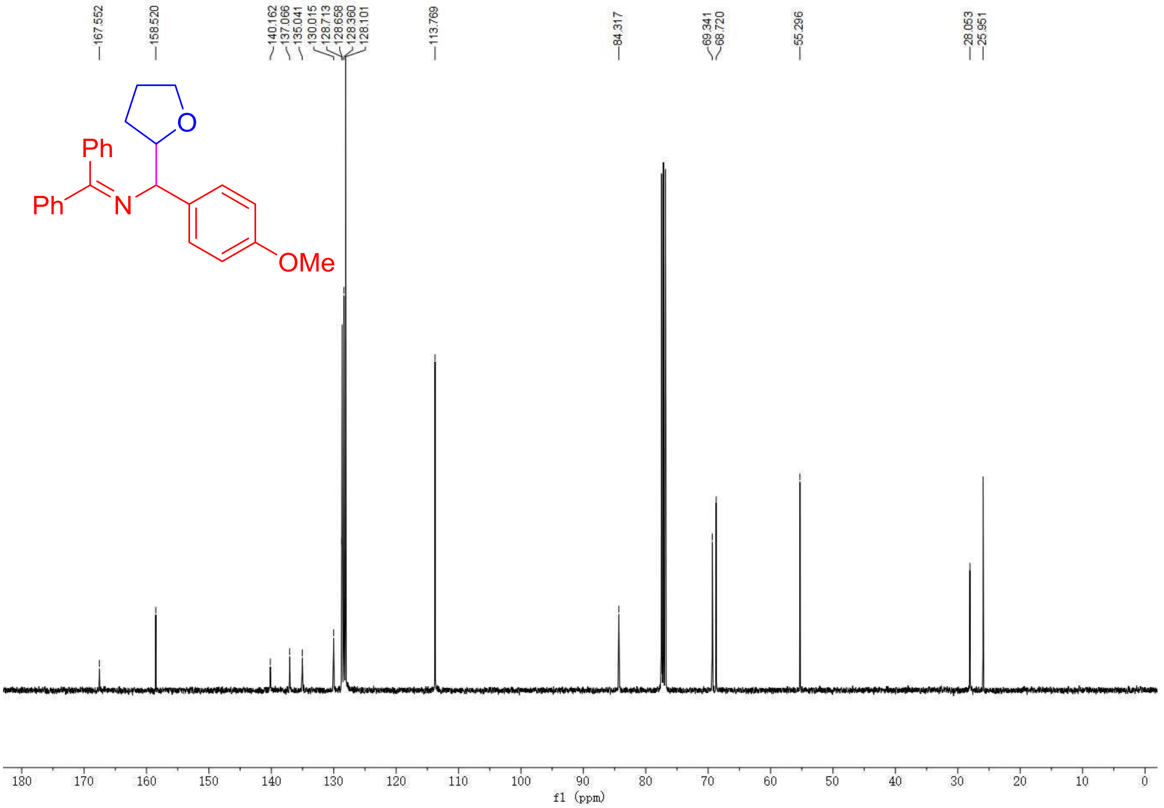

Figure S41. ^{1}**H NMR spectra (400 MHz, Chloroform-*d*) of *N*-((4-methoxyphenyl)(tetrahydrofuran-2-yl)methyl)-1,1-diphenylmethanimine (4ca**(minor)**):**

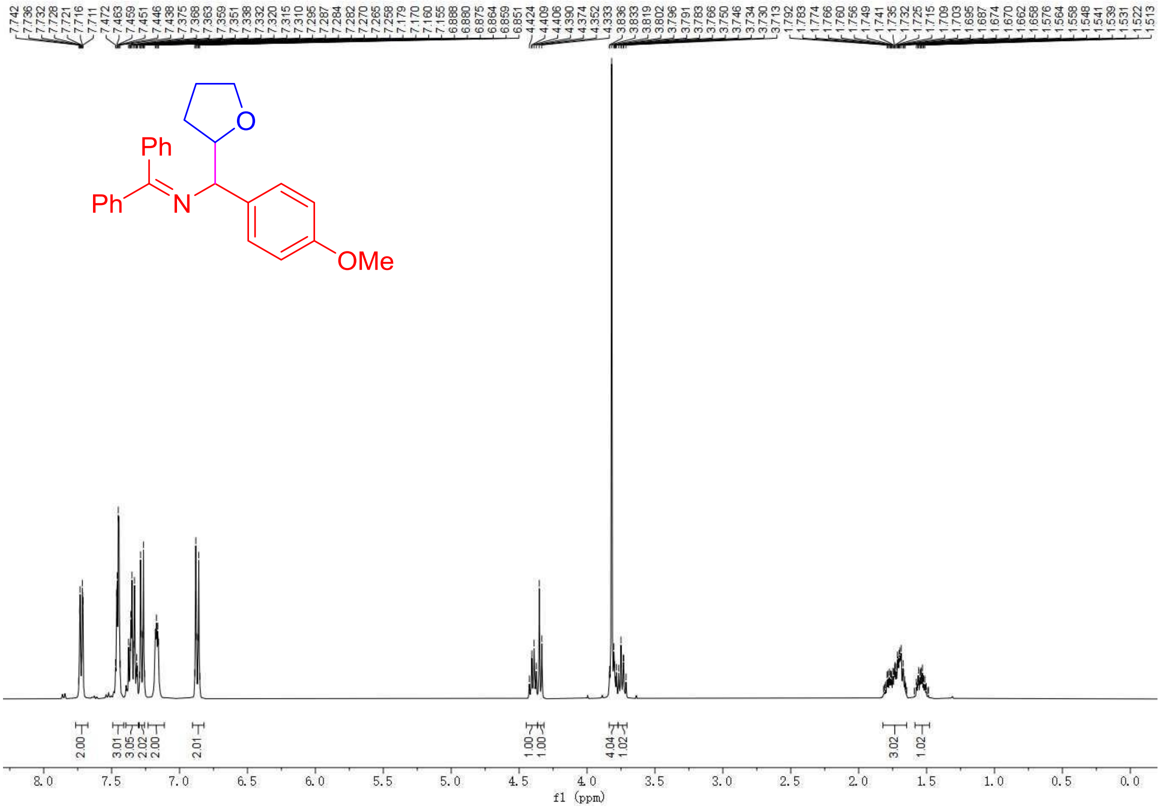

Figure S42. ^{13}**C{**^{1}**H} NMR spectra (100 MHz, Chloroform-*d*) of *N*-((4-methoxyphenyl)(tetrahydrofuran-2-yl)methyl)-1,1-diphenylmethanimine (4ca**(minor)**):**

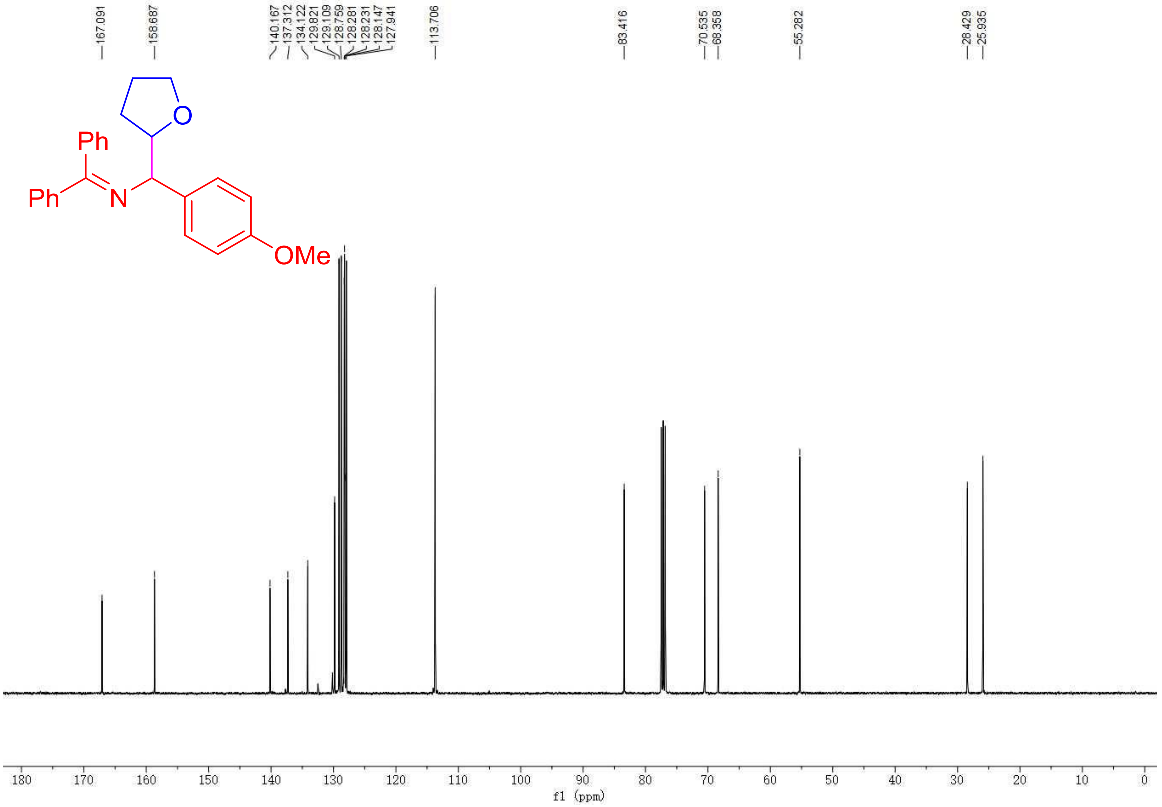

Figure S43. **^{1}H NMR spectra (400 MHz, Chloroform-*d*) of *N*-(benzo[*d*][1,3]dioxol-5-yl(tetrahydrofuran-2-yl)methyl)-1,1-diphenylmethanimine (4da**(major)**):**

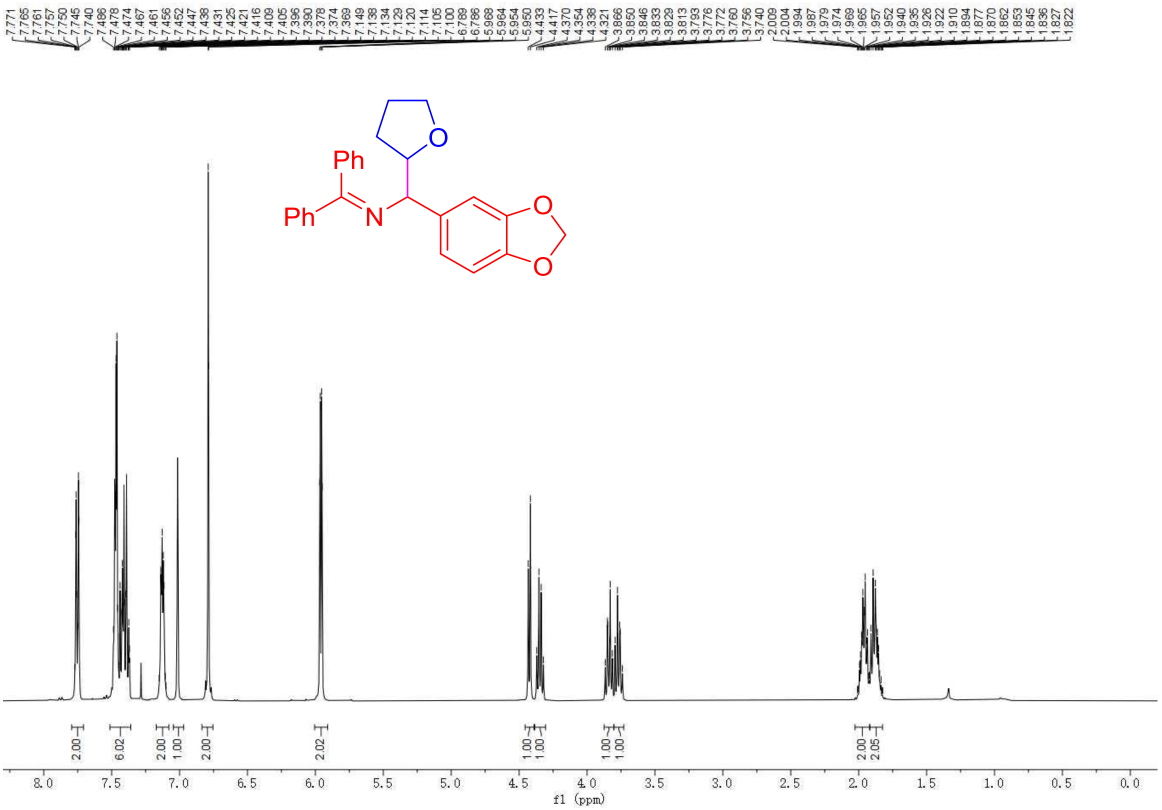

Figure S44. **^{13}C{^{1}H} NMR spectra (100 MHz, Chloroform-*d*) of *N*-(benzo[*d*][1,3]dioxol-5-yl(tetrahydrofuran-2-yl)methyl)-1,1-diphenylmethanimine (4da**(major)**):**

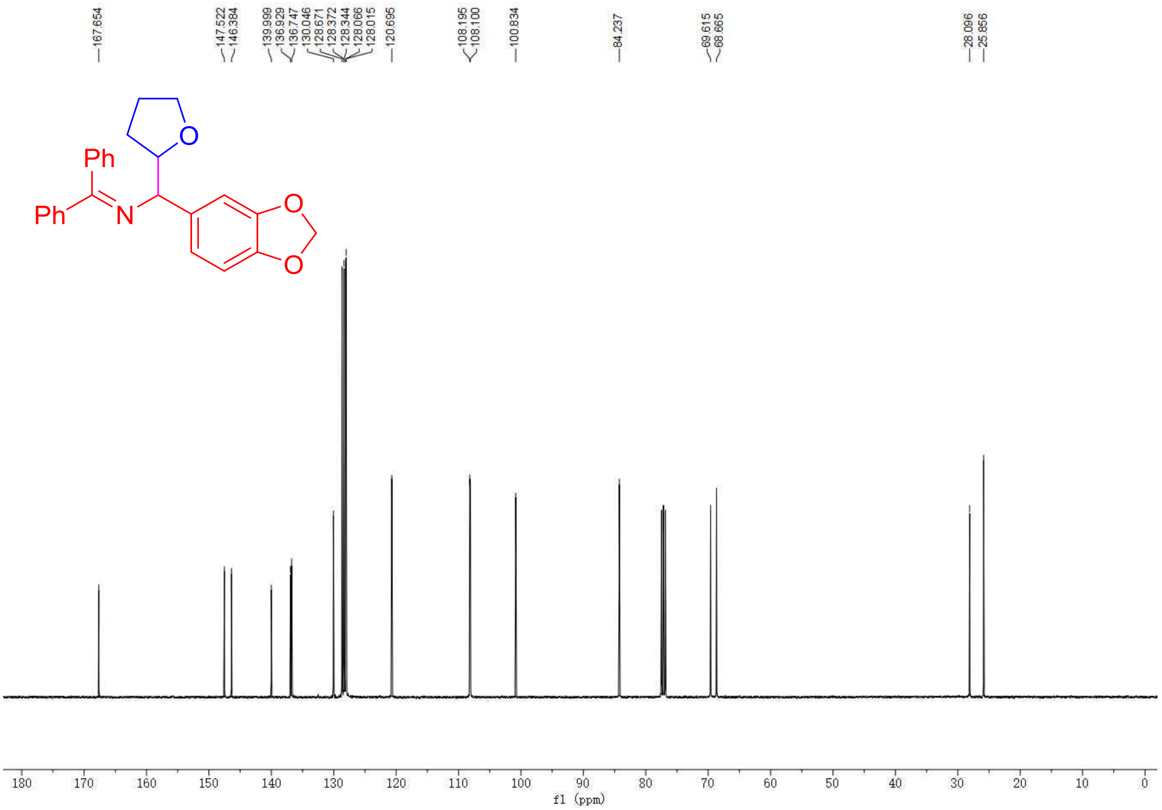

Figure S45. **^{1}H NMR spectra (400 MHz, Chloroform-*d*) of *N*-(benzo[*d*][1,3]dioxol-5-yl(tetrahydrofuran-2-yl)methyl)-1,1-diphenylmethanimine (4da**(minor)**):**

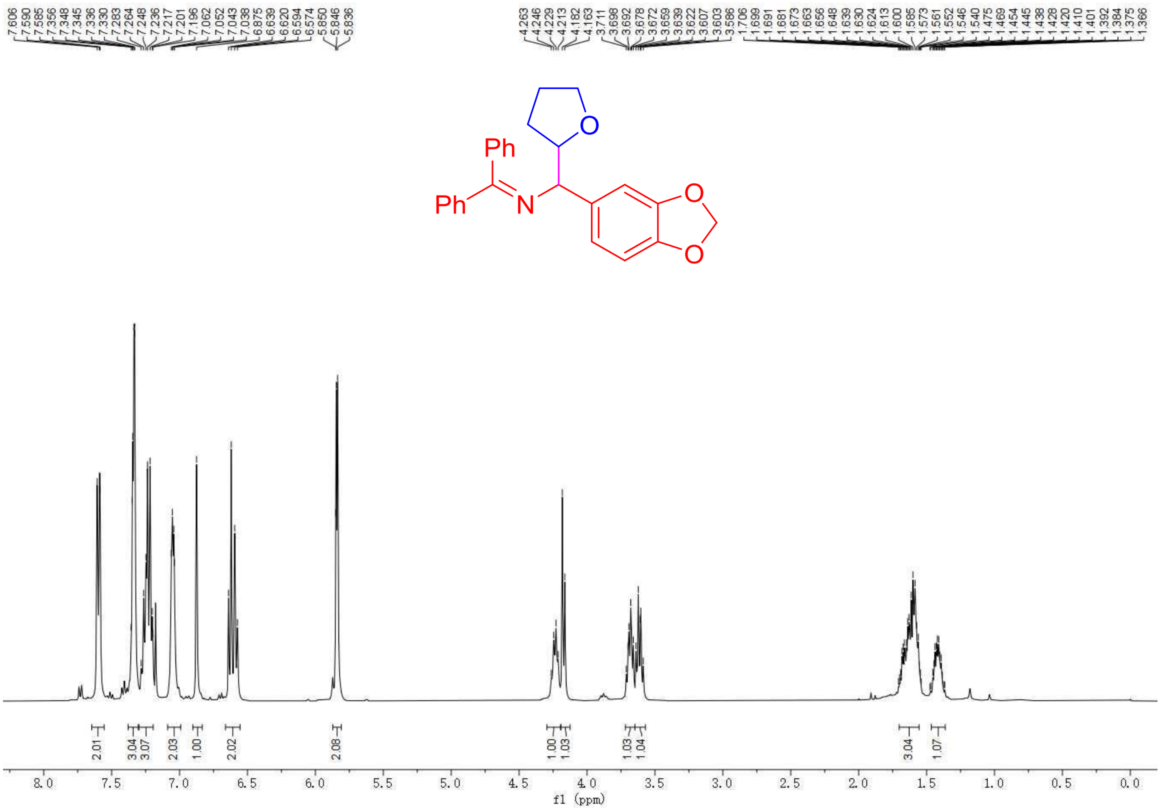

Figure S46. **^{13}C{^{1}H} NMR spectra (100 MHz, Chloroform-*d*) of *N*-(benzo[*d*][1,3]dioxol-5-yl(tetrahydrofuran-2-yl)methyl)-1,1-diphenylmethanimine (4da**(minor)**):**

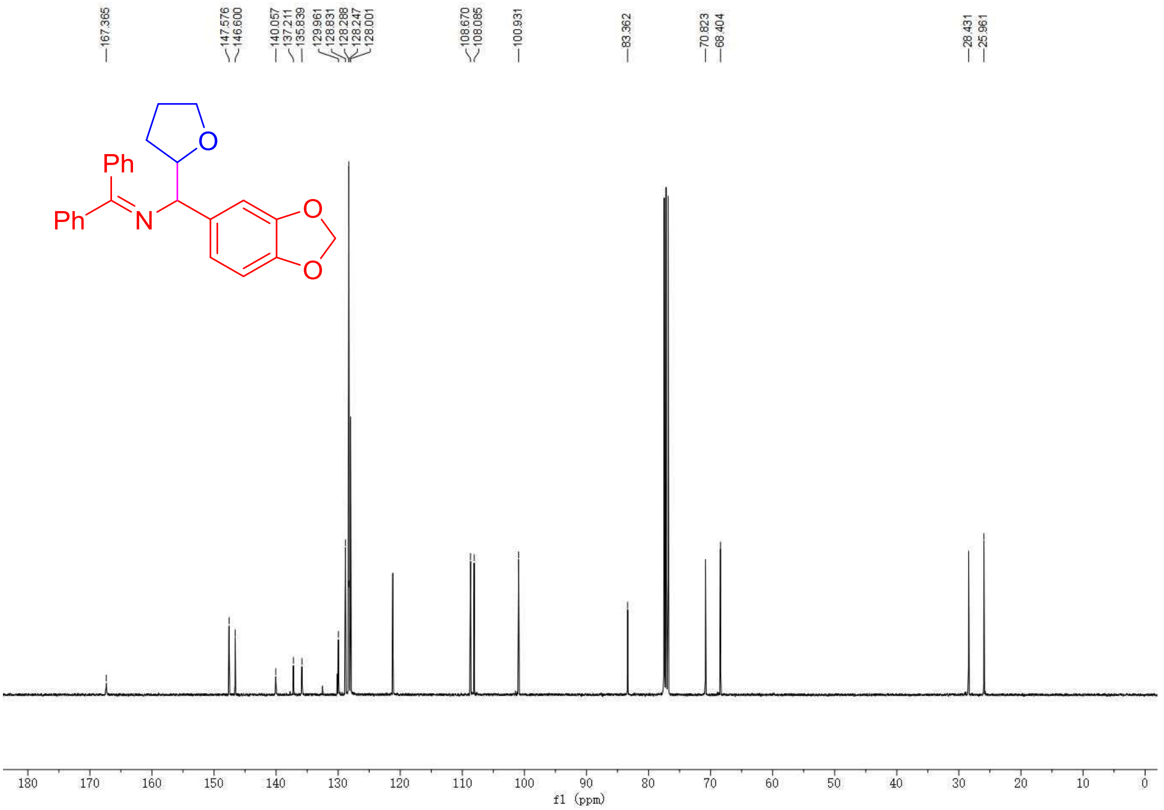

Figure S47. ^{1}**H NMR spectra (600 MHz, Chloroform-*d*) of *N*-((4-fluorophenyl)(tetrahydrofuran-2-yl)methyl)-1,1-diphenylmethanimine (4ea**(major)**):**

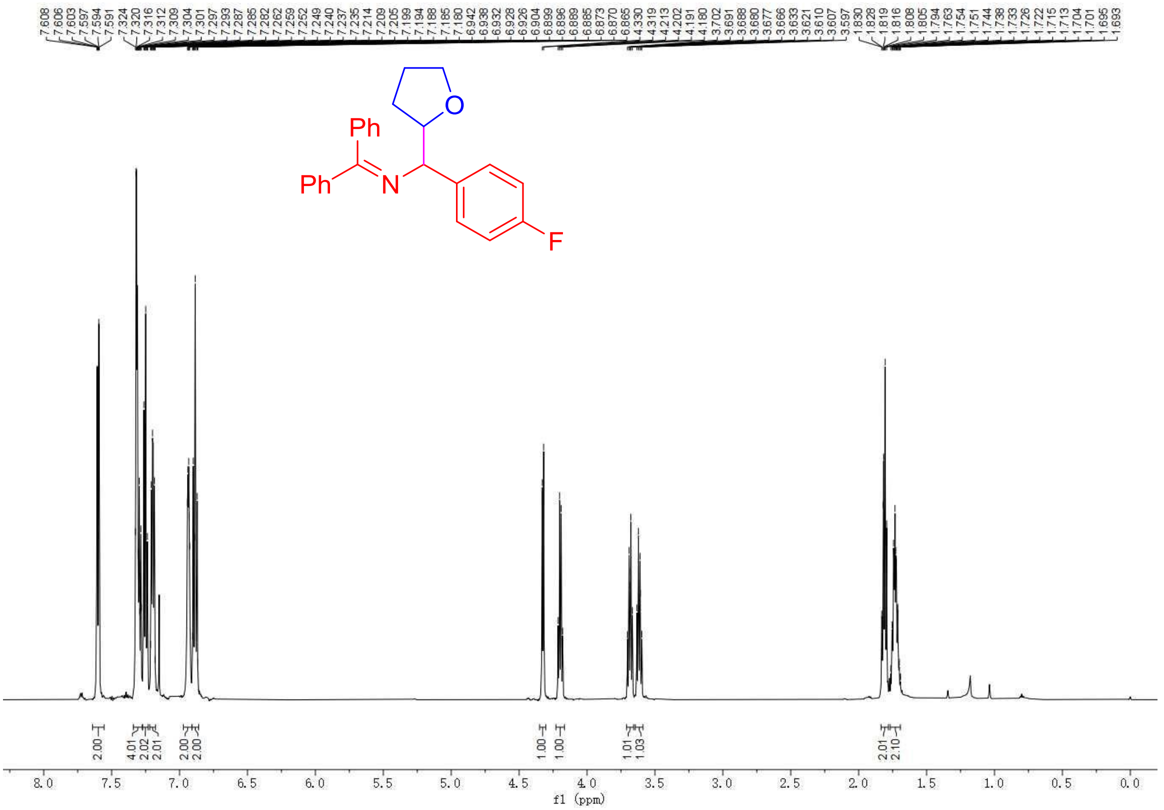

Figure S48. ^{13}**C{**^{1}**H} NMR spectra (150 MHz, Chloroform-*d*) of *N*-((4-fluorophenyl)(tetrahydrofuran-2-yl)methyl)-1,1-diphenylmethanimine (4ea**(major)**):**

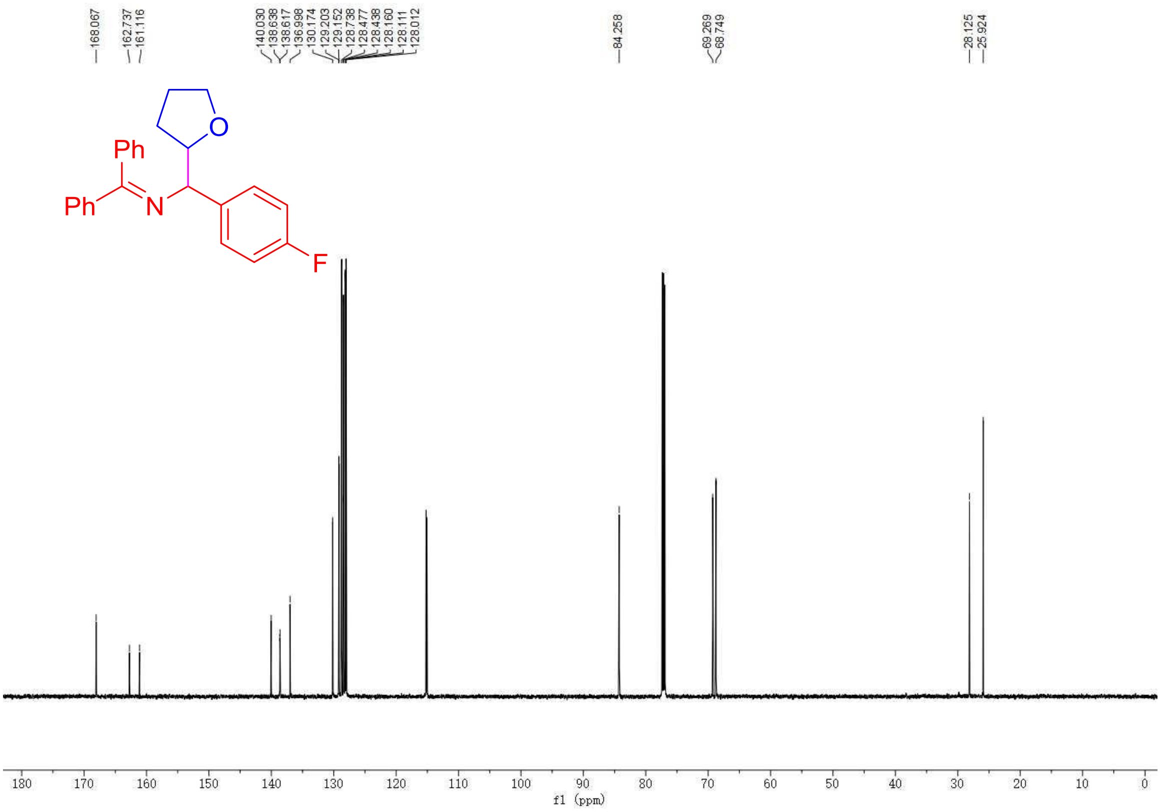

Figure S49. **^{1}H NMR spectra (600 MHz, Chloroform-*d*) of *N*-((4-fluorophenyl)(tetrahydrofuran-2-yl)methyl)-1,1-diphenylmethanimine (4ea**(minor)**):**

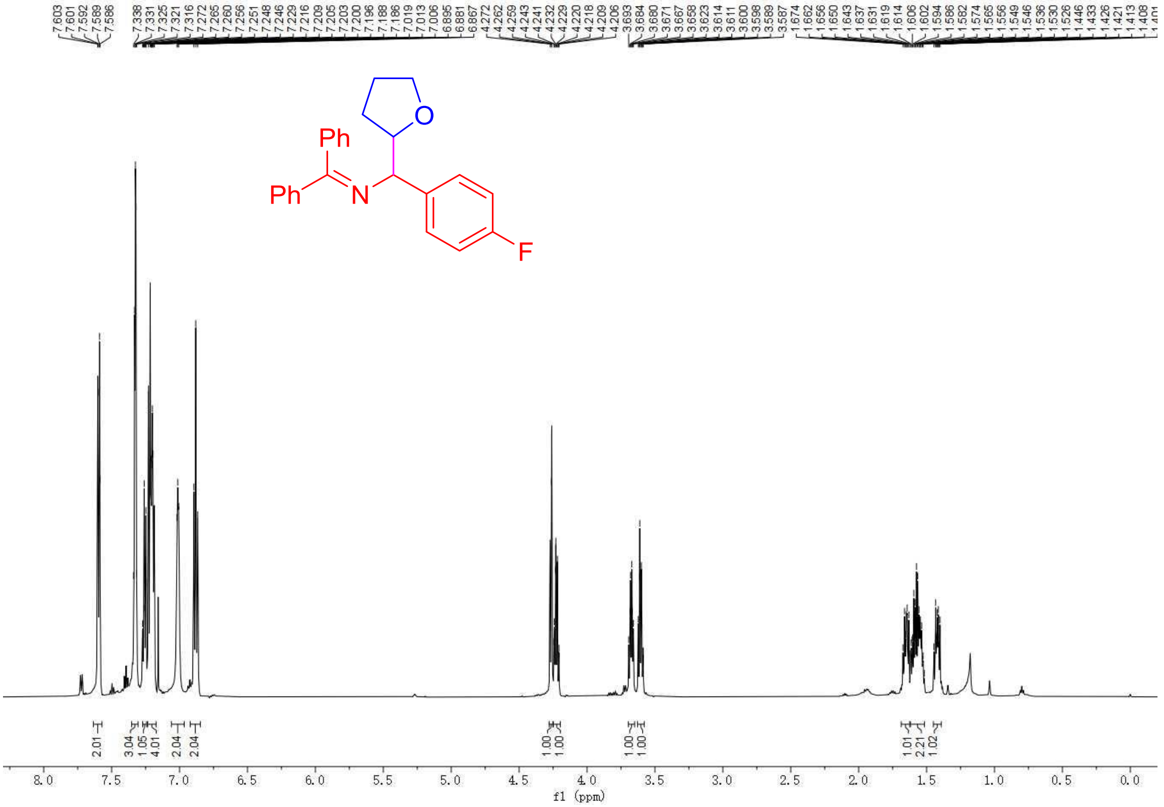

Figure S50. **^{13}C{^{1}H} NMR spectra (150 MHz, Chloroform-*d*) of *N*-((4-fluorophenyl)(tetrahydrofuran-2-yl)methyl)-1,1-diphenylmethanimine (4ea**(minor)**):**

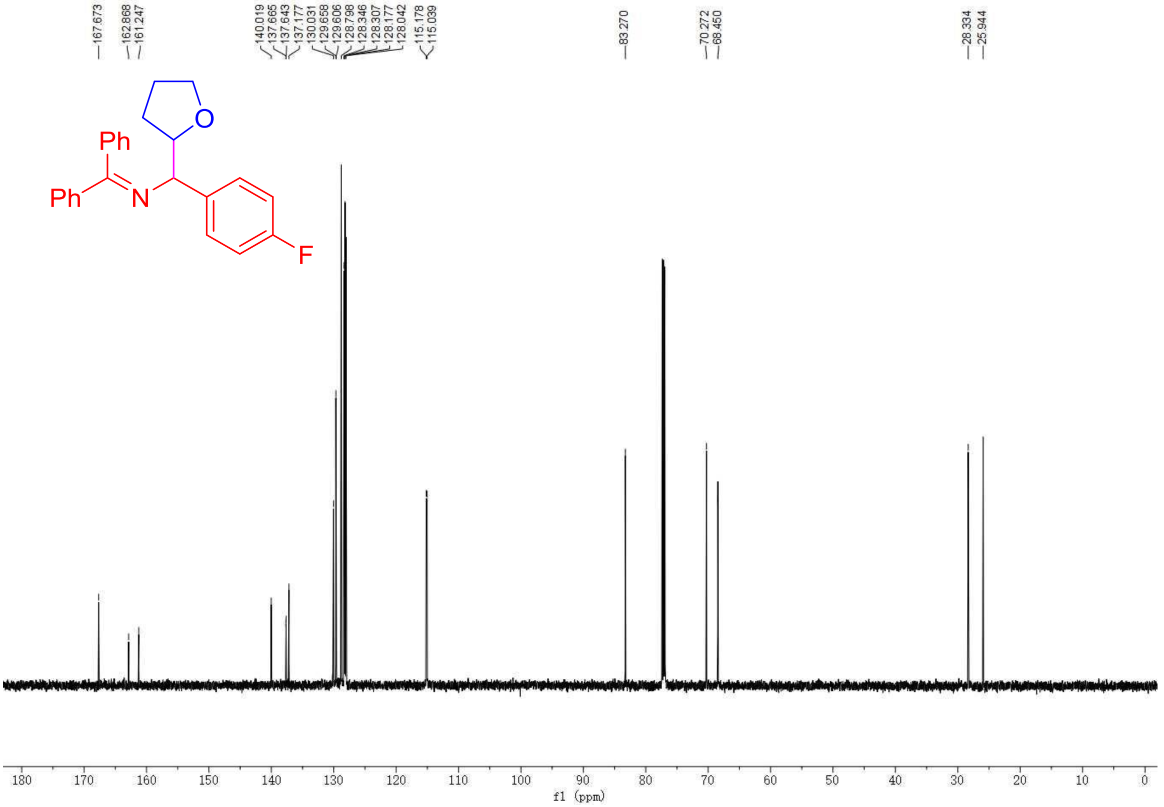

Figure S51. **^{1}H NMR spectra (400 MHz, Chloroform-*d*) of *N*-((4-chlorophenyl)(tetrahydrofuran-2-yl)methyl)-1,1-diphenylmethanimine (4fa**(major)**):**

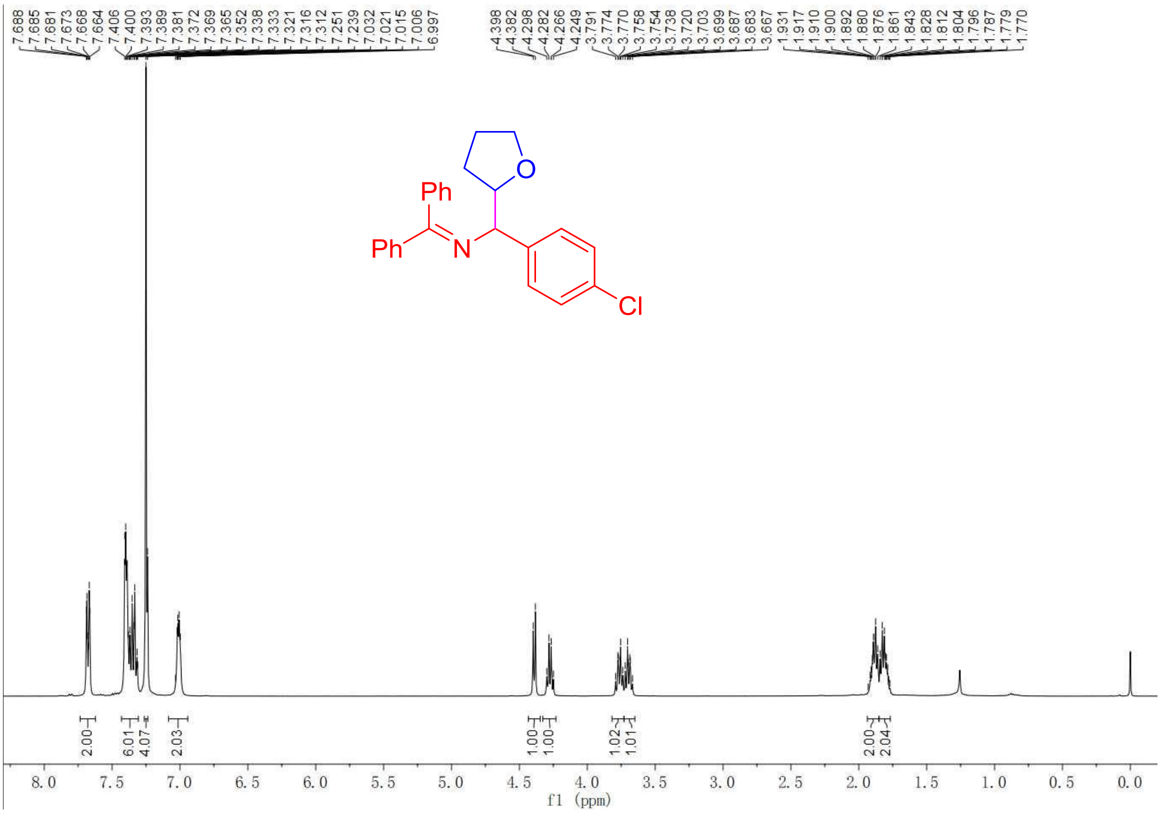

Figure S52. **^{13}C{^{1}H} NMR spectra (100 MHz, Chloroform-*d*) of *N*-((4-chlorophenyl)(tetrahydrofuran-2-yl)methyl)-1,1-diphenylmethanimine (4fa**(major)**):**

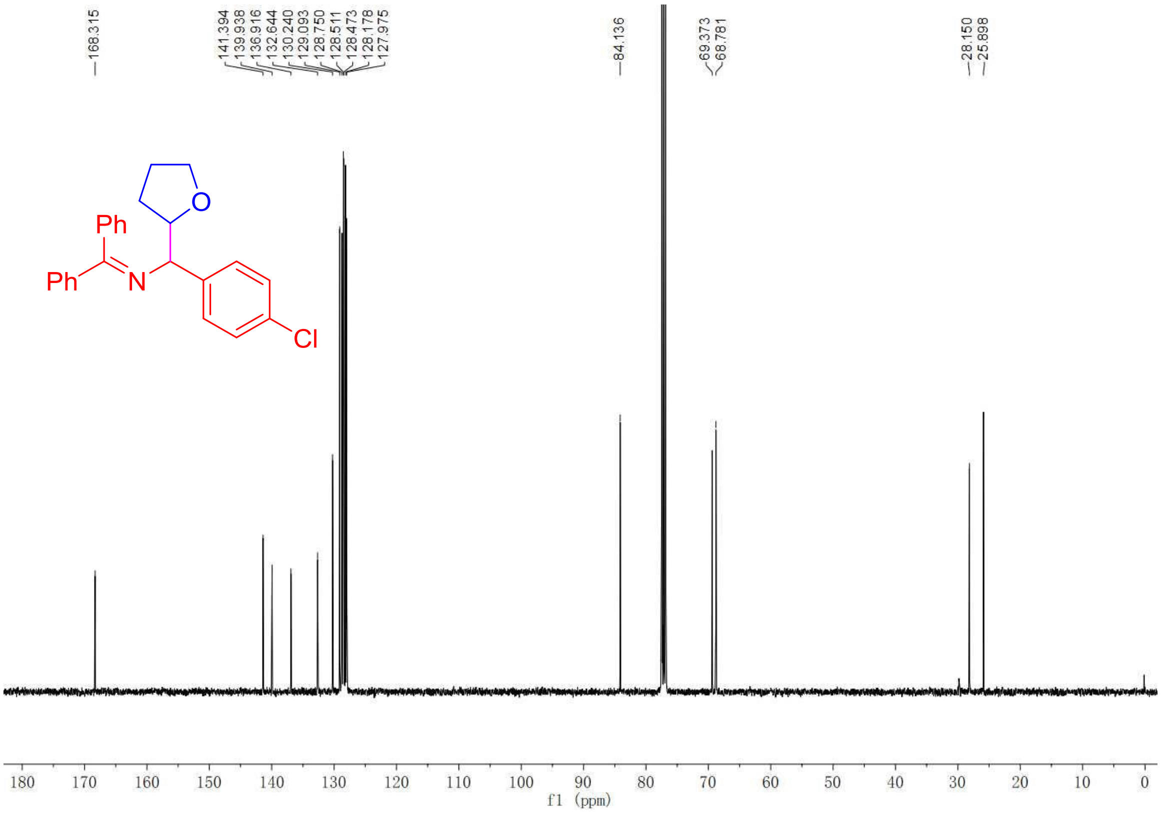

Figure S53. ^{1}H **NMR spectra (400 MHz, Chloroform-*d*) of *N*-((4-chlorophenyl)(tetrahydrofuran-2-yl)methyl)-1,1-diphenylmethanimine (4fa**(minor)**):**

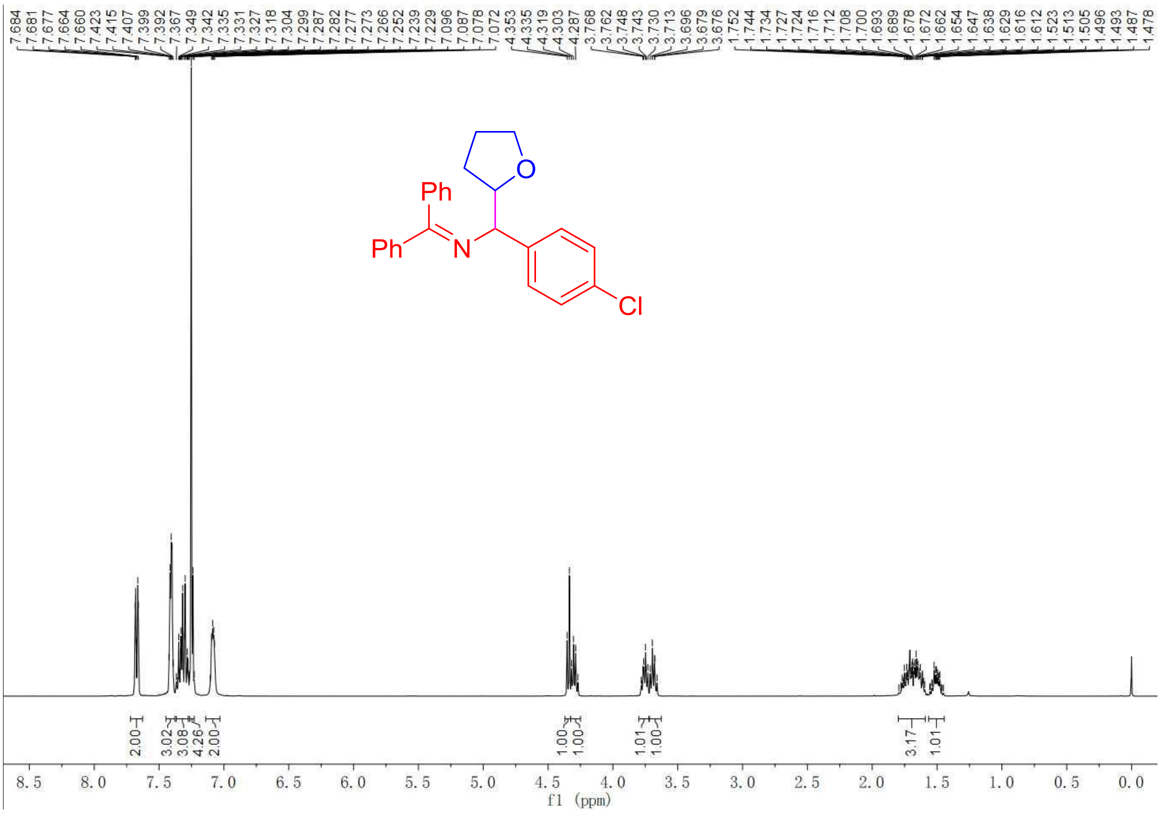

Figure S54. $^{13}C\{^{1}H\}$ **NMR spectra (100 MHz, Chloroform-*d*) of *N*-((4-chlorophenyl)(tetrahydrofuran-2-yl)methyl)-1,1-diphenylmethanimine (4fa**(minor)**):**

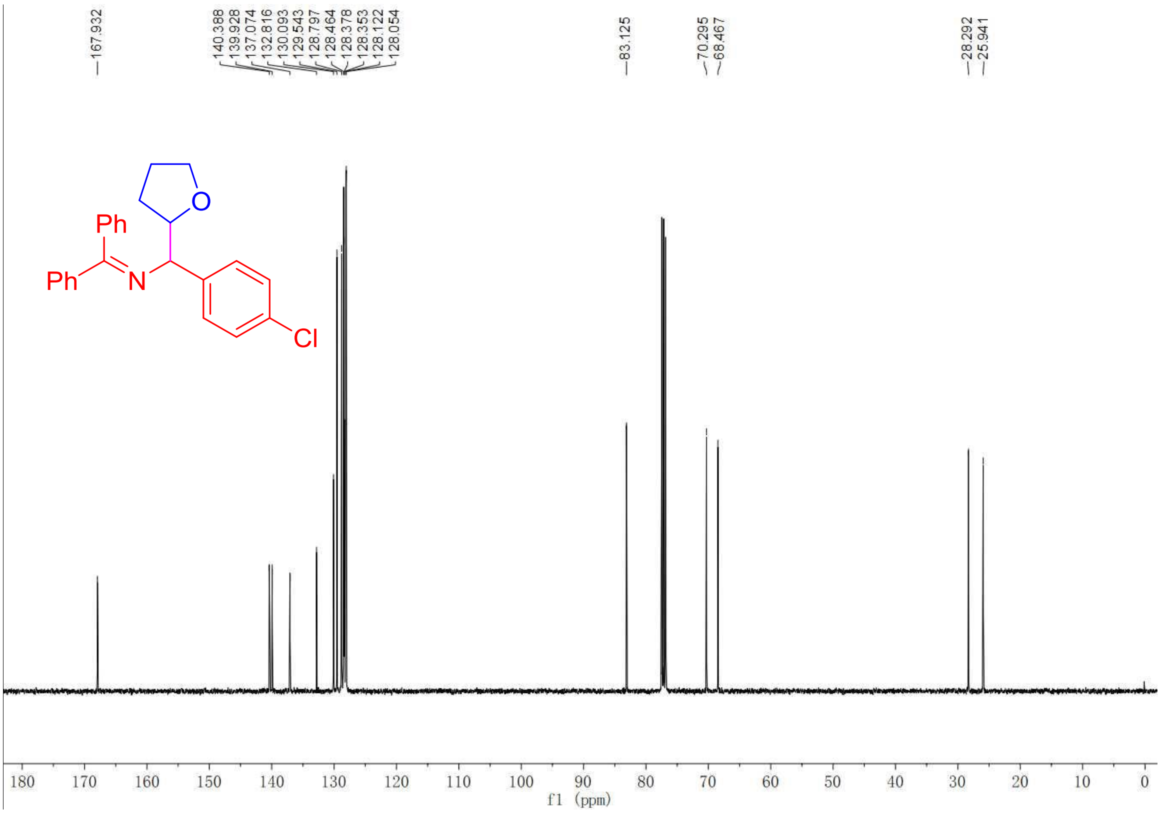

Figure S55. **^{1}H NMR spectra (400 MHz, Chloroform-*d*) of *N*-((4-bromophenyl)(tetrahydrofuran-2-yl)methyl)-1,1-diphenylmethanimine (4ga**(major)**):**

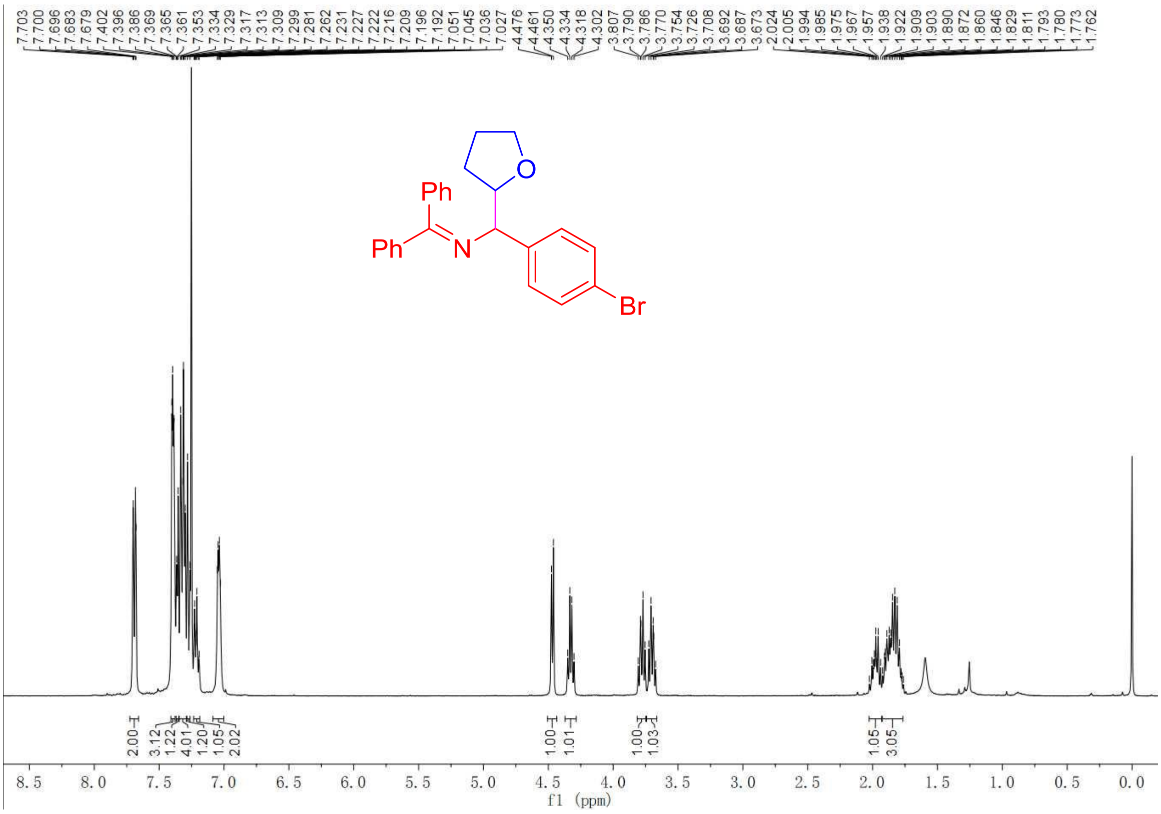

Figure S56. **^{13}C{^{1}H} NMR spectra (100 MHz, Chloroform-*d*) of *N*-((4-bromophenyl)(tetrahydrofuran-2-yl)methyl)-1,1-diphenylmethanimine (4ga**(major)**):**

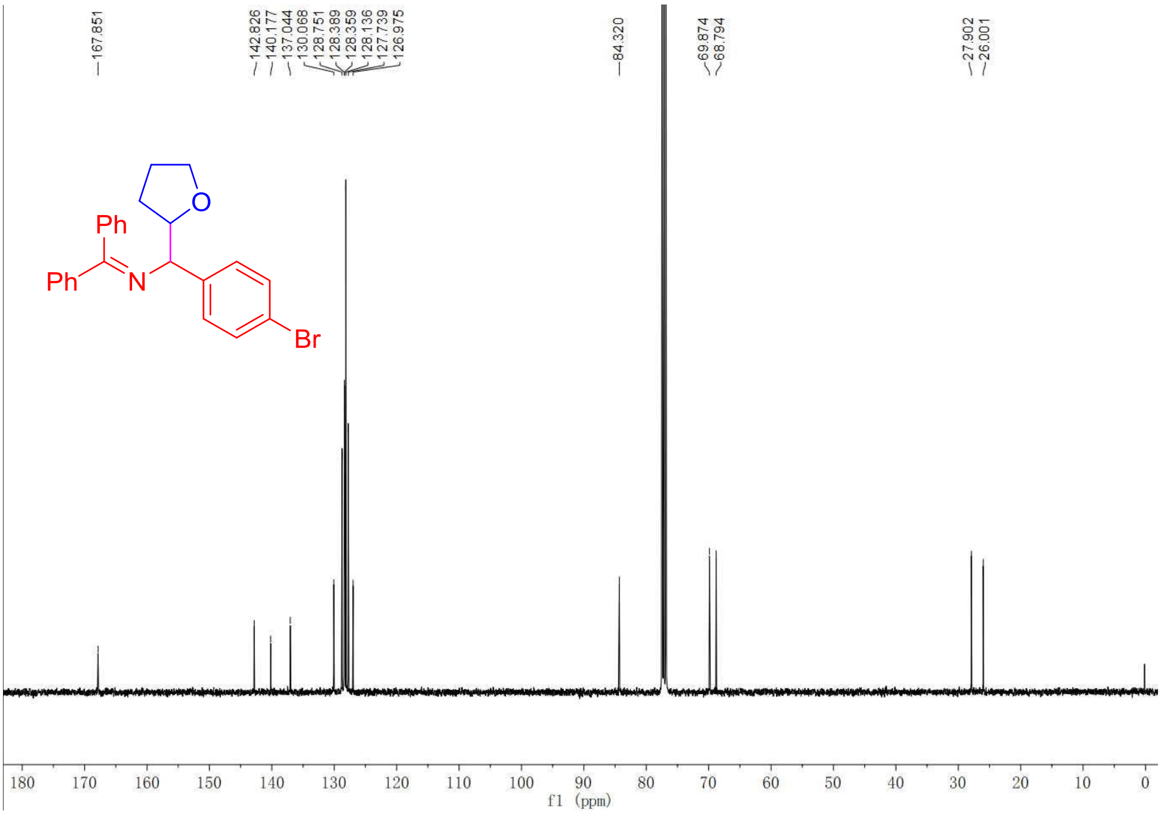

Figure S57. ^{1}**H NMR spectra (400 MHz, Chloroform-*d*) of *N*-((4-bromophenyl)(tetrahydrofuran-2-yl)methyl)-1,1-diphenylmethanimine (4ga**(minor)**):**

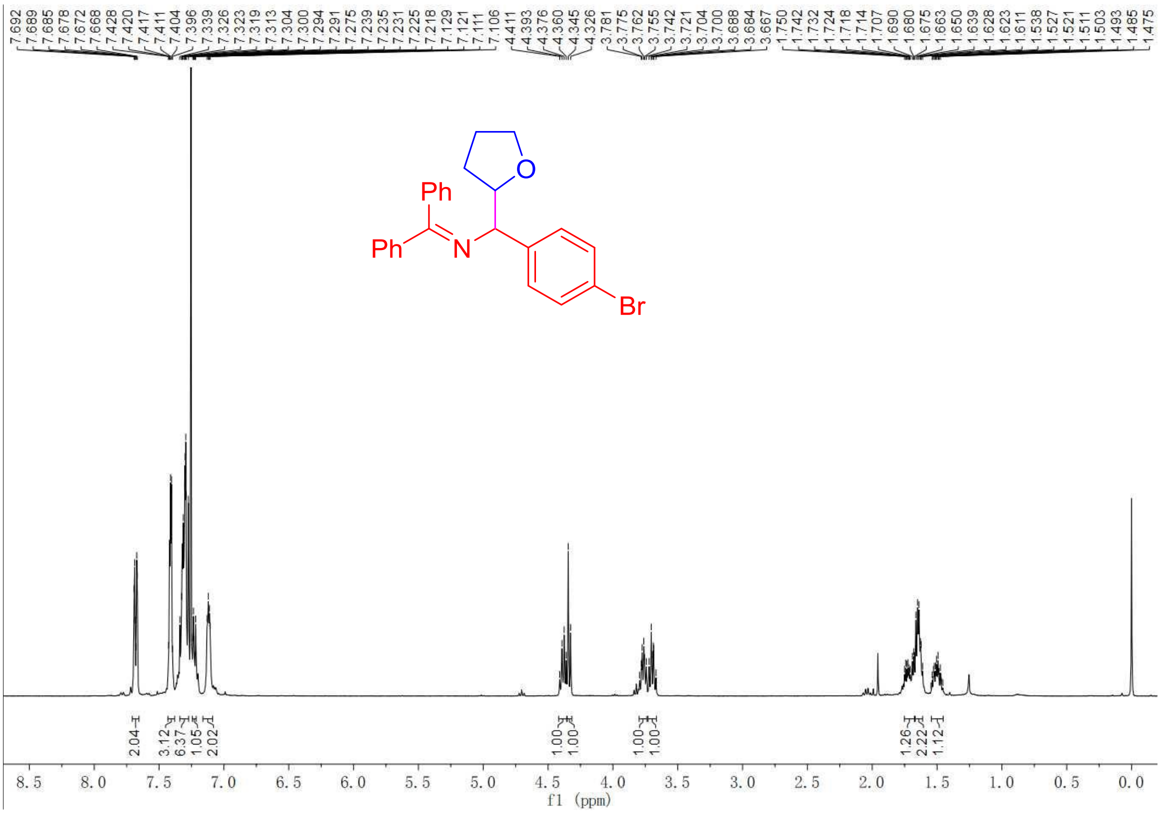

Figure S58. ^{13}**C{**^{1}**H} NMR spectra (100 MHz, Chloroform-*d*) of *N*-((4-bromophenyl)(tetrahydrofuran-2-yl)methyl)-1,1-diphenylmethanimine (4ga**(minor)**):**

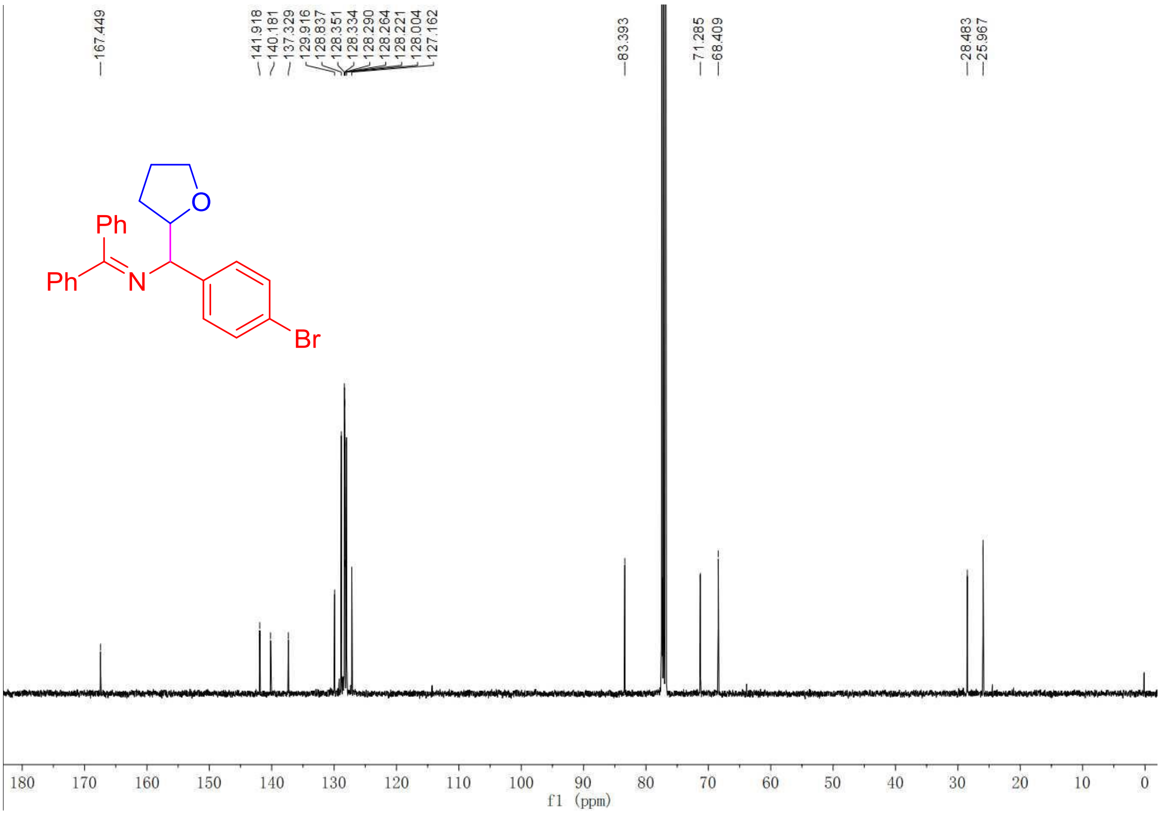

Figure S59. ^{1}**H NMR spectra (400 MHz, Chloroform-*d*) of *N*-([1,1'-biphenyl]-4-yl(tetrahydrofuran-2-yl)methyl)-1,1-diphenylmethanimine (4ha**(major)**):**

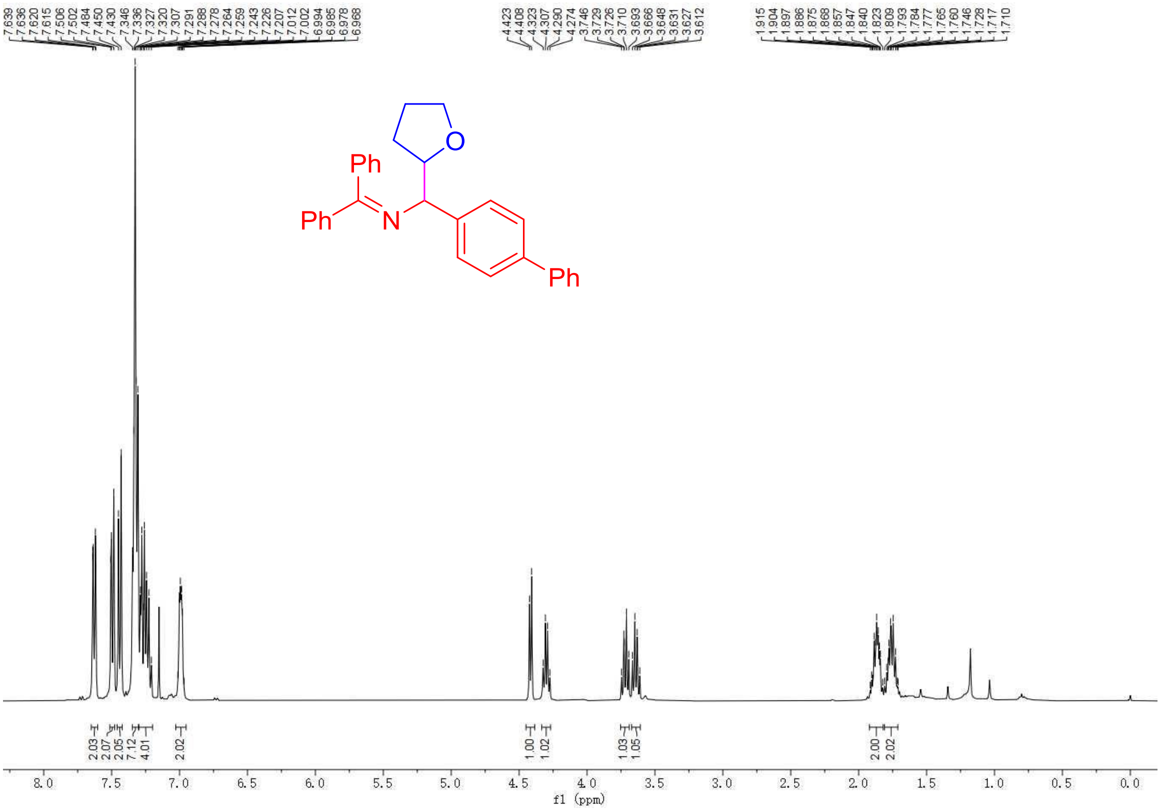

Figure S60. ^{13}**C{**^{1}**H} NMR spectra (100 MHz, Chloroform-*d*) of *N*-([1,1'-biphenyl]-4-yl(tetrahydrofuran-2-yl)methyl)-1,1-diphenylmethanimine (4ha**(major)**):**

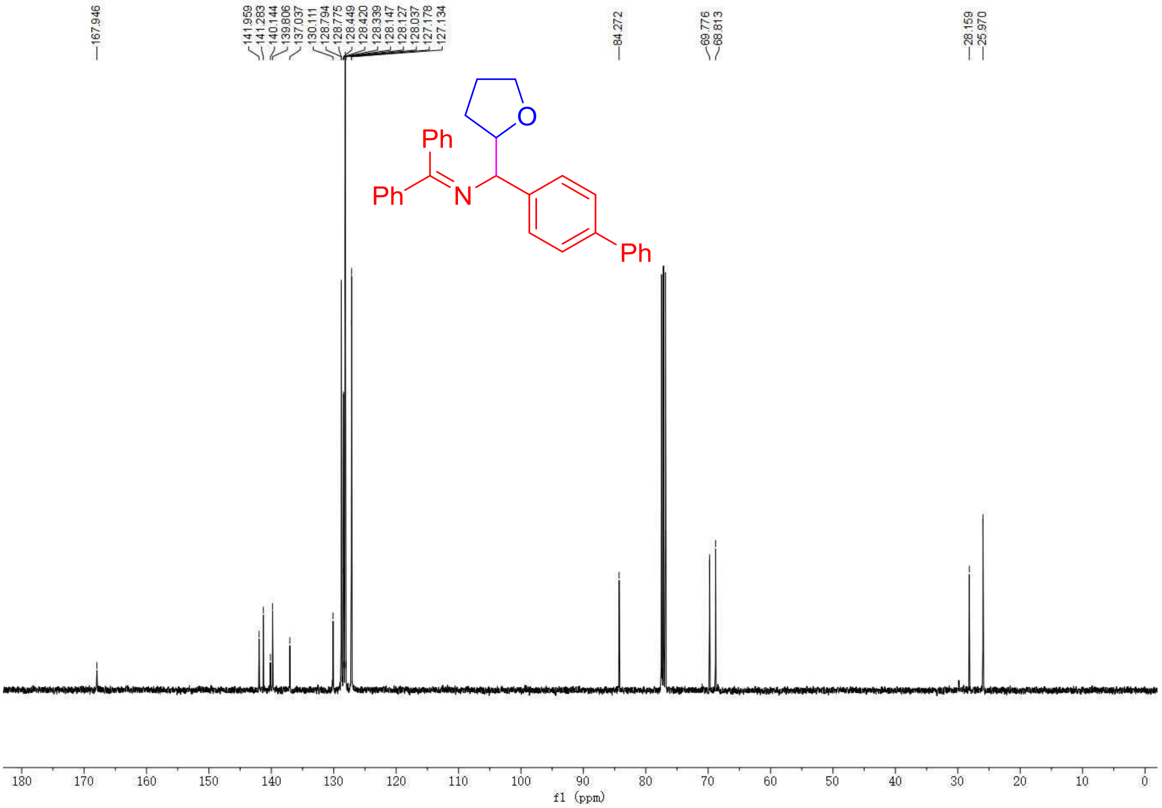

Figure S61. ^{1}H NMR spectra (400 MHz, Chloroform-*d*) of *N*-([1,1'-biphenyl]-4-yl(tetrahydrofuran-2-yl)methyl)-1,1-diphenylmethanimine (4ha(minor)**):**

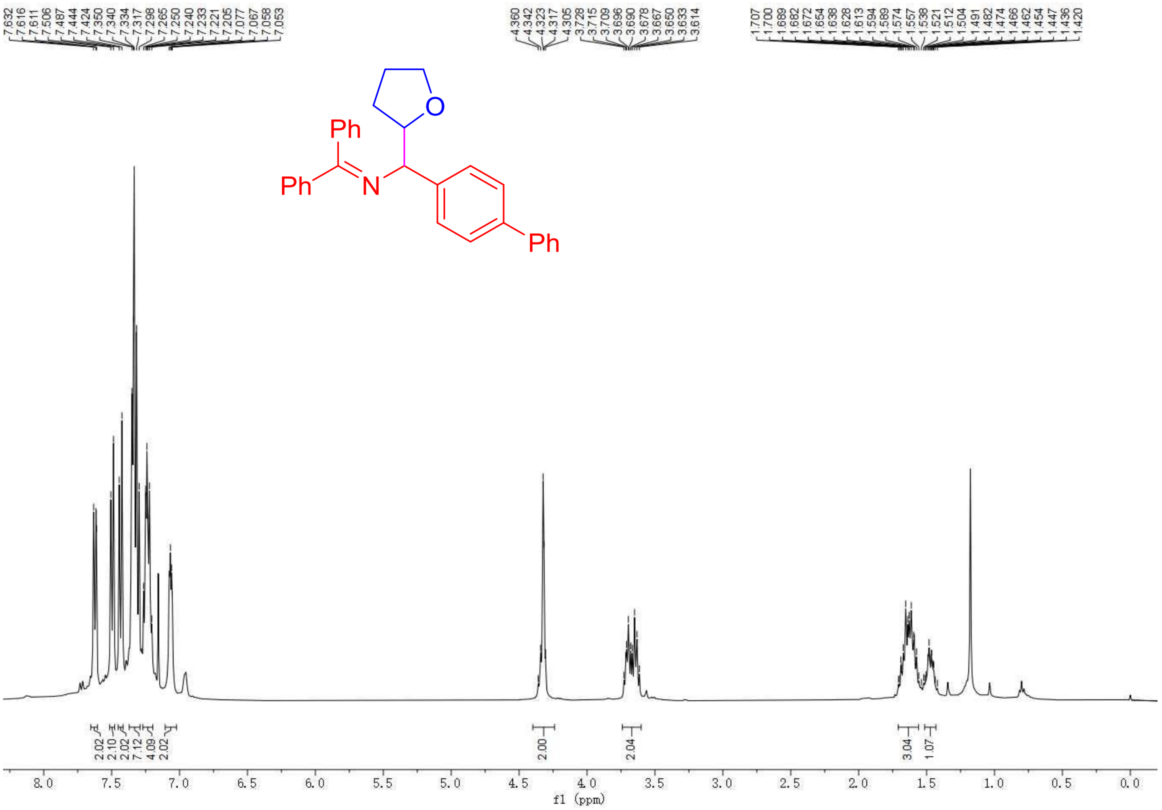

Figure S62. ^{13}C{^{1}H} NMR spectra (100 MHz, Chloroform-*d*) of *N*-([1,1'-biphenyl]-4-yl(tetrahydrofuran-2-yl)methyl)-1,1-diphenylmethanimine (4ha(minor)**):**

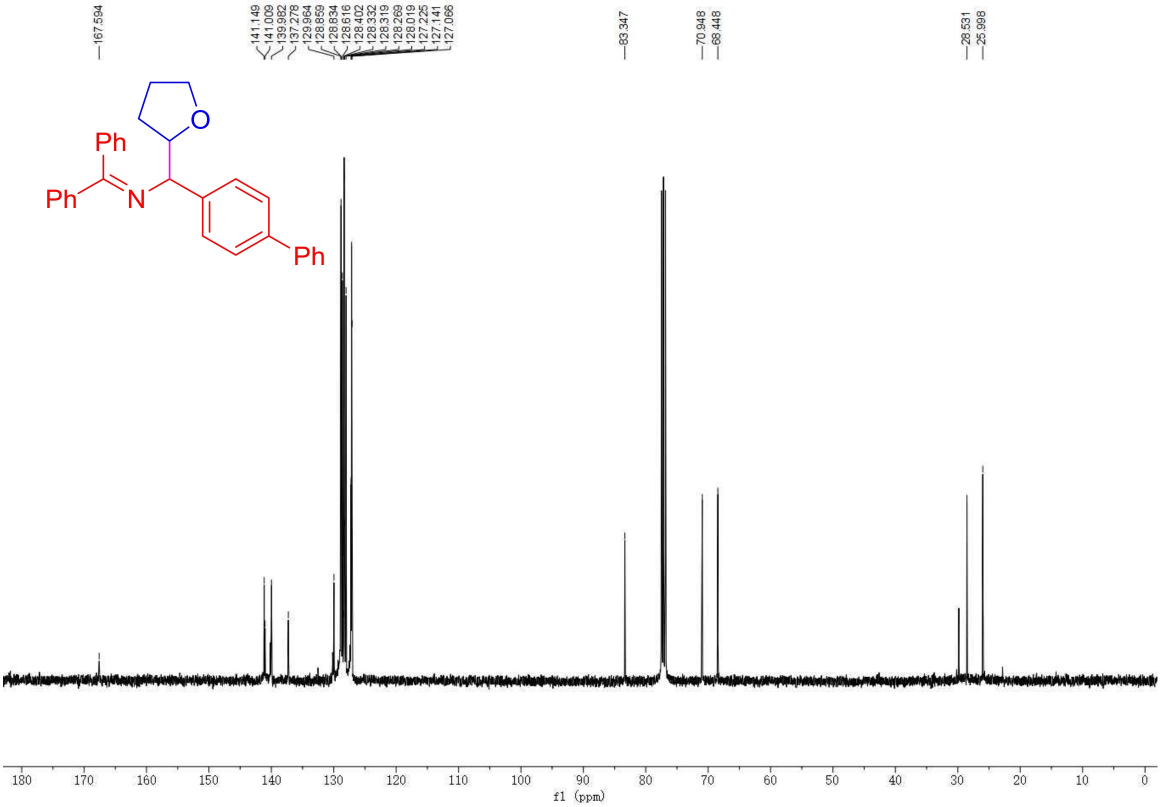

Figure S63. **^{1}H NMR spectra (400 MHz, Chloroform-*d*) of *N*-(naphthalen-1-yl(tetrahydrofuran-2-yl)methyl)-1,1-diphenylmethanimine (4ia**(major)**):**

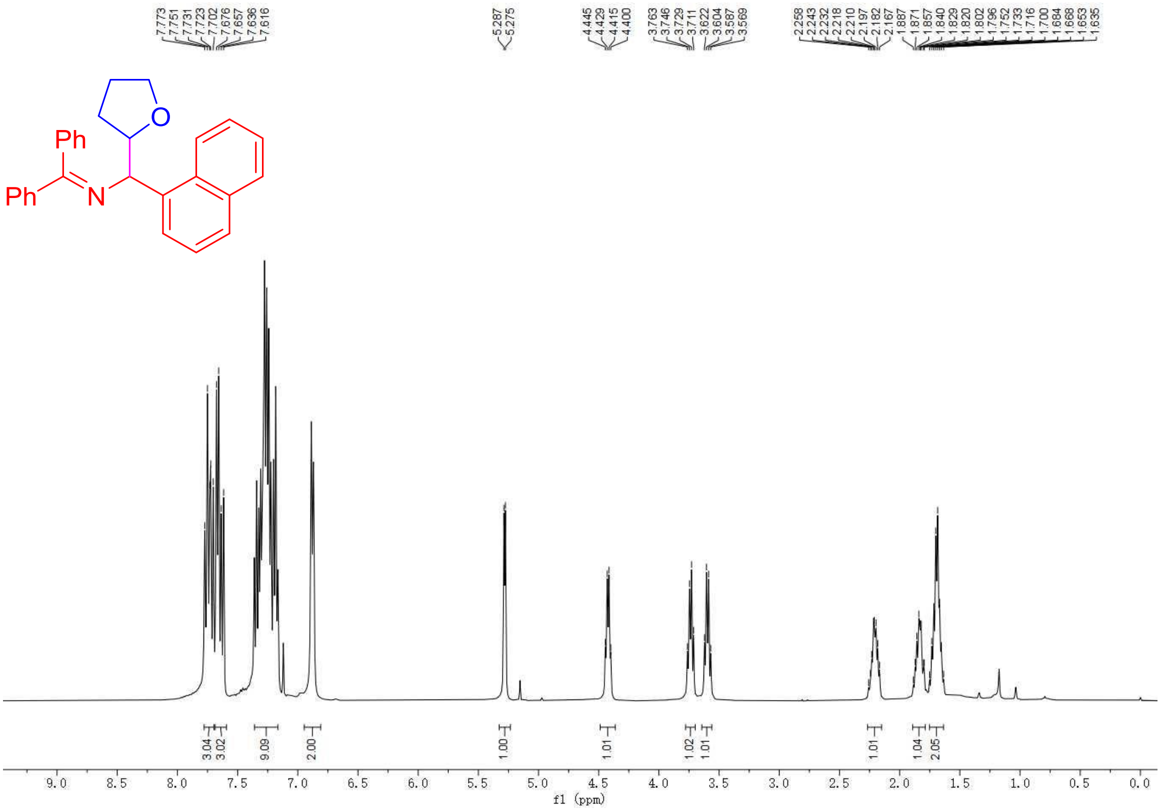

Figure S64. **^{13}C{^{1}H} NMR spectra (100 MHz, Chloroform-*d*) of *N*-(naphthalen-1-yl(tetrahydrofuran-2-yl)methyl)-1,1-diphenylmethanimine (4ia**(major)**):**

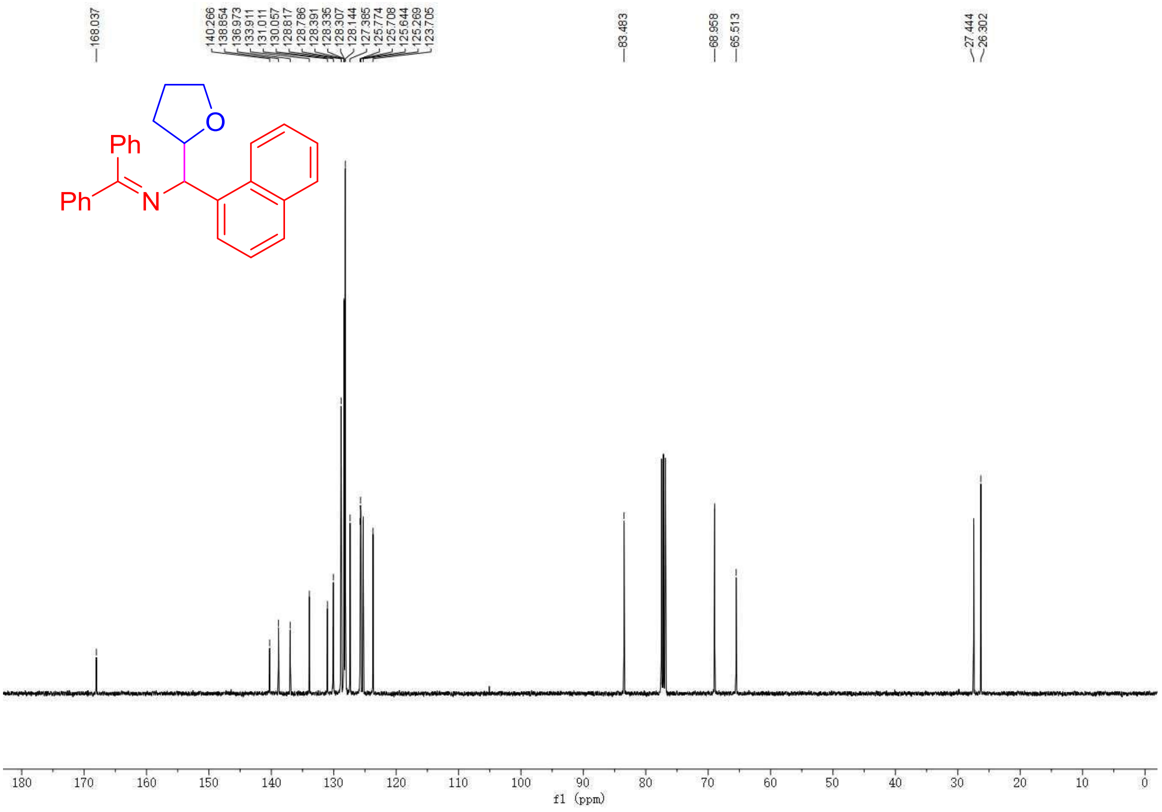

Figure S65. **^{1}H NMR spectra (400 MHz, Chloroform-*d*) of *N*-(naphthalen-1-yl(tetrahydrofuran-2-yl)methyl)-1,1-diphenylmethanimine (4ia**(minor)**):**

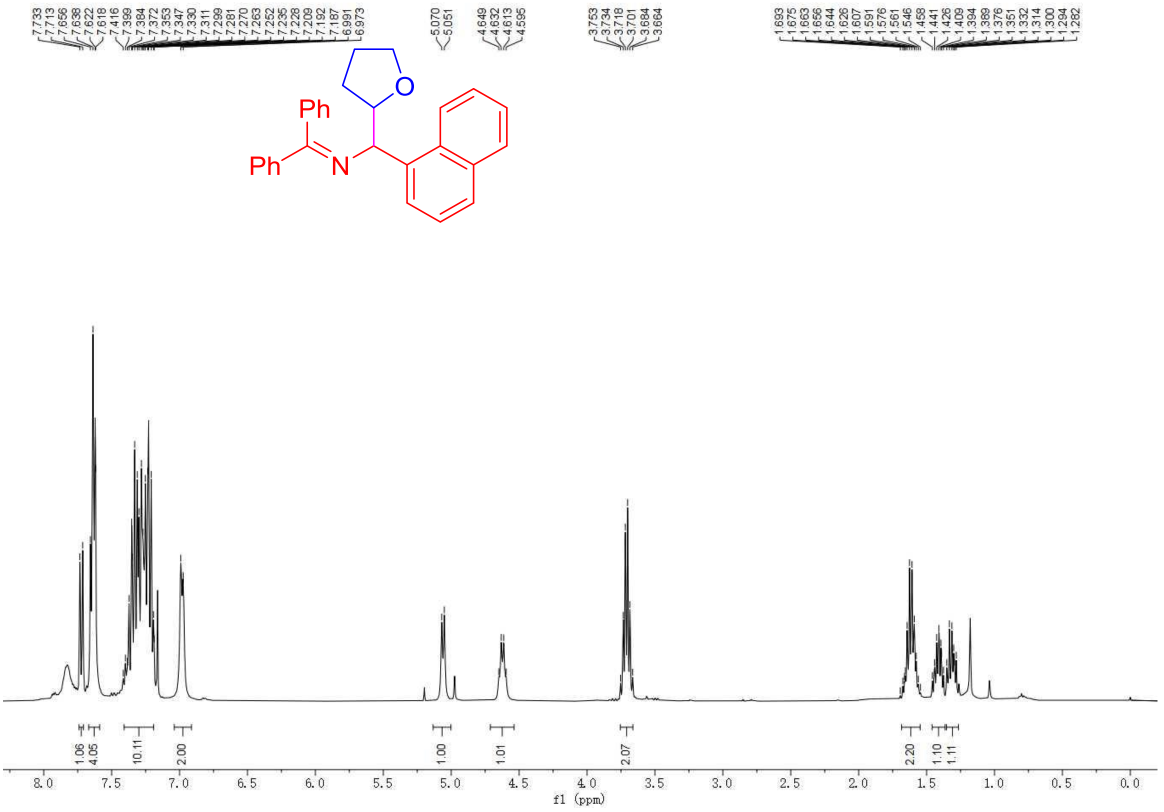

Figure S66. **^{13}C{^{1}H} NMR spectra (100 MHz, Chloroform-*d*) of *N*-(naphthalen-1-yl(tetrahydrofuran-2-yl)methyl)-1,1-diphenylmethanimine (4ia**(minor)**):**

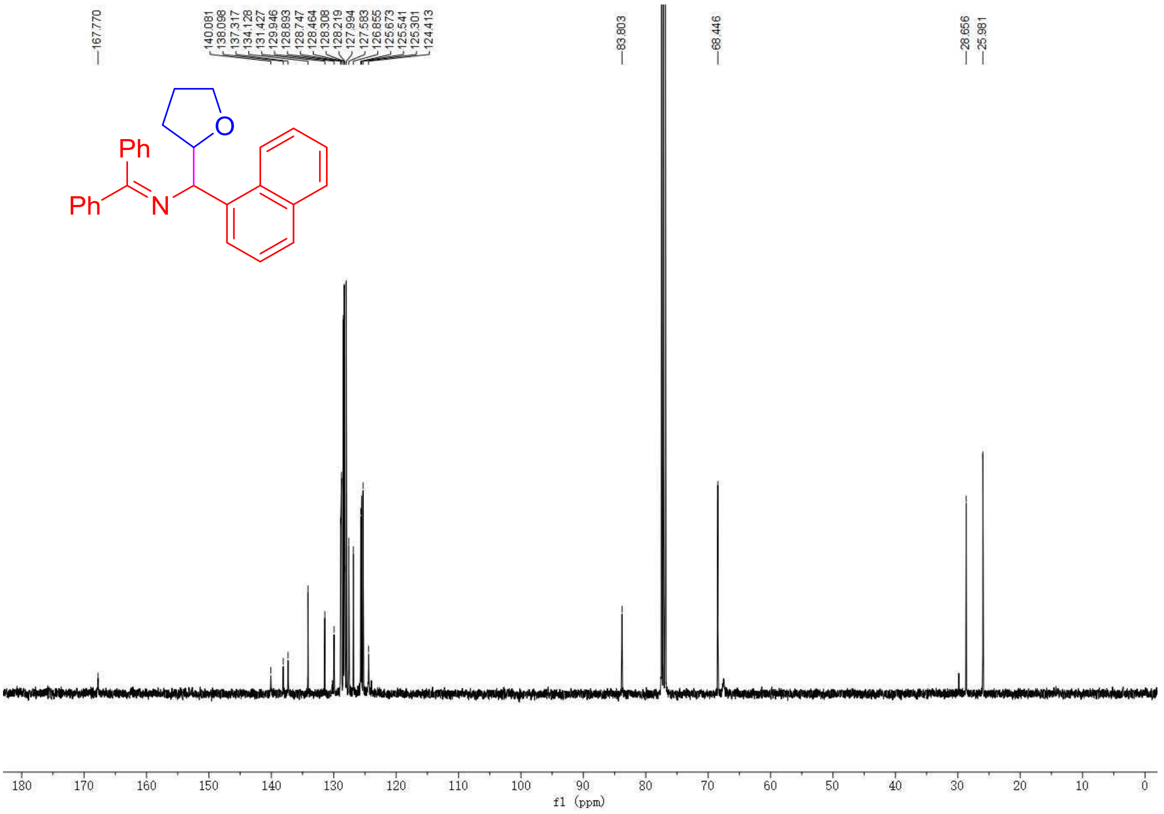

Figure S67. ^{1}H **NMR spectra (400 MHz, Chloroform-*d*) of 1,1-diphenyl-*N*-((tetrahydrofuran-2-yl)(*o*-tolyl)methyl)methanimine (4ja**(major)**):**

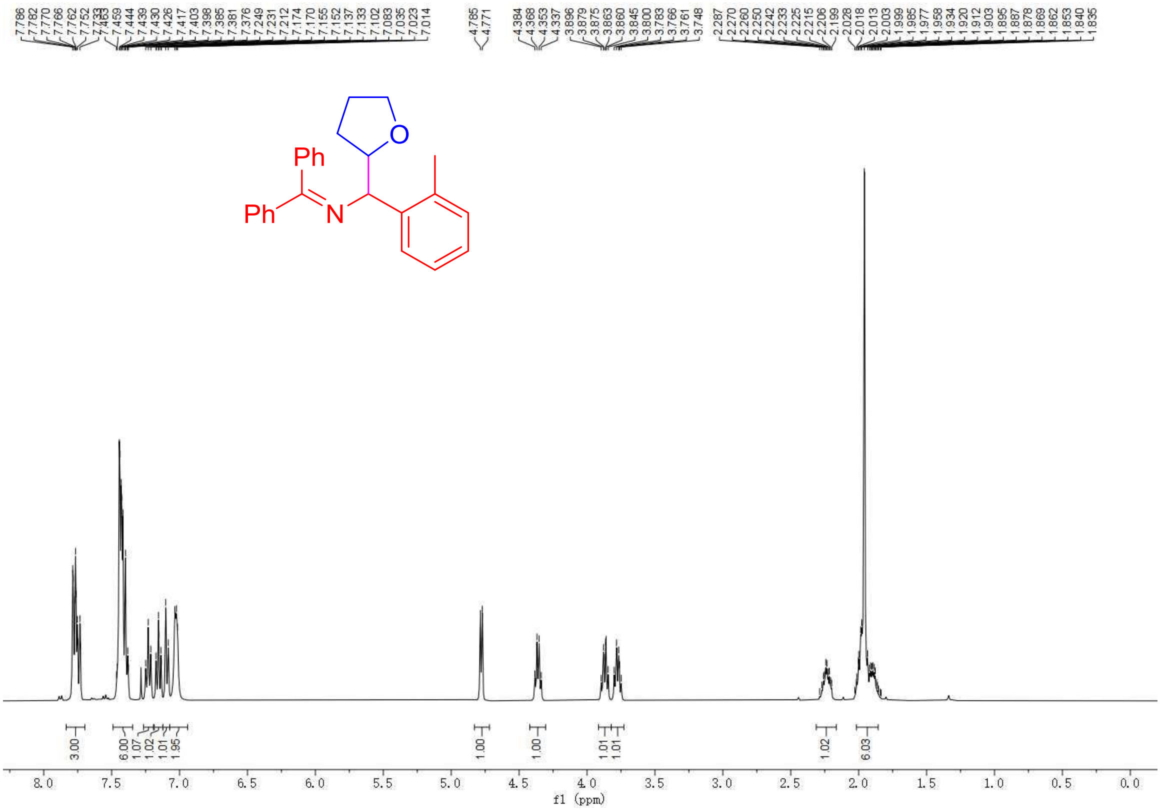

Figure S68. $^{13}C\{^{1}H\}$ **NMR spectra (100 MHz, Chloroform-*d*) of 1,1-diphenyl-*N*-((tetrahydrofuran-2-yl)(*o*-tolyl)methyl)methanimine (4ja**(major)**):**

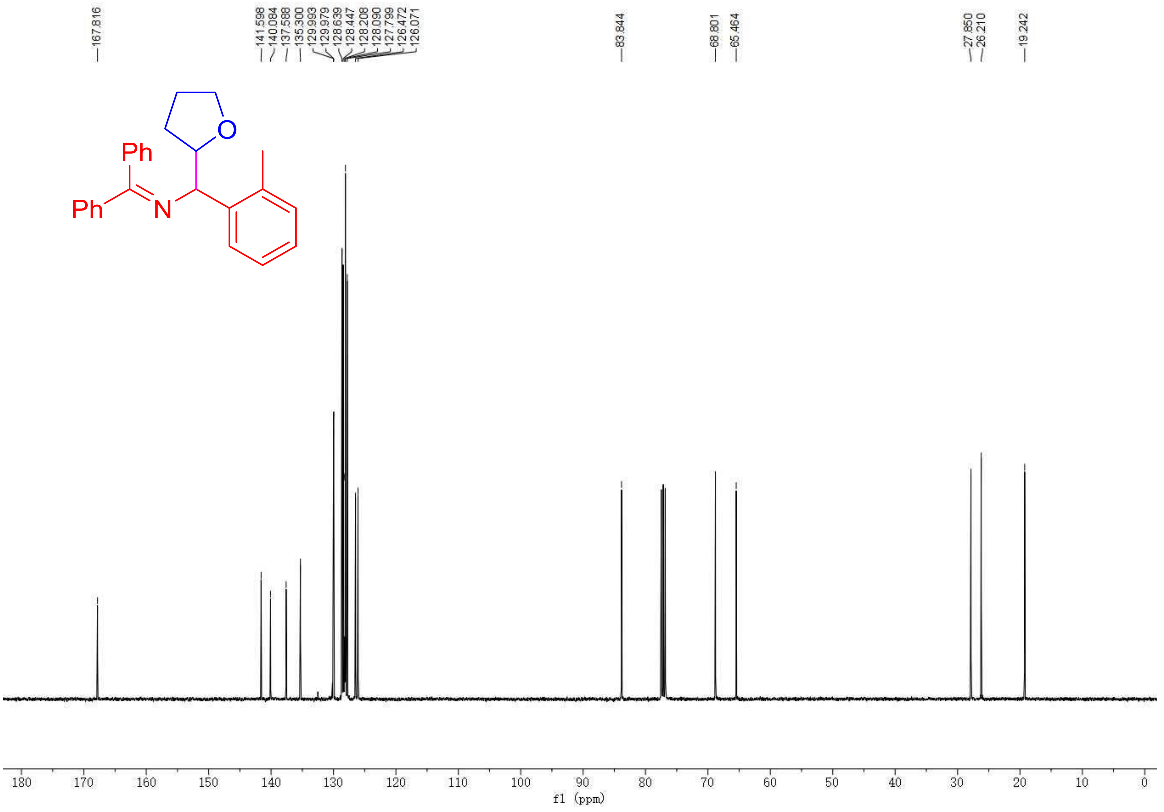

Figure S69. **^{1}H NMR spectra (600 MHz, Chloroform-*d*) of 1,1-diphenyl-*N*-((tetrahydrofuran-2-yl)(*o*-tolyl)methyl)methanimine (4ja**(minor)**):**

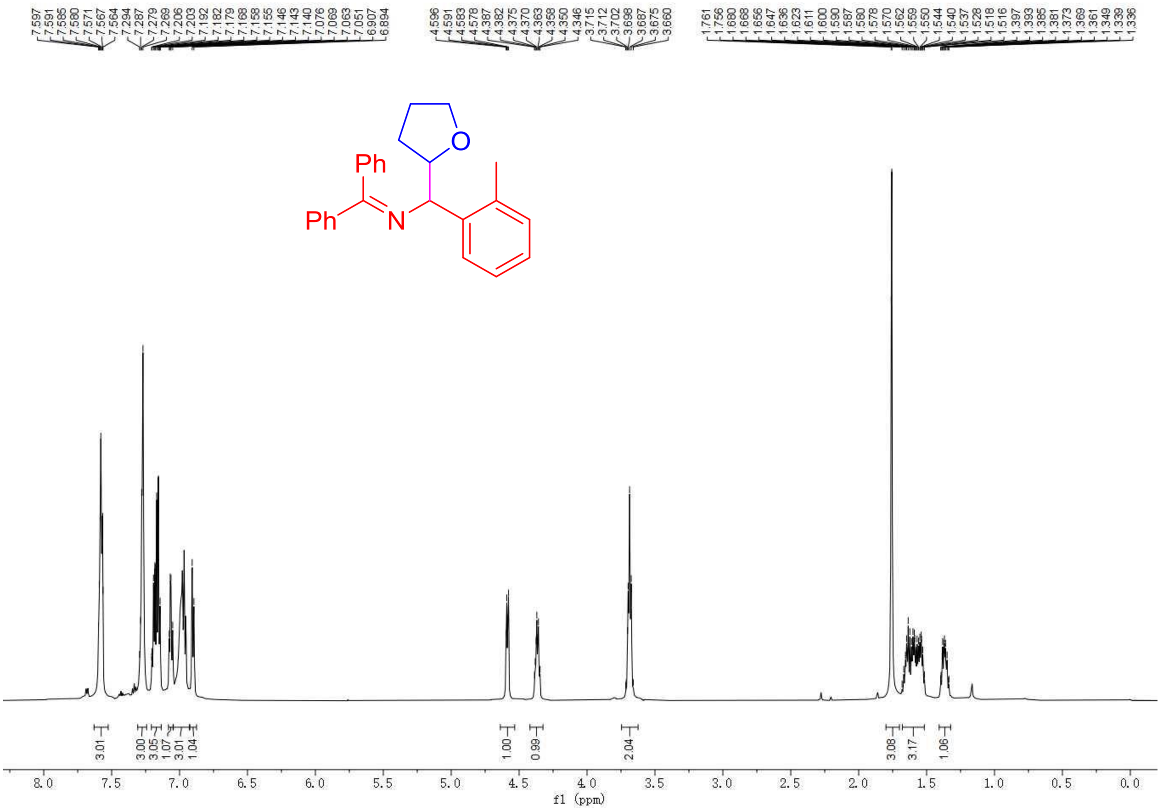

Figure S70. **^{13}C{^{1}H} NMR spectra (150 MHz, Chloroform-*d*) of 1,1-diphenyl-*N*-((tetrahydrofuran-2-yl)(*o*-tolyl)methyl)methanimine (4ja**(minor)**):**

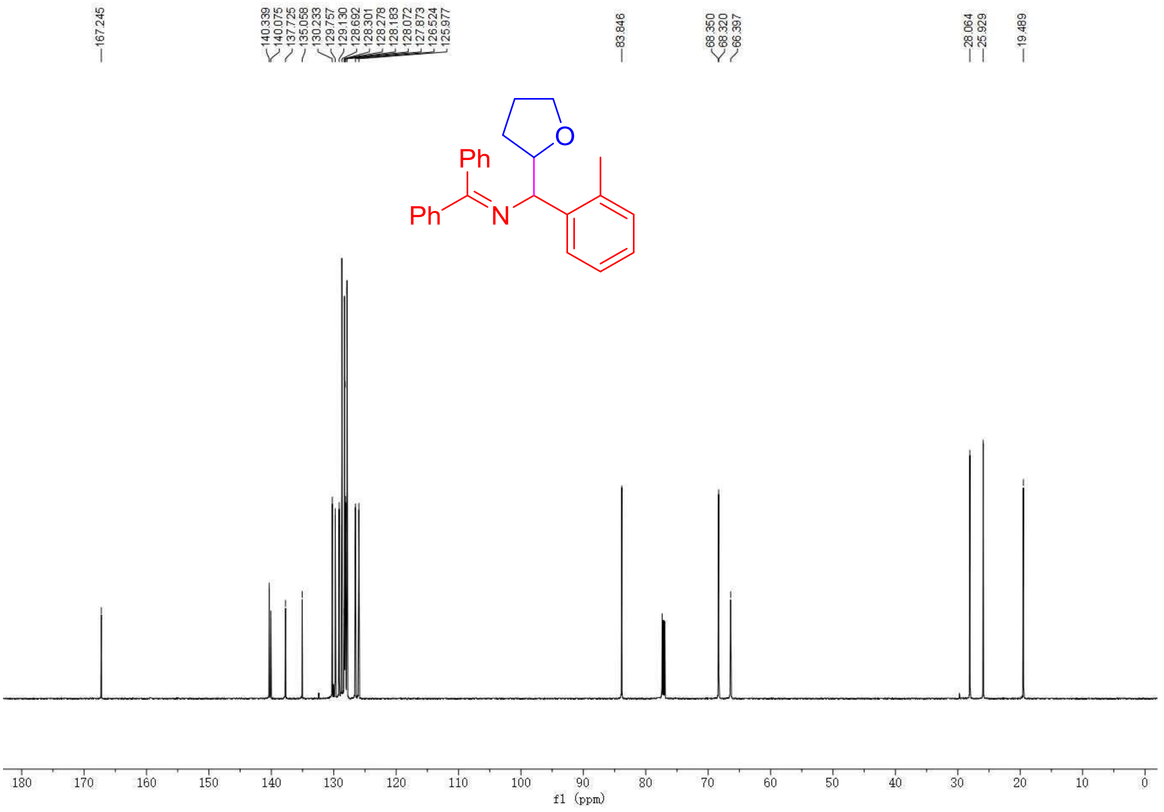

Figure S71. **^{1}H NMR spectra (400 MHz, Chloroform-*d*) of *N*-(diphenyl(tetrahydrofuran-2-yl)methyl)-1,1-diphenylmethanimine (4ka):**

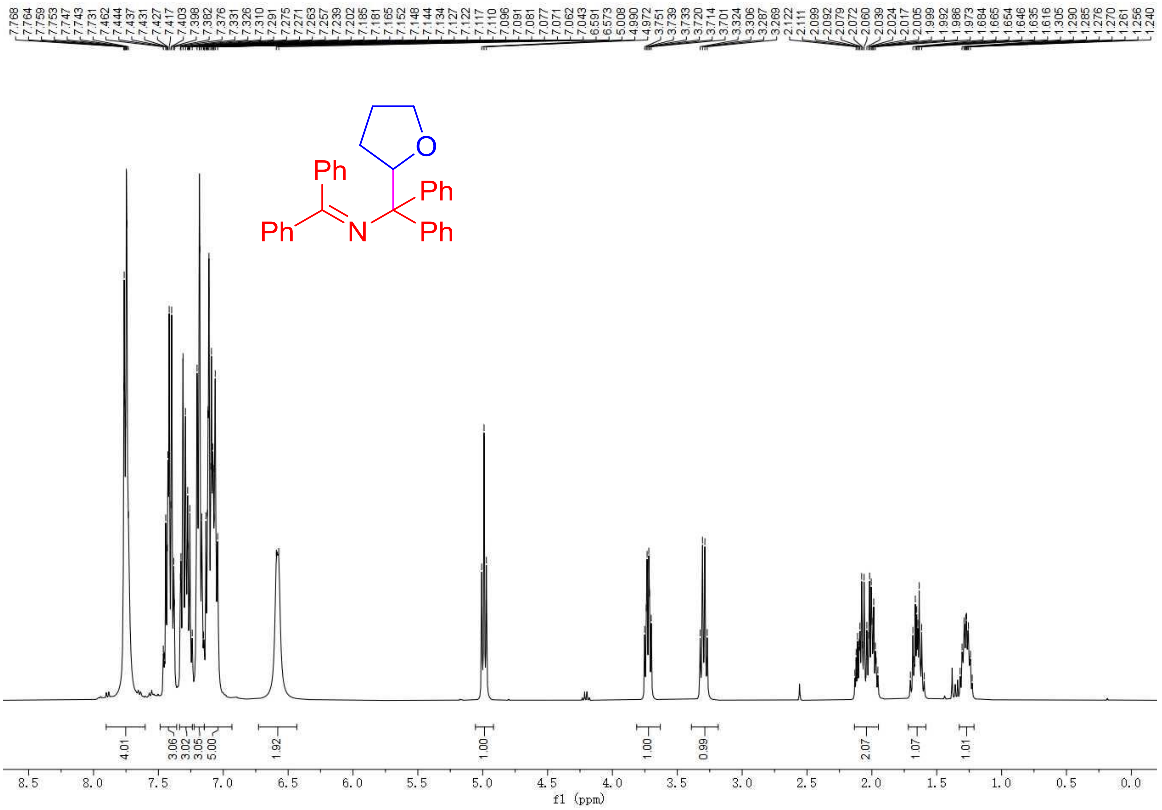

Figure S72. **^{13}C{^{1}H} NMR spectra (100 MHz, Chloroform-*d*) of *N*-(diphenyl(tetrahydrofuran-2-yl)methyl)-1,1-diphenylmethanimine (4ka):**

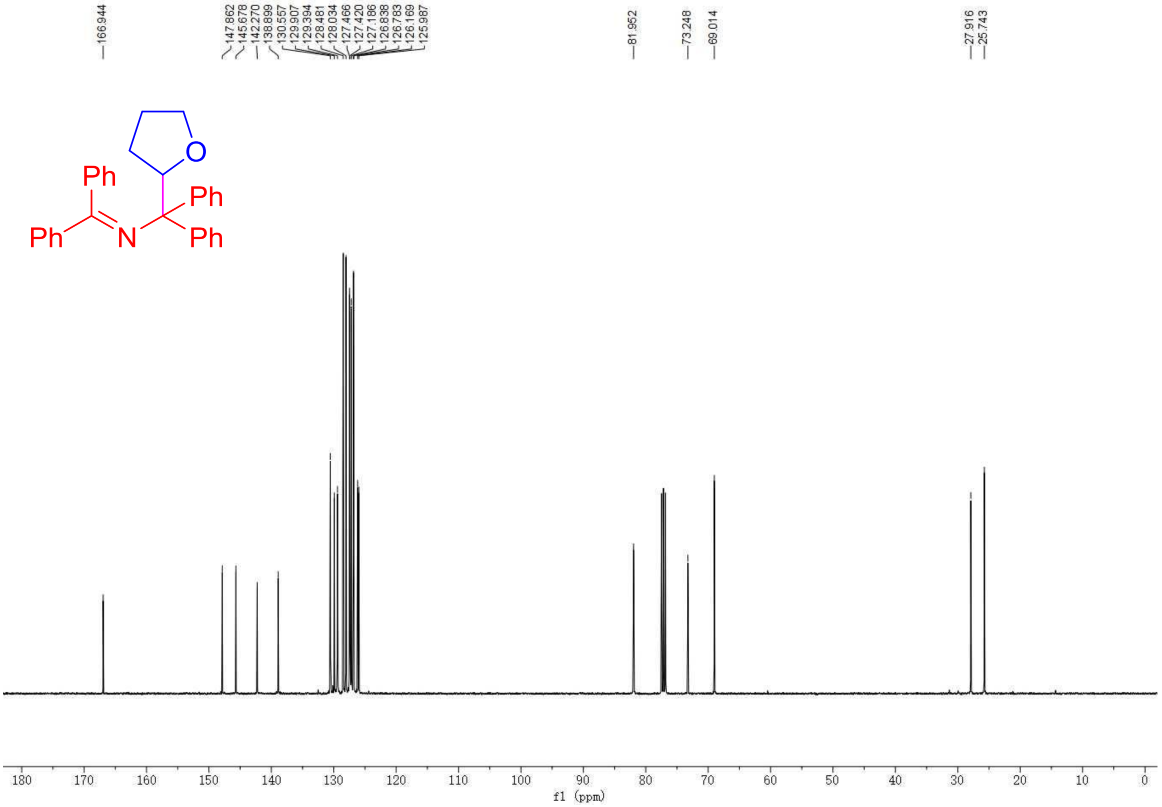

Figure S73. ^{1}**H NMR spectra (400 MHz, Chloroform-*d*) of *N*-((1,4-dioxan-2-yl)diphenylmethyl)-1,1-diphenylmethanimine (4kc):**

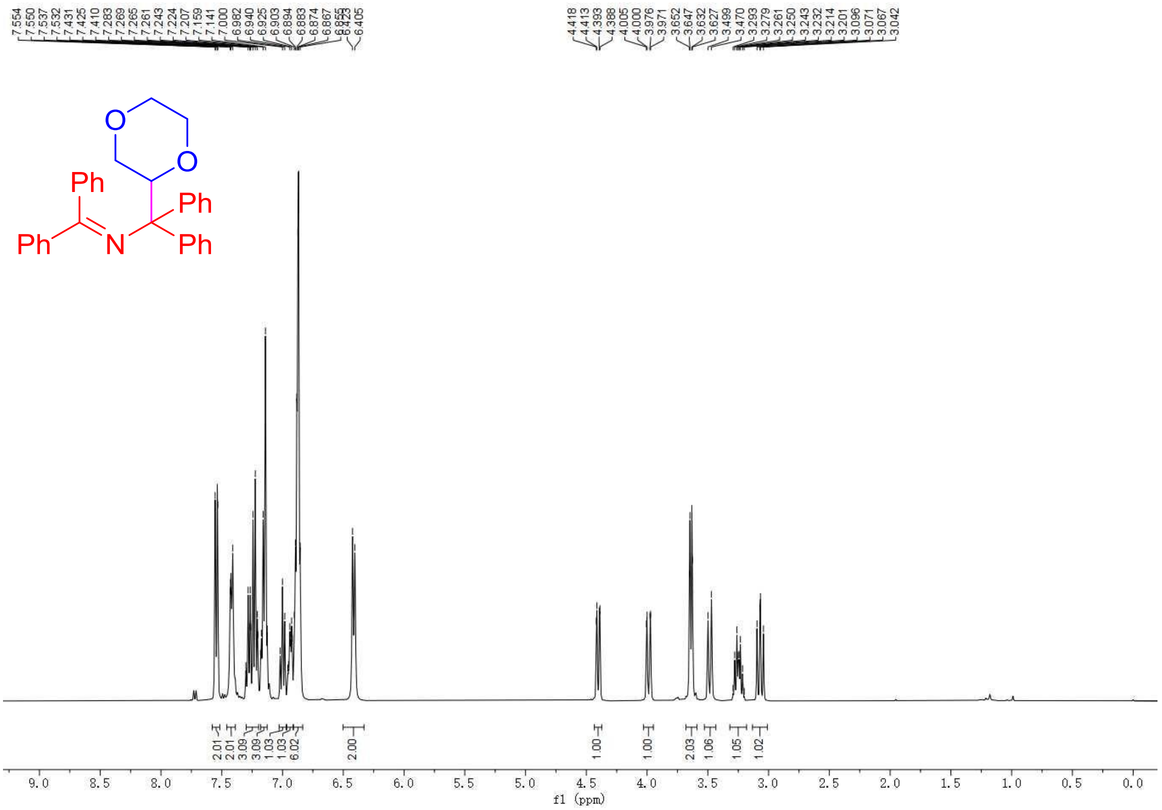

Figure S74. ^{13}**C{**^{1}**H} NMR spectra (100 MHz, Chloroform-*d*) of *N*-((1,4-dioxan-2-yl)diphenylmethyl)-1,1-diphenylmethanimine (4kc):**

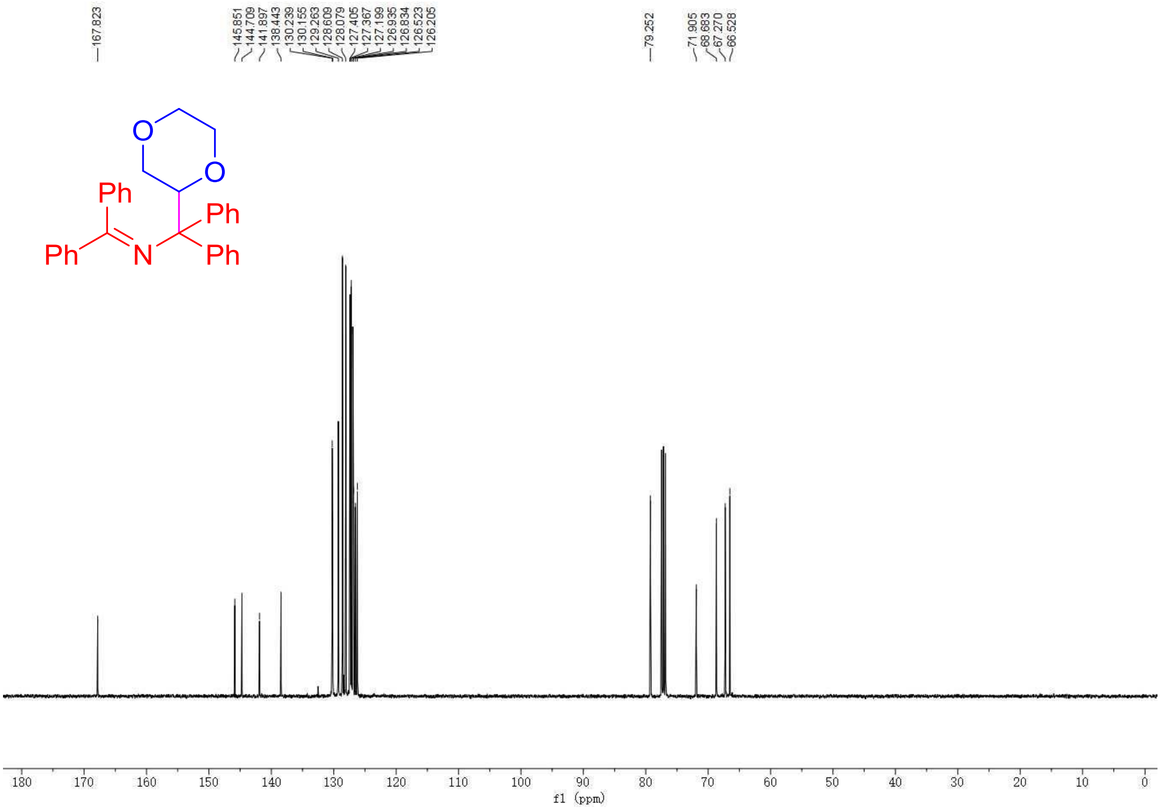

Figure S75. **^{1}H NMR spectra (400 MHz, Chloroform-*d*) of *N*-(1,2-diphenylethyl)-1,1-diphenylmethanimine (6aa):**

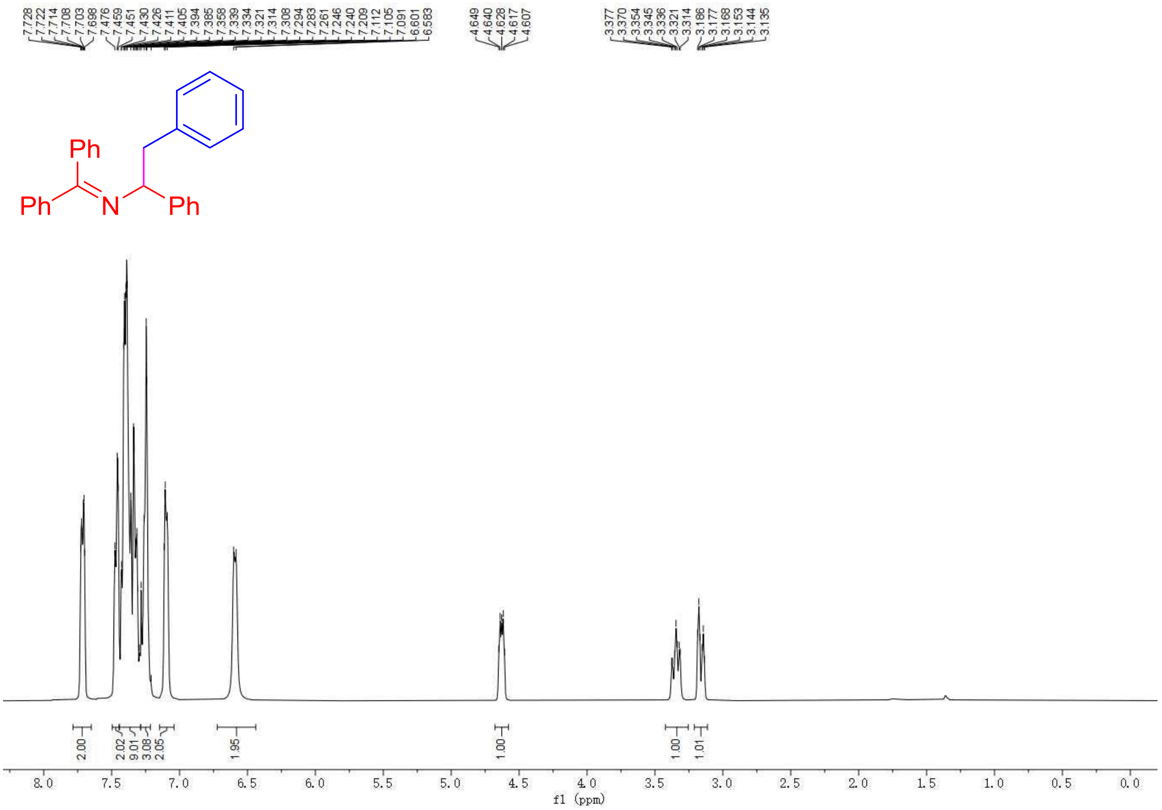

Figure S76. $^{13}C\{^{1}H\}$ NMR spectra (100 MHz, Chloroform-*d*) of *N*-(1,2-diphenylethyl)-1,1-diphenylmethanimine (6aa):

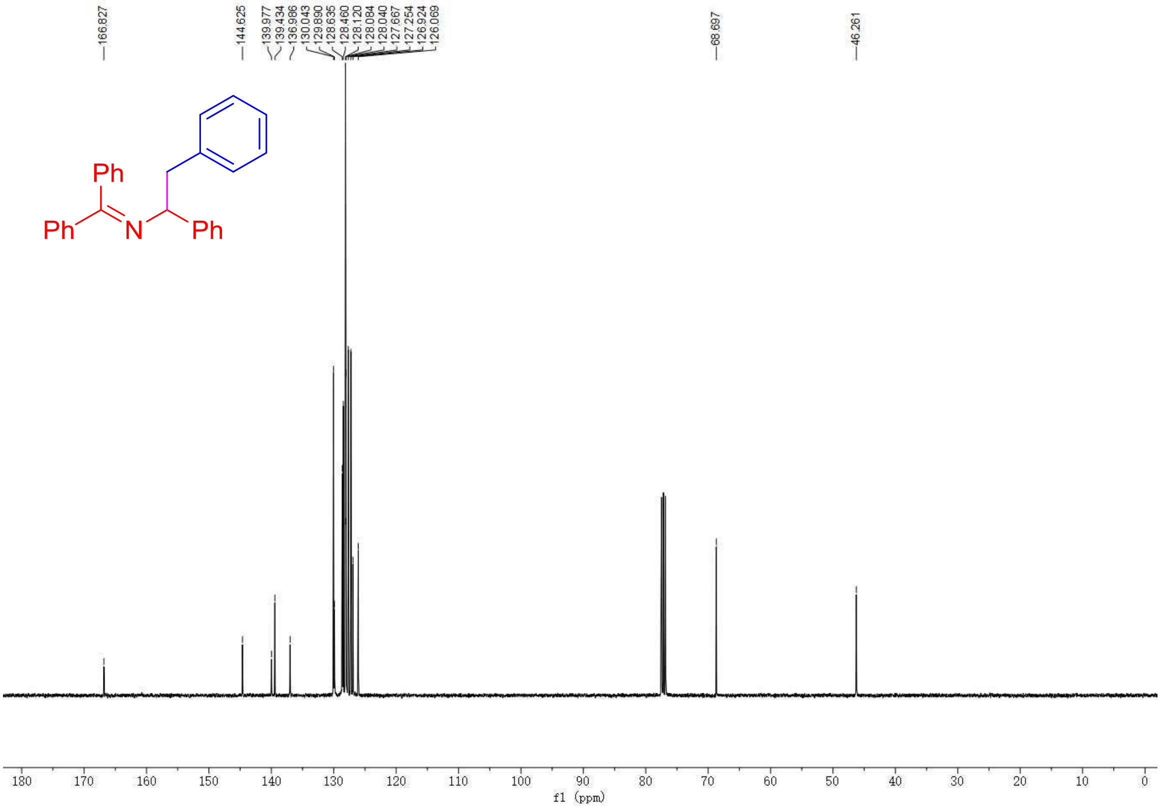

Figure S77. ^{1}H NMR spectra (400 MHz, Chloroform-*d*) of 1,1-diphenyl-*N*-(1-phenyl-2-(*o*-tolyl)ethyl)methanimine (**6ab**):

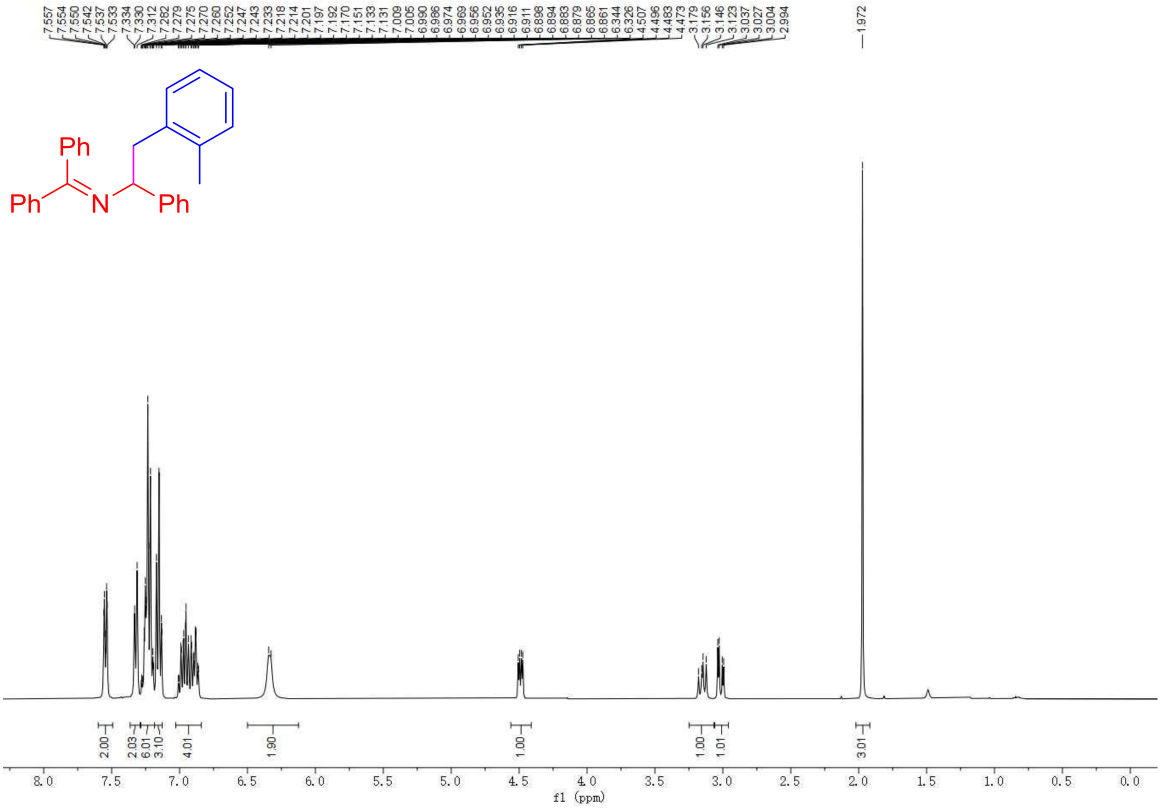

Figure S78. ^{13}C{^{1}H} NMR spectra (100 MHz, Chloroform-*d*) of 1,1-diphenyl-*N*-(1-phenyl-2-(*o*-tolyl)ethyl)methanimine (**6ab**):

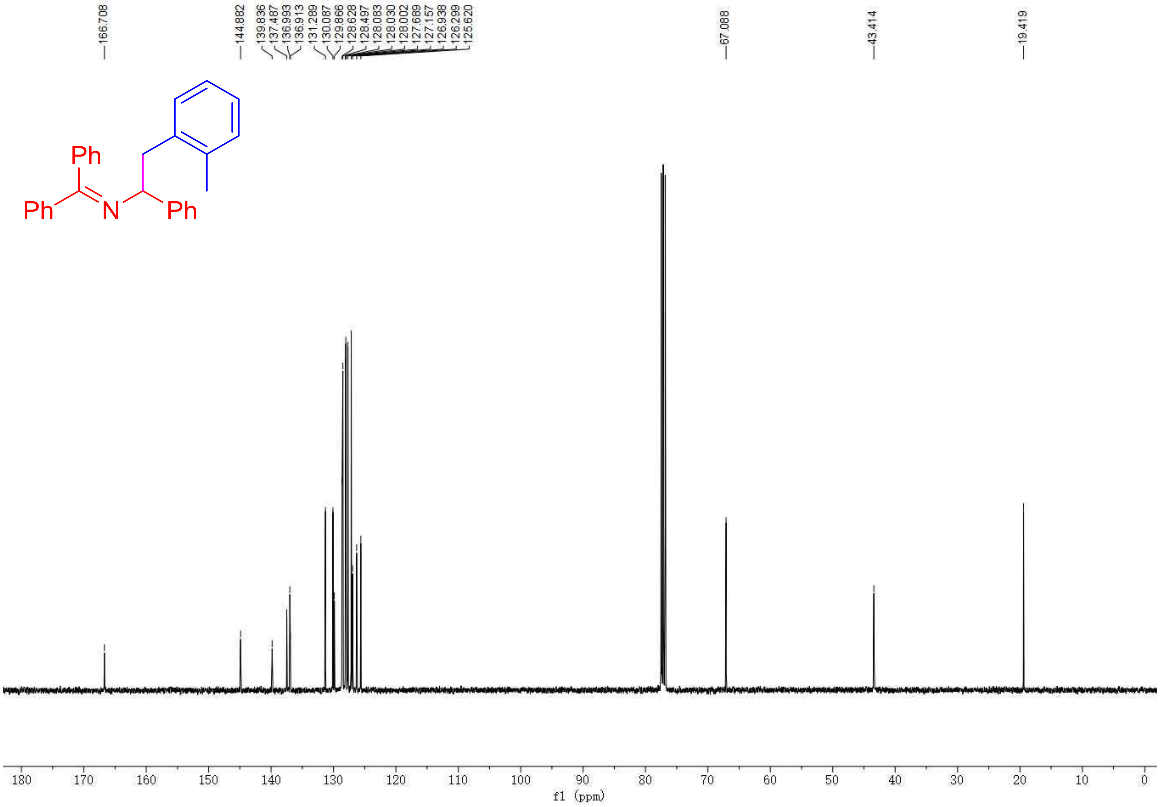

Figure S79. ^{1}H **NMR spectra (400 MHz, Chloroform-*d*) of 1,1-diphenyl-*N*-(1-phenyl-2-(*m*-tolyl)ethyl)methanimine (6ac):**

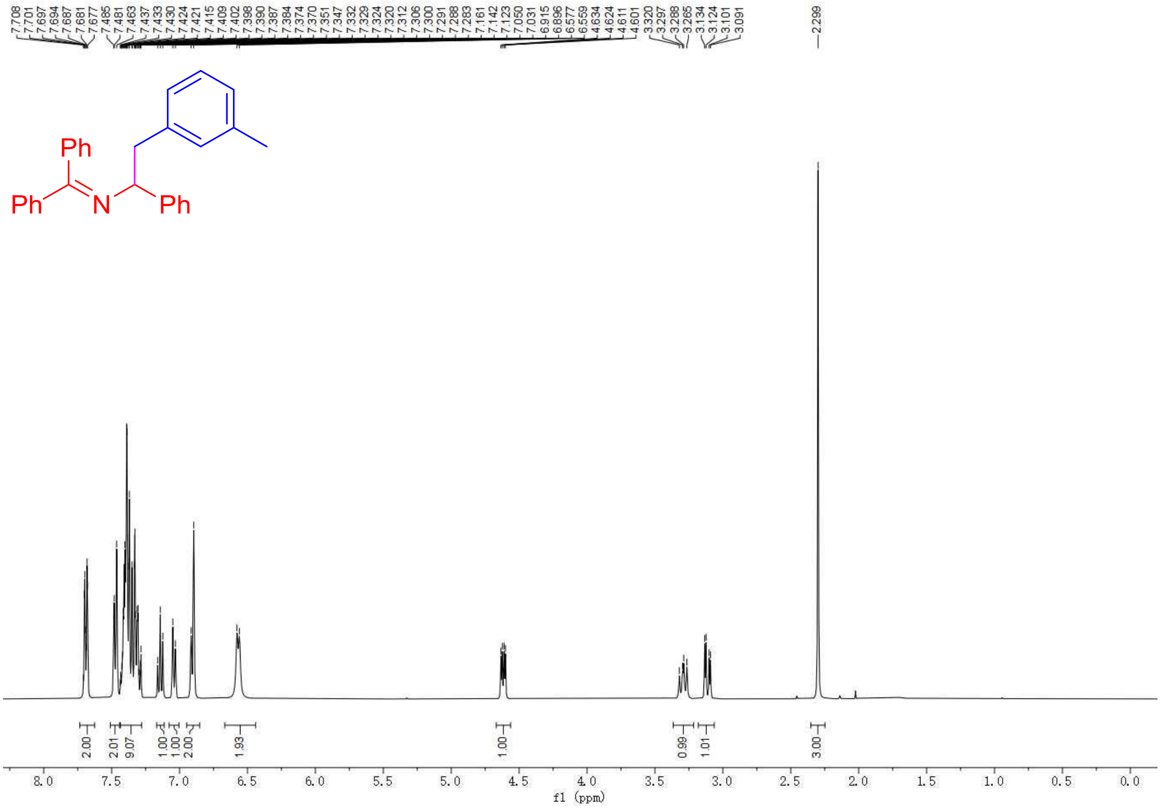

Figure S80. ^{13}C{^{1}H} **NMR spectra (100 MHz, Chloroform-*d*) of 1,1-diphenyl-*N*-(1-phenyl-2-(*m*-tolyl)ethyl)methanimine (6ac):**

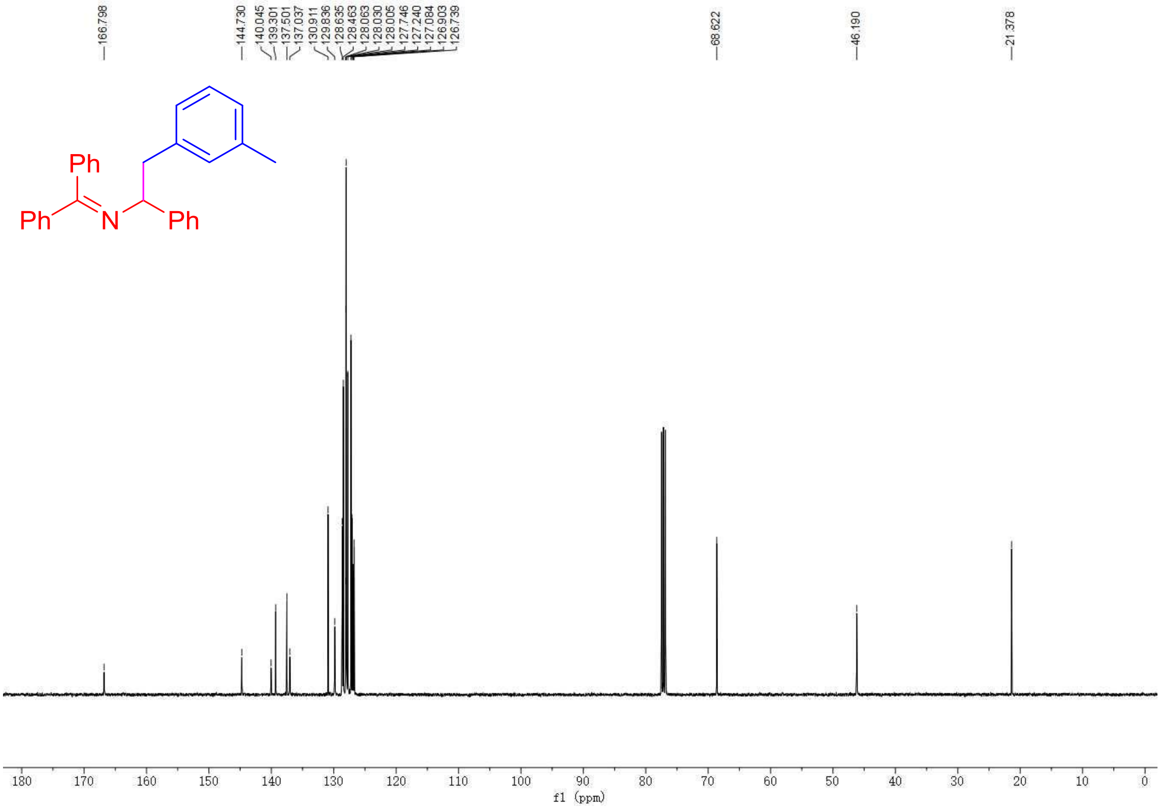

Figure S81. ^{1}**H NMR spectra (600 MHz, Chloroform-*d*) of 1,1-diphenyl-*N*-(1-phenyl-2-(*p*-tolyl)ethyl)methanimine (6ad):**

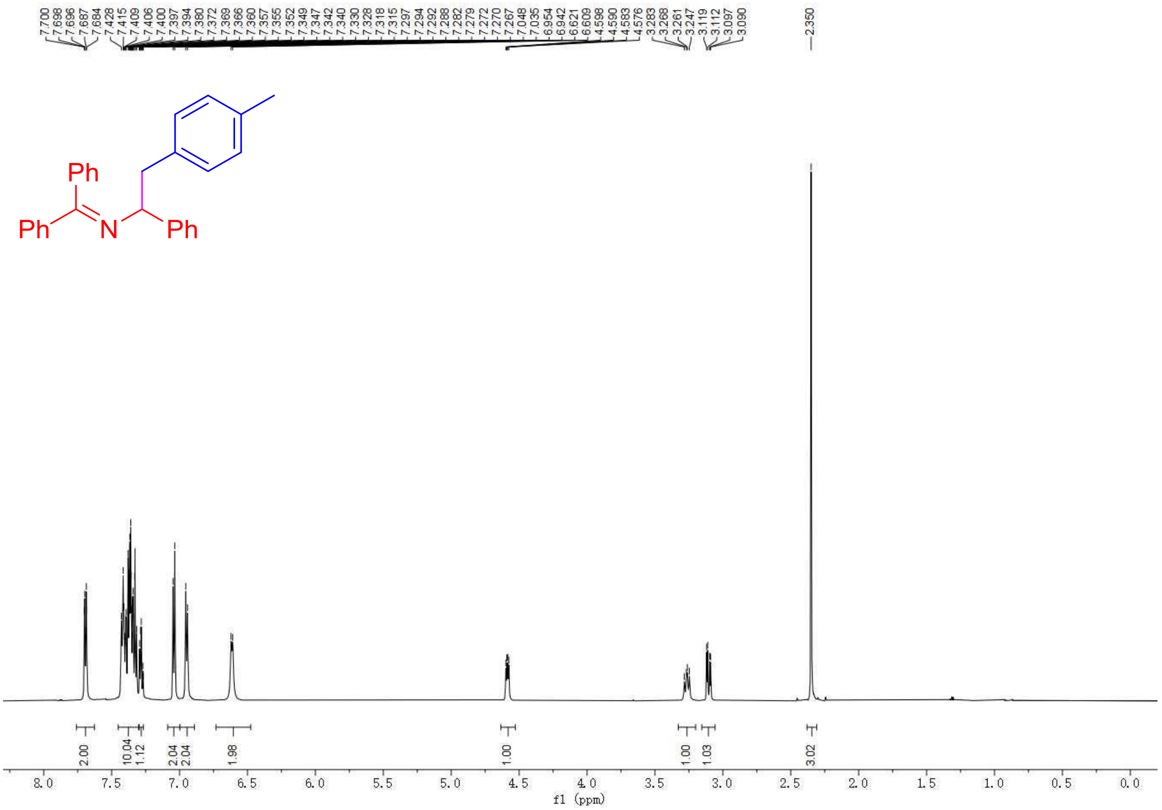

Figure S82. ^{13}**C{**^{1}**H} NMR spectra (150 MHz, Chloroform-*d*) of 1,1-diphenyl-*N*-(1-phenyl-2-(*p*-tolyl)ethyl)methanimine (6ad):**

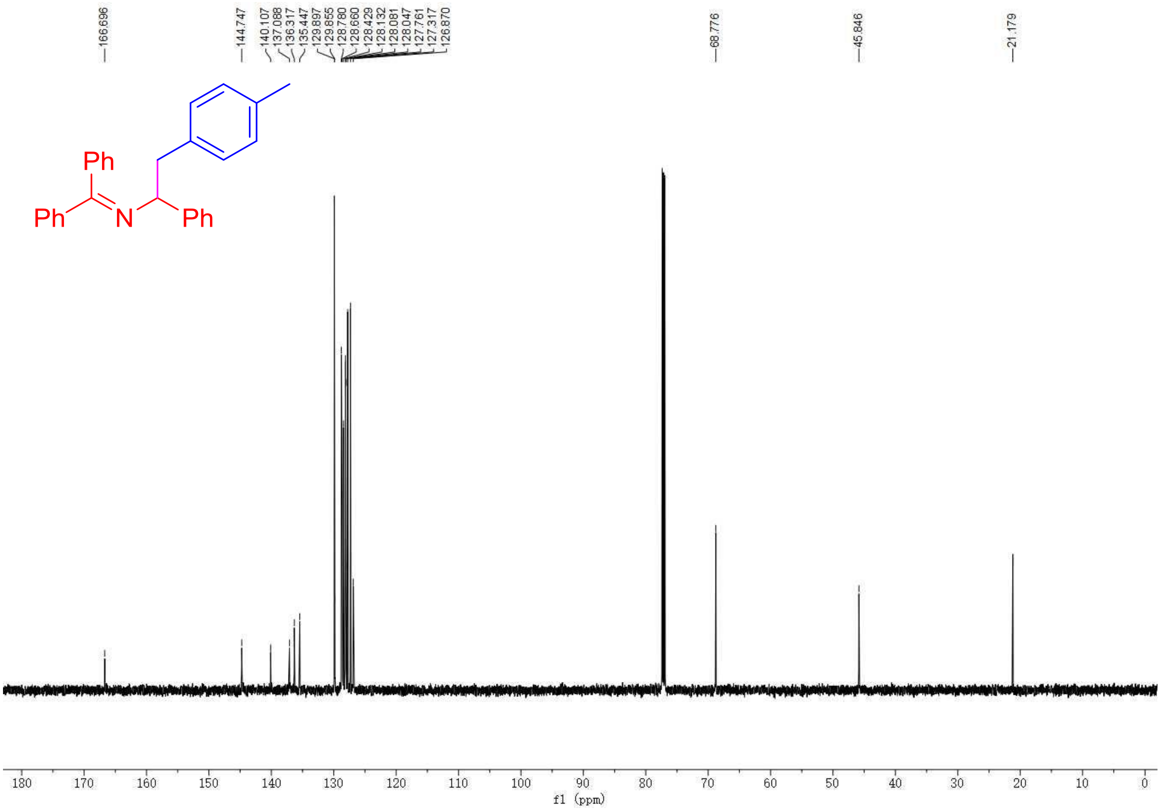

Figure S83. **^{1}H NMR spectra (600 MHz, Chloroform-*d*) of *N*-(2-(3,5-dimethylphenyl)-1-phenylethyl)-1,1-diphenylmethanimine (6ae):**

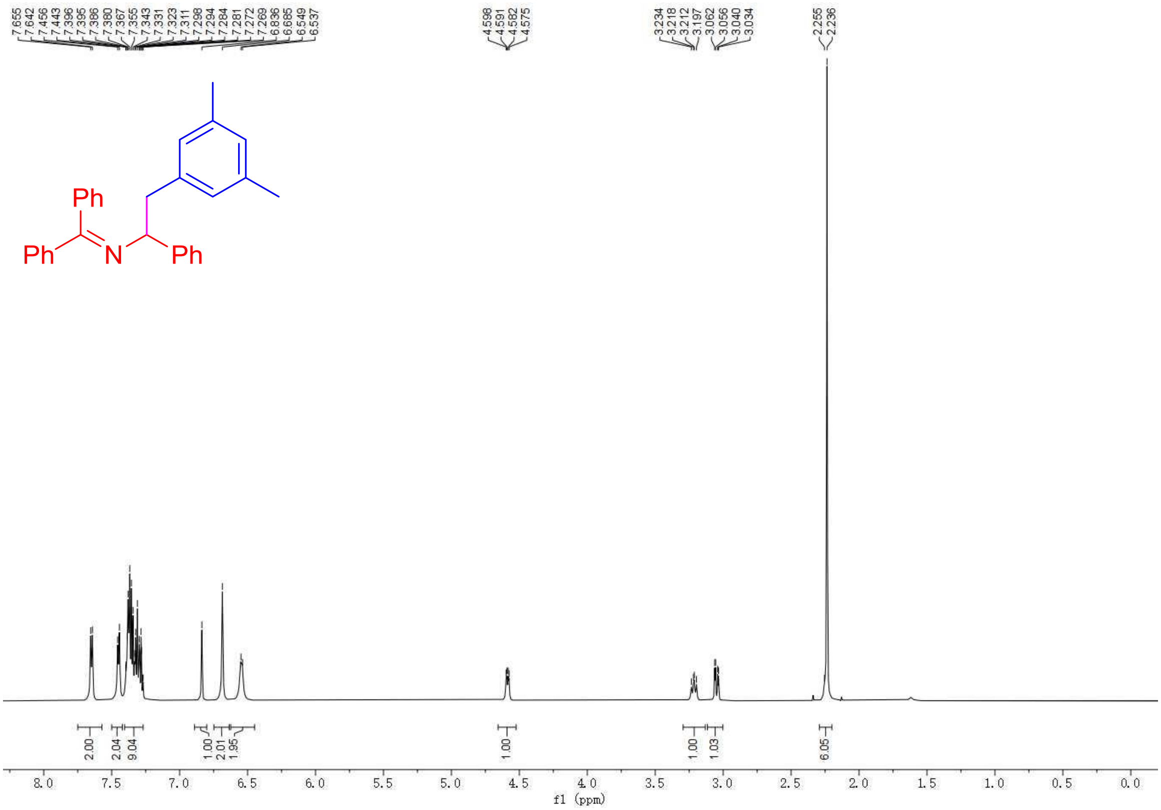

Figure S84. **$^{13}C\{^{1}H\}$ NMR spectra (150 MHz, Chloroform-*d*) of *N*-(2-(3,5-dimethylphenyl)-1-phenylethyl)-1,1-diphenylmethanimine (6ae):**

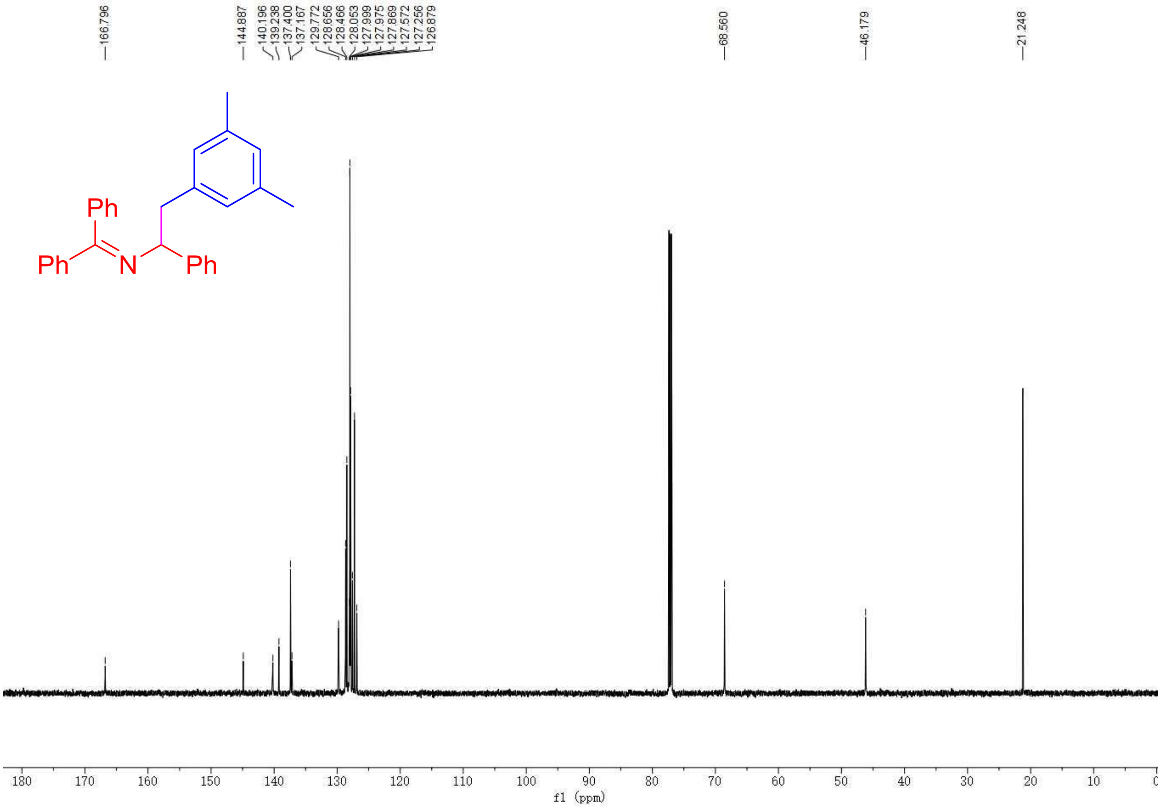

Figure S85. ^{1}H **NMR spectra (400 MHz, Chloroform-*d*) of *N*-(2-(naphthalen-1-yl)-1-phenylethyl)-1,1-diphenylmethanimine (6af):**

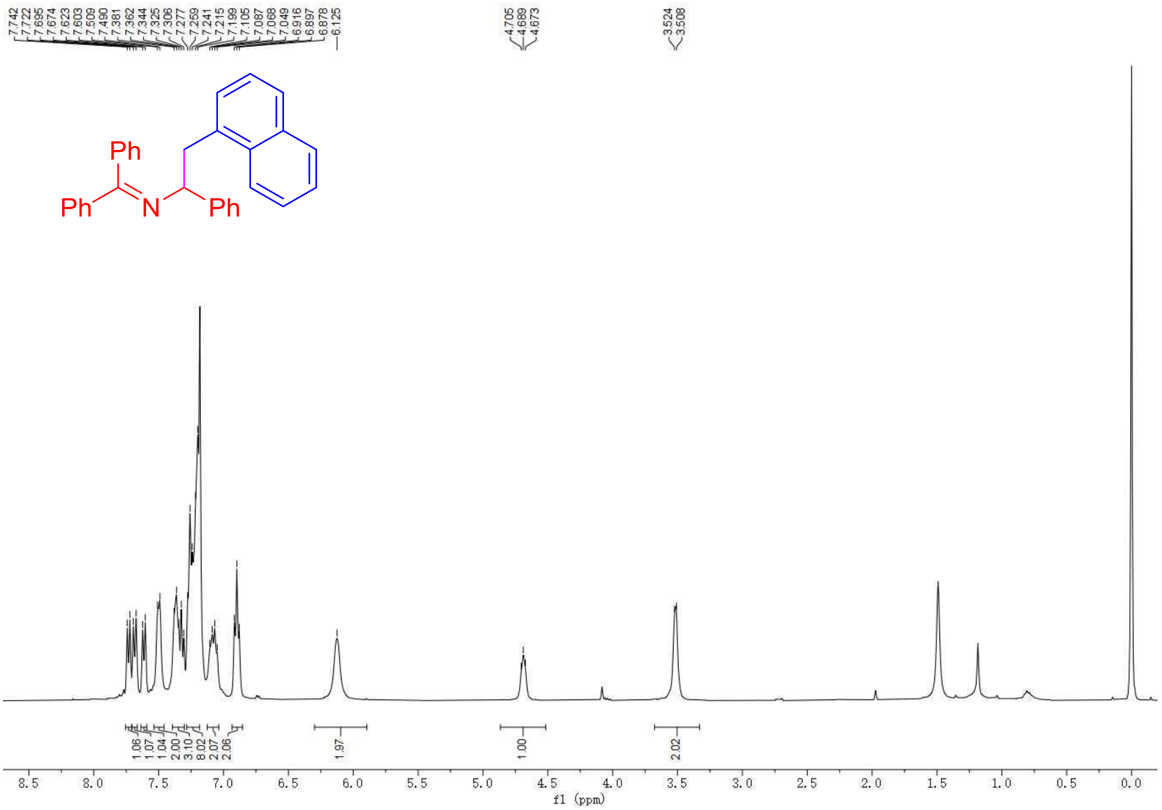

Figure S86. $^{13}C\{^{1}H\}$ **NMR spectra (100 MHz, Chloroform-*d*) of *N*-(2-(naphthalen-1-yl)-1-phenylethyl)-1,1-diphenylmethanimine (6af):**

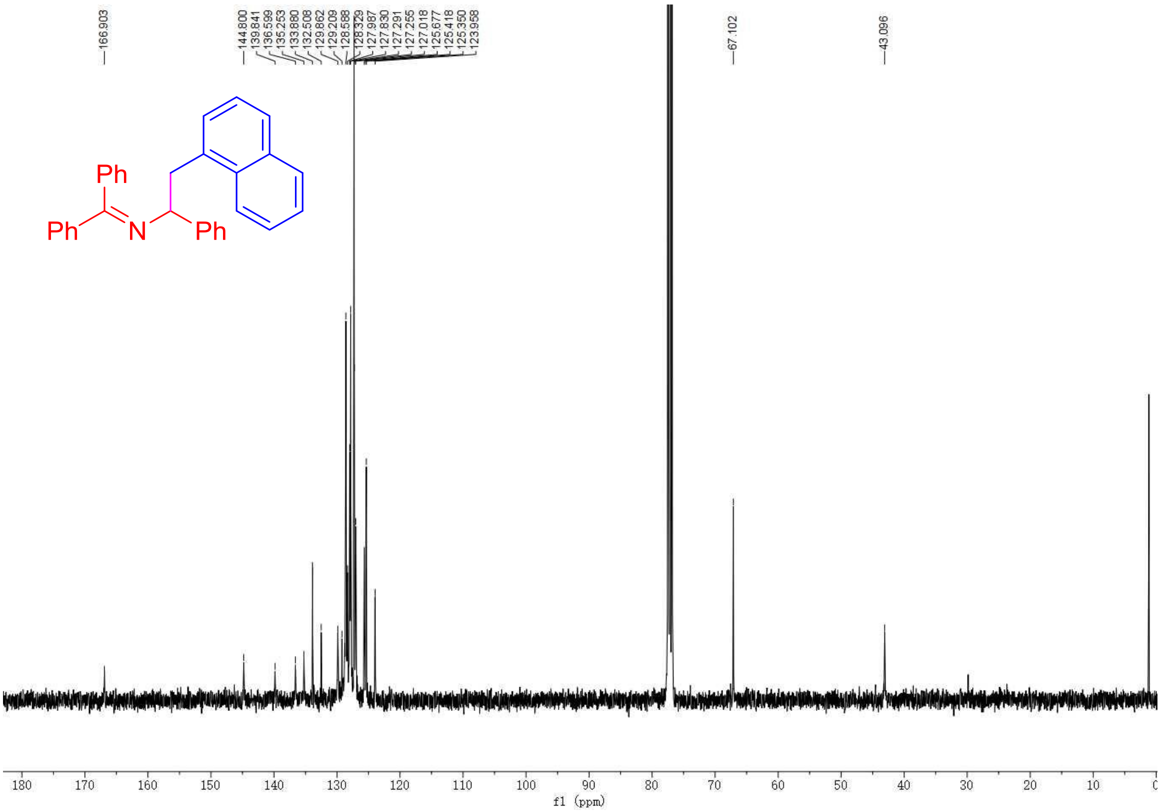

Figure S87. ^{1}H **NMR spectra (400 MHz, Chloroform-*d*) of *N*-(1,2-di-*p*-tolylethyl)-1,1-diphenylmethanimine (6ld):**

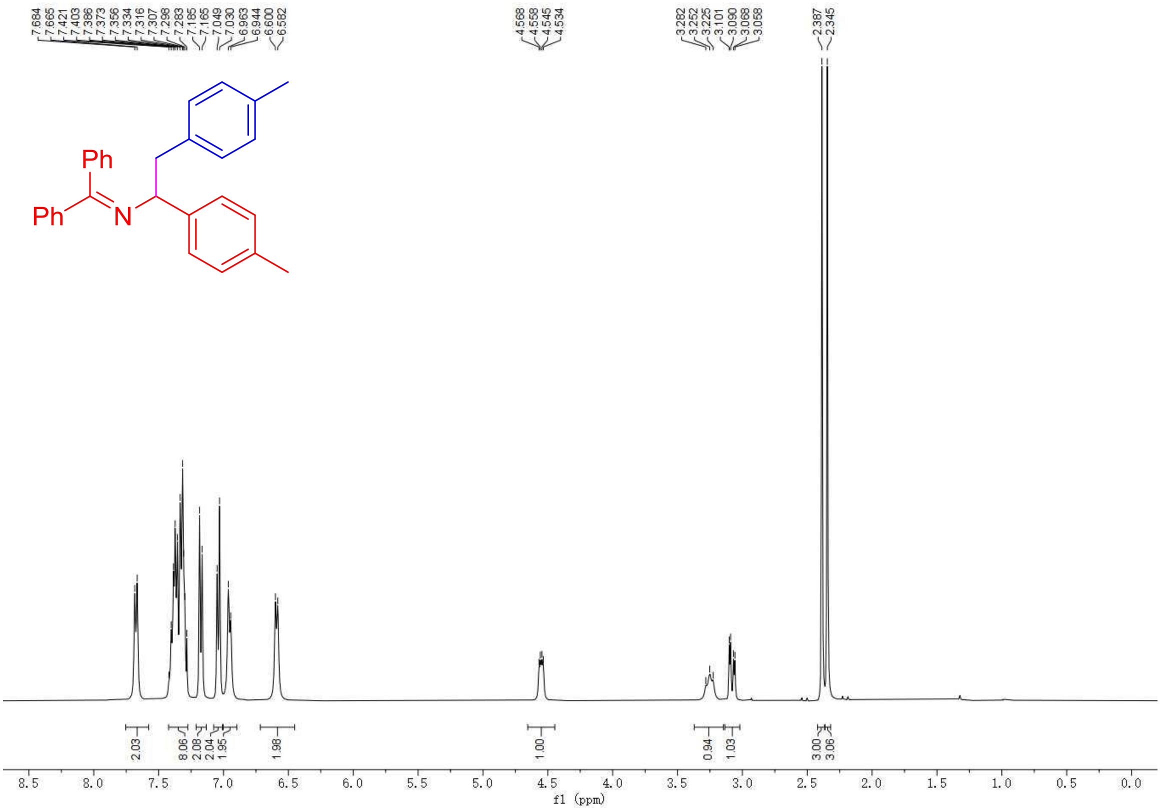

Figure S88. $^{13}C\{^{1}H\}$ **NMR spectra (100 MHz, Chloroform-*d*) of *N*-(1,2-di-*p*-tolylethyl)-1,1-diphenylmethanimine (6ld):**

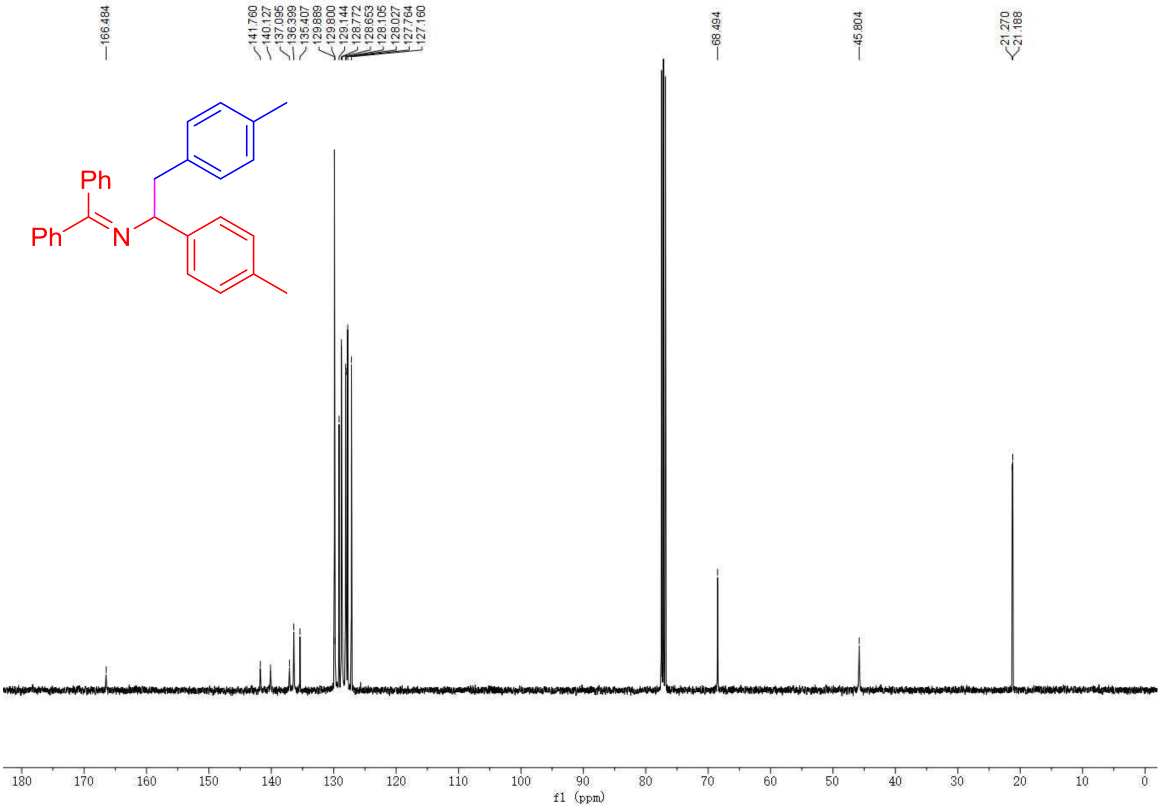

Figure S89. ^{1}**H NMR spectra (600 MHz, Chloroform-*d*) of *N*-(1-(4-methoxyphenyl)-2-(*p*-tolyl)ethyl)-1,1-diphenylmethanimine (6cd):**

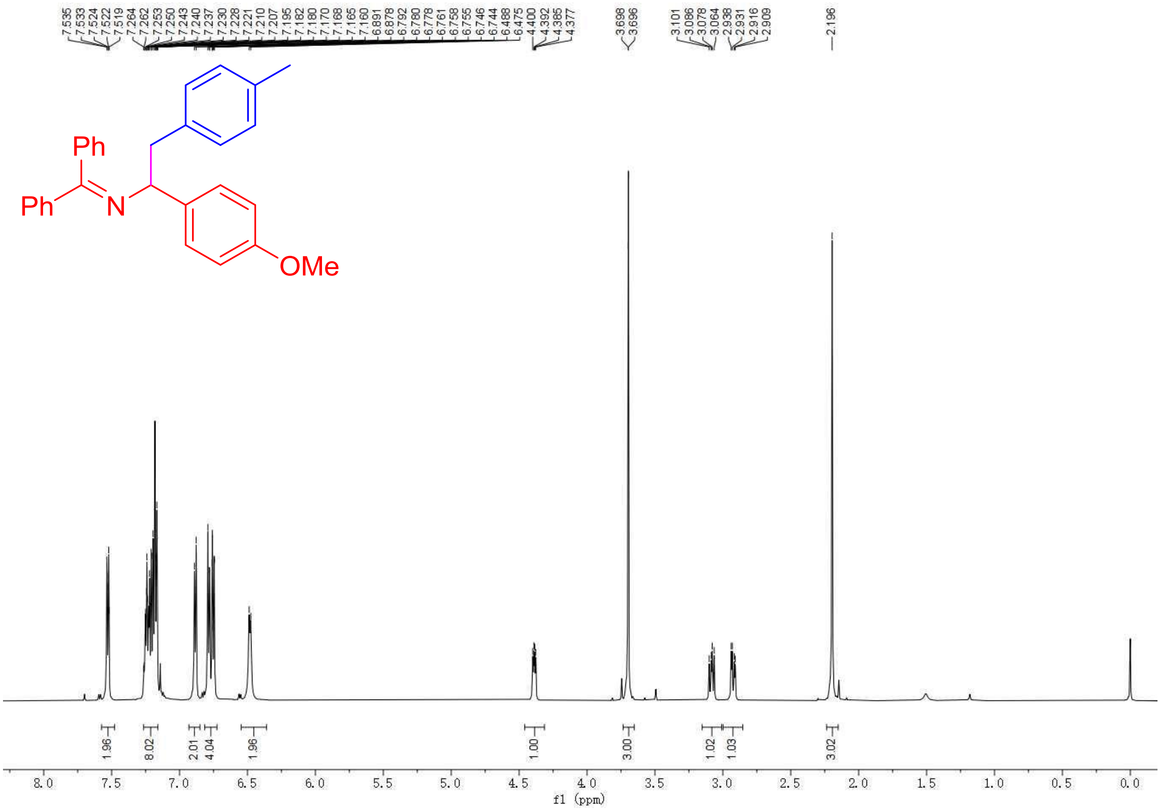

Figure S90. ^{13}**C{**^{1}**H} NMR spectra (150 MHz, Chloroform-*d*) of *N*-(1-(4-methoxyphenyl)-2-(*p*-tolyl)ethyl)-1,1-diphenylmethanimine (6cd):**

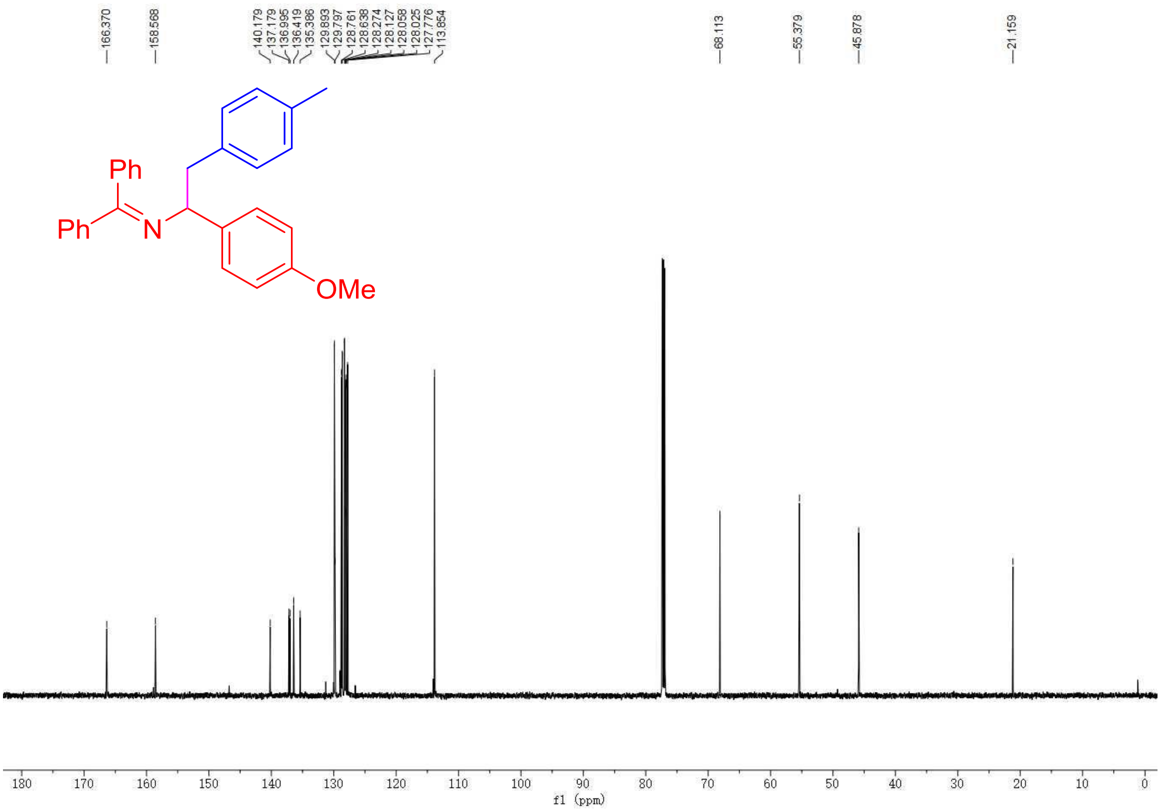

Figure S91. ^{1}**H NMR spectra (600 MHz, Chloroform-*d*) of 1,1-diphenyl-*N*-(1-(*o*-tolyl)-2-(*p*-tolyl)ethyl)methanimine (6jd):**

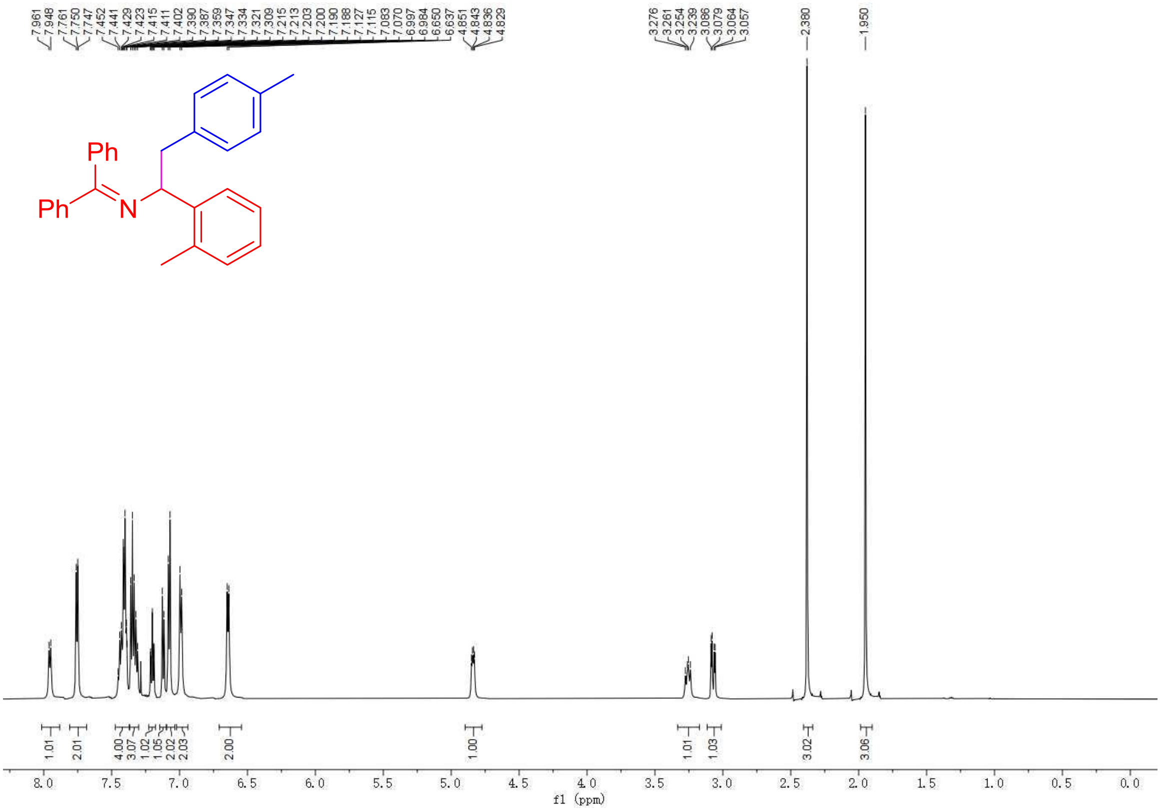

Figure S92. ^{13}**C{**^{1}**H} NMR spectra (150 MHz, Chloroform-*d*) of 1,1-diphenyl-*N*-(1-(*o*-tolyl)-2-(*p*-tolyl)ethyl)methanimine (6jd):**

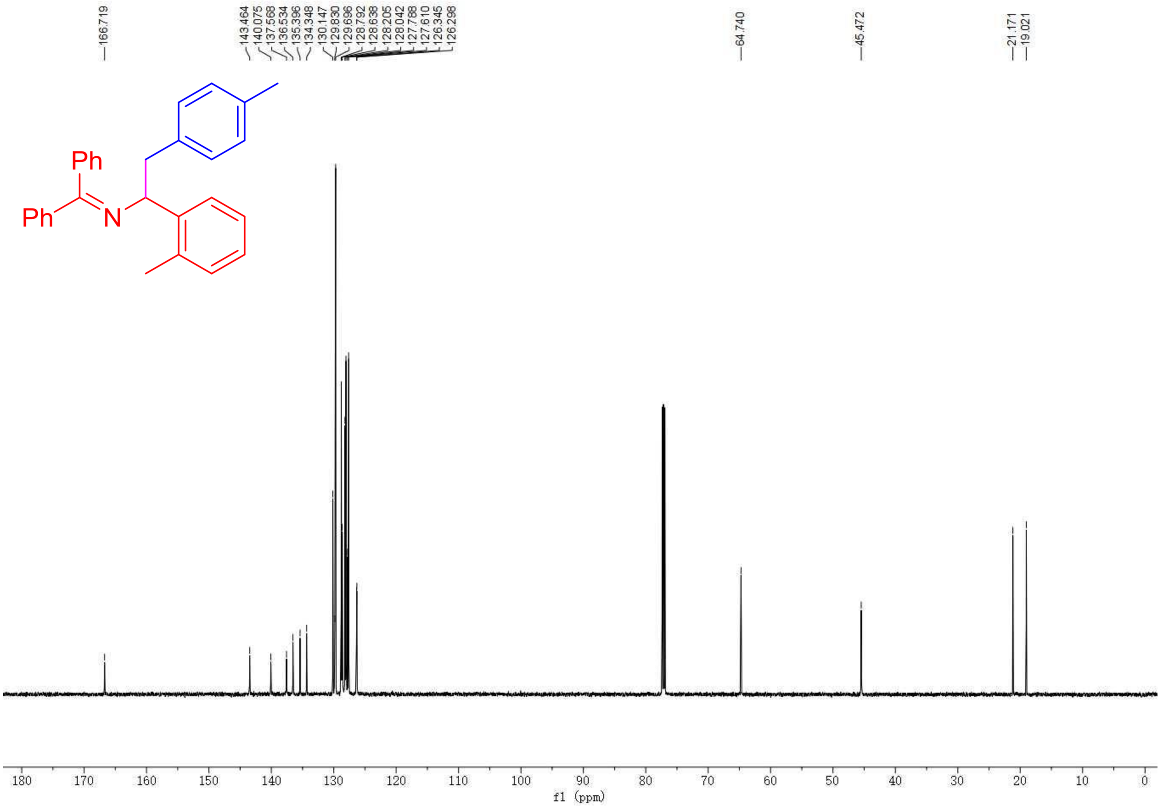

Figure S93. ^{1}**H NMR spectra (400 MHz, Chloroform-*d*) of 1,1-diphenyl-*N*-(phenyl(tetrahydrofuran-3-yl)methyl)methanimine (β-4aa,** dr = 1.5:1)**:**

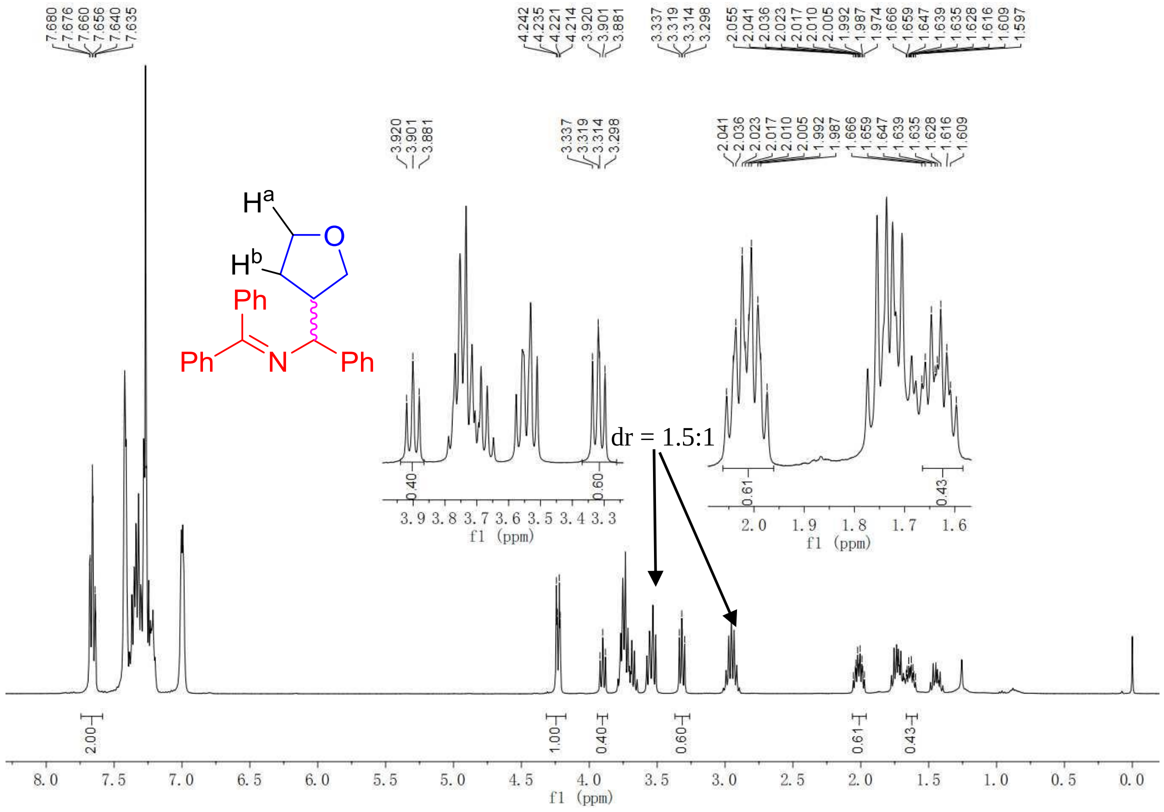

Figure S94. ^{13}**C{**^{1}**H} NMR spectra (100 MHz, Chloroform-*d*) of 1,1-diphenyl-*N*-(phenyl(tetrahydrofuran-3-yl)methyl)methanimine (β-4aa,** dr = 1.5:1)**:**

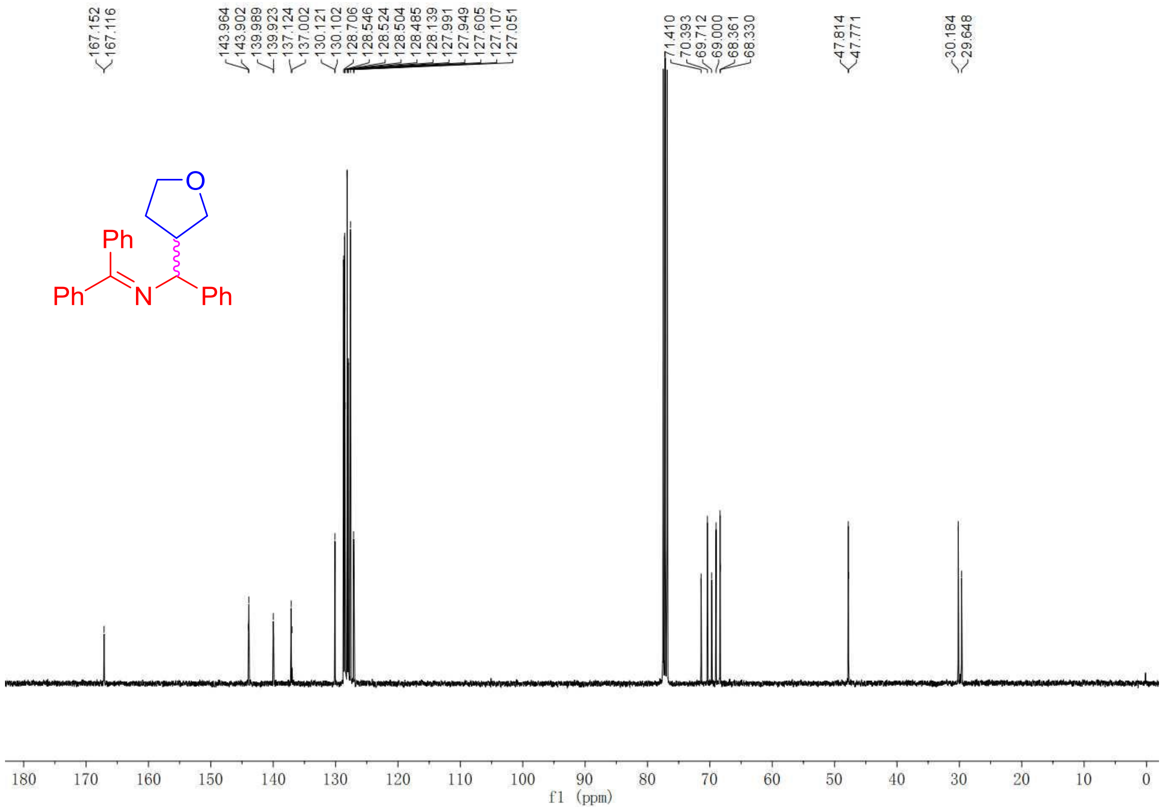

Figure S95. ^{1}H NMR spectra (400 MHz, Chloroform-*d*) of phenyl(tetrahydrofuran-2-yl)methanamine (7aa):

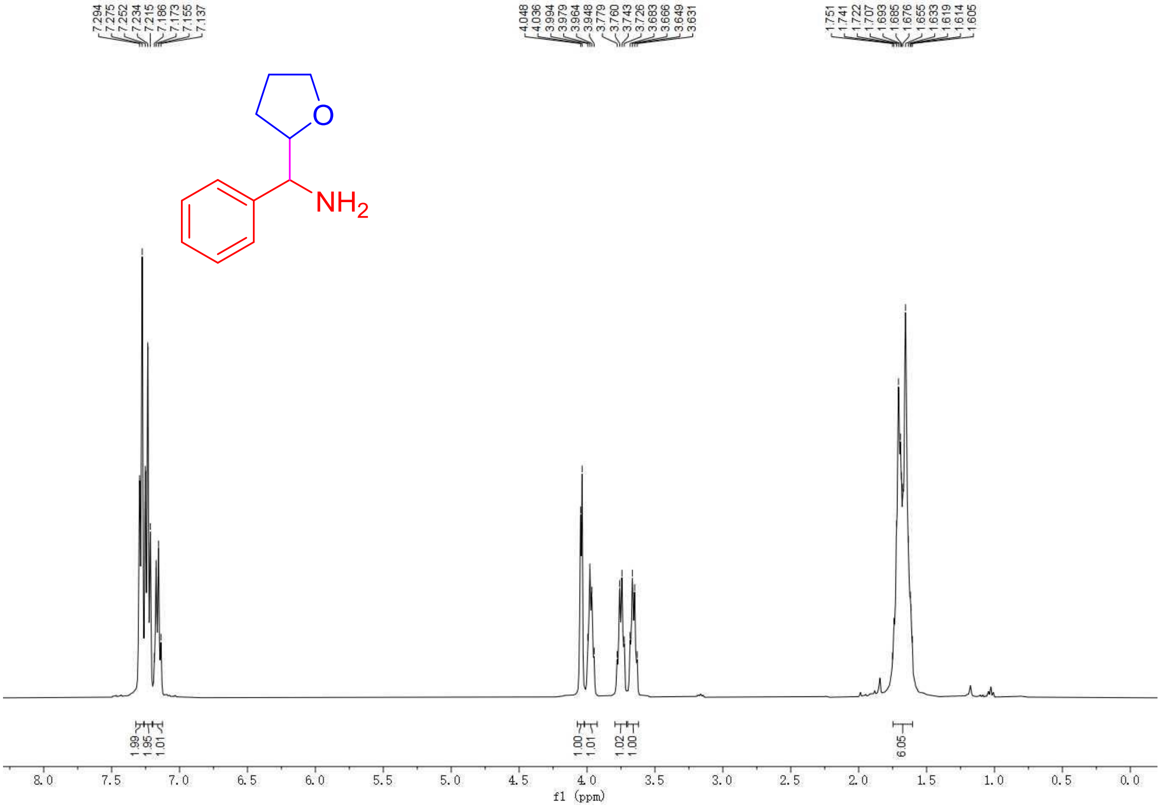

Figure S96. $^{13}C\{^{1}H\}$ NMR spectra (100 MHz, Chloroform-*d*) of phenyl(tetrahydrofuran-2-yl)methanamine (7aa):

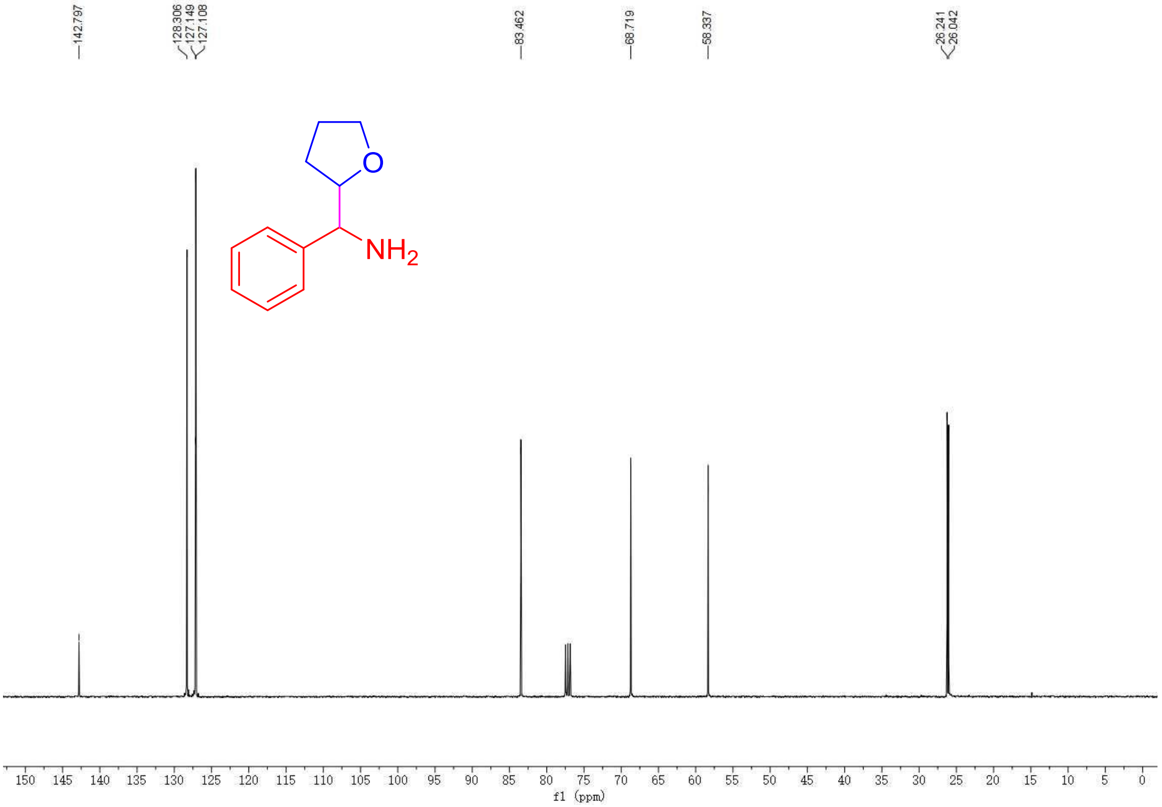

Figure S97. ^{1}**H NMR spectra (600 MHz, Chloroform-*d*) of 1-(*tert*-butyl)-3,5-dimethylbenzene-4-*d* (8):**

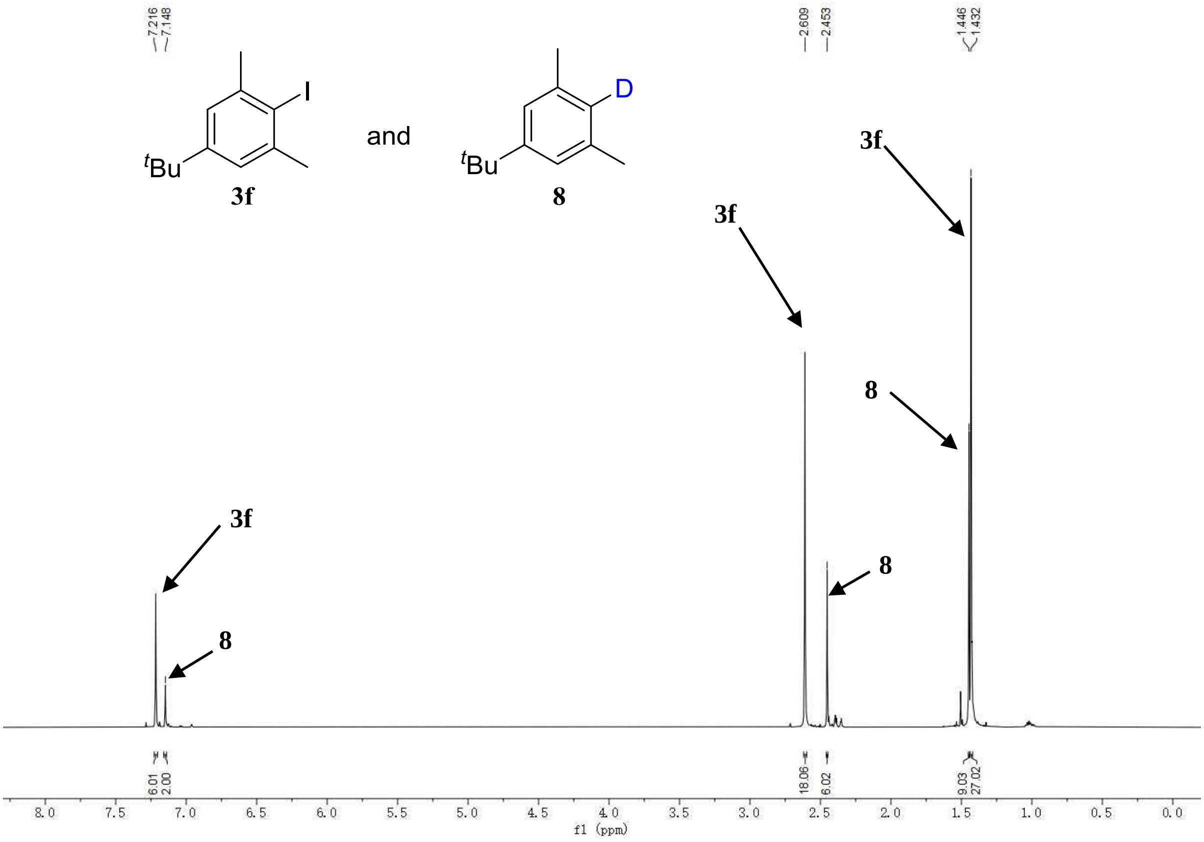

Figure S98. ^{13}**C{**^{1}**H} NMR spectra (150 MHz, Chloroform-*d*) of 1-(*tert*-butyl)-3,5-dimethylbenzene-4-*d* (8):**

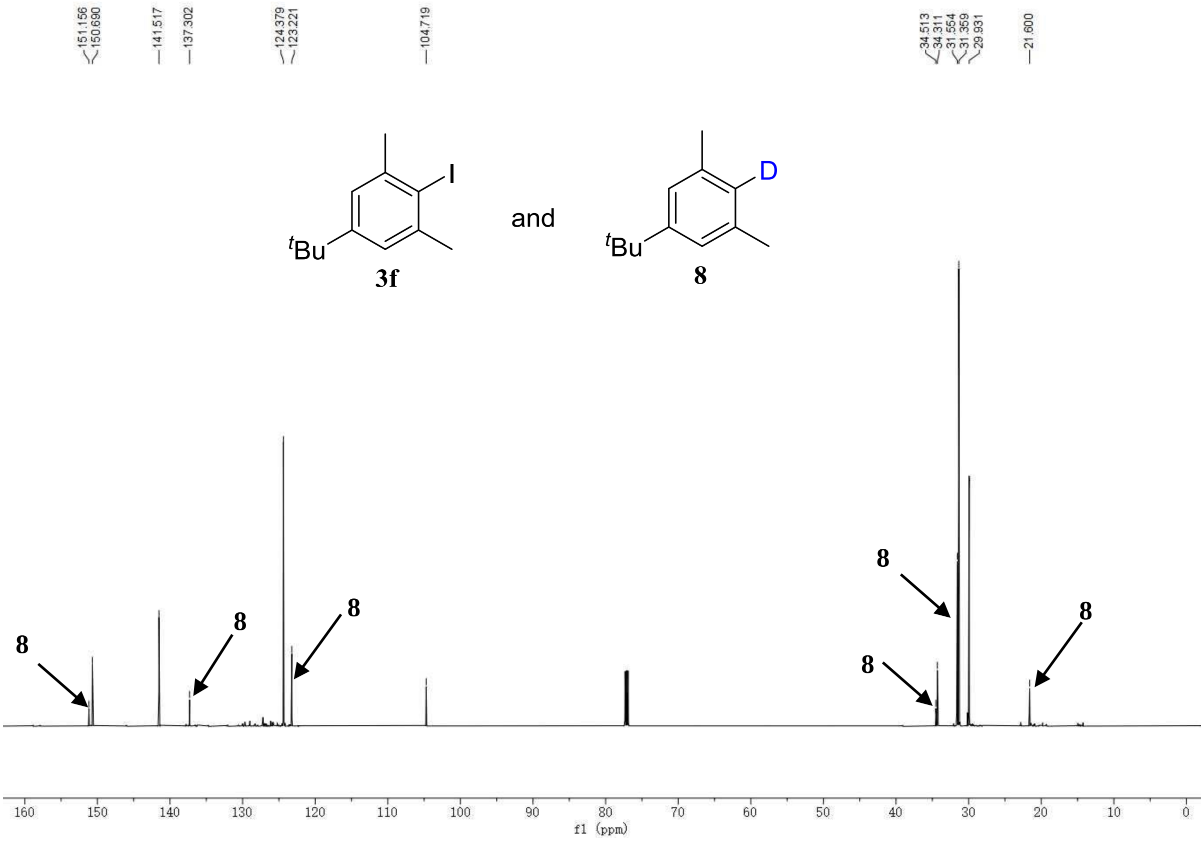

Figure S99. ^{1}**H NMR spectra (400 MHz, Chloroform-*d*) of 1,1-diphenyl-*N*-(phenyl(tetrahydrofuran-2-yl-*d*$_7$)methyl)methanimine** (D-**4aa**(major))**:**

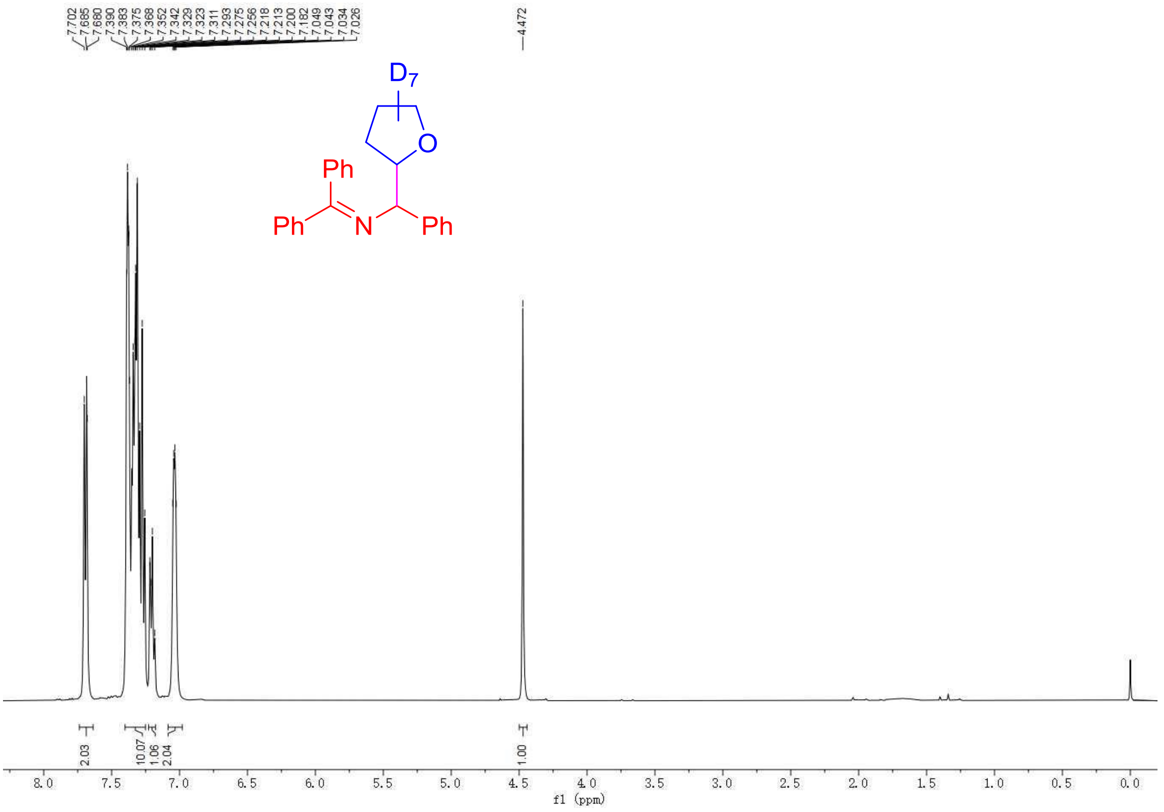

Figure S100. ^{13}**C{**^{1}**H} NMR spectra (100 MHz, Chloroform-*d*) of 1,1-diphenyl-*N*-(phenyl(tetrahydrofuran-2-yl-*d*$_7$)methyl)methanimine** (D-**4aa**(major))**:**

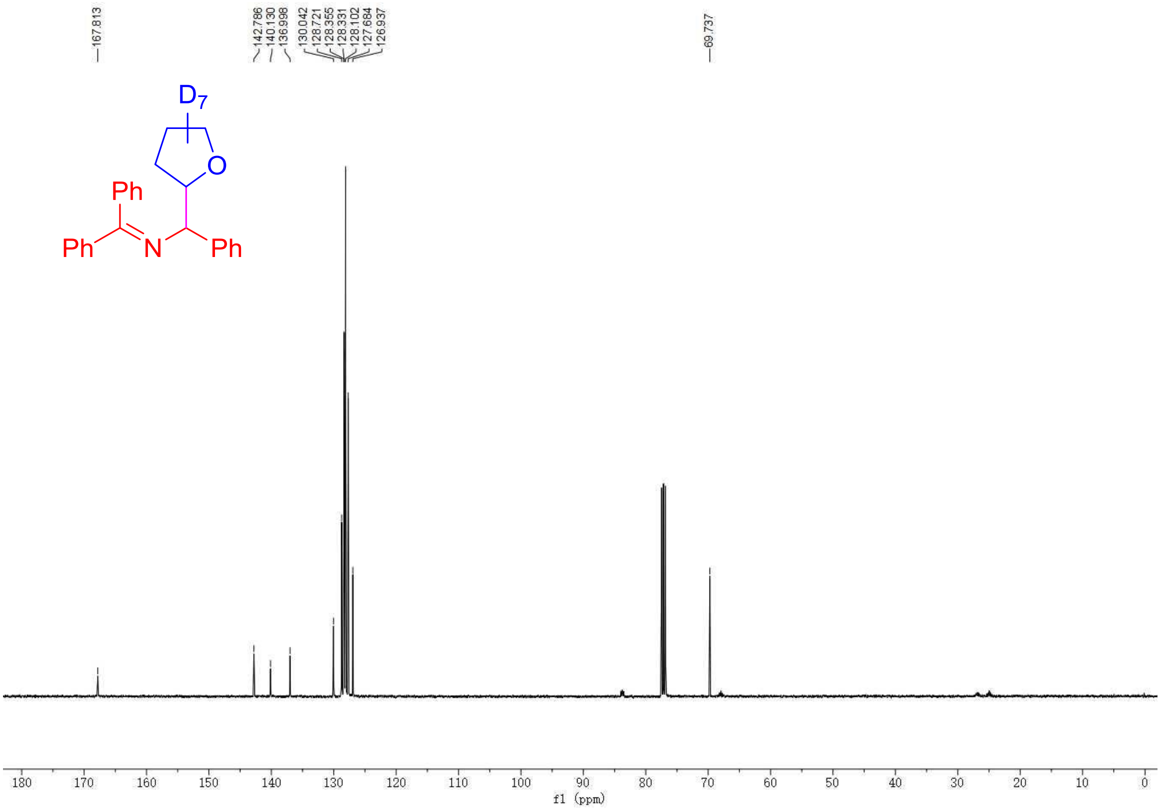

Figure S101. **^{1}H NMR spectra (400 MHz, Chloroform-*d*) of 1,1-diphenyl-*N*-(phenyl(tetrahydrofuran-2-yl-*d*$_7$)methyl)methanimine** (D-**4aa**(minor))**:**

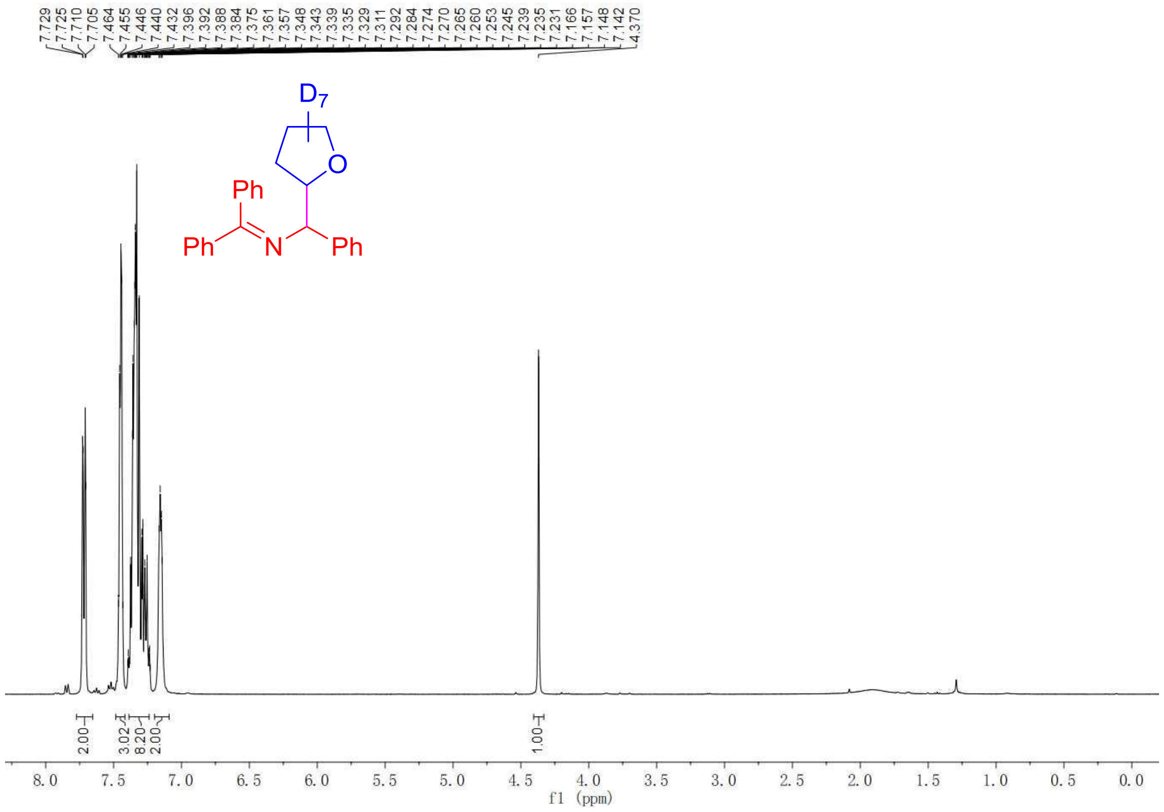

Figure S102. **^{13}C{^{1}H} NMR spectra (100 MHz, Chloroform-*d*) of 1,1-diphenyl-*N*-(phenyl(tetrahydrofuran-2-yl-*d*$_7$)methyl)methanimine** (D-**4aa**(minor))**:**

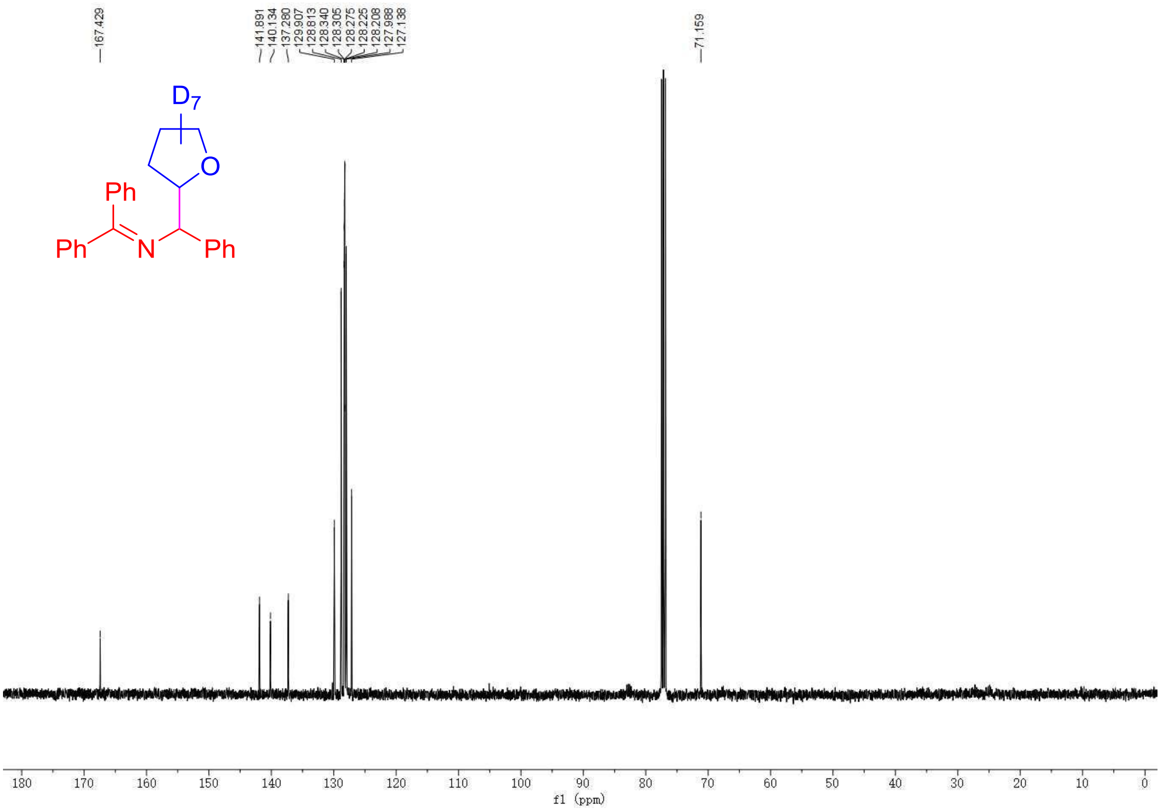

Supplement: SC-011-D0SC00031K-s001 [file SC-011-D0SC00031K-s001.pdf]
